# Circular RNAs are abundantly expressed and upregulated during repair of the damaged endometrium by Wharton’s jelly-derived mesenchymal stem cells

**DOI:** 10.1186/s13287-018-1046-3

**Published:** 2018-11-15

**Authors:** Baolan Sun, Lei Shi, Qin Shi, Yao Jiang, Zhangyao Su, Xiaoqing Yang, Yuquan Zhang

**Affiliations:** 1 Department of Obstetrics and Gynecology, Affiliated Hospital of Nantong University, Nantong, People’s Republic of China; 2 Department of Obstetrics and Gynecology, The Second Affiliated Hospital of Soochow University, Soochow, People’s Republic of China; 3 Department of Obstetrics and Gynecology, Affiliated Hospital of Nantong University School of Medicine, 19 Xishi Road, Nantong, 226006 Jiangsu People’s Republic of China

**Keywords:** Circular RNA, Endometrial stromal cells, Wharton’s jelly-derived mesenchymal stem cells, Repair

## Abstract

**Background:**

Wharton’s jelly-derived mesenchymal stem cells (WJ-MSCs) exhibit strong and powerful potential in repairing different diseases. The expression profile of circular RNA (circRNA) provides valuable insight for regulation of the repair process and exploration of reparative effect mechanisms.

**Methods:**

Human endometrial stromal cells (ESCs) were cultured with mifepristone to obtain damaged ESCs, which were then cocultured with or without WJ-MSCs (cocultured group versus non-cocultured group) to observe the reparative effect upon damaged ESCs by WJ-MSCs. CircRNA microarray was performed between the two groups. Based on the transcriptomics data, the differential gene expression profiles of the two groups were analyzed by Gene Ontology (GO), Kyoto Encyclopedia of Genes and Genomes (KEGG) pathway, and network analysis methods. Screening of a circRNA database was performed, and the results were confirmed by quantitative polymerase chain reaction (qPCR).

**Results:**

WJ-MSCs exerted a reparative effect upon damaged ESCs in the cocultured group such as improved cell morphology, higher proliferative ability, and lower apoptosis rate. CircRNA array showed that 7757 circRNAs were differentially expressed in ESCs from the cocultured group. Mitotic cell cycle, cell cycle process, and nuclear division ranked top in the GO upregulated list of the two groups, while DNA replication and cell cycle ranked top in the KEGG pathway analysis upregulated list of the two groups. The nine most aberrantly expressed circRNAs were selected for further verification in the same cohort of samples by microarray analysis. Seven of the nine most aberrantly circRNAs were confirmed to be significantly upregulated in the cocultured group. And four of the seven circRNAs (hsa_circ_0015825, hsa-circRNA4049-38, hsa-circRNA5028-15, and hsa_circ_0111659) expression both in ESCs and WJ-MSCs tended to decrease with time by qPCR. The levels of the remaining three circRNAs (hsa-circRNA8881-21, hsa_circ_0020492 and hsa_circ_ 0026141) did not change significantly over time in either ESCs or WJ-MSCs. Moreover, we focused on hsa_circRNA_0111659 and predicted its miRNAs and targeted mRNA. The association of circRNA-miRNA-mRNA is likely to be involved in regulating the repair of endometrial damage.

**Conclusions:**

Our results presented the abundant and upregulated circRNAs profile during repair of the damaged endometrium by WJ-MSCs and provided a novel perspective for circRNAs in the regulation of WJ-MSCs for endometrial repair.

**Electronic supplementary material:**

The online version of this article (10.1186/s13287-018-1046-3) contains supplementary material, which is available to authorized users.

## Background

The human endometrium not only maintains and affects normal menstruation but also plays a vital role in female fertility [1, 2]. Menopause or amenorrhea due to endometrial damage is one of the most important factors influencing infertility. Previous studies have found that the pathological mechanism underlying endometrial damage involves damage to the basal layer of the endometrium; this changes the regular growth and abscission of the endometrium during the normal menstrual cycle [3, 4]. The main route of endometrial repair after injury involves the repair or production of new basal layer cells via endogenous or exogenous pathways [5].

Human umbilical cord mesenchymal stem cells (WJ-MSCs), which exist in the umbilical cord Wharton’s jelly and the perivascular area, are increasingly being used in the field of regenerative medicine [6]. Studies have shown that WJ-MSCs exhibit a strong and powerful ability to repair different diseases, including cardiovascular disease, hematopathy, diabetes, muscular degeneration, liver disease, and endometrial damage [7–13]. Our previous studies showed that WJ-MSCs are able to ameliorate damaged human endometrial stromal cells (ESCs) both in vitro and in vivo [13, 14], thus indicating that it is the possible to use WJ-MSCs to repair endometrial damage. However, in vivo, we also found that a large number of WJ-MSCs (10^6^–10^7^) are required to achieve a repair effect. Because the acquisition, cultivation, and storage of WJ-MSCs require a substantial amount of manpower and material resources, it is imperative to improve the efficiency of working with WJ-MSCs. For example, it is important to investigate the regulatory mechanisms involved in the repair of ESCs by WJ-MSCs.

Circular RNA (circRNA) is a unique class of endogenous non-coding RNA (ncRNA) forming a closed continuous loop by back-splicing with covalently joined 3′- and 5′-ends. Because of their closed structure, circRNAs are very stable and are resistant to RNA degradation [15–17]. Most often, they are derived from annotated host gene exons, which may give rise to alternative circRNAs, depending on the exact exons involved in back-splicing; they also compose a large class of animal RNAs with developmental and tissue-specific expression patterns [18]. Recent studies have revealed that circRNAs regulate gene expression via a range of different mechanisms, specifically, by serving as miRNA sponges [19, 20], facilitating transcription of their host gene by directly associating with RNA polymerase II [21]. Furthermore, circRNAs are extensively involved in the pathological process of damage and repair induced by different causes, such as the priming phase of rat liver regeneration, neuronal injury or damage caused by chemical carcinogens [22–25]. However, whether circRNAs are involved in the process of WJ-MSC-mediated endometrial repair remains unclear, as do the specific mechanisms involved.

In this study, we aimed to explore the underlying role of circRNAs in the WJ-MSC-mediated repair of damaged ESCs and focused particularly on circRNAs vital for the repair of damaged ESCs. To do so, we used circRNA microarray to detect differentially expressed circRNA profiles in damaged ESCs cocultured with or without WJ-MSCs.

## Methods

### Cells and culture

To obtain WJ-MSCs, we collected human umbilical cords from healthy full-term deliveries. We also obtained endometrium samples from patients with intramural or pedunculated sub-serosal hysteromyoma without endometrial abnormalities to obtain ESCs. None of the women had taken medication or had received hormonal therapy for at least 6 months before undergoing hysterectomy. All samples were obtained after the patients provided signed informed consent, and the procedures were approved by the Ethics Committee of the Affiliated Hospital of Nantong University. Both WJ-MSCs and ESCs were isolated, cultured, and identified as previously described [26]. WJ-MSCs and ESCs were cultured in Dulbecco’s modified Eagle’s medium F12 (DMEM/F-12; Hyclone, Logan, UT, USA) containing 10% fetal bovine serum (FBS; Gibco, Waltham, MA, USA), 100 U/ml penicillin, and 100 μg/ml streptomycin at 37 °C in a humidified 5% CO_2_ atmosphere, respectively.

### ESCs treated with mifepristone and cocultured with WJ-MSCs

Establishment of the damaged-ESC model and the WJ-MSCs coculture system was performed according to our previous protocol [13]. In brief, the ESCs of passage 2 were dispensed into 24-well plates at a density of 1.5 × 10^4^ cells/well and cultured in DMEM-F12 with 10% FBS. After 24 h, this medium was changed to a medium containing 2% FBS for 12 h. Then, the cells were treated with mifepristone (M8046; Sigma-Aldrich, Saint Louis, MO, USA) at a concentration of 60 μmol/L. After 48 h, the medium was replaced by fresh medium (2% FBS) to allow culture to proceed, but without mifepristone. The Transwell system (24 mm Transwell with a 0.4-μm pore polycarbonate membrane insert, #3412; Corning) was used to establish the coculture system. WJ-MSCs from passage 3 were seeded on top of the artificial basement membrane and placed in the upper part of the tissue culture plate for coculture with damaged ESCs for 24 h, 48 h, and 72 h. Control plates were not seeded with WJ-MSCs. ESCs and WJ-MSCs were subsequently digested and collected for use. We took the damaged ESCs and cocultured these with WJ-MSCs as the cocultured group; damaged ESCs without coculture with WJ-MSCs were used as the non-cocultured group.

### Morphological changes of ESCs

The Transwell chambers in the culture plates were removed, leaving the lower layer of ESCs. The morphological changes of ESCs in each group were then observed by optical microscopy (Olympus, Japan), and the morphological effects of WJ-MSCs on the repair of damaged ESCs were investigated.

### CCK-8 assay as an indicator of ESC proliferation

The proliferation of ESCs was evaluated using a Cell Counting Kit (CCK-8; Beyotime, Jiangsu, China) according to the manufacturer’s instructions. After cocultured with WJ-MSCs for 24 h, 48 h, and 72 h, the proliferative ability of damaged ESCs in the cocultured or non-cocultured groups was determined. At the end of each experiment, 10 μl/well of CCK-8 assay solution was added to each culture, and samples were incubated for 2 h. Absorbance was recorded at 450 nm on an automatic microplate reader (Bio Teck, CA, USA) within 30 min. Cells in each group were prepared in triplicate.

### Detection of apoptosis in ESCs by flow cytometry

To detect apoptosis, the ESCs from each group were collected and washed twice with cold PBS and then resuspended in 1× binding buffer at a concentration of 1 × 10^6^/ml. A 100 μl volume of this solution was then transferred to a 5-ml culture tube, and 5 μl fluorescein isothiocyanate-Annexin V and 5 μl propidium iodide (Becton Dickinson, San Jose, CA, USA) were added. After incubation for 15 min at 25 °C in the dark, 400 μl of 1× binding buffer was added to each tube. Stained cells were then sorted with a FACScan flow cytometer (Becton Dickinson, NJ, USA) within 1 h. Cells in each group were prepared in triplicate.

### Circular RNA microarray analysis

A CapitalBio Technology Human CircRNA Array v2 was designed with four identical arrays per slide (4 × 180 K format), with each array containing probes interrogating approximately 170,340 human circRNAs. The circRNA array also contained 4974 Agilent control probes. In total, 7755 circRNAs were detected. All microarray analysis was performed by CapitalBio Technology (Beijing, China). The circRNA array data were analyzed for data summarization, normalization, and quality control with GeneSpring software V13.0 (Agilent). To select differentially expressed genes, we used threshold values of ≥ 2 and ≤ − 2-fold change, and a *t* test *P* value of 0.05. Data were log_2_ transformed and median-centered by genes in CLUSTER 3.0 software, then further analyzed by hierarchical clustering with average linkage. Finally, tree visualization was performed in Java Treeview (Stanford University School of Medicine, Stanford, CA, USA).

### Extraction of total RNA and quantitative polymerase chain reaction (qPCR)

Total RNA was extracted from all groups by using Trizol reagent (Invitrogen, Carlsbad, CA, USA) and purified with a mirVana miRNA Isolation Kit (Ambion, Austen, TX, USA) according to the manufacturer’s protocol. The purity and concentration of RNA were then determined from OD260/280 readings with a NanoDrop 2000 spectrophotometer (Thermo Scientific). RNA integrity was then determined by 1% formaldehyde denaturing gel electrophoresis. According to the manufacturer’s instructions, we used a PrimeScript RT reagent kit (Takara Bio, Japan) to perform reverse transcription with the quantified RNA to synthesize complementary DNA (cDNA). QPCR was performed using SYBR-Green Premix Ex Taq (Takara Bio, Japan) and evaluated using an ABI PRISM 7500 Sequence Detection System. GAPDH was used to normalize RNA preparations. The relative expression levels of selected circRNAs and host gene mRNA were determined using the 2^−△△CT^ method. Primer sequences for circRNAs and host genes for qPCR are listed in Table 1 and Table 2, respectively.

**Table 1:** The characteristics and primers of the nine circRNAs

Serial number	circRNAs	gene	Sequence (5′- > 3′) (Forward/Reverse)	Template strand	Product length
1	hsa-circRNA8881-21	ASPM	TGTCTCTTCTGTAAAGATGCCGAA	Plus	92
			ACCAATTCGAAGCCACAAAGGA	Minus	
2	hsa_circ_0020488	MKI67	CAGTGACCAGCCACAGGAGA	Plus	188
			CGACCCCGCTCCTTTTGATA	Minus	
3	hsa_circ_0020492	MKI67	AATCCATGAGCAGGAGGCAAT	Plus	166
			GGGGAAGGCCAGAAGCAAA	Minus	
4	hsa_circ_0026141	TROAP	TAACCGCCATCCACTGCTTC	Plus	178
			GGGCGAGTGGAAGGGTGAAA	Minus	
5	hsa-circRNA4049-38	WDR62	GTTCCTCCGCCACCACTTTGA	Plus	195
			TCATGGGGGTAAAGTAGCAATCCA	Minus	
6	hsa_circ_0015825	KIF14	AGGGGTGAAGATGCCTTTTGTG	Plus	164
			GACCCTAAGCTCTTCTTTGGACAT	Minus	
7	hsa-circRNA5028-15	MYBL2	GAAAGTCCGGAAGTCTCTGGC	Plus	171
			GTGGTTGTGCCAGCGTTCA	Minus	
8	hsa_circ_0020487	MKI67	CTCTATCCCAGTGACCAGCCA	Plus	145
			ATTGCCTCCTGCTCATGGATTT	Minus	
9	hsa_circ_0111659	KIF14	GGCTGAGTGATTTACTGCCTTGT	Plus	174
			GGTCTTTTCCTGCAGAGGTGTT	Minus	
10		GAPDH	GGACTCATGGTATGAGAGCTGG	Plus	180
			CAGCGTACTCCCCACATCAC	Minus

**Table 2:** The primers of the host genes

Serial number	gene	Sequence (5′- > 3′) (Forward/Reverse)	Template strand	Length
1	ASPM	GAGCATTTCTGTCTGCAAAACATC	Plus	24
		TTGCAGGCAGCTTTCACTTTAC	Minus	22
2	MKI67	TTGTAAATTTGCTTCTGGCCTTC	Plus	23
		ACGGATGTCACATTCAATACCC	Minus	22
3	TROAP	GGATCAGTCAGCCTCGGAAC	Plus	20
		CAGGGTCAGCCACAAACTCT	Minus	20
4	WDR62	TGAGAAGAGGGTGCTGGAGA	Plus	20
		CCTGTGCCACGTCTACCC	Minus	18
5	KIF14	GACTTCAGGGCCTCTCGG	Plus	18
		GACCCTAAGCTCTTCTTTGGAC	Minus	22
6	MYBL2	GAGGGATAGCAAGTGCAAGGT	Plus	21
		TTCCAGTCCTGCTGTCCAAA	Minus	20

### Bioinformatics analysis

CircRNA can target miRNA and indirectly regulate mRNA translation. To further elucidate the role of circRNAs in the repair of endometrial damage, we first selected differentially expressed circRNAs and then predicted the target miRNAs for each selected circRNA in miRanda software. According to the relationship between circRNAs and target miRNAs, the first ten circRNAs with the largest fold change (FC) were selected to create a circRNA-miRNA network diagram. circRNA function was predicted by Gene Ontology (GO) functional annotation of co-expressed genes. Gene functions were classified into three subgroups: biological process, cellular component, and molecular function. GO terms with a *P* value less than 0.05 were considered statistically significant. The top 30 enriched GO terms among the two groups, ranked by fold enrichment and enrichment score, are presented herein. KEGG pathway analysis was then performed to determine the involvement of co-expressed genes in different biological pathways.

Because we previously found that the expression of VEGF is upregulated and involved in the WJ-MSC-mediated repair of damaged ESCs [13], the prediction of circRNA-miRNA-VEGF gene associations was also determined by using two databases: Targetscan (http://www.targetscan.org/vert_72/) and miRwalk (http://zmf.umm. uni-heidelberg.de/apps/zmf/mirwalk2/index.html).

### Statistical analysis

Data were analyzed in SPSS20.0 software. All quantitative data are expressed as mean ± standard deviation (SD). Student’s *t* test was used to compare data between the cocultured and non-cocultured groups. One-way analysis of variance was used to analyze the circRNAs levels of ESCs or WJ-MSCs after coculture for 24 h, 48 h, and 72 h. *P* < 0.05 was considered to indicate a statistically significant difference.

## Results

### WJ-MSCs exert a reparative effect upon damaged ESCs

**Fig. 1:**
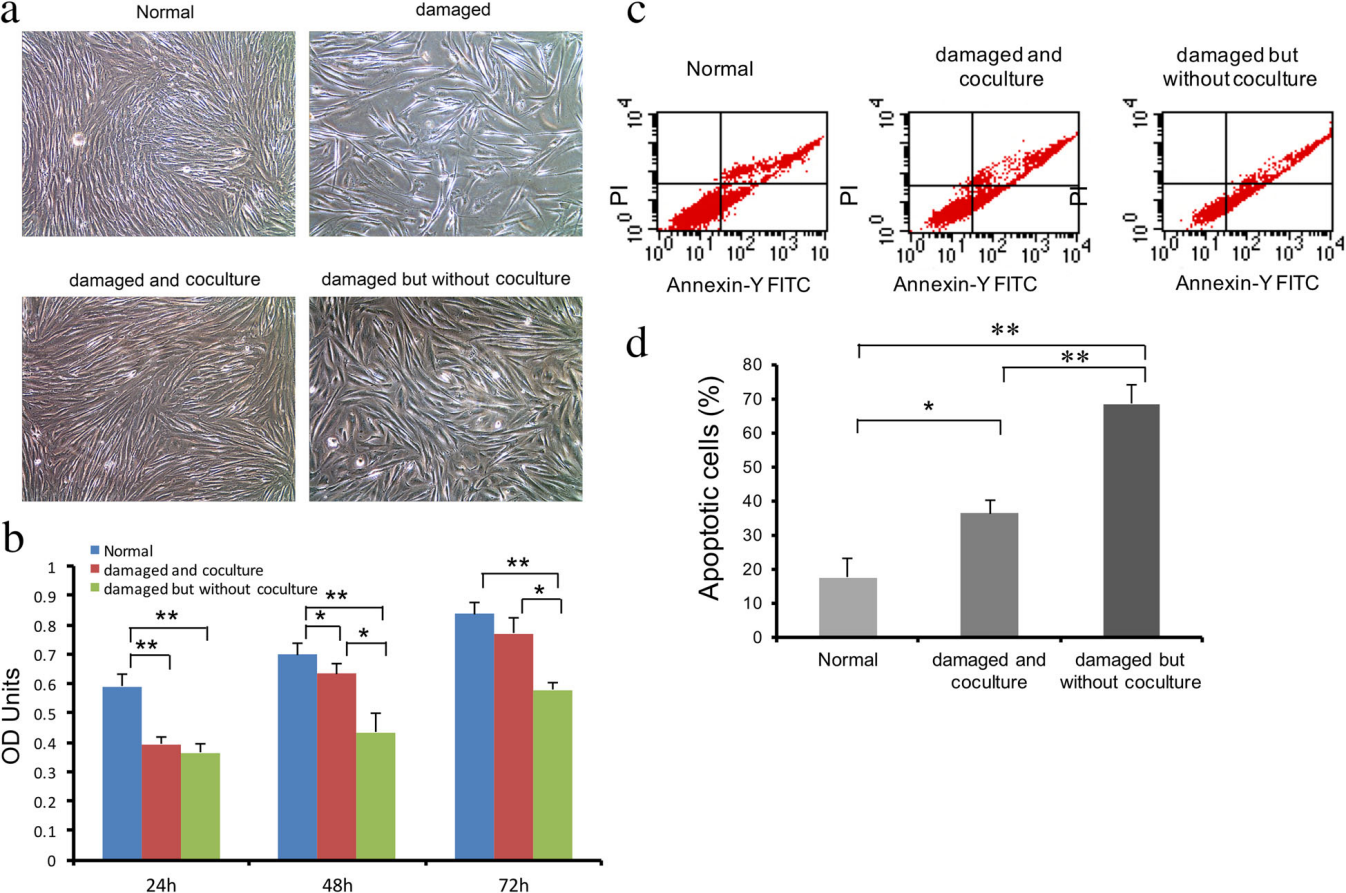
WJ-MSCs had a reparative repair effect on damaged ESCs. **a** Morphological changes of ESCs after treatment with mifepristone (60 μmol/L) for 48 h. **b** Absorbance of ESCs in different groups at 24 h, 48 h, and 72 h, as shown by CCK-8 assays. **c**, **d** The proportions of apoptotic cells in the three groups were evaluated by Annexin V/propidium iodide staining and flow cytometry. The proportion of apoptotic cells was higher in the non-cocultured group than the cocultured group and normal group. Data are expressed as mean ± standard deviation (SD). Scale bars = 10 μm.*P < 0.05; **P < 0.01

After the treatment of ESCs with mifepristone for 48 h, optical microscopy revealed disordered distribution, larger cell intervals, vacuolization phenomena, and poor shading of damaged cells compared with normal ESCs. After the damaged ESCs were cocultured with WJ-MSCs for 48 h, the growth state of cells was similar to that of normal cells, and the number of cells was significantly higher, the arrangement of cells was more regular and dense, although the density was still lower than that in the normal group. However, although the cell morphology of ESCs in the non-cocultured group recovered to a certain extent, and the number of nuclear vacuoles was lower than that initially seen in the damaged cells, the cells were still rare and disordered, as compared with cells in the normal group (Fig. 1a).

After coculture for 48 h, the proliferative ability of cells in the cocultured group was higher than that in the non-cocultured group, and this difference continued up to 72 h (*P* < 0.05). The proliferative ability of damaged ESCs in the non-cocultured group was significantly lower than that in the normal group after 24 h, 48 h, and 72 h (*P* < 0.01; Fig. 1b).

The apoptosis rate of the cocultured group (36.29 ± 3.70%) was significantly lower than that of the non-cocultured group (68.58 ± 5.47%, *P* < 0.01) but was still higher than that of ESCs in the normal group (17.72 ± 7.42%, *P* < 0.05). However, the apoptosis rate of ESCs in the non-cocultured was significantly higher than that of the normal group (*P* < 0.01; Fig. 1c, d).

### Analysis of circRNA expression profiles

**Fig. 2:**
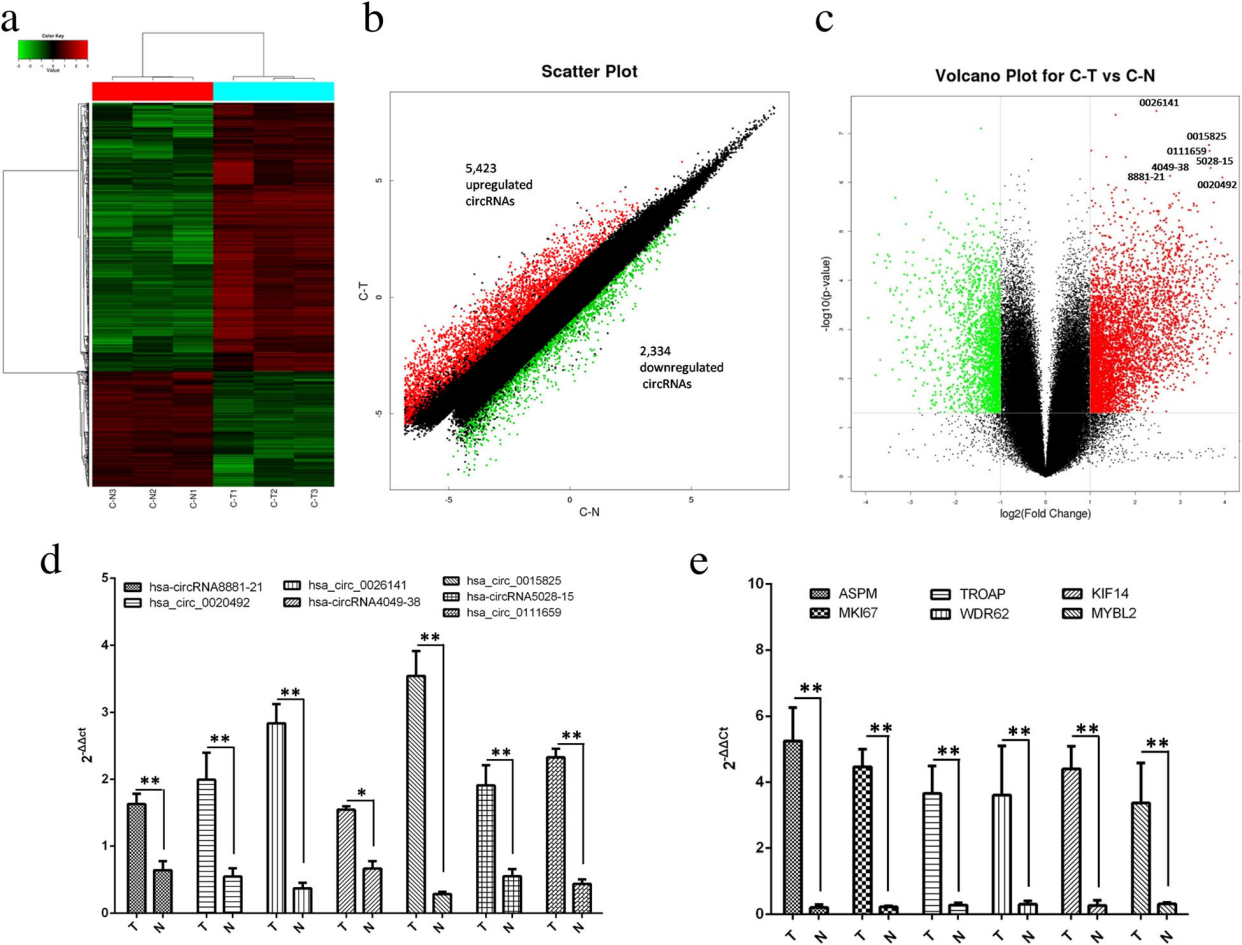
CircRNA expression profile in the cocultured group (Group T) relative to the non-cocultured group (Group N). **a** Hierarchical clustering of circRNAs. Each group consisted of three individuals. circRNAs are represented by single rows and samples by single columns. **b** Scatter plot of circRNAs. The values corresponding to the *X*- and *Y*-axes represent normalized signal values. **c** Volcano plot of circRNAs. The values on the *X*- and *Y*-axes represent normalized fold changes and *P* values, respectively. The color scale indicates relative expression, upregulation (red) and downregulation (green). CircRNAs with fold change > 2 and *P* < 0.05 were regarded as differentially expressed. CircRNAs: circular RNAs. **d** CircRNA levels in damaged ESCs cocultured with or without WJ-MSCs for 48 h. **e** Host gene levels of the selected circRNAs. T: cocultured group; N: non-cocultured group

CircRNA expression profiles are presented in the form of a heat map (Fig. 2a). The differences in circRNA expression profiles between the two groups are further shown as a scatter plot (Fig. 2b) and volcano plot (Fig. 2c). Overall, 7757 circRNAs were significantly differentially expressed (fold change > 2.0 or < − 2.0, *P* < 0.05). In comparison with samples from the non-cocultured group, 5423 circRNAs were upregulated (Additional file 1: Table S1) and 2334 circRNAs (Additional file 1: Table S2) were downregulated in samples from the cocultured group (Fig. 2b).

Subsequently, we narrowed the scope of our analysis to the nine most aberrantly expressed circRNAs. Table 1 shows the nine circRNAs that showed the largest FC in our microarray. We found that all nine of these circRNAs were upregulated, according to our microarray analysis. We further confirmed the expression levels of these nine circRNAs by qPCR using the same cohort of samples in RNA microarray analysis. Seven of the nine circRNAs were confirmed to be significantly upregulated in the cocultured group (*P* < 0.05), including hsa-circRNA8881-21, hsa_circ_0020492, hsa_circ_ 0026141, hsa-circRNA4049-38, hsa_circ_0015825, hsa-circRNA5028-15 and hsa_circ_ 0111659 (Fig. 2c, d), whose expression levels were consistent with the RNA microarray analysis. However, the expression levels of hsa_circ_0020487 and hsa_circ_0020488 were too low to be detected. Meanwhile, we also evaluated host genes of the seven validated circRNAs (Table 2), including abnormal spindle-like microcephaly (ASPM), marker of proliferation Ki-67, (MKI67), trophinin-associated protein (TROAP, also named TASTIN), WD repeat domain 62 (WDR62), kinesin family protein 14 (KIF14), and V-Myb avian myeloblastosis viral oncogene homolog-like 2 (MYBL2) using qPCR. Compared with those in the non-cocultured samples, the expression levels of the host genes in the cocultured group were significantly higher, although the expression level of host genes was not completely consistent with that of their respective circRNAs (Fig. 2e).

**Fig. 3:**
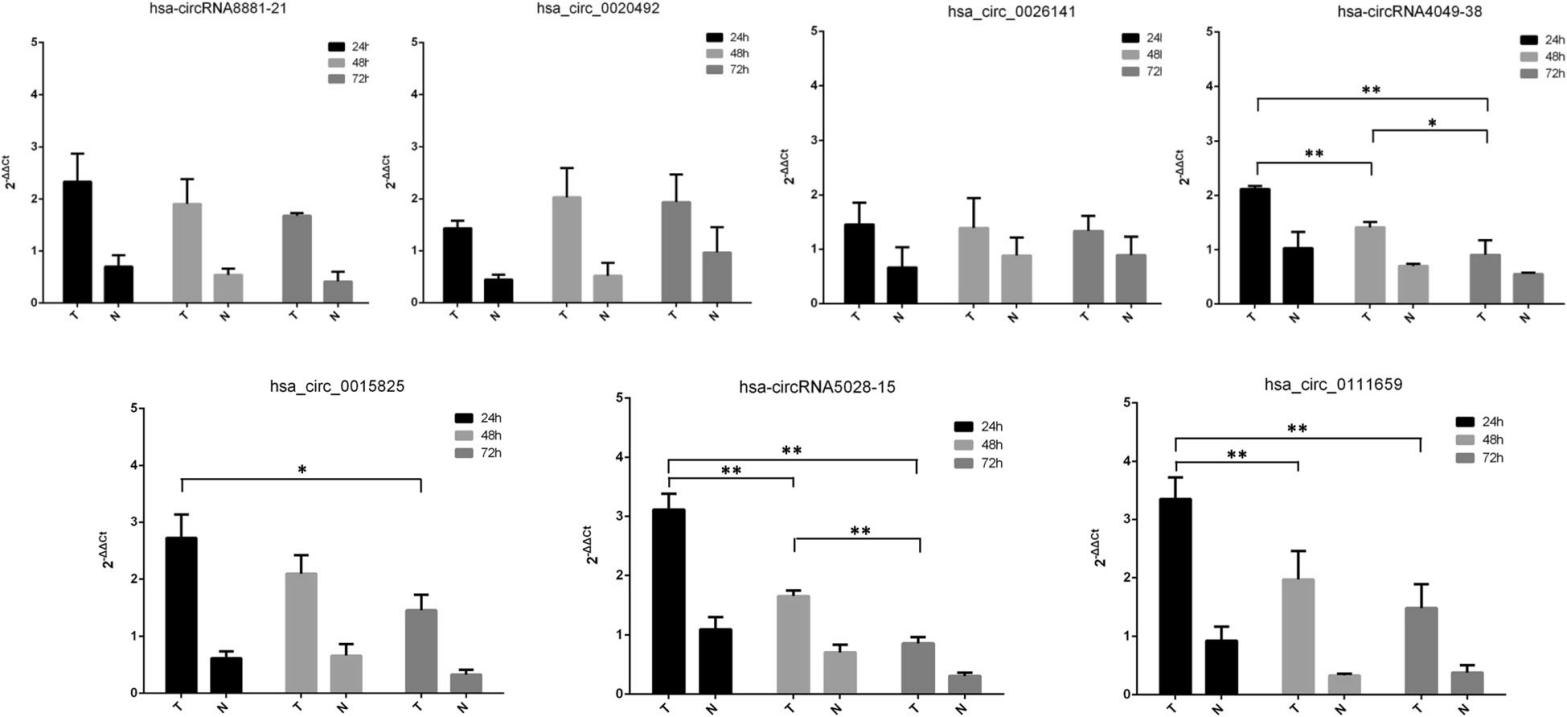
The expression of selected circRNAs (hsa-circRNA8881-21, hsa_circ_ 0020492, hsa_circ_0026141, hsa-circRNA4049-38, hsa_circ_0015825, hsa-circRNA5028-15, and hsa_ circ_0111659) in damaged ESCs cocultured with or without WJ-MSCs at 24 h, 48 h, and 72 h, according to qPCR. Data are expressed as mean ± standard deviation (SD). *P < 0.05; **P < 0.01; T: cocultured group; N: non-cocultured group

**Fig. 4:**
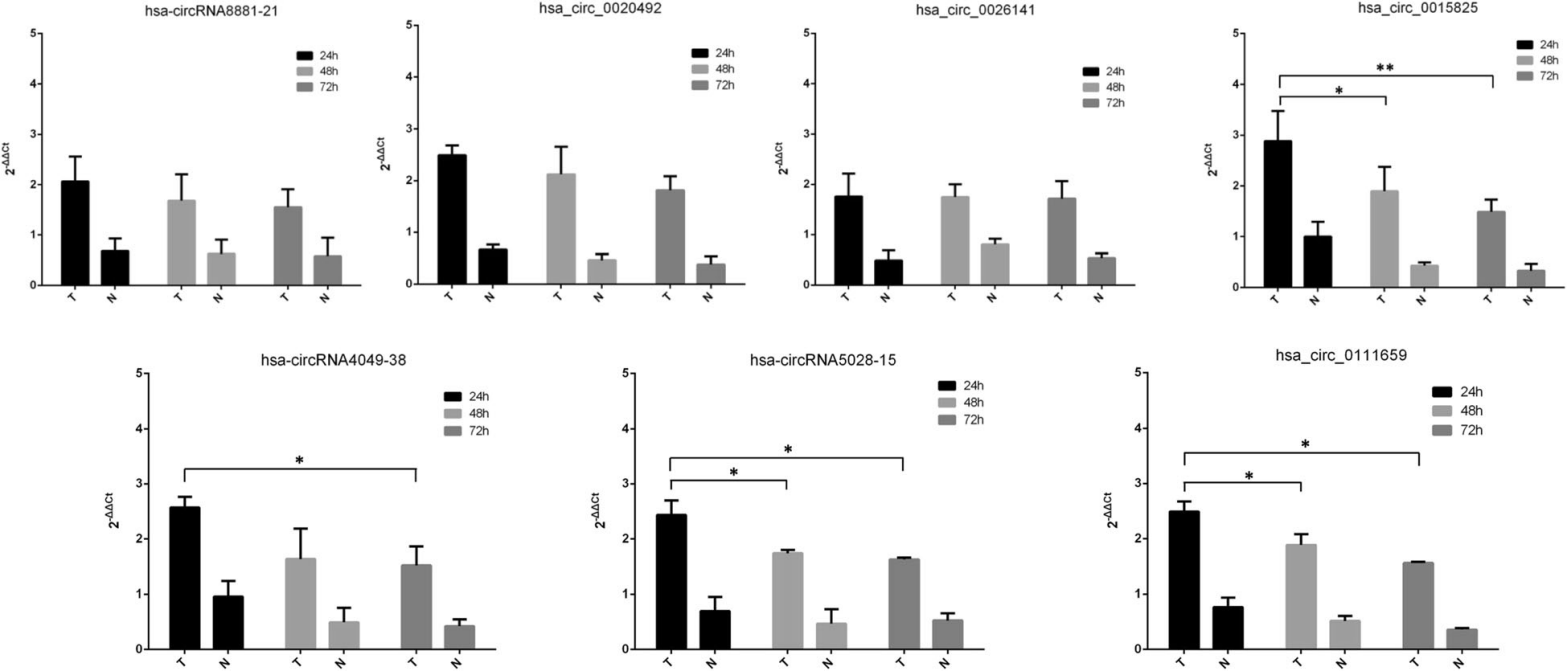
The expression of selected circRNAs (hsa-circRNA8881-21, hsa_circ_0020492, hsa_circ_0026141, hsa-circRNA4049-38, hsa_circ_0015825, hsa-circRNA5028–15, and hsa_ circ_0111659) in WJ-MSCs cocultured with or without damaged ESCs at 24 h, 48 h, and 72 h, according to qPCR. Data are expressed as mean ± standard deviation (SD). *P < 0.05; **P < 0.01; *T*: cocultured group; *N:* non-cocultured group

Furthermore, we observed trends in the dynamic expression trend of seven circRNAs in both ESCs and WJ-MSCs of the cocultured group during the repair process. The expression levels of the seven circRNAs at 24 h, 48 h, and 72 h were compared using qPCR. Trend comparison showed that the expression of the four circRNAs continued to decrease over time while the expression of four circRNAs (hsa-circRNA4049-38, hsa_circ_0015825, hsa-circRNA5028-15, and hsa_circ_0111659) in ESCs at 24 h were significantly higher than at 48 h or 72 h (*P* < 0.01 or *P* < 0.05). However, the levels of the remaining three circRNAs (hsa-circRNA8881-21, hsa_circ_0020492, and hsa_circ_0026141) did not change significantly over time (Fig. 3). Meanwhile, we evaluated the expression profiles of seven circRNAs in WJ-MSCs from the cocultured group. The expression profiles of the seven circRNAs in WJ-MSCs were similar to that seen in ESCs (Fig. 4).

### Enrichment analysis of the biological function of circRNA co-expressed genes

**Fig. 5:**
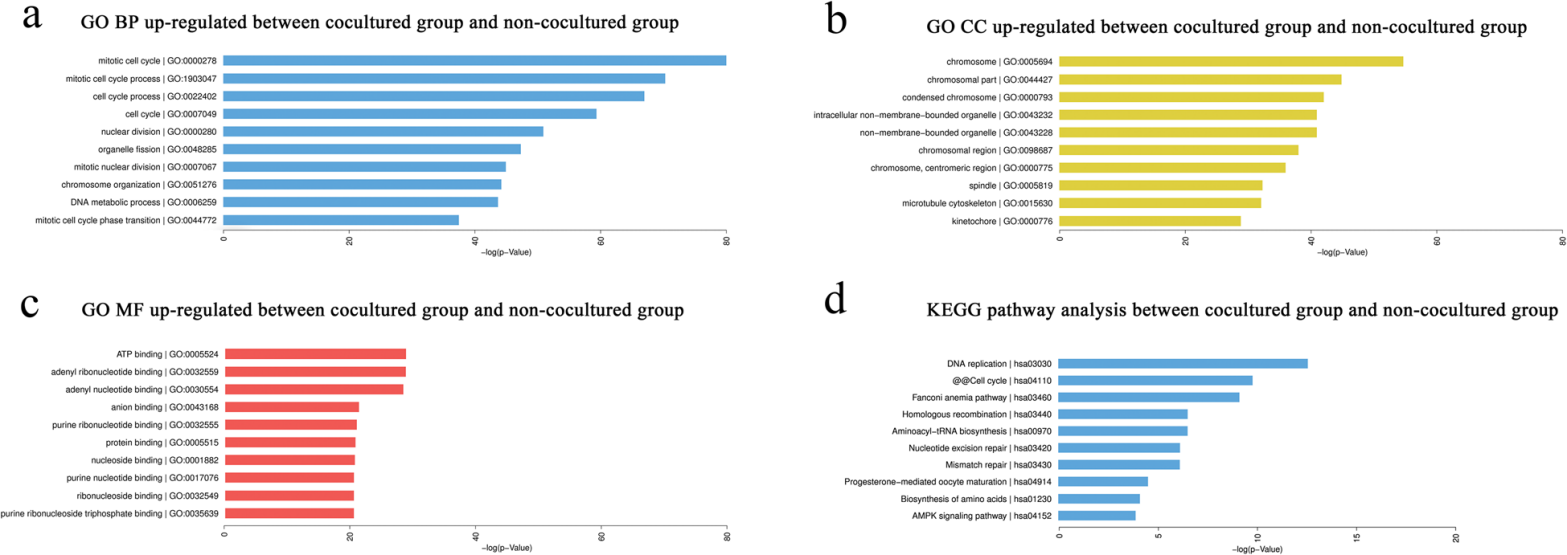
GO and KEGG analysis for circRNA-targeted mRNAs. GO enrichment for the target mRNAs, including biological processes (**a**), cell component (**b**), and molecular function (**c**). **d** KEGG pathway analysis. GO: Gene Ontology; KEGG: Kyoto Encyclopedia of Genes and Genomes; circRNAs: circular RNAs

The GO analysis results between cocultured group and non-cocultured group showed that the upregulated circRNAs were significantly enriched in the BP, including mitotic cell cycle, cell cycle process, and nuclear division (Additional file 1: Table S3). The CC analysis showed that the upregulated circRNAs were significantly enriched in the chromosome, intracellular non-membrane-bounded and nuclear replication fork (Additional file 1: Table S4). In addition, in the MF analysis, the upregulated circRNAs were enriched in ATP binding and DNA-dependent ATPase activity (Additional file 1: Table S5). We generated a list of the circRNAs with the top 10 *P* values according to the GOTERM_BP, CC, MF_FAT list for biological processes, cellular component, and molecular function of general interest. After comparing the BP, CC, and MF, it was found that the enrichment of circRNAs related to the repair in the cocultured group was upregulated. The upregulated circRNA enrichment in cocultured group occurred in the mitotic cell cycle, cell cycle process, nuclear division, chromosome, ATP binding, and adenyl ribonucleotide binding (Fig. 5a–c). The most significantly enriched pathways of the upregulated circRNAs in the KEGG analysis conducted on DAVID are shown in Fig. 5d. Between cocultured group and non-cocultured group, the upregulated circRNAs were enriched in DNA replication, cell cycle, Fanconi anemia pathway, homologous recombination, aminoacyl-tRNA biosynthesis, and nucleotide excision repair. Specific information on the top ten pathways in the KEGG pathway analysis for upregulated circRNAs is listed in Additional file 1: Table S6.

### Prediction of circRNA-miRNA-mRNA

**Fig. 6:**
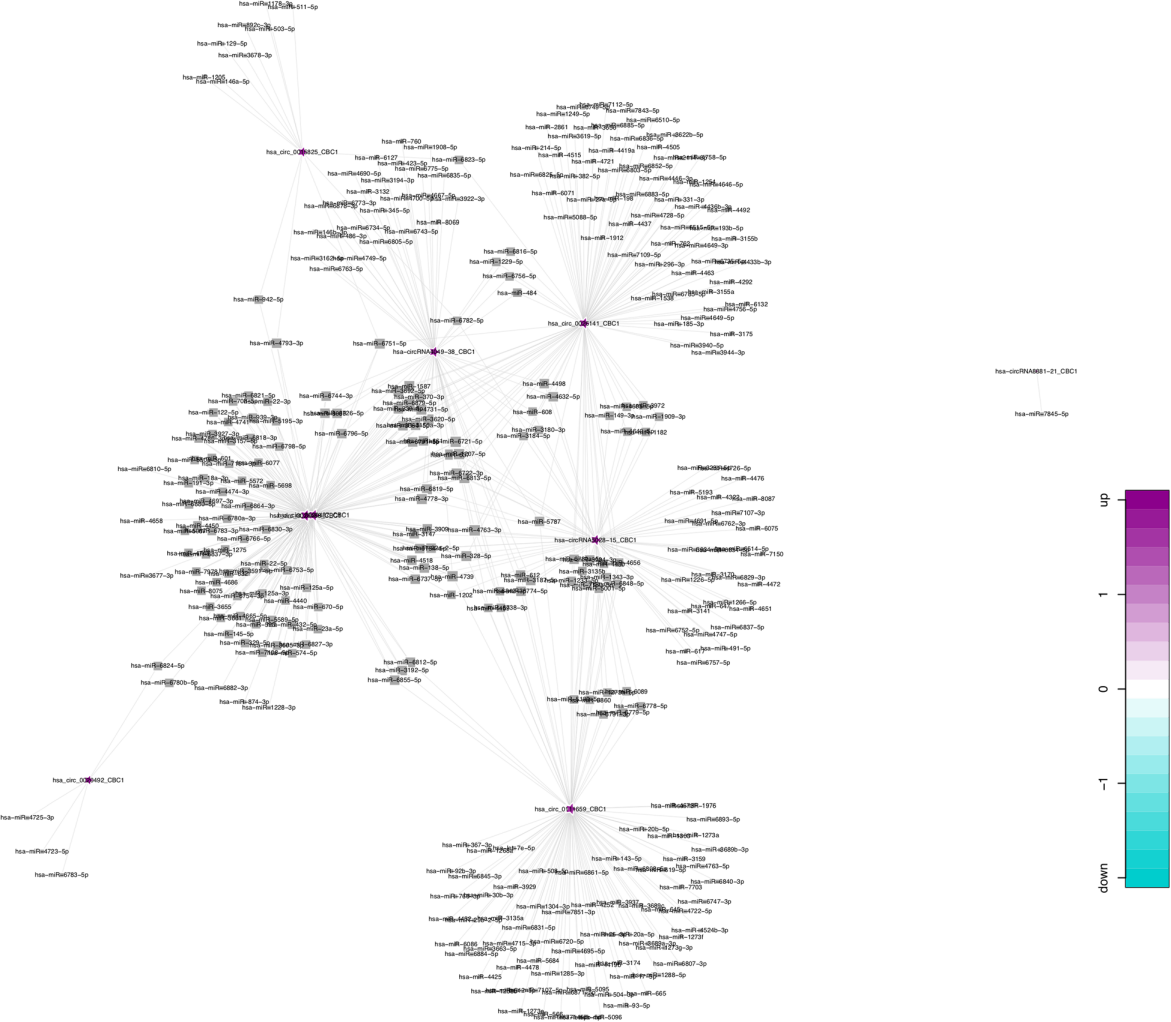
A circRNA-miRNA network diagram**.** The pentagram nodes represent circRNA, while the square nodes represent target miRNAs. Purple color and green color represents up- and downregulation, respectively. CircRNAs: circular RNAs. miRNA: microRNA

**Fig. 7:**
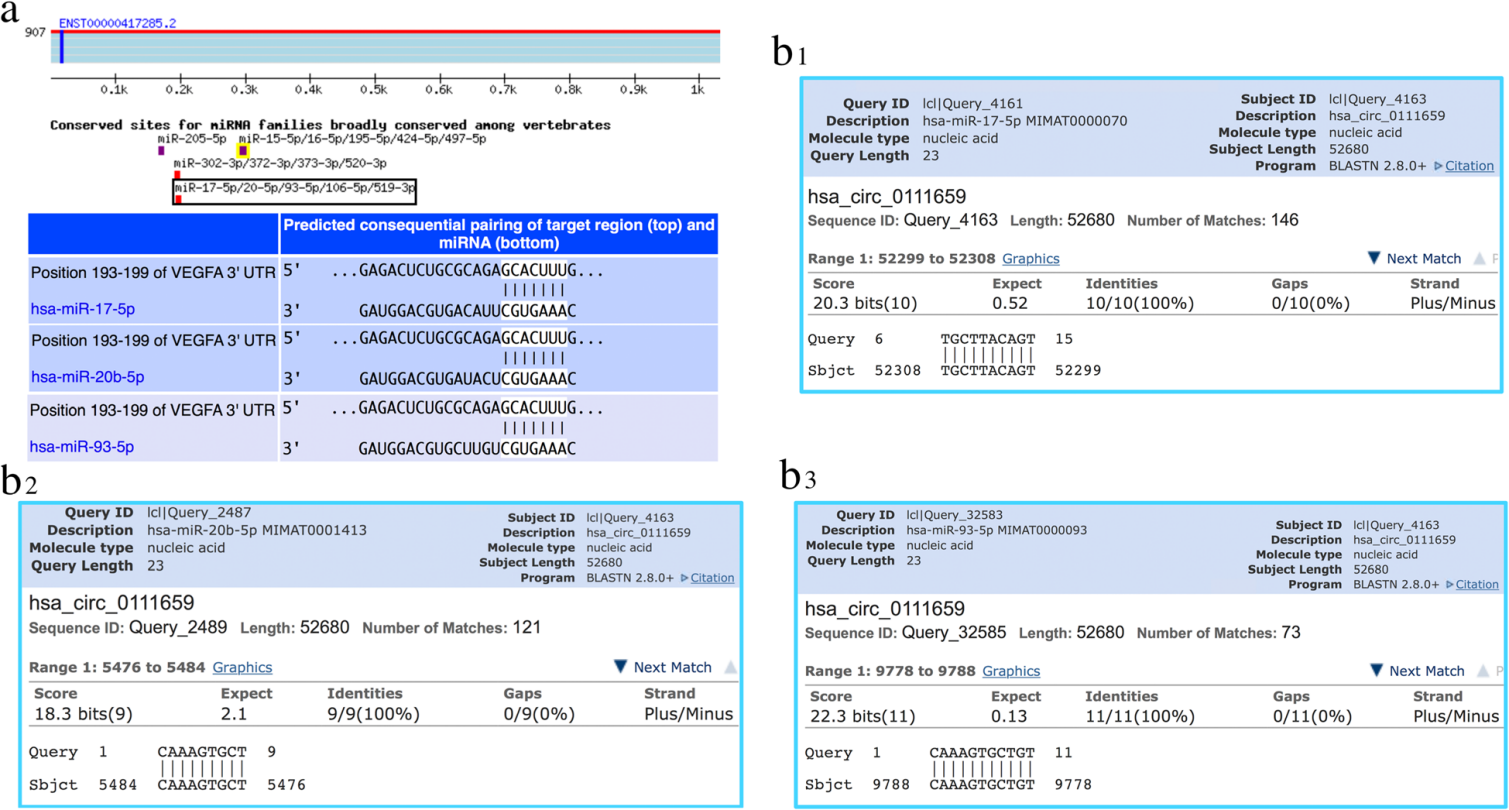
Prediction of circRNA-miRNAs-VEGF. (**a**) Molecular interactions between the VEGF gene and its target miRNAs. (**b1**–**b3**) Molecular interactions between hsa_circRNA_0111659 and its dominant target miRNAs. The common and dominant target miRNAs of hsa_circRNA_0111659 were miR-17-5p, miR-20b-5p, and miR-93-5p. CircRNAs: circular RNAs

CircRNA can target miRNA and indirectly regulate the translation of mRNA. MiRanda software was used to select the circRNAs showing differential expression and thus predict the target miRNAs; a list of the miRNAs generated is given in Additional file 1: Table S7. According to the relationship between circRNAs and target miRNAs, the first ten circRNAs with the largest FC were selected to create a circRNA-miRNA network map. Only one of the ten circRNAs did not predict a target miRNA. The remaining nine circRNAs were predicted to 545 target miRNAs. A network map was constructed containing 9 circRNAs and 545 miRNAs (Fig. 6). To investigate whether VEGF was regulated by ncRNAs, we also predicted the target miRNAs of VEGF by using the miRwalk database and identified 102 targeted VEGF miRNAs (Additional file 1: Table S8). Of these, miR-17-5p, miR-20b-5p, and miR-93-5p were predicted to be common targets of hsa_circRNA_0111659. The co-expression network showed that miR-17-5p, miR-20b-5p, and miR-93-5p were co-related with hsa_circRNA_0111659 (Fig. 7a). Using circBase to further predict miRNAs associated with circRNA_0111659 binding, we found that miR-17-5p, miR-20b-5p, and miR-93-5p have multiple binding sites with has_circRNA_0111659 (Fig. 7b1–b3). Further details of the molecular interactions between VEGF, hsa_circRNA_0111659 and miR-17-5p, miR-20b-5p, and miR-93-5p are depicted in Fig. 7.

## Discussion

WJ-MSCs are believed to facilitate the repair of damaged tissue and organs by differentiating into specific cells or/and by secreting a large number of bioactive factors such as VEGF, hepatocyte growth factor, hepatocyte growth factor, and interstitial cell-derived factor-1 (stromal cell). Previous studies and the current study clearly demonstrate that WJ-MSCs exert a reparative effect on damaged ESCs [13, 14]. In the present study, we first found that circRNAs are abundantly expressed and upregulated during the repair of damaged endometrium by WJ-MSCs; this may play an important role in improving the effect of WJ-MSCs on the repair of endometrial damage.

Recent studies have also shown that circRNAs are involved in the process of tissue damage repair. Li and colleagues [22] have used high-throughput sequencing technology to identify six key circRNAs showing significant changes during the repair of liver injury. In another study, Lin et al. [23] have also reported the upregulation of the expression three circRNAs and the downregulation of 12 circRNAs in the HT22 cell model of cerebral ischemia-reperfusion injury. Collectively, these results suggest that circRNAs are widely involved in the pathological process of damage repair and exert regulatory function at the transcriptional or post transcriptional level. circRNA expression profiles were found to be tissue-specific, thus enabling prediction of potential biological functions. However, whether circRNAs play a role in the repair of endometrial injury remains unclear.

In the present study, we found that circRNAs are abundantly expressed and upregulated during the repair of ESCs by WJ-MSCs. More than 140,000 circRNAs were detected in our study. Of these, approximately 5.5% (~ 7757 circRNAs) showed dysregulated expression in the damaged ESCs that were cocultured with WJ-MSCs for 48 h. Specifically, 5423 circRNAs were upregulated, and 2334 circRNAs were downregulated. We then focused on nine circRNAs in the cocultured group showing the largest FC in expression. All were shown to be upregulated by microarray, and seven of these nine circRNAs were significantly upregulated in microarray analysis and other cohort samples, including ESCs and WJ-MSCs. These results were not surprising, because circRNAs are known to be upregulated during cell cycle regulation and DNA damage repair [24, 25]. However, we found that the expression of four circRNAs gradually decreased in both ESCs and WJ-MSCs over time in vitro experiment. These observations can be explained, in part, by circRNAs being passively diluted during cell proliferation. Interestingly, we also found that the levels of seven circRNAs in WJ-MSCs were lower than those in ESCs. This may be because ESCs can also secrete a small proportion of circRNAs, in addition to the circRNAs secreted by WJ-MSCs.

The function of circRNAs, which are transformed from their linear host RNAs, may be related to the known functions of their host linear transcripts. In our study, both the most significant biological processes, such as cell metabolic process, developmental process and biological regulation determined by GO analysis, and the most significant pathways such as DNA replication, cell cycle, homologous recombination, nucleotide excision repair, and AMPK signaling pathway determined by KEGG pathway analysis, were related to cell proliferation or DNA repair. Moreover, the host genes of the seven validated circRNAs were ASPM, MKI67, TROAP, WDR62, KIF14, and MYBL2. All of these genes are closely related to cell proliferation, the cell cycle, cell survival, and cell differentiation [27–33]. For example, one of the most upregulated circRNAs (hsa_circRNA_0111659) is derived from the KIF14 gene encoding a member of the kinesin family of proteins, which is involved in cell division, microtubule polymer dynamics, intracellular transportation, and signal transduction [32]. The developmental gene MKI67 produced three different circRNAs and encoded a protein that binds the perichromosomal layer during mitosis [28]. Higher levels of MKI67 expression in tumor tissue have been associated with a higher tumor grade and an earlier recurrence of disease [29]. We also found that the expression of seven circRNAs was not completely consistent with their host genes. A previous report has stated that most circular RNAs are rarely generated and accumulate in low levels [34]; however, some are expressed at levels tenfold higher than their associated linear mRNA [35]. Collectively, these results suggested that the main function of some protein-coding genes may be to generate circular RNAs rather than linear mRNAs or proteins. This conclusion would be logical because circRNAs, once produced, are naturally resistant to degradation by exonucleases and thus have long half-lives; moreover, some accumulate to high levels [36]. These upregulated circRNAs are derived from cell developmental genes involved in cell proliferation and growth factor signaling and may have other functions unrelated to their host genes such as binding specific miRNAs and regulating the expression of mRNA, or binding to other proteins and regulating their activities or functions [37–39]. Furthermore, in some cases, circRNAs can regulate the expression of their own host genes [40, 41]. However, it remains unclear whether this is a general mechanism among circRNAs.

From the circRNA-miRNA co-expression network, we found most of the circRNAs in the co-expression network had not been annotated as yet. It would therefore be very useful to perform further studies to reveal the underlying mechanisms of these circRNAs. CircRNAs might act as competing endogenous RNAs, thus regulating the function of miRNAs [19]. In our study, we predicted the differentially expressed circRNAs and found hsa_circRNA_0111659 possesses miR-17-5p, miR-20b-5p, and miR-93-5p binding sites, thus suggesting that hsa_circRNA_0111659 may be involved in the functional regulation of these three miRNAs in the process of repair by WJ-MSCs. Previous studies using PCR have also shown that the expression of miR-17-5p, miR-20b-5p, and miR-93-5p is downregulated in various types of cancer, thus suggesting that these act as tumor suppressors [42–50]. MiR-17-5p, one of the angiogenesis-associated miRNAs in human placentas, has been reported to be downregulated in endometriotic tissue [51, 52]. These studies collectively indicate that the downregulated expression of miR-17-5p, miR-20b-5p, and miR-93-5p is associated with cell proliferation and tissue repair. The reduction in the expression of these miRNAs eases the post-transcriptional suppression of its target mRNA encoding the growth factor VEGF, thus promoting angiogenesis during the repair of endometrial lesions [53]. It has been suggested that the differentially expressed circRNAs, hsa_circRNA_0111659, act as miRNA sponges and may competitively bind miR-17-5p, miR-20b-5p, and miR-93-5p and release the target VEGF mRNA. However, the targeted verification of hsa_circRNA_0111659/miR-17-5p, miR-20b-5p, and miR-93-5p/VEGF, as well as the function and molecular mechanism of hsa_circRNA_0111659 in the repair of endometrium by WJ-MSC, remains unclear and requires further investigation.

## Conclusions

The present study is the first to identify the expression profile of circRNAs in the WJ-MSC-mediated repair of endometrial damage. We described circRNA expression patterns during the repair of endometrium damage by WJ-MSCs and found that more than 7000 circRNAs are differentially expressed in this process. Many circRNAs may participate in the biological pathways related to the repair of endometrial damage by different regulatory mechanisms. Moreover, we focused on hsa_circRNA_ 0111659 and predicted its miRNAs and targeted mRNA. The association of circRNA-miRNA-mRNA is likely to be involved in regulating the repair of endometrial damage. Our findings present a clearer understanding of the expression profile during WJ-MSC-mediated repair of the endometrium and provide a novel perspective for using WJ-MSCs effectively in the treatment of endometrial damage.

## Additional files

Additional file 1: **Table S1.** Upregulated circRNAs in damaged ESCs from the cocultured group. **Table S2.** Downregulated circRNAs in damaged ESCs from the cocultured group. **Table S3.** GO BP analysis between cocultured group and non-cocultured group. **Table S4.** GO CC analysis between cocultured group and non-cocultured group. **Table S5.** GO MF analysis between cocultured group and non-cocultured group. **Table S6.** Top 10 KEGG pathway analysis between cocultured group and non-cocultured group. **Table S7.** Top 10 circRNA_ miRNAs. **Table S8. **Predicted miRNAs of VEGF by miRwalk database. (DOCX 1130 kb)

## Abbreviations

BP: Biological process
CC: Cellular component
CircRNA: Circular RNA
ESC: Endometrial stromal cell
FBS: Fetal bovine serum
GO: Gene Ontology
KEGG: Kyoto Encyclopedia of Genes and Genomes
MF: Molecular function
miRNA: MicroRNA
NcRNA: Non-coding RNA
QPCR: Quantitative polymerase chain reaction
VEGF: Vascular endothelial growth factor
WJ-MSC: Wharton’s jelly-derived mesenchymal stem cell
