# Supplementary material for: Circular RNAs are abundantly expressed and upregulated during repair of the damaged endometrium by Wharton’s jelly-derived mesenchymal stem cells
Source: Stem Cell Res Ther. 2018 Nov 15;9:314. doi: 10.1186/s13287-018-1046-3 (PMC6238312; doi:10.1186/s13287-018-1046-3)
Supplement: Supplementary file 1 — Table S1. Upregulated circRNAs in damaged ESCs from the cocultured group. Table S2. Downregulated circRNAs in damaged ESCs from the cocultured group. Table S3. GO BP analysis between cocultured group and non-cocultured group. Table S4. GO CC analysis between cocultured group and non-cocultured group. Table S5. GO MF analysis between cocultured group and non-cocultured group. Table S6. Top 10 KEGG pathway analysis between cocultured group and non-cocultured group. Table S7. Top 10 circRNA_ miRNAs. Table S8. Predicted miRNAs of VEGF by miRwalk database. (DOCX 1130 kb) [file 13287_2018_1046_MOESM1_ESM.docx]

| **Table S1 Upregulated circRNAs between cocultured group and non-cocultured group** | | |
| --- | --- | --- |
| **ProbeName** | **P** | **FC (abs)** |
| hsa_circ_0015321_CBC1 | 0.033682812 | 2.568726791 |
| hsa_circ_0088927_CBC1 | 0.001768292 | 2.29947933 |
| hsa_circ_0018493_CBC1 | 0.001574306 | 2.093240956 |
| hsa-circRNA72-57_CBC1 | 0.024706291 | 2.493062925 |
| hsa-circRNA7754-4_CBC1 | 0.005089512 | 2.788097032 |
| hsa_circ_0098848_CBC1 | 0.020763924 | 3.29677972 |
| hsa_circ_0022679_CBC1 | 0.00250338 | 2.099542313 |
| hsa_circ_0036712_CBC1 | 0.000373627 | 4.131147464 |
| hsa_circ_0046793_CBC1 | 0.001355985 | 2.310738343 |
| hsa_circ_0111169_CBC1 | 0.030490854 | 2.457816057 |
| hsa_circ_0068016_CBC1 | 0.000968645 | 4.585367895 |
| hsa_circ_0079875_CBC1 | 9.86628E-05 | 4.722602044 |
| hsa_circ_0131542_CBC1 | 0.022715692 | 2.26065954 |
| hsa_circ_0084896_CBC1 | 0.019449013 | 2.9327559 |
| hsa_circ_0049220_CBC1 | 0.000901084 | 2.879230809 |
| hsa_circ_0060552_CBC1 | 0.000317839 | 10.70337789 |
| hsa-circRNA4326-7_CBC1 | 0.000115192 | 8.540012608 |
| hsa_circ_0001885_CBC1 | 0.018084958 | 2.2103333 |
| hsa_circ_0063048_CBC1 | 0.000160975 | 4.041685589 |
| hsa_circ_0111000_CBC1 | 0.005769792 | 3.34098146 |
| hsa-circRNA15355-21_CBC1 | 0.007970654 | 3.332497604 |
| hsa_circ_0043997_CBC1 | 0.027159179 | 2.404173966 |
| hsa-circRNA14131-21_CBC1 | 7.15195E-05 | 2.648134903 |
| hsa-circRNA9867-45_CBC1 | 0.008808269 | 2.221913071 |
| hsa-circRNA8433-7_CBC1 | 0.001155076 | 8.706133802 |
| hsa_circ_0049198_CBC1 | 0.000531541 | 3.678138487 |
| hsa_circ_0000533_CBC1 | 0.015718705 | 2.891509429 |
| hsa_circ_0014770_CBC1 | 0.047134542 | 2.012793724 |
| hsa-circRNA9154-36_CBC1 | 0.029940663 | 2.230027737 |
| hsa_circ_0036741_CBC1 | 0.000169525 | 2.443264622 |
| hsa_circ_0035672_CBC1 | 0.016917197 | 2.322997223 |
| hsa_circ_0071292_CBC1 | 7.63763E-06 | 2.436897038 |
| hsa_circ_0107503_CBC1 | 0.040497426 | 2.071180977 |
| hsa-circRNA8726-3_CBC1 | 0.000593301 | 2.362670653 |
| hsa_circ_0040484_CBC1 | 9.65862E-05 | 3.455900718 |
| hsa-circRNA2313-12_CBC1 | 0.003903064 | 3.709654874 |
| hsa_circ_0081299_CBC1 | 0.011129873 | 2.002233435 |
| hsa_circ_0119652_CBC1 | 0.000480235 | 7.093069882 |
| hsa-circRNA14654-5_CBC1 | 0.026223085 | 2.092128787 |
| hsa_circ_0088995_CBC1 | 0.000121964 | 2.302394061 |
| hsa-circRNA14643-1_CBC1 | 0.009349216 | 3.000497583 |
| hsa_circ_0043505_CBC1 | 0.027073699 | 2.229341632 |
| hsa_circ_0140381_CBC1 | 0.000937008 | 2.716021107 |
| hsa-circRNA946-16_CBC1 | 0.000211291 | 2.156115793 |
| hsa-circRNA4355-3_CBC1 | 0.003203769 | 3.339108722 |
| hsa-circRNA391-32_CBC1 | 0.005392253 | 3.556261463 |
| hsa_circ_0007255_CBC1 | 0.000294914 | 7.74873311 |
| hsa_circ_0045017_CBC1 | 0.003252055 | 2.275787822 |
| hsa_circ_0047941_CBC1 | 0.029635163 | 2.632450585 |
| hsa-circRNA13520-16_CBC1 | 0.012066924 | 3.554546995 |
| hsa_circ_0129343_CBC1 | 0.000374635 | 4.924369717 |
| hsa_circ_0059710_CBC1 | 0.000177629 | 8.080137943 |
| hsa_circ_0088964_CBC1 | 0.000553278 | 2.501965076 |
| hsa-circRNA1030-14_CBC1 | 0.00100427 | 6.660263904 |
| hsa_circ_0058319_CBC1 | 0.007346753 | 2.652165901 |
| hsa_circ_0086196_CBC1 | 7.728E-05 | 3.230856583 |
| hsa_circ_0036098_CBC1 | 0.004734127 | 2.593360017 |
| hsa_circ_0081393_CBC1 | 0.009536882 | 2.675006286 |
| hsa_circ_0029084_CBC1 | 0.000341988 | 2.410182587 |
| hsa_circ_0053015_CBC1 | 0.025319409 | 3.083197216 |
| hsa_circ_0074318_CBC1 | 0.005095708 | 2.233890709 |
| hsa-circRNA8326-6_CBC1 | 0.005124636 | 2.647145471 |
| hsa_circ_0094261_CBC1 | 0.000293486 | 2.99112714 |
| hsa-circRNA13787-3_CBC1 | 5.18151E-05 | 3.108286197 |
| hsa_circ_0103438_CBC1 | 0.001138846 | 2.916285755 |
| hsa_circ_0066933_CBC1 | 0.011976191 | 9.88145356 |
| hsa_circ_0094369_CBC1 | 0.00639222 | 4.377216884 |
| hsa_circ_0084219_CBC1 | 0.002853006 | 2.813728947 |
| hsa_circ_0079200_CBC1 | 0.001521508 | 2.798315872 |
| hsa_circ_0087038_CBC1 | 0.002576526 | 3.479699152 |
| hsa_circ_0092383_CBC1 | 0.00058977 | 2.104883993 |
| hsa-circRNA2520-18_CBC1 | 1.87451E-05 | 8.474470033 |
| hsa_circ_0036710_CBC1 | 8.70173E-05 | 5.379666407 |
| hsa-circRNA7275-24_CBC1 | 0.007057098 | 2.527713625 |
| hsa_circ_0000889_CBC1 | 0.010362696 | 2.17030023 |
| hsa-circRNA5124-1_CBC1 | 0.008235858 | 2.402020455 |
| hsa-circRNA7413-9_CBC1 | 0.001640358 | 2.832213495 |
| hsa_circ_0028451_CBC1 | 0.031783886 | 3.068345197 |
| hsa-circRNA11242-46_CBC1 | 0.00519926 | 3.601826751 |
| hsa_circ_0019227_CBC1 | 0.017318858 | 2.142974933 |
| hsa_circ_0034613_CBC1 | 0.003832983 | 4.20524438 |
| hsa_circ_0126557_CBC1 | 0.020760141 | 2.080508728 |
| hsa_circ_0126443_CBC1 | 0.004468579 | 2.643479891 |
| hsa_circ_0032089_CBC1 | 0.027233273 | 2.169393452 |
| hsa_circ_0044124_CBC1 | 0.000122484 | 6.582048146 |
| hsa_circ_0045875_CBC1 | 0.001457814 | 4.200711818 |
| hsa_circ_0098772_CBC1 | 0.000622646 | 4.498024314 |
| hsa_circ_0063039_CBC1 | 0.000230623 | 3.019009269 |
| hsa-circRNA11084-12_CBC1 | 0.000907527 | 11.40428419 |
| hsa_circ_0029503_CBC1 | 0.005955235 | 2.233392896 |
| hsa_circ_0086190_CBC1 | 0.009323266 | 2.090615073 |
| hsa_circ_0069272_CBC1 | 0.000937806 | 4.734202653 |
| hsa-circRNA15428-4_CBC1 | 0.02595817 | 2.097397552 |
| hsa-circRNA3153_CBC1 | 0.004568328 | 2.177578357 |
| hsa_circ_0138107_CBC1 | 0.000713418 | 2.352149206 |
| hsa-circRNA9968-41_CBC1 | 0.012047398 | 2.072145077 |
| hsa_circ_0090644_CBC1 | 3.22607E-05 | 3.356425098 |
| hsa-circRNA13442-16_CBC1 | 0.021341732 | 2.133965248 |
| hsa_circ_0060455_CBC1 | 0.000419181 | 2.994735246 |
| hsa_circ_0065351_CBC1 | 0.043429525 | 2.69030208 |
| hsa-circRNA626-7_CBC1 | 0.008514685 | 2.120225988 |
| hsa_circ_0097697_CBC1 | 0.003341342 | 2.539152163 |
| hsa-circRNA7601-18_CBC1 | 0.008502747 | 2.105089911 |
| hsa_circ_0080557_CBC1 | 0.000369195 | 2.868817919 |
| hsa-circRNA4049-8_CBC1 | 7.01151E-05 | 10.78309195 |
| hsa_circ_0027569_CBC1 | 0.017821296 | 2.120013988 |
| hsa-circRNA11137-19_CBC1 | 0.006374323 | 2.556829124 |
| hsa_circ_0047929_CBC1 | 0.000720614 | 2.156250012 |
| hsa_circ_0012602_CBC1 | 0.01113912 | 2.521913286 |
| hsa_circ_0135614_CBC1 | 0.041745607 | 2.527488607 |
| hsa_circ_0131169_CBC1 | 0.041311572 | 2.175964035 |
| hsa-circRNA1203-19_CBC1 | 0.000970357 | 2.066250344 |
| hsa_circ_0058056_CBC1 | 0.016768501 | 2.188226098 |
| hsa_circ_0059676_CBC1 | 0.001299682 | 4.083746046 |
| hsa_circ_0010978_CBC1 | 0.001010561 | 2.086464763 |
| hsa_circ_0133656_CBC1 | 0.004702273 | 2.971469517 |
| hsa-circRNA626-22_CBC1 | 0.014460806 | 2.020630854 |
| hsa_circ_0068017_CBC1 | 0.00324514 | 3.030788048 |
| hsa_circ_0036734_CBC1 | 0.000187565 | 6.657303415 |
| hsa_circ_0045205_CBC1 | 0.008769435 | 2.119533542 |
| hsa-circRNA11458-1_CBC1 | 0.003164503 | 4.642754481 |
| hsa-circRNA9917-1_CBC1 | 0.00201318 | 2.110436825 |
| hsa-circRNA15486-158_CBC1 | 0.001884233 | 3.372917016 |
| hsa_circ_0043956_CBC1 | 0.00407431 | 5.316434108 |
| hsa-circRNA10466-21_CBC1 | 0.013854216 | 3.253382753 |
| hsa_circ_0120402_CBC1 | 0.000205325 | 2.286751108 |
| hsa_circ_0035487_CBC1 | 3.08862E-05 | 7.856897898 |
| hsa_circ_0049195_CBC1 | 0.000142847 | 2.492943685 |
| hsa_circ_0061066_CBC1 | 0.010870931 | 2.862782713 |
| hsa_circ_0106909_CBC1 | 0.006689779 | 3.750070517 |
| hsa_circ_0029509_CBC1 | 0.004822151 | 3.525792509 |
| hsa-circRNA5429-1_CBC1 | 0.00489857 | 2.741965031 |
| hsa_circ_0016382_CBC1 | 0.03392622 | 3.561682573 |
| hsa_circ_0085039_CBC1 | 0.000352703 | 2.47134035 |
| hsa-circRNA7836-92_CBC1 | 0.000525278 | 2.121842641 |
| hsa_circ_0019158_CBC1 | 0.005535839 | 3.800168082 |
| hsa_circ_0071678_CBC1 | 1.98381E-05 | 8.139282716 |
| hsa_circ_0029101_CBC1 | 0.000453276 | 4.824784408 |
| hsa-circRNA2866-207_CBC1 | 0.00073117 | 2.105585761 |
| hsa_circ_0018482_CBC1 | 0.000820984 | 2.42865859 |
| hsa-circRNA8500-49_CBC1 | 0.00103794 | 4.624312581 |
| hsa-circRNA7393-8_CBC1 | 0.000809916 | 8.87696579 |
| hsa_circ_0131804_CBC1 | 0.001437926 | 2.682815077 |
| hsa_circ_0046674_CBC1 | 1.84462E-05 | 6.155344613 |
| hsa_circ_0123801_CBC1 | 0.016490136 | 2.293145349 |
| hsa_circ_0090958_CBC1 | 0.000304401 | 8.585096191 |
| hsa_circ_0054470_CBC1 | 0.005916721 | 2.23599379 |
| hsa-circRNA1649-18_CBC1 | 0.000220015 | 3.892127382 |
| hsa-circRNA11493-11_CBC1 | 0.010456219 | 3.855190843 |
| hsa_circ_0114514_CBC1 | 0.010829296 | 4.06279131 |
| hsa_circ_0041008_CBC1 | 0.040334534 | 4.865719607 |
| hsa-circRNA11595-13_CBC1 | 0.001005731 | 3.411036396 |
| hsa-circRNA711-6_CBC1 | 0.000182994 | 2.350352303 |
| hsa-circRNA9443-12_CBC1 | 0.002925611 | 8.826601878 |
| hsa_circ_0039099_CBC1 | 2.07454E-05 | 2.176069697 |
| hsa_circ_0020957_CBC1 | 0.00694619 | 5.753004008 |
| hsa_circ_0034327_CBC1 | 0.004916103 | 3.192770419 |
| hsa_circ_0106819_CBC1 | 0.000221588 | 6.360687777 |
| hsa_circ_0032897_CBC1 | 0.000753711 | 2.48713351 |
| hsa-circRNA7413-8_CBC1 | 0.001788461 | 2.581683584 |
| hsa-circRNA10149-28_CBC1 | 9.18237E-05 | 5.744096782 |
| hsa_circ_0021926_CBC1 | 0.002680696 | 2.005475249 |
| hsa-circRNA7044-4_CBC1 | 0.001495474 | 4.729450006 |
| hsa_circ_0119154_CBC1 | 0.00819488 | 2.412200131 |
| hsa_circ_0013323_CBC1 | 0.002668789 | 2.033103704 |
| hsa-circRNA9867-98_CBC1 | 0.008153083 | 2.104196678 |
| hsa-circRNA1510-5_CBC1 | 0.009774088 | 2.005848331 |
| hsa_circ_0106160_CBC1 | 5.44268E-05 | 5.484675075 |
| hsa_circ_0023988_CBC1 | 0.010605741 | 2.036725741 |
| hsa-circRNA275-17_CBC1 | 0.006394891 | 2.10902356 |
| hsa_circ_0048612_CBC1 | 0.000471294 | 3.093184617 |
| hsa-circRNA10441-7_CBC1 | 0.007306302 | 2.744706914 |
| hsa-circRNA6520-1_CBC1 | 1.75098E-05 | 5.55874714 |
| hsa-circRNA12269-23_CBC1 | 4.22918E-06 | 5.045416895 |
| hsa_circ_0127799_CBC1 | 0.005404945 | 4.414482945 |
| hsa-circRNA10746-2_CBC1 | 0.001406672 | 2.119669637 |
| hsa_circ_0060326_CBC1 | 0.000516711 | 2.768967644 |
| hsa_circ_0124913_CBC1 | 0.000594831 | 8.968848163 |
| hsa_circ_0071994_CBC1 | 0.005131449 | 2.110537635 |
| hsa_circ_0009800_CBC1 | 0.011944142 | 2.231218796 |
| hsa_circ_0041346_CBC1 | 0.015149275 | 2.316625227 |
| hsa-circRNA946-13_CBC1 | 0.004003527 | 4.524738524 |
| hsa_circ_0087441_CBC1 | 0.00119651 | 3.351622052 |
| hsa-circRNA15177-5_CBC1 | 0.002377482 | 2.02980288 |
| hsa_circ_0050708_CBC1 | 5.21416E-05 | 12.06280771 |
| hsa-circRNA12437-22_CBC1 | 0.009430496 | 2.279585022 |
| hsa_circ_0109002_CBC1 | 0.000398961 | 2.813501325 |
| hsa_circ_0026927_CBC1 | 0.004030505 | 2.362960508 |
| hsa_circ_0115130_CBC1 | 0.040648972 | 3.300453284 |
| hsa_circ_0125624_CBC1 | 0.003984629 | 2.566032195 |
| hsa_circ_0048435_CBC1 | 0.003105505 | 2.046180427 |
| hsa_circ_0022803_CBC1 | 0.000419999 | 3.66943559 |
| hsa-circRNA10149-13_CBC1 | 0.001485399 | 12.87096605 |
| hsa_circ_0065035_CBC1 | 0.000197912 | 5.438258189 |
| hsa_circ_0050694_CBC1 | 0.021167453 | 2.54802984 |
| hsa_circ_0064245_CBC1 | 0.000642475 | 5.168918333 |
| hsa_circ_0084766_CBC1 | 0.00943489 | 5.075508035 |
| hsa_circ_0062873_CBC1 | 0.000225457 | 2.842389684 |
| hsa_circ_0008346_CBC1 | 0.000300793 | 3.013215789 |
| hsa-circRNA15486-23_CBC1 | 0.004754026 | 2.024726341 |
| hsa_circ_0062833_CBC1 | 5.78884E-05 | 2.108836372 |
| hsa-circRNA15902-41_CBC1 | 0.002173808 | 2.526177526 |
| hsa_circ_0026587_CBC1 | 0.001470852 | 5.570583277 |
| hsa_circ_0015730_CBC1 | 0.0045767 | 2.575917774 |
| hsa_circ_0090956_CBC1 | 0.000421988 | 3.895057088 |
| hsa_circ_0086781_CBC1 | 0.016421713 | 2.022274475 |
| hsa_circ_0049222_CBC1 | 0.000344697 | 2.800811523 |
| hsa-circRNA2329-9_CBC1 | 0.000464286 | 4.235094959 |
| hsa-circRNA2415-11_CBC1 | 0.014294028 | 2.634689728 |
| hsa_circ_0102375_CBC1 | 0.001540068 | 2.481707035 |
| hsa-circRNA8511-10_CBC1 | 0.013493839 | 2.292401265 |
| hsa_circ_0125975_CBC1 | 2.65853E-06 | 2.157066745 |
| hsa_circ_0083599_CBC1 | 0.001626823 | 2.709666839 |
| hsa_circ_0113454_CBC1 | 0.034206085 | 2.050262723 |
| hsa_circ_0034848_CBC1 | 0.008142277 | 2.742139333 |
| hsa_circ_0012524_CBC1 | 0.003302861 | 7.406013962 |
| hsa_circ_0018880_CBC1 | 0.000122534 | 2.414197417 |
| hsa_circ_0034599_CBC1 | 0.001004076 | 5.173154859 |
| hsa_circ_0068330_CBC1 | 0.001841325 | 2.620375741 |
| hsa_circ_0088938_CBC1 | 0.00417905 | 4.340327936 |
| hsa-circRNA13165-11_CBC1 | 0.002275811 | 3.594943307 |
| hsa_circ_0036918_CBC1 | 3.98743E-05 | 5.716229731 |
| hsa_circ_0075885_CBC1 | 0.01782715 | 2.376439501 |
| hsa-circRNA3771-2_CBC1 | 0.004864475 | 2.37439602 |
| hsa_circ_0026144_CBC1 | 0.001449322 | 4.394250039 |
| hsa-circRNA5308-10_CBC1 | 8.1861E-05 | 4.895247034 |
| hsa_circ_0088316_CBC1 | 0.000225322 | 5.231250374 |
| hsa_circ_0007533_CBC1 | 0.011135391 | 2.010482817 |
| hsa_circ_0015763_CBC1 | 0.000802643 | 4.816503672 |
| hsa_circ_0028411_CBC1 | 0.025197856 | 2.609795511 |
| hsa_circ_0070213_CBC1 | 0.009858678 | 2.045727763 |
| hsa_circ_0069280_CBC1 | 0.002860785 | 8.132220379 |
| hsa_circ_0021536_CBC1 | 0.001434486 | 4.028626967 |
| hsa-circRNA1004-4_CBC1 | 4.73054E-05 | 2.217264901 |
| hsa_circ_0050720_CBC1 | 8.53656E-05 | 5.133155275 |
| hsa_circ_0089148_CBC1 | 0.000743937 | 2.280673806 |
| hsa-circRNA2527-16_CBC1 | 0.001480813 | 7.136209189 |
| hsa_circ_0095973_CBC1 | 0.000645436 | 2.218587864 |
| hsa_circ_0075962_CBC1 | 9.1272E-05 | 12.13540038 |
| hsa_circ_0038062_CBC1 | 0.007741975 | 2.303425001 |
| hsa_circ_0098258_CBC1 | 0.014847143 | 2.67536001 |
| hsa_circ_0049205_CBC1 | 9.47215E-05 | 2.893127541 |
| hsa_circ_0113631_CBC1 | 0.036251 | 2.095730733 |
| hsa_circ_0082469_CBC1 | 0.02280239 | 2.899238537 |
| hsa_circ_0029128_CBC1 | 0.02009419 | 3.609455876 |
| hsa_circ_0004855_CBC1 | 0.012322817 | 5.278136815 |
| hsa_circ_0014745_CBC1 | 0.002287611 | 2.126659358 |
| hsa_circ_0090145_CBC1 | 0.004340483 | 2.960715421 |
| hsa-circRNA10893-14_CBC1 | 1.03425E-05 | 7.069674506 |
| hsa_circ_0133418_CBC1 | 0.004393778 | 2.434629148 |
| hsa_circ_0003109_CBC1 | 0.002996948 | 2.065310375 |
| hsa-circRNA5535-40_CBC1 | 0.002289683 | 2.928314449 |
| hsa_circ_0120409_CBC1 | 0.018509239 | 2.10571673 |
| hsa_circ_0022132_CBC1 | 0.000575062 | 2.367470449 |
| hsa_circ_0041040_CBC1 | 0.003929118 | 6.333604299 |
| hsa-circRNA9867-74_CBC1 | 0.01049373 | 2.006454011 |
| hsa_circ_0043173_CBC1 | 0.003786479 | 2.4515404 |
| hsa_circ_0003088_CBC1 | 0.005180405 | 2.307401989 |
| hsa_circ_0081413_CBC1 | 0.000130694 | 4.022097255 |
| hsa_circ_0029119_CBC1 | 0.009798012 | 2.562928564 |
| hsa-circRNA427-7_CBC1 | 0.002402429 | 3.237296158 |
| hsa-circRNA2033-20_CBC1 | 0.000132107 | 3.15060612 |
| hsa_circ_0102390_CBC1 | 0.002410175 | 2.602903303 |
| hsa_circ_0064220_CBC1 | 0.011535096 | 4.270761645 |
| hsa_circ_0013307_CBC1 | 0.001502136 | 2.39860634 |
| hsa_circ_0026622_CBC1 | 0.004075471 | 2.53521631 |
| hsa_circ_0072576_CBC1 | 0.000102359 | 4.300926721 |
| hsa_circ_0041041_CBC1 | 0.015278292 | 2.636571861 |
| hsa-circRNA2033-96_CBC1 | 0.008974732 | 2.411837711 |
| hsa_circ_0060230_CBC1 | 0.03539511 | 3.772444624 |
| hsa_circ_0115129_CBC1 | 0.016381159 | 3.999264886 |
| hsa-circRNA2719-7_CBC1 | 0.000943917 | 5.185780216 |
| hsa_circ_0025150_CBC1 | 4.61694E-05 | 3.503223164 |
| hsa_circ_0012166_CBC1 | 0.001348993 | 5.549857681 |
| hsa_circ_0029521_CBC1 | 0.006853067 | 5.868326487 |
| hsa_circ_0022962_CBC1 | 0.003680052 | 3.067029244 |
| hsa-circRNA14303-4_CBC1 | 0.010903343 | 4.942205054 |
| hsa_circ_0050702_CBC1 | 0.000203031 | 7.654315035 |
| hsa_circ_0040498_CBC1 | 5.66971E-05 | 2.682991119 |
| hsa_circ_0047930_CBC1 | 0.004218588 | 2.557699683 |
| hsa_circ_0106907_CBC1 | 0.001469047 | 3.183428929 |
| hsa_circ_0026195_CBC1 | 0.000121462 | 5.29574696 |
| hsa_circ_0073947_CBC1 | 0.004690767 | 2.054129747 |
| hsa_circ_0020211_CBC1 | 0.000802318 | 2.444414617 |
| hsa-circRNA2033-100_CBC1 | 0.002133348 | 3.312067378 |
| hsa-circRNA8791-32_CBC1 | 7.34226E-05 | 7.301052389 |
| hsa_circ_0074312_CBC1 | 0.016699184 | 2.215687722 |
| hsa_circ_0045023_CBC1 | 0.01274451 | 3.356378138 |
| hsa_circ_0022419_CBC1 | 3.89219E-06 | 5.781443998 |
| hsa_circ_0049676_CBC1 | 0.003901483 | 3.653512369 |
| hsa-circRNA10467_CBC1 | 3.68252E-06 | 9.384119872 |
| hsa_circ_0113151_CBC1 | 0.001659445 | 2.158815382 |
| hsa_circ_0040500_CBC1 | 0.000285231 | 2.26687327 |
| hsa_circ_0116587_CBC1 | 0.001689283 | 2.0691274 |
| hsa-circRNA5831-21_CBC1 | 0.000343968 | 2.311944638 |
| hsa-circRNA5041-2_CBC1 | 0.000639713 | 3.356585869 |
| hsa_circ_0035040_CBC1 | 0.002619987 | 2.337285945 |
| hsa_circ_0005401_CBC1 | 0.000566854 | 2.781473709 |
| hsa-circRNA1352_CBC1 | 0.000150086 | 2.405274187 |
| hsa_circ_0025288_CBC1 | 0.001615836 | 2.039353598 |
| hsa_circ_0021725_CBC1 | 0.001155295 | 2.794161889 |
| hsa_circ_0050712_CBC1 | 3.2101E-05 | 9.322021048 |
| hsa_circ_0019224_CBC1 | 0.027033592 | 2.04307419 |
| hsa_circ_0085466_CBC1 | 0.011192374 | 3.802708222 |
| hsa_circ_0102378_CBC1 | 0.004767882 | 3.336209984 |
| hsa_circ_0025664_CBC1 | 0.003727316 | 2.141997022 |
| hsa_circ_0012170_CBC1 | 6.61541E-05 | 7.108316088 |
| hsa_circ_0049909_CBC1 | 0.001301706 | 4.090032894 |
| hsa_circ_0025265_CBC1 | 0.003082315 | 2.202330143 |
| hsa_circ_0095738_CBC1 | 0.013480817 | 2.316204573 |
| hsa-circRNA2313-24_CBC1 | 0.001272782 | 2.863653205 |
| hsa_circ_0109088_CBC1 | 0.001516433 | 2.412940853 |
| hsa-circRNA6411-4_CBC1 | 0.028754172 | 3.854262325 |
| hsa_circ_0095894_CBC1 | 0.001163901 | 2.190568335 |
| hsa_circ_0088990_CBC1 | 3.85797E-05 | 2.136638716 |
| hsa_circ_0038196_CBC1 | 0.002256531 | 2.250663521 |
| hsa-circRNA6717-75_CBC1 | 0.002410252 | 2.310883011 |
| hsa_circ_0028677_CBC1 | 0.000110857 | 4.144359766 |
| hsa_circ_0138112_CBC1 | 0.005250648 | 2.1585711 |
| hsa_circ_0039872_CBC1 | 0.004401403 | 2.224671514 |
| hsa_circ_0021342_CBC1 | 0.022115988 | 2.585985081 |
| hsa-circRNA4714_CBC1 | 0.000877107 | 2.308708084 |
| hsa_circ_0108736_CBC1 | 0.045659058 | 2.165230445 |
| hsa-circRNA6201-12_CBC1 | 0.000385609 | 7.225768818 |
| hsa_circ_0016645_CBC1 | 0.007899077 | 2.36611683 |
| hsa_circ_0011151_CBC1 | 0.016121173 | 2.005709592 |
| hsa_circ_0049215_CBC1 | 0.000584566 | 3.840510352 |
| hsa_circ_0029973_CBC1 | 0.013597909 | 3.551000683 |
| hsa_circ_0050798_CBC1 | 1.33997E-06 | 2.092643137 |
| hsa_circ_0139036_CBC1 | 0.000220199 | 2.937273933 |
| hsa_circ_0104377_CBC1 | 0.000483064 | 4.771890922 |
| hsa_circ_0038057_CBC1 | 0.001148842 | 2.394341917 |
| hsa_circ_0036052_CBC1 | 0.000330337 | 2.238561442 |
| hsa_circ_0031702_CBC1 | 0.014435981 | 2.094601112 |
| hsa_circ_0069279_CBC1 | 0.003049447 | 7.017636152 |
| hsa_circ_0009791_CBC1 | 0.012596304 | 2.160467174 |
| hsa_circ_0077696_CBC1 | 0.002251188 | 2.038520843 |
| hsa_circ_0126437_CBC1 | 0.003431563 | 3.765997256 |
| hsa_circ_0134618_CBC1 | 0.000197575 | 2.674242816 |
| hsa_circ_0103429_CBC1 | 0.001214292 | 10.59124531 |
| hsa_circ_0051717_CBC1 | 0.001352724 | 4.829031774 |
| hsa_circ_0028501_CBC1 | 0.002377238 | 2.321745608 |
| hsa-circRNA946-7_CBC1 | 0.000134442 | 4.37398707 |
| hsa_circ_0065835_CBC1 | 0.008277354 | 2.260350272 |
| hsa_circ_0070828_CBC1 | 0.000213704 | 6.762687734 |
| hsa_circ_0102645_CBC1 | 0.008286057 | 2.798773014 |
| hsa_circ_0006025_CBC1 | 0.005105156 | 4.112421887 |
| hsa_circ_0076748_CBC1 | 0.00349667 | 3.008514732 |
| hsa_circ_0137797_CBC1 | 6.60715E-05 | 2.211470636 |
| hsa-circRNA15598-26_CBC1 | 0.01805539 | 4.509172858 |
| hsa_circ_0111960_CBC1 | 0.004978527 | 12.15525641 |
| hsa_circ_0021488_CBC1 | 0.000514045 | 2.730987045 |
| hsa-circRNA10541-5_CBC1 | 0.002578747 | 2.833934149 |
| hsa_circ_0029102_CBC1 | 0.014957889 | 2.96714605 |
| hsa_circ_0052579_CBC1 | 0.000752211 | 6.670621648 |
| hsa_circ_0093344_CBC1 | 0.002393197 | 2.713266617 |
| hsa_circ_0114471_CBC1 | 0.011468827 | 2.390800358 |
| hsa_circ_0028679_CBC1 | 1.29668E-05 | 9.262080484 |
| hsa_circ_0035994_CBC1 | 0.014578513 | 4.019823911 |
| hsa_circ_0012369_CBC1 | 0.000577092 | 3.758516632 |
| hsa_circ_0049199_CBC1 | 0.000206491 | 3.370967829 |
| hsa_circ_0127802_CBC1 | 9.47846E-05 | 3.745779948 |
| hsa_circ_0091817_CBC1 | 0.01755883 | 2.860322892 |
| hsa-circRNA11655-8_CBC1 | 0.011464706 | 2.147473142 |
| hsa_circ_0140532_CBC1 | 0.005646787 | 6.502728685 |
| hsa-circRNA5427-46_CBC1 | 0.000262948 | 4.066601118 |
| hsa_circ_0046678_CBC1 | 4.47317E-06 | 5.724856629 |
| hsa_circ_0012018_CBC1 | 3.93097E-06 | 10.49307449 |
| hsa_circ_0059856_CBC1 | 0.006489791 | 2.868422913 |
| hsa_circ_0004420_CBC1 | 0.002671957 | 2.130064215 |
| hsa-circRNA2407-2_CBC1 | 4.16255E-08 | 2.947605504 |
| hsa_circ_0106945_CBC1 | 0.000562666 | 4.448364478 |
| hsa_circ_0082835_CBC1 | 0.000465158 | 3.581803437 |
| hsa-circRNA10447-9_CBC1 | 0.018446728 | 2.5095866 |
| hsa_circ_0038928_CBC1 | 0.00093031 | 7.483140826 |
| hsa_circ_0073951_CBC1 | 0.013479994 | 2.065571684 |
| hsa_circ_0100697_CBC1 | 0.004311131 | 3.136832933 |
| hsa-circRNA11433-10_CBC1 | 0.00139597 | 2.353222768 |
| hsa_circ_0088979_CBC1 | 0.001103044 | 2.178386502 |
| hsa_circ_0012612_CBC1 | 0.00257557 | 2.142670263 |
| hsa_circ_0063036_CBC1 | 0.002435122 | 2.825217932 |
| hsa_circ_0014232_CBC1 | 0.030674992 | 2.12405894 |
| hsa_circ_0050686_CBC1 | 0.002368328 | 3.587975875 |
| hsa_circ_0059396_CBC1 | 0.032362735 | 2.269804705 |
| hsa-circRNA743-1_CBC1 | 0.001089631 | 8.43237048 |
| hsa_circ_0009033_CBC1 | 0.001038594 | 2.433131395 |
| hsa_circ_0016361_CBC1 | 0.000266028 | 9.349727345 |
| hsa-circRNA3795-15_CBC1 | 0.011503198 | 3.197610397 |
| hsa_circ_0005083_CBC1 | 0.010881151 | 4.988512861 |
| hsa_circ_0015171_CBC1 | 0.00081641 | 2.39321838 |
| hsa-circRNA1034-3_CBC1 | 0.000724027 | 7.801242759 |
| hsa-circRNA4166-16_CBC1 | 0.029487728 | 2.099391004 |
| hsa_circ_0047726_CBC1 | 0.009639249 | 2.543633434 |
| hsa_circ_0022806_CBC1 | 0.007197584 | 4.528335047 |
| hsa-circRNA2313-17_CBC1 | 0.00398443 | 2.914708189 |
| hsa_circ_0089978_CBC1 | 0.00600418 | 2.731536632 |
| hsa_circ_0139454_CBC1 | 0.003075306 | 6.286433459 |
| hsa-circRNA7836-23_CBC1 | 0.00187109 | 3.652723467 |
| hsa_circ_0028687_CBC1 | 0.000507645 | 5.608119316 |
| hsa_circ_0070917_CBC1 | 0.001859495 | 5.552904316 |
| hsa-circRNA2561-2_CBC1 | 0.03827052 | 2.395354115 |
| hsa_circ_0007244_CBC1 | 0.003676607 | 9.484654762 |
| hsa_circ_0000769_CBC1 | 0.000125009 | 2.613696484 |
| hsa_circ_0029129_CBC1 | 0.00603443 | 3.638259307 |
| hsa_circ_0043545_CBC1 | 2.15383E-06 | 4.62485553 |
| hsa_circ_0051815_CBC1 | 3.1108E-05 | 2.22707473 |
| hsa_circ_0020952_CBC1 | 0.004996263 | 2.069735008 |
| hsa-circRNA15727-15_CBC1 | 0.000268337 | 5.377963579 |
| hsa_circ_0002234_CBC1 | 0.001671022 | 5.556647223 |
| hsa_circ_0040492_CBC1 | 0.006058233 | 2.998251704 |
| hsa_circ_0065323_CBC1 | 0.001180495 | 2.202274296 |
| hsa_circ_0045014_CBC1 | 0.001673985 | 3.346099048 |
| hsa_circ_0082494_CBC1 | 0.00109636 | 2.16044445 |
| hsa-circRNA1257-13_CBC1 | 0.000241736 | 2.731369698 |
| hsa_circ_0135664_CBC1 | 0.002943031 | 4.846743676 |
| hsa_circ_0063573_CBC1 | 0.022645915 | 2.860915349 |
| hsa_circ_0053527_CBC1 | 0.035453551 | 2.247957482 |
| hsa-circRNA10441-19_CBC1 | 0.014334005 | 2.126308408 |
| hsa-circRNA6034-75_CBC1 | 0.012723716 | 2.041964585 |
| hsa-circRNA422-2_CBC1 | 0.000223504 | 2.190956669 |
| hsa_circ_0095944_CBC1 | 0.027159427 | 2.64702116 |
| hsa_circ_0022802_CBC1 | 0.001751603 | 3.776124504 |
| hsa_circ_0048430_CBC1 | 0.002988761 | 2.157844026 |
| hsa_circ_0048611_CBC1 | 0.003906549 | 6.489240871 |
| hsa_circ_0066750_CBC1 | 0.001598455 | 5.735687778 |
| hsa_circ_0115512_CBC1 | 0.000851872 | 4.144854247 |
| hsa-circRNA13990-26_CBC1 | 0.006654013 | 2.200806964 |
| hsa-circRNA10819-6_CBC1 | 0.029149075 | 2.139528922 |
| hsa_circ_0090627_CBC1 | 1.61917E-06 | 2.784354348 |
| hsa_circ_0125191_CBC1 | 0.048592602 | 2.673946878 |
| hsa_circ_0097639_CBC1 | 0.009147254 | 2.427091709 |
| hsa_circ_0008002_CBC1 | 0.012726177 | 2.349298744 |
| hsa_circ_0002011_CBC1 | 0.001522182 | 4.566548009 |
| hsa_circ_0042025_CBC1 | 0.001072655 | 2.294777135 |
| hsa_circ_0066748_CBC1 | 0.000859512 | 6.516568023 |
| hsa-circRNA14091-1_CBC1 | 0.001832279 | 14.53693145 |
| hsa_circ_0056654_CBC1 | 0.041758948 | 2.758777026 |
| hsa_circ_0103599_CBC1 | 0.031966396 | 2.318690983 |
| hsa_circ_0095274_CBC1 | 0.020411513 | 2.41667631 |
| hsa_circ_0040693_CBC1 | 0.017695529 | 2.15132775 |
| hsa_circ_0111696_CBC1 | 0.000493029 | 2.16741672 |
| hsa-circRNA8492-1_CBC1 | 0.01437893 | 2.924157895 |
| hsa_circ_0078999_CBC1 | 0.003665499 | 2.286185265 |
| hsa-circRNA2866-51_CBC1 | 0.003377045 | 2.153873529 |
| hsa-circRNA946-6_CBC1 | 0.000344236 | 4.804040471 |
| hsa_circ_0134224_CBC1 | 0.000705402 | 7.396939815 |
| hsa_circ_0041024_CBC1 | 2.31948E-05 | 7.871242837 |
| hsa_circ_0002441_CBC1 | 0.002120436 | 3.162977637 |
| hsa-circRNA9996-7_CBC1 | 0.033371533 | 2.41029473 |
| hsa_circ_0116524_CBC1 | 0.015134712 | 3.489008073 |
| hsa_circ_0040493_CBC1 | 0.00015011 | 3.28514677 |
| hsa-circRNA5028-11_CBC1 | 0.004119379 | 3.140262997 |
| hsa_circ_0044833_CBC1 | 0.000988542 | 2.105943674 |
| hsa-circRNA2639-17_CBC1 | 0.000265008 | 6.230400452 |
| hsa_circ_0070557_CBC1 | 0.000192999 | 10.15001157 |
| hsa_circ_0013388_CBC1 | 0.002264848 | 2.104110887 |
| hsa-circRNA2313-5_CBC1 | 0.002098902 | 2.344336603 |
| hsa_circ_0084483_CBC1 | 0.018200413 | 3.150451978 |
| hsa-circRNA1781-18_CBC1 | 0.000757254 | 2.194235967 |
| hsa-circRNA2033-132_CBC1 | 0.000967753 | 2.434771995 |
| hsa-circRNA15902-39_CBC1 | 0.001171905 | 2.110814384 |
| hsa_circ_0022924_CBC1 | 0.000334653 | 2.56321479 |
| hsa_circ_0029110_CBC1 | 0.001818994 | 2.293153103 |
| hsa_circ_0050687_CBC1 | 0.001380505 | 3.185814341 |
| hsa_circ_0067575_CBC1 | 0.030951457 | 2.190916152 |
| hsa_circ_0091247_CBC1 | 0.005403265 | 4.240486907 |
| hsa_circ_0063527_CBC1 | 0.004438321 | 2.737956838 |
| hsa_circ_0049678_CBC1 | 0.000459712 | 5.423275664 |
| hsa_circ_0125978_CBC1 | 0.014490805 | 2.154247721 |
| hsa_circ_0001921_CBC1 | 0.008559381 | 2.477655132 |
| hsa-circRNA5792-2_CBC1 | 0.000140155 | 2.112313944 |
| hsa-circRNA2639-19_CBC1 | 0.007544834 | 4.663620186 |
| hsa_circ_0137650_CBC1 | 0.003424761 | 2.107380921 |
| hsa-circRNA5806-11_CBC1 | 0.000948411 | 3.002307631 |
| hsa_circ_0098768_CBC1 | 0.001781789 | 3.339362671 |
| hsa_circ_0028379_CBC1 | 0.004090472 | 2.159909034 |
| hsa-circRNA14455-4_CBC1 | 0.000115883 | 2.291662796 |
| hsa_circ_0013202_CBC1 | 0.00037617 | 3.706090849 |
| hsa_circ_0060950_CBC1 | 0.000507843 | 3.684846739 |
| hsa_circ_0043168_CBC1 | 0.007489935 | 2.075083749 |
| hsa-circRNA2033-77_CBC1 | 0.010887757 | 3.218413156 |
| hsa_circ_0064583_CBC1 | 0.001104215 | 3.02090061 |
| hsa-circRNA10988-3_CBC1 | 0.000382721 | 3.172238327 |
| hsa_circ_0090949_CBC1 | 0.000541067 | 7.414511498 |
| hsa_circ_0082002_CBC1 | 0.001877852 | 2.004998303 |
| hsa_circ_0046769_CBC1 | 0.000843314 | 2.427630883 |
| hsa_circ_0097515_CBC1 | 0.000133344 | 4.628528431 |
| hsa_circ_0004291_CBC1 | 0.000433335 | 2.229628759 |
| hsa-circRNA15714-3_CBC1 | 0.007754157 | 2.490165403 |
| hsa_circ_0092302_CBC1 | 0.005394733 | 2.193895492 |
| hsa_circ_0105075_CBC1 | 0.002552915 | 2.733641812 |
| hsa_circ_0068324_CBC1 | 0.000124422 | 2.494804584 |
| hsa_circ_0064210_CBC1 | 0.000297922 | 5.684022937 |
| hsa_circ_0042371_CBC1 | 0.043705981 | 2.689625459 |
| hsa_circ_0041864_CBC1 | 0.001276688 | 2.031927908 |
| hsa_circ_0026937_CBC1 | 0.00281989 | 2.299806967 |
| hsa_circ_0067167_CBC1 | 0.001136638 | 3.288117376 |
| hsa-circRNA12986-1_CBC1 | 0.016434117 | 3.515776895 |
| hsa_circ_0037201_CBC1 | 0.016448202 | 2.003202637 |
| hsa-circRNA3767-7_CBC1 | 0.000348776 | 2.147770413 |
| hsa_circ_0103440_CBC1 | 0.00053895 | 12.73821318 |
| hsa_circ_0125976_CBC1 | 0.00089587 | 2.336863095 |
| hsa_circ_0072693_CBC1 | 0.010515026 | 5.123604068 |
| hsa-circRNA1790-13_CBC1 | 0.000238817 | 4.542785486 |
| hsa_circ_0090631_CBC1 | 6.28186E-06 | 2.927574179 |
| hsa-circRNA11493-24_CBC1 | 0.01110027 | 3.288693426 |
| hsa_circ_0002694_CBC1 | 0.01881437 | 2.361304107 |
| hsa-circRNA5134-3_CBC1 | 0.00613135 | 2.291341814 |
| hsa-circRNA990-1_CBC1 | 0.000208093 | 2.366893949 |
| hsa_circ_0036868_CBC1 | 0.0141801 | 3.510551782 |
| hsa_circ_0136685_CBC1 | 0.001720716 | 4.026845522 |
| hsa_circ_0084645_CBC1 | 0.002792963 | 3.530943198 |
| hsa_circ_0062674_CBC1 | 0.016339743 | 2.409347219 |
| hsa-circRNA9031-21_CBC1 | 0.000976739 | 2.364860395 |
| hsa-circRNA5806-16_CBC1 | 0.013044792 | 2.689732144 |
| hsa_circ_0056037_CBC1 | 1.21622E-05 | 9.56812776 |
| hsa-circRNA6530-2_CBC1 | 0.020248382 | 2.125184666 |
| hsa_circ_0012378_CBC1 | 0.007075355 | 2.986589542 |
| hsa_circ_0032007_CBC1 | 0.049827641 | 2.820353673 |
| hsa-circRNA12292-2_CBC1 | 0.000850883 | 2.105955778 |
| hsa-circRNA10149-30_CBC1 | 0.000101561 | 6.624028443 |
| hsa_circ_0070558_CBC1 | 0.001557873 | 10.46928329 |
| hsa_circ_0070682_CBC1 | 0.028927304 | 2.70449095 |
| hsa-circRNA13178-22_CBC1 | 0.00105876 | 2.057803093 |
| hsa-circRNA2033-48_CBC1 | 0.001276857 | 2.474832237 |
| hsa-circRNA6497-1_CBC1 | 0.001111178 | 2.308795226 |
| hsa_circ_0012600_CBC1 | 0.009345069 | 2.212193117 |
| hsa_circ_0000767_CBC1 | 0.001626227 | 2.025256109 |
| hsa_circ_0015168_CBC1 | 0.002574967 | 2.941338988 |
| hsa_circ_0097650_CBC1 | 0.006379618 | 2.742445127 |
| hsa_circ_0113470_CBC1 | 0.013695745 | 3.121280958 |
| hsa_circ_0059736_CBC1 | 8.92986E-05 | 9.860948043 |
| hsa_circ_0033323_CBC1 | 0.022984772 | 2.059622501 |
| hsa_circ_0013999_CBC1 | 0.025427808 | 2.37512359 |
| hsa_circ_0004053_CBC1 | 0.003177624 | 2.510319144 |
| hsa-circRNA7148-20_CBC1 | 0.0327047 | 2.076657834 |
| hsa_circ_0033240_CBC1 | 0.021702834 | 2.304466824 |
| hsa-circRNA8511-2_CBC1 | 0.002054422 | 6.162566171 |
| hsa_circ_0076737_CBC1 | 0.008971577 | 2.207637253 |
| hsa_circ_0097644_CBC1 | 0.021569185 | 2.311835381 |
| hsa-circRNA10133-4_CBC1 | 0.000275465 | 2.468343013 |
| hsa_circ_0046706_CBC1 | 0.003697976 | 9.544414205 |
| hsa-circRNA7825-13_CBC1 | 0.000377063 | 2.780036295 |
| hsa_circ_0050617_CBC1 | 0.014438177 | 2.128005392 |
| hsa-circRNA11521-1_CBC1 | 0.000173791 | 3.71450735 |
| hsa_circ_0040581_CBC1 | 0.00056525 | 3.325023051 |
| hsa_circ_0010843_CBC1 | 0.010719909 | 2.049764674 |
| hsa_circ_0009838_CBC1 | 0.004624908 | 2.188891951 |
| hsa-circRNA851-42_CBC1 | 0.023367267 | 2.242211028 |
| hsa_circ_0002512_CBC1 | 0.003299773 | 4.958495337 |
| hsa-circRNA8877-8_CBC1 | 0.033920774 | 2.228191281 |
| hsa_circ_0090146_CBC1 | 0.011159907 | 4.758936243 |
| hsa-circRNA7713-1_CBC1 | 0.000688726 | 4.806731673 |
| hsa-circRNA11180-2_CBC1 | 0.001718968 | 2.475161039 |
| hsa-circRNA10466-11_CBC1 | 0.02896913 | 2.989386986 |
| hsa_circ_0041991_CBC1 | 1.68952E-05 | 3.750359112 |
| hsa_circ_0029512_CBC1 | 0.003814218 | 5.450127093 |
| hsa-circRNA7663-13_CBC1 | 0.028788491 | 2.956186522 |
| hsa_circ_0101885_CBC1 | 0.030480705 | 2.677983874 |
| hsa-circRNA11458-19_CBC1 | 0.002892367 | 4.236641297 |
| hsa-circRNA2639-8_CBC1 | 0.009258675 | 4.598609714 |
| hsa_circ_0097654_CBC1 | 0.012014751 | 2.42355076 |
| hsa_circ_0076436_CBC1 | 0.001279909 | 2.558170496 |
| hsa_circ_0102358_CBC1 | 0.000253297 | 5.57233898 |
| hsa_circ_0032095_CBC1 | 0.007402804 | 2.519456145 |
| hsa_circ_0060447_CBC1 | 0.000104206 | 4.041802576 |
| hsa-circRNA13121-10_CBC1 | 0.002236337 | 2.513717304 |
| hsa_circ_0065737_CBC1 | 0.014902672 | 2.603574767 |
| hsa-circRNA1257-2_CBC1 | 0.000143099 | 3.136841729 |
| hsa_circ_0034568_CBC1 | 0.000906933 | 7.802129018 |
| hsa_circ_0051732_CBC1 | 1.57738E-05 | 5.958800448 |
| hsa_circ_0043540_CBC1 | 0.004081097 | 4.642404413 |
| hsa-circRNA15162-122_CBC1 | 0.000311698 | 2.263215364 |
| hsa_circ_0059377_CBC1 | 0.026720024 | 2.043976261 |
| hsa_circ_0036099_CBC1 | 0.031103288 | 2.008663644 |
| hsa_circ_0035750_CBC1 | 0.029428244 | 2.270380726 |
| hsa_circ_0049194_CBC1 | 0.000382784 | 4.141603818 |
| hsa_circ_0068022_CBC1 | 0.001846072 | 2.962489785 |
| hsa_circ_0026442_CBC1 | 0.004146461 | 2.484821714 |
| hsa_circ_0082540_CBC1 | 0.004497953 | 2.009304763 |
| hsa_circ_0080553_CBC1 | 0.000314936 | 2.219402452 |
| hsa_circ_0066938_CBC1 | 0.004196722 | 5.658381881 |
| hsa_circ_0012020_CBC1 | 0.000195665 | 9.023336581 |
| hsa_circ_0002563_CBC1 | 0.01078037 | 4.731963201 |
| hsa_circ_0024917_CBC1 | 0.000330588 | 4.040401655 |
| hsa_circ_0029175_CBC1 | 0.038146613 | 2.207062791 |
| hsa_circ_0129341_CBC1 | 0.012204119 | 2.472421484 |
| hsa_circ_0111020_CBC1 | 0.002856412 | 2.194358487 |
| hsa_circ_0006699_CBC1 | 0.003715622 | 2.14262378 |
| hsa_circ_0063913_CBC1 | 0.018397794 | 2.053820964 |
| hsa_circ_0046103_CBC1 | 2.98414E-06 | 2.854903481 |
| hsa_circ_0112415_CBC1 | 0.002278726 | 2.115451721 |
| hsa_circ_0002764_CBC1 | 0.024946668 | 3.496012998 |
| hsa-circRNA5806-12_CBC1 | 0.005089608 | 2.69577253 |
| hsa_circ_0070915_CBC1 | 0.013378383 | 2.800387156 |
| hsa_circ_0051721_CBC1 | 0.000137541 | 6.06135448 |
| hsa_circ_0044835_CBC1 | 6.84678E-05 | 3.150144636 |
| hsa_circ_0137791_CBC1 | 0.007836808 | 2.151823254 |
| hsa_circ_0060456_CBC1 | 9.88961E-05 | 8.461041687 |
| hsa-circRNA11521-12_CBC1 | 0.000868712 | 4.133394579 |
| hsa_circ_0088940_CBC1 | 0.002344131 | 2.145639651 |
| hsa-circRNA8518-27_CBC1 | 0.000338206 | 2.3692281 |
| hsa_circ_0073884_CBC1 | 0.002126428 | 2.48329239 |
| hsa_circ_0014753_CBC1 | 0.000998351 | 5.08483865 |
| hsa-circRNA7044-11_CBC1 | 0.000107308 | 7.92374607 |
| hsa-circRNA11493-32_CBC1 | 0.01266782 | 3.699395589 |
| hsa-circRNA3260-3_CBC1 | 2.24746E-07 | 2.026621048 |
| hsa_circ_0043533_CBC1 | 0.00273338 | 8.875609827 |
| hsa_circ_0035044_CBC1 | 0.043814673 | 2.164964736 |
| hsa_circ_0003457_CBC1 | 0.000137739 | 4.718481784 |
| hsa_circ_0015173_CBC1 | 4.07425E-05 | 3.800425485 |
| hsa-circRNA8500-1_CBC1 | 0.001063456 | 3.979447604 |
| hsa_circ_0088954_CBC1 | 0.000629915 | 2.179236613 |
| hsa_circ_0058046_CBC1 | 0.023805696 | 2.194532776 |
| hsa_circ_0029108_CBC1 | 0.001687358 | 2.248008717 |
| hsa-circRNA10466-25_CBC1 | 0.001199769 | 4.034141842 |
| hsa-circRNA7275-34_CBC1 | 0.00136678 | 2.575063997 |
| hsa-circRNA8491_CBC1 | 0.000137115 | 3.518455626 |
| hsa_circ_0043955_CBC1 | 0.000319636 | 5.330033671 |
| hsa_circ_0052588_CBC1 | 1.69262E-05 | 6.384777916 |
| hsa-circRNA5806-22_CBC1 | 0.001297716 | 3.302671484 |
| hsa_circ_0104376_CBC1 | 0.000697401 | 3.592913327 |
| hsa_circ_0136674_CBC1 | 0.003282684 | 2.200220653 |
| hsa_circ_0105094_CBC1 | 0.001393179 | 2.211625698 |
| hsa-circRNA1940-1_CBC1 | 0.002451879 | 3.014916306 |
| hsa_circ_0047950_CBC1 | 0.001769253 | 2.395300516 |
| hsa-circRNA11595-16_CBC1 | 0.00244084 | 3.582657477 |
| hsa_circ_0029774_CBC1 | 0.046370183 | 3.267948821 |
| hsa_circ_0046675_CBC1 | 2.21429E-05 | 5.465176477 |
| hsa-circRNA13330-8_CBC1 | 0.001074776 | 2.533536123 |
| hsa_circ_0021497_CBC1 | 6.62386E-05 | 2.928396624 |
| hsa_circ_0005686_CBC1 | 0.000848448 | 2.223365504 |
| hsa-circRNA7706-3_CBC1 | 0.020544098 | 2.157947376 |
| hsa-circRNA4065-18_CBC1 | 0.001975747 | 2.154524255 |
| hsa_circ_0038633_CBC1 | 6.43578E-05 | 11.25414714 |
| hsa_circ_0111693_CBC1 | 0.009760046 | 2.138084749 |
| hsa_circ_0105092_CBC1 | 0.008926177 | 2.37765958 |
| hsa-circRNA2866-265_CBC1 | 0.001934425 | 2.085159495 |
| hsa-circRNA6777-16_CBC1 | 0.006846516 | 3.987943272 |
| hsa-circRNA5028-15_CBC1 | 0.000286026 | 18.78673951 |
| hsa_circ_0074090_CBC1 | 1.14557E-05 | 10.35777656 |
| hsa-circRNA7777-15_CBC1 | 0.000482982 | 3.006613176 |
| hsa_circ_0133919_CBC1 | 0.000753215 | 2.025126605 |
| hsa-circRNA14781-3_CBC1 | 0.014716475 | 2.86439467 |
| hsa_circ_0065030_CBC1 | 0.00672384 | 3.670385979 |
| hsa_circ_0013390_CBC1 | 0.001533685 | 2.95432969 |
| hsa-circRNA5707-6_CBC1 | 0.001128641 | 4.401640889 |
| hsa_circ_0059884_CBC1 | 0.012672679 | 2.042886003 |
| hsa_circ_0015578_CBC1 | 0.000445492 | 2.223208079 |
| hsa_circ_0043670_CBC1 | 0.002785466 | 2.074320447 |
| hsa_circ_0032167_CBC1 | 0.004750778 | 3.992122773 |
| hsa_circ_0076626_CBC1 | 3.06468E-06 | 2.085638172 |
| hsa-circRNA8092-22_CBC1 | 0.005763646 | 2.187359166 |
| hsa_circ_0111432_CBC1 | 0.021515178 | 2.083792947 |
| hsa_circ_0000411_CBC1 | 0.000171451 | 2.972871921 |
| hsa-circRNA5213-19_CBC1 | 0.001640548 | 4.789997913 |
| hsa-circRNA2424-4_CBC1 | 0.014190019 | 2.302303592 |
| hsa-circRNA2536-4_CBC1 | 0.000102734 | 2.41161704 |
| hsa-circRNA1623-6_CBC1 | 0.015665982 | 2.462536652 |
| hsa_circ_0014758_CBC1 | 0.000152733 | 3.603499174 |
| hsa-circRNA11838-57_CBC1 | 0.042677865 | 2.135458222 |
| hsa-circRNA10466-3_CBC1 | 0.022038328 | 2.805125211 |
| hsa_circ_0082293_CBC1 | 0.019958878 | 2.947443247 |
| hsa-circRNA8885-28_CBC1 | 0.001036337 | 4.97590479 |
| hsa_circ_0007306_CBC1 | 2.13618E-05 | 2.452785924 |
| hsa_circ_0104337_CBC1 | 0.004514539 | 2.592332372 |
| hsa_circ_0004904_CBC1 | 0.011721431 | 2.410852052 |
| hsa_circ_0095560_CBC1 | 1.27456E-06 | 2.977505552 |
| hsa-circRNA4049-38_CBC1 | 0.00021719 | 20.12401537 |
| hsa-circRNA3767-11_CBC1 | 0.009479644 | 2.160581522 |
| hsa_circ_0082208_CBC1 | 0.001985666 | 2.800629126 |
| hsa_circ_0046104_CBC1 | 0.002203045 | 2.035692078 |
| hsa_circ_0027110_CBC1 | 0.002435196 | 3.888614274 |
| hsa-circRNA14645-6_CBC1 | 0.00340402 | 2.501170171 |
| hsa_circ_0043178_CBC1 | 0.013802685 | 2.078192667 |
| hsa_circ_0081408_CBC1 | 0.000247351 | 3.902432903 |
| hsa-circRNA2561-3_CBC1 | 0.012425672 | 2.020831847 |
| hsa_circ_0028705_CBC1 | 2.87664E-06 | 6.786619235 |
| hsa_circ_0071295_CBC1 | 0.000220612 | 2.29901727 |
| hsa_circ_0110153_CBC1 | 0.002736134 | 5.820870919 |
| hsa-circRNA5149-19_CBC1 | 0.000760171 | 3.725210728 |
| hsa_circ_0096967_CBC1 | 0.000986066 | 3.512989265 |
| hsa_circ_0088973_CBC1 | 0.036127899 | 2.354982685 |
| hsa_circ_0020488_CBC1 | 0.000259939 | 24.53645623 |
| hsa-circRNA15902-56_CBC1 | 0.003718872 | 2.360173069 |
| hsa_circ_0015826_CBC1 | 0.000595441 | 3.399461442 |
| hsa-circRNA4035-1_CBC1 | 1.21704E-05 | 2.481018065 |
| hsa_circ_0026199_CBC1 | 0.000567859 | 7.314645616 |
| hsa-circRNA2599-8_CBC1 | 4.96187E-05 | 6.304655079 |
| hsa_circ_0108579_CBC1 | 0.000263511 | 4.837684822 |
| hsa_circ_0061938_CBC1 | 0.040981171 | 2.174553097 |
| hsa-circRNA1357-5_CBC1 | 0.000241773 | 4.855332667 |
| hsa_circ_0014567_CBC1 | 0.010103447 | 2.299914463 |
| hsa_circ_0046128_CBC1 | 0.000274553 | 2.327347847 |
| hsa_circ_0048409_CBC1 | 0.00049798 | 3.969764683 |
| hsa_circ_0138109_CBC1 | 0.000173491 | 2.289246637 |
| hsa-circRNA7663-34_CBC1 | 0.008168011 | 2.973865371 |
| hsa-circRNA11838-47_CBC1 | 0.001926105 | 5.098625037 |
| hsa-circRNA12558-6_CBC1 | 0.003890769 | 2.236643569 |
| hsa_circ_0042338_CBC1 | 0.001741304 | 3.997123326 |
| hsa-circRNA11084-9_CBC1 | 0.000671822 | 4.800762428 |
| hsa_circ_0064244_CBC1 | 0.000122603 | 13.26926723 |
| hsa_circ_0017552_CBC1 | 0.006451323 | 2.09339426 |
| hsa-circRNA11467-84_CBC1 | 0.00066569 | 2.141650294 |
| hsa-circRNA2407-16_CBC1 | 0.000250303 | 2.527651689 |
| hsa_circ_0027824_CBC1 | 0.002233325 | 4.956906929 |
| hsa-circRNA9333-5_CBC1 | 3.65237E-05 | 13.68487494 |
| hsa-circRNA11242-106_CBC1 | 0.002583098 | 6.433336609 |
| hsa_circ_0081183_CBC1 | 0.010269736 | 2.047249134 |
| hsa_circ_0073948_CBC1 | 0.002350392 | 2.359884797 |
| hsa_circ_0043601_CBC1 | 0.008980661 | 2.085372057 |
| hsa-circRNA1612-2_CBC1 | 0.012744217 | 2.5743081 |
| hsa_circ_0130046_CBC1 | 0.021648946 | 2.721063172 |
| hsa-circRNA1376-17_CBC1 | 0.001071569 | 2.036652756 |
| hsa-circRNA15355-29_CBC1 | 0.000157397 | 3.050959603 |
| hsa_circ_0009035_CBC1 | 0.018677931 | 2.392154507 |
| hsa_circ_0049186_CBC1 | 0.000156949 | 4.654968502 |
| hsa_circ_0059963_CBC1 | 0.001597304 | 2.075175252 |
| hsa_circ_0012364_CBC1 | 0.003124082 | 5.481665962 |
| hsa_circ_0022619_CBC1 | 0.002944235 | 2.007929433 |
| hsa-circRNA2520-6_CBC1 | 3.22213E-05 | 15.87363787 |
| hsa_circ_0015899_CBC1 | 0.006308454 | 2.088093172 |
| hsa_circ_0090634_CBC1 | 2.50966E-05 | 3.500708337 |
| hsa-circRNA743-3_CBC1 | 0.000163183 | 6.30319458 |
| hsa-circRNA13698-35_CBC1 | 0.006687522 | 6.352389228 |
| hsa_circ_0001266_CBC1 | 9.2503E-05 | 4.371005866 |
| hsa_circ_0097703_CBC1 | 0.037668956 | 2.019795563 |
| hsa_circ_0004930_CBC1 | 0.001205225 | 2.980647293 |
| hsa_circ_0102384_CBC1 | 0.000339396 | 5.330711135 |
| hsa_circ_0076632_CBC1 | 0.001388148 | 3.217123217 |
| hsa_circ_0011716_CBC1 | 2.95171E-06 | 9.864215819 |
| hsa-circRNA10312-15_CBC1 | 0.007235101 | 2.053470295 |
| hsa-circRNA9821_CBC1 | 2.02659E-05 | 9.795432204 |
| hsa_circ_0104841_CBC1 | 0.001994363 | 4.128085903 |
| hsa-circRNA4758-3_CBC1 | 0.005956205 | 2.393732308 |
| hsa_circ_0033141_CBC1 | 0.022541321 | 2.279352113 |
| hsa_circ_0042330_CBC1 | 0.016558643 | 2.107409914 |
| hsa_circ_0053014_CBC1 | 0.01043153 | 3.621623742 |
| hsa_circ_0102362_CBC1 | 0.018097635 | 3.407302617 |
| hsa_circ_0090622_CBC1 | 0.000952465 | 3.054704163 |
| hsa_circ_0029695_CBC1 | 0.003647735 | 5.59554743 |
| hsa_circ_0032012_CBC1 | 0.03453792 | 2.112063675 |
| hsa_circ_0032207_CBC1 | 0.041175804 | 2.056293768 |
| hsa_circ_0088945_CBC1 | 0.002786457 | 2.308958981 |
| hsa_circ_0072769_CBC1 | 0.001566904 | 2.868726911 |
| hsa-circRNA14844-14_CBC1 | 0.02581048 | 3.074918261 |
| hsa_circ_0106967_CBC1 | 0.001387918 | 2.200382417 |
| hsa_circ_0066943_CBC1 | 0.039668179 | 4.378129467 |
| hsa_circ_0012168_CBC1 | 0.002919096 | 4.701468014 |
| hsa-circRNA2536-16_CBC1 | 0.00020476 | 5.71980678 |
| hsa-circRNA8881-1_CBC1 | 0.000367959 | 13.8174334 |
| hsa_circ_0088884_CBC1 | 0.002604964 | 3.117856463 |
| hsa-circRNA14439-1_CBC1 | 0.000257066 | 10.03591571 |
| hsa_circ_0068014_CBC1 | 0.00116081 | 4.60732077 |
| hsa_circ_0117791_CBC1 | 0.003707727 | 3.465223486 |
| hsa-circRNA626-3_CBC1 | 0.00100048 | 2.183111752 |
| hsa-circRNA9555-4_CBC1 | 0.039833265 | 2.898775596 |
| hsa_circ_0052390_CBC1 | 0.022663201 | 2.073561482 |
| hsa_circ_0134616_CBC1 | 0.040659671 | 3.248377934 |
| hsa_circ_0003091_CBC1 | 0.000621804 | 2.55922808 |
| hsa-circRNA2033-27_CBC1 | 0.007654583 | 3.718648984 |
| hsa_circ_0041009_CBC1 | 0.002374053 | 3.338537078 |
| hsa-circRNA836-2_CBC1 | 0.004945369 | 2.052450521 |
| hsa_circ_0108735_CBC1 | 0.009676156 | 2.069542041 |
| hsa-circRNA8518-1_CBC1 | 0.001731436 | 2.181695636 |
| hsa_circ_0042794_CBC1 | 0.003269696 | 2.637951424 |
| hsa_circ_0050974_CBC1 | 0.027650133 | 2.855341286 |
| hsa-circRNA7275-53_CBC1 | 0.020580748 | 2.71454355 |
| hsa_circ_0044575_CBC1 | 0.048537039 | 2.391266584 |
| hsa_circ_0102728_CBC1 | 0.013900875 | 3.739378528 |
| hsa-circRNA4701-4_CBC1 | 0.04073854 | 2.165151875 |
| hsa_circ_0102834_CBC1 | 8.25236E-05 | 2.9078359 |
| hsa-circRNA12677-113_CBC1 | 0.000529282 | 8.2224518 |
| hsa-circRNA14838-132_CBC1 | 0.003227793 | 2.437201318 |
| hsa_circ_0043660_CBC1 | 0.001742567 | 2.178609493 |
| hsa-circRNA2520-21_CBC1 | 0.000314008 | 4.496489248 |
| hsa_circ_0129445_CBC1 | 4.17075E-05 | 6.774416456 |
| hsa_circ_0036925_CBC1 | 0.000101483 | 6.226812105 |
| hsa_circ_0055672_CBC1 | 1.86498E-05 | 8.1938926 |
| hsa_circ_0106159_CBC1 | 0.000532783 | 2.039517891 |
| hsa_circ_0021689_CBC1 | 0.012439958 | 2.028527003 |
| hsa_circ_0030278_CBC1 | 0.010869721 | 2.936574619 |
| hsa_circ_0026322_CBC1 | 0.000671074 | 4.841118583 |
| hsa-circRNA9376-78_CBC1 | 0.000657379 | 2.047037136 |
| hsa-circRNA13698-1_CBC1 | 0.001502043 | 7.107693723 |
| hsa-circRNA14279-1_CBC1 | 0.026882688 | 2.616975301 |
| hsa_circ_0088987_CBC1 | 8.0578E-05 | 2.239945017 |
| hsa-circRNA8791-28_CBC1 | 5.13072E-05 | 10.27530786 |
| hsa_circ_0015770_CBC1 | 0.001105252 | 15.18253833 |
| hsa_circ_0100097_CBC1 | 0.003008373 | 2.983601573 |
| hsa_circ_0026134_CBC1 | 0.015250989 | 3.784424853 |
| hsa-circRNA3533-5_CBC1 | 0.008079737 | 2.302993944 |
| hsa_circ_0116799_CBC1 | 0.000491763 | 2.584091599 |
| hsa_circ_0058980_CBC1 | 0.000842567 | 6.526677074 |
| hsa_circ_0070919_CBC1 | 0.005212675 | 7.101932573 |
| hsa-circRNA2407-15_CBC1 | 0.000239162 | 2.396542543 |
| hsa_circ_0114513_CBC1 | 0.008034944 | 3.833589429 |
| hsa-circRNA2536-21_CBC1 | 0.000120741 | 3.060648651 |
| hsa_circ_0086010_CBC1 | 0.005415486 | 2.294414919 |
| hsa_circ_0029488_CBC1 | 0.000111776 | 3.705744467 |
| hsa_circ_0050100_CBC1 | 0.022079654 | 2.625810364 |
| hsa_circ_0105074_CBC1 | 0.000297597 | 2.098913757 |
| hsa-circRNA4547-16_CBC1 | 0.000417789 | 11.97450646 |
| hsa-circRNA2033-6_CBC1 | 0.004197691 | 4.394236498 |
| hsa_circ_0044702_CBC1 | 0.006155806 | 2.235594775 |
| hsa_circ_0025040_CBC1 | 1.01401E-06 | 4.695120416 |
| hsa_circ_0088965_CBC1 | 0.007655905 | 2.501688788 |
| hsa-circRNA14131-15_CBC1 | 0.011457873 | 2.184108301 |
| hsa-circRNA2407-6_CBC1 | 0.000314863 | 2.332912532 |
| hsa-circRNA5535-35_CBC1 | 0.00885199 | 3.286269547 |
| hsa-circRNA12292-1_CBC1 | 6.4984E-05 | 2.027150984 |
| hsa_circ_0036698_CBC1 | 0.001027279 | 5.969781998 |
| hsa_circ_0001728_CBC1 | 0.000181628 | 3.739297237 |
| hsa-circRNA5308-20_CBC1 | 0.000317873 | 3.275323429 |
| hsa-circRNA7445-5_CBC1 | 0.024309899 | 2.528591869 |
| hsa-circRNA2974_CBC1 | 0.019359409 | 2.113667128 |
| hsa_circ_0006285_CBC1 | 0.001020657 | 6.914420104 |
| hsa_circ_0045605_CBC1 | 0.016217884 | 2.108065785 |
| hsa_circ_0068469_CBC1 | 0.025634159 | 2.137589938 |
| hsa_circ_0082524_CBC1 | 0.001220116 | 2.44486924 |
| hsa_circ_0018490_CBC1 | 0.000340376 | 2.934311974 |
| hsa_circ_0018034_CBC1 | 0.003273482 | 2.933620498 |
| hsa_circ_0125037_CBC1 | 0.031760821 | 2.29723495 |
| hsa_circ_0040486_CBC1 | 0.000264556 | 3.065090387 |
| hsa_circ_0091667_CBC1 | 0.002306545 | 3.446610354 |
| hsa-circRNA11595-10_CBC1 | 0.001016307 | 3.104197645 |
| hsa_circ_0060461_CBC1 | 2.11434E-05 | 2.90441899 |
| hsa-circRNA3215-24_CBC1 | 0.002383436 | 2.166596756 |
| hsa_circ_0047535_CBC1 | 0.00089658 | 2.183514156 |
| hsa_circ_0038704_CBC1 | 0.022839981 | 2.939160532 |
| hsa-circRNA15655-5_CBC1 | 0.00158134 | 2.306133266 |
| hsa_circ_0098846_CBC1 | 0.019378968 | 2.375032063 |
| hsa_circ_0014744_CBC1 | 0.001194778 | 6.668625907 |
| hsa_circ_0100250_CBC1 | 0.029217866 | 2.753481667 |
| hsa_circ_0024748_CBC1 | 0.031635926 | 2.378712093 |
| hsa-circRNA2718-9_CBC1 | 0.038460628 | 4.197847804 |
| hsa_circ_0103040_CBC1 | 0.020984035 | 2.722416307 |
| hsa_circ_0102371_CBC1 | 6.11434E-05 | 3.864327652 |
| hsa_circ_0021726_CBC1 | 0.001314291 | 4.135087082 |
| hsa_circ_0079197_CBC1 | 0.046240182 | 2.079086382 |
| hsa-circRNA8297-14_CBC1 | 0.000961176 | 2.062941579 |
| hsa_circ_0049196_CBC1 | 0.001524704 | 4.777922835 |
| hsa-circRNA7445-30_CBC1 | 0.001597694 | 3.449963526 |
| hsa-circRNA15906-16_CBC1 | 0.000616828 | 2.748731981 |
| hsa_circ_0107329_CBC1 | 0.008637742 | 2.751205432 |
| hsa_circ_0101889_CBC1 | 0.044477837 | 3.541083386 |
| hsa_circ_0009827_CBC1 | 0.001354817 | 2.039162987 |
| hsa_circ_0089518_CBC1 | 0.000241724 | 2.368584864 |
| hsa_circ_0066932_CBC1 | 0.006790832 | 6.942717965 |
| hsa_circ_0029143_CBC1 | 0.004715239 | 2.668144548 |
| hsa_circ_0056354_CBC1 | 0.005214057 | 2.720408477 |
| hsa_circ_0100254_CBC1 | 0.001669849 | 5.35568373 |
| hsa_circ_0001235_CBC1 | 7.79905E-05 | 2.096062238 |
| hsa_circ_0104340_CBC1 | 0.013155766 | 2.68973914 |
| hsa-circRNA2528-12_CBC1 | 0.004235859 | 8.331523729 |
| hsa_circ_0088330_CBC1 | 0.002064651 | 3.509102216 |
| hsa_circ_0038058_CBC1 | 0.001756191 | 2.437899837 |
| hsa_circ_0067577_CBC1 | 0.025898638 | 2.096450686 |
| hsa_circ_0039709_CBC1 | 0.040198959 | 2.183737811 |
| hsa-circRNA14824-3_CBC1 | 0.022468013 | 2.406757658 |
| hsa_circ_0097999_CBC1 | 0.000265006 | 3.008937877 |
| hsa_circ_0071683_CBC1 | 0.000221988 | 10.25708659 |
| hsa_circ_0005346_CBC1 | 0.007673349 | 4.439799361 |
| hsa_circ_0011480_CBC1 | 0.001777354 | 2.112407541 |
| hsa_circ_0049202_CBC1 | 0.000464284 | 3.03420346 |
| hsa_circ_0060207_CBC1 | 2.53586E-05 | 3.564829581 |
| hsa-circRNA11493-26_CBC1 | 0.001559106 | 2.939858737 |
| hsa_circ_0051729_CBC1 | 8.44967E-05 | 3.518924823 |
| hsa_circ_0063033_CBC1 | 3.89292E-05 | 4.172815186 |
| hsa-circRNA10466-24_CBC1 | 0.043934439 | 2.553148429 |
| hsa_circ_0060449_CBC1 | 0.000835841 | 7.011908542 |
| hsa_circ_0025671_CBC1 | 0.018211297 | 3.128878881 |
| hsa-circRNA7970-16_CBC1 | 0.038619149 | 2.33273165 |
| hsa_circ_0066757_CBC1 | 0.001568898 | 6.926672024 |
| hsa_circ_0069281_CBC1 | 0.00214124 | 12.71930416 |
| hsa-circRNA15727-14_CBC1 | 6.87032E-05 | 8.477375464 |
| hsa_circ_0087029_CBC1 | 0.035081344 | 2.238843233 |
| hsa_circ_0084587_CBC1 | 0.001550613 | 2.90051833 |
| hsa_circ_0040482_CBC1 | 0.000629135 | 3.402408173 |
| hsa-circRNA946-10_CBC1 | 0.002044096 | 5.110347654 |
| hsa_circ_0056034_CBC1 | 1.21381E-05 | 8.816788118 |
| hsa_circ_0069274_CBC1 | 0.005576054 | 6.693684417 |
| hsa_circ_0093983_CBC1 | 0.031272709 | 2.282848927 |
| hsa_circ_0132712_CBC1 | 0.042288981 | 3.849325269 |
| hsa_circ_0090633_CBC1 | 4.11043E-06 | 3.682513957 |
| hsa_circ_0133410_CBC1 | 0.010632713 | 2.303372963 |
| hsa-circRNA11393-5_CBC1 | 0.000702692 | 6.996759218 |
| hsa_circ_0034698_CBC1 | 0.002717089 | 3.666225641 |
| hsa_circ_0057751_CBC1 | 0.042803557 | 2.0710617 |
| hsa_circ_0084643_CBC1 | 0.032282916 | 2.674228087 |
| hsa_circ_0015764_CBC1 | 7.38629E-05 | 9.275928243 |
| hsa_circ_0025154_CBC1 | 0.001491509 | 3.852703117 |
| hsa_circ_0088984_CBC1 | 0.000594378 | 2.231261723 |
| hsa_circ_0112244_CBC1 | 0.012937883 | 2.700448002 |
| hsa_circ_0140578_CBC1 | 0.005483754 | 2.28737498 |
| hsa-circRNA2805-27_CBC1 | 0.001555386 | 3.055369132 |
| hsa-circRNA8518-49_CBC1 | 0.019140008 | 3.316219814 |
| hsa_circ_0002547_CBC1 | 0.000641844 | 2.759033701 |
| hsa_circ_0118201_CBC1 | 0.006650796 | 3.173986816 |
| hsa_circ_0018972_CBC1 | 0.00074491 | 2.437972198 |
| hsa-circRNA6498-3_CBC1 | 8.40605E-05 | 2.546458406 |
| hsa-circRNA654-38_CBC1 | 0.028164761 | 2.001020563 |
| hsa_circ_0043865_CBC1 | 5.58453E-05 | 2.272886947 |
| hsa_circ_0012092_CBC1 | 0.00306231 | 2.050010333 |
| hsa_circ_0140470_CBC1 | 0.006999487 | 3.19244409 |
| hsa_circ_0032157_CBC1 | 0.003935061 | 4.636196616 |
| hsa_circ_0001593_CBC1 | 0.014391778 | 2.433588919 |
| hsa-circRNA6107-21_CBC1 | 0.000482246 | 2.996338093 |
| hsa_circ_0022346_CBC1 | 0.007944918 | 2.591667577 |
| hsa-circRNA2033-43_CBC1 | 0.016896953 | 2.714266753 |
| hsa_circ_0116181_CBC1 | 0.017235269 | 2.18851511 |
| hsa_circ_0026838_CBC1 | 0.002246139 | 2.106980476 |
| hsa_circ_0093917_CBC1 | 0.002091918 | 2.604408204 |
| hsa_circ_0091238_CBC1 | 0.004516837 | 9.170897516 |
| hsa_circ_0054623_CBC1 | 0.026479966 | 3.086073265 |
| hsa_circ_0090144_CBC1 | 0.027588946 | 3.813321323 |
| hsa-circRNA3588-1_CBC1 | 0.002263029 | 2.916635183 |
| hsa_circ_0026797_CBC1 | 0.035383995 | 3.277698495 |
| hsa_circ_0094592_CBC1 | 0.038608304 | 4.083137648 |
| hsa-circRNA7228-2_CBC1 | 0.007101451 | 2.532973775 |
| hsa_circ_0034326_CBC1 | 0.011803864 | 4.122116302 |
| hsa_circ_0020377_CBC1 | 0.001539079 | 2.19079535 |
| hsa-circRNA3585-6_CBC1 | 3.50261E-05 | 5.638873536 |
| hsa_circ_0047952_CBC1 | 0.000778188 | 2.284489813 |
| hsa-circRNA2528-11_CBC1 | 0.003055472 | 6.207721244 |
| hsa_circ_0051580_CBC1 | 0.008191891 | 2.011859328 |
| hsa_circ_0094606_CBC1 | 0.008688975 | 2.046024673 |
| hsa_circ_0048865_CBC1 | 0.045475123 | 2.652012165 |
| hsa_circ_0086741_CBC1 | 0.04611142 | 2.049862528 |
| hsa_circ_0090470_CBC1 | 0.014284565 | 2.008575482 |
| hsa_circ_0074098_CBC1 | 1.24692E-05 | 5.044960664 |
| hsa-circRNA7685-5_CBC1 | 0.009622255 | 2.435097844 |
| hsa_circ_0026187_CBC1 | 0.005854585 | 3.263148832 |
| hsa_circ_0098814_CBC1 | 0.005480377 | 2.784531146 |
| hsa_circ_0138739_CBC1 | 0.002159899 | 4.04219543 |
| hsa-circRNA10297-2_CBC1 | 0.00734748 | 4.158622127 |
| hsa_circ_0002969_CBC1 | 0.002141171 | 2.109554882 |
| hsa_circ_0058781_CBC1 | 5.9977E-05 | 3.174126003 |
| hsa_circ_0105306_CBC1 | 0.000881338 | 2.109079762 |
| hsa-circRNA1721-33_CBC1 | 0.01277139 | 2.230896495 |
| hsa_circ_0026200_CBC1 | 0.007718179 | 3.846757128 |
| hsa_circ_0086188_CBC1 | 0.009147802 | 2.710779678 |
| hsa_circ_0029515_CBC1 | 0.000273989 | 3.977716634 |
| hsa_circ_0110443_CBC1 | 0.006188928 | 2.170784716 |
| hsa_circ_0017787_CBC1 | 0.004268895 | 8.82500127 |
| hsa-circRNA970-5_CBC1 | 0.02928137 | 2.140096413 |
| hsa-circRNA12871-15_CBC1 | 0.024804161 | 2.993462902 |
| hsa_circ_0018684_CBC1 | 0.012521269 | 2.893280466 |
| hsa_circ_0036697_CBC1 | 0.000228077 | 5.443356003 |
| hsa_circ_0125928_CBC1 | 0.006177297 | 3.180861502 |
| hsa_circ_0078047_CBC1 | 0.013762841 | 2.133081971 |
| hsa_circ_0029187_CBC1 | 0.007984522 | 2.22144348 |
| hsa_circ_0029100_CBC1 | 0.002581778 | 2.442786546 |
| hsa_circ_0076096_CBC1 | 0.030953794 | 2.016179369 |
| hsa_circ_0033315_CBC1 | 0.023305396 | 2.175041939 |
| hsa_circ_0063794_CBC1 | 7.43128E-05 | 4.614421461 |
| hsa-circRNA15172-8_CBC1 | 0.001172834 | 2.007845112 |
| hsa_circ_0048610_CBC1 | 0.005078624 | 4.667654294 |
| hsa-circRNA8518-22_CBC1 | 0.001343755 | 2.960182106 |
| hsa_circ_0072453_CBC1 | 0.023755756 | 2.766341689 |
| hsa_circ_0048608_CBC1 | 0.005848881 | 2.057854732 |
| hsa-circRNA9957-23_CBC1 | 0.002052286 | 2.324114736 |
| hsa_circ_0138742_CBC1 | 0.008097994 | 2.959137606 |
| hsa-circRNA14934-7_CBC1 | 0.000981079 | 2.819727251 |
| hsa_circ_0004748_CBC1 | 0.000395566 | 2.051287419 |
| hsa_circ_0088951_CBC1 | 0.000862441 | 2.633742105 |
| hsa_circ_0119166_CBC1 | 0.030571982 | 2.239498328 |
| hsa_circ_0025035_CBC1 | 5.24644E-05 | 3.893884215 |
| hsa-circRNA12701-38_CBC1 | 0.004663705 | 2.350223156 |
| hsa_circ_0049219_CBC1 | 0.001573659 | 2.803173608 |
| hsa_circ_0069277_CBC1 | 0.001549801 | 10.24928632 |
| hsa_circ_0035993_CBC1 | 0.004923501 | 2.42448075 |
| hsa_circ_0013387_CBC1 | 0.000964249 | 2.016300683 |
| hsa_circ_0067399_CBC1 | 0.018750745 | 2.63677409 |
| hsa-circRNA6201-6_CBC1 | 0.00149197 | 10.06420664 |
| hsa_circ_0082838_CBC1 | 0.011194822 | 3.197326213 |
| hsa_circ_0111961_CBC1 | 0.001553356 | 5.751216209 |
| hsa-circRNA16133-12_CBC1 | 0.014929641 | 2.840906961 |
| hsa_circ_0104341_CBC1 | 0.020212527 | 2.580349941 |
| hsa_circ_0083776_CBC1 | 0.026960405 | 2.04485449 |
| hsa_circ_0053157_CBC1 | 0.008164076 | 3.391923463 |
| hsa-circRNA11411-29_CBC1 | 0.022710681 | 2.967821365 |
| hsa-circRNA14257-9_CBC1 | 0.005832324 | 2.091681674 |
| hsa_circ_0043661_CBC1 | 0.019378419 | 2.748984004 |
| hsa_circ_0005883_CBC1 | 0.025159257 | 2.258404288 |
| hsa_circ_0040995_CBC1 | 0.001239753 | 4.573350575 |
| hsa-circRNA6112-3_CBC1 | 0.022477499 | 5.441927276 |
| hsa_circ_0084223_CBC1 | 0.007069774 | 2.016012338 |
| hsa_circ_0063529_CBC1 | 0.002378016 | 2.048760216 |
| hsa_circ_0032094_CBC1 | 0.002730226 | 2.297638233 |
| hsa-circRNA7275-92_CBC1 | 0.00589046 | 2.485770849 |
| hsa-circRNA2520-35_CBC1 | 0.000102055 | 6.681939029 |
| hsa_circ_0073954_CBC1 | 5.19775E-05 | 2.257594468 |
| hsa_circ_0076019_CBC1 | 0.001357487 | 2.006538123 |
| hsa-circRNA1021-6_CBC1 | 0.006518757 | 10.23726206 |
| hsa_circ_0034277_CBC1 | 0.00345727 | 2.879728518 |
| hsa-circRNA11433-33_CBC1 | 0.012557624 | 2.197704771 |
| hsa-circRNA7859-26_CBC1 | 0.003179043 | 2.318381588 |
| hsa-circRNA5796-4_CBC1 | 0.002108807 | 5.480793077 |
| hsa_circ_0048979_CBC1 | 0.000133695 | 2.55584159 |
| hsa_circ_0025167_CBC1 | 0.00112307 | 3.668387033 |
| hsa_circ_0006084_CBC1 | 0.006106929 | 5.321018606 |
| hsa_circ_0029083_CBC1 | 0.015688819 | 2.097383679 |
| hsa-circRNA2050-1_CBC1 | 0.000302238 | 2.469392947 |
| hsa_circ_0065433_CBC1 | 0.025991091 | 2.18136292 |
| hsa_circ_0079866_CBC1 | 0.000361177 | 5.663165077 |
| hsa-circRNA10229-6_CBC1 | 0.020372615 | 2.142104547 |
| hsa-circRNA1795_CBC1 | 0.004355823 | 2.422150048 |
| hsa_circ_0011530_CBC1 | 0.00030561 | 2.667517765 |
| hsa-circRNA5535-15_CBC1 | 0.004968461 | 4.076390794 |
| hsa_circ_0043960_CBC1 | 0.001579467 | 3.93765265 |
| hsa_circ_0135135_CBC1 | 0.002152911 | 2.248652736 |
| hsa-circRNA12516-20_CBC1 | 0.01498374 | 2.379406229 |
| hsa_circ_0022811_CBC1 | 0.004493491 | 3.692616717 |
| hsa-circRNA1501-7_CBC1 | 0.021261335 | 2.414686502 |
| hsa-circRNA5894-18_CBC1 | 0.001399558 | 6.430758503 |
| hsa-circRNA7413-4_CBC1 | 0.002808882 | 2.202111942 |
| hsa_circ_0049225_CBC1 | 0.000567259 | 2.906217812 |
| hsa-circRNA11458-24_CBC1 | 0.000246795 | 5.421922304 |
| hsa_circ_0032936_CBC1 | 0.011931541 | 2.729089621 |
| hsa_circ_0103473_CBC1 | 0.000528239 | 2.930571976 |
| hsa-circRNA5028-32_CBC1 | 1.2663E-05 | 7.225298433 |
| hsa-circRNA385-7_CBC1 | 0.03238733 | 2.61573832 |
| hsa_circ_0002933_CBC1 | 0.04689174 | 2.353751059 |
| hsa-circRNA1994-16_CBC1 | 0.001791986 | 2.112687857 |
| hsa-circRNA6034-101_CBC1 | 0.000108411 | 3.642871613 |
| hsa-circRNA5566-5_CBC1 | 0.033484099 | 2.244609249 |
| hsa-circRNA5886-38_CBC1 | 0.002446943 | 2.30111685 |
| hsa_circ_0065350_CBC1 | 0.001614748 | 7.050127302 |
| hsa_circ_0042564_CBC1 | 0.000325775 | 4.219900903 |
| hsa-circRNA1988-1_CBC1 | 0.001295195 | 4.489087426 |
| hsa_circ_0084486_CBC1 | 0.001780966 | 5.097817994 |
| hsa_circ_0083163_CBC1 | 0.02628035 | 2.137468838 |
| hsa_circ_0049110_CBC1 | 0.000375033 | 2.50342954 |
| hsa-circRNA13520-15_CBC1 | 0.007107229 | 3.18227456 |
| hsa_circ_0027011_CBC1 | 0.000238859 | 2.141511542 |
| hsa_circ_0006332_CBC1 | 3.24329E-05 | 4.834258957 |
| hsa_circ_0039192_CBC1 | 0.00027703 | 8.434184311 |
| hsa_circ_0018250_CBC1 | 0.011212928 | 2.523393059 |
| hsa-circRNA5897-51_CBC1 | 0.000874036 | 2.052023995 |
| hsa-circRNA10149-37_CBC1 | 0.000291773 | 4.217997597 |
| hsa_circ_0084475_CBC1 | 0.008508505 | 2.753421392 |
| hsa_circ_0067289_CBC1 | 0.025281085 | 2.368066738 |
| hsa-circRNA14716-17_CBC1 | 0.048519716 | 3.344296914 |
| hsa_circ_0006391_CBC1 | 0.020708155 | 2.603807122 |
| hsa-circRNA11966-36_CBC1 | 0.001347027 | 2.726697569 |
| hsa_circ_0029368_CBC1 | 0.015944025 | 2.024442457 |
| hsa-circRNA7970-25_CBC1 | 0.010552761 | 2.206750981 |
| hsa_circ_0026969_CBC1 | 0.016942624 | 3.773777434 |
| hsa_circ_0068856_CBC1 | 0.0004753 | 8.196030119 |
| hsa_circ_0086746_CBC1 | 0.000206582 | 7.01630008 |
| hsa-circRNA5333-3_CBC1 | 0.000138594 | 4.840322943 |
| hsa_circ_0098769_CBC1 | 0.003499097 | 3.021541658 |
| hsa_circ_0108843_CBC1 | 0.012178118 | 2.102520548 |
| hsa_circ_0074304_CBC1 | 0.030406937 | 3.61413497 |
| hsa_circ_0046136_CBC1 | 0.0002508 | 2.203369247 |
| hsa_circ_0009098_CBC1 | 5.06708E-05 | 7.055775165 |
| hsa_circ_0076727_CBC1 | 0.004601515 | 2.24060613 |
| hsa_circ_0096242_CBC1 | 0.012266735 | 2.166288993 |
| hsa_circ_0074088_CBC1 | 0.000255402 | 2.133835823 |
| hsa_circ_0094368_CBC1 | 0.024013822 | 2.314256126 |
| hsa_circ_0088928_CBC1 | 0.008175618 | 2.804703019 |
| hsa-circRNA7275-65_CBC1 | 0.013648105 | 2.157713838 |
| hsa_circ_0005947_CBC1 | 0.01727968 | 2.909174293 |
| hsa_circ_0068895_CBC1 | 9.37762E-05 | 2.08975471 |
| hsa_circ_0065445_CBC1 | 0.000907598 | 2.184177692 |
| hsa-circRNA5535-11_CBC1 | 0.004855579 | 4.1045149 |
| hsa_circ_0110932_CBC1 | 0.03547182 | 2.998052247 |
| hsa-circRNA14781-17_CBC1 | 0.012049005 | 2.215021177 |
| hsa_circ_0029085_CBC1 | 0.000219418 | 2.633503526 |
| hsa_circ_0041125_CBC1 | 0.031982114 | 3.16234034 |
| hsa_circ_0041023_CBC1 | 0.000658795 | 7.222989077 |
| hsa_circ_0097061_CBC1 | 0.024323649 | 2.964753762 |
| hsa-circRNA15598-15_CBC1 | 0.006320008 | 6.417301419 |
| hsa_circ_0117985_CBC1 | 0.008679125 | 9.079157677 |
| hsa_circ_0121970_CBC1 | 0.001118196 | 3.349744746 |
| hsa_circ_0107909_CBC1 | 0.004349689 | 2.10162778 |
| hsa_circ_0039710_CBC1 | 0.038908854 | 2.004797451 |
| hsa_circ_0014750_CBC1 | 0.000228047 | 7.600909627 |
| hsa_circ_0028991_CBC1 | 0.001675817 | 2.531096482 |
| hsa_circ_0048614_CBC1 | 0.001506715 | 2.4319668 |
| hsa_circ_0063030_CBC1 | 0.000489595 | 4.22189818 |
| hsa_circ_0004522_CBC1 | 0.008136297 | 2.663319752 |
| hsa_circ_0133416_CBC1 | 0.003862717 | 2.82589713 |
| hsa_circ_0080550_CBC1 | 0.003205006 | 2.655741521 |
| hsa_circ_0031781_CBC1 | 0.002176194 | 4.116716804 |
| hsa-circRNA3588-6_CBC1 | 0.011144354 | 2.276647312 |
| hsa-circRNA2719-45_CBC1 | 0.000817196 | 4.406951961 |
| hsa_circ_0039813_CBC1 | 5.88478E-05 | 4.284843531 |
| hsa_circ_0065038_CBC1 | 0.022798465 | 2.577616757 |
| hsa-circRNA14844-15_CBC1 | 0.008632717 | 3.523646072 |
| hsa-circRNA10541-2_CBC1 | 0.001325092 | 4.071158397 |
| hsa-circRNA2313-25_CBC1 | 0.021430352 | 2.179552895 |
| hsa_circ_0044027_CBC1 | 0.009174216 | 3.019680202 |
| hsa_circ_0021704_CBC1 | 0.004575134 | 2.207755104 |
| hsa_circ_0064243_CBC1 | 0.000573825 | 7.588725012 |
| hsa_circ_0098257_CBC1 | 0.019707052 | 2.162931023 |
| hsa-circRNA12483-3_CBC1 | 0.006610949 | 2.566665776 |
| hsa_circ_0088943_CBC1 | 0.005849118 | 2.06194094 |
| hsa_circ_0021539_CBC1 | 0.001315962 | 4.707247131 |
| hsa_circ_0084644_CBC1 | 0.036222757 | 2.463783175 |
| hsa_circ_0128479_CBC1 | 0.012347171 | 3.056458594 |
| hsa_circ_0102361_CBC1 | 0.000722223 | 3.48157236 |
| hsa_circ_0006402_CBC1 | 0.018392816 | 2.314937097 |
| hsa_circ_0028636_CBC1 | 0.002249939 | 2.655290376 |
| hsa_circ_0091228_CBC1 | 0.034153099 | 2.504096715 |
| hsa_circ_0082841_CBC1 | 0.00179402 | 2.115237942 |
| hsa-circRNA1951-1_CBC1 | 0.012500439 | 2.114806756 |
| hsa_circ_0043580_CBC1 | 0.01091334 | 2.283379244 |
| hsa_circ_0021703_CBC1 | 0.003076185 | 2.562567278 |
| hsa_circ_0043531_CBC1 | 0.000204292 | 5.271092409 |
| hsa_circ_0073745_CBC1 | 0.007189407 | 2.39987521 |
| hsa_circ_0082846_CBC1 | 0.000546017 | 2.782393186 |
| hsa_circ_0082847_CBC1 | 0.002394073 | 2.380186666 |
| hsa_circ_0040491_CBC1 | 0.002012836 | 2.668225093 |
| hsa_circ_0140512_CBC1 | 0.024105119 | 2.213367115 |
| hsa_circ_0059324_CBC1 | 0.003071508 | 2.023527928 |
| hsa-circRNA10206-15_CBC1 | 0.000653102 | 4.32450298 |
| hsa_circ_0129996_CBC1 | 8.80272E-05 | 6.588708002 |
| hsa_circ_0084726_CBC1 | 0.027441812 | 2.123061994 |
| hsa_circ_0131601_CBC1 | 0.002137912 | 2.281567334 |
| hsa-circRNA1594-12_CBC1 | 0.015146183 | 2.459629049 |
| hsa_circ_0005764_CBC1 | 0.000451772 | 3.830363044 |
| hsa_circ_0133419_CBC1 | 0.006635518 | 3.27545632 |
| hsa-circRNA858-19_CBC1 | 0.048536464 | 5.213723152 |
| hsa_circ_0060553_CBC1 | 0.001113813 | 3.831582266 |
| hsa_circ_0036738_CBC1 | 4.95081E-05 | 2.939853335 |
| hsa_circ_0033143_CBC1 | 0.030971766 | 2.002443194 |
| hsa_circ_0007356_CBC1 | 0.003560755 | 4.546092541 |
| hsa_circ_0009803_CBC1 | 0.008041752 | 2.153367057 |
| hsa_circ_0041016_CBC1 | 0.01469886 | 2.274889395 |
| hsa-circRNA15902-4_CBC1 | 0.003965343 | 2.21988111 |
| hsa_circ_0026182_CBC1 | 0.000883721 | 5.306728022 |
| hsa_circ_0091700_CBC1 | 0.003669283 | 4.103760281 |
| hsa-circRNA882-1_CBC1 | 0.000687849 | 3.397468651 |
| hsa-circRNA4049-22_CBC1 | 0.003457702 | 7.767370718 |
| hsa_circ_0024902_CBC1 | 0.000384304 | 3.33146336 |
| hsa_circ_0097918_CBC1 | 0.001437528 | 3.219366409 |
| hsa_circ_0038232_CBC1 | 0.0034559 | 2.022374216 |
| hsa_circ_0065979_CBC1 | 0.00346962 | 4.436091258 |
| hsa_circ_0061638_CBC1 | 0.001505624 | 2.829344455 |
| hsa_circ_0050693_CBC1 | 6.38025E-05 | 6.128031033 |
| hsa-circRNA2866-239_CBC1 | 0.001103666 | 3.047217505 |
| hsa-circRNA6451-2_CBC1 | 1.67119E-05 | 10.88845822 |
| hsa-circRNA9443-13_CBC1 | 0.009157163 | 3.313616483 |
| hsa-circRNA12453-6_CBC1 | 0.009321533 | 2.595506181 |
| hsa_circ_0029117_CBC1 | 0.005155771 | 2.300309111 |
| hsa_circ_0049122_CBC1 | 0.022866918 | 2.360403318 |
| hsa_circ_0137510_CBC1 | 0.001273322 | 3.524081696 |
| hsa_circ_0071681_CBC1 | 0.00018219 | 3.567855857 |
| hsa_circ_0036054_CBC1 | 0.00548143 | 2.343757953 |
| hsa_circ_0016378_CBC1 | 0.002009694 | 6.451027588 |
| hsa_circ_0022805_CBC1 | 0.004022529 | 3.40153406 |
| hsa_circ_0064209_CBC1 | 0.000120638 | 4.31462363 |
| hsa_circ_0041002_CBC1 | 0.000101579 | 4.40947141 |
| hsa-circRNA8657-21_CBC1 | 0.008729708 | 2.232821034 |
| hsa_circ_0107534_CBC1 | 6.7079E-05 | 2.50689634 |
| hsa_circ_0099153_CBC1 | 0.041892524 | 3.136136961 |
| hsa-circRNA15038-4_CBC1 | 0.003059547 | 2.89735142 |
| hsa_circ_0049609_CBC1 | 9.22909E-05 | 3.467050349 |
| hsa_circ_0084646_CBC1 | 0.005488114 | 3.284507435 |
| hsa-circRNA4361-1_CBC1 | 0.001092122 | 6.132153415 |
| hsa_circ_0106537_CBC1 | 0.030482358 | 3.3010945 |
| hsa_circ_0088889_CBC1 | 0.000348966 | 2.590156258 |
| hsa_circ_0025721_CBC1 | 0.006939942 | 2.117821485 |
| hsa_circ_0087511_CBC1 | 8.94821E-06 | 5.819086894 |
| hsa_circ_0091592_CBC1 | 0.000483198 | 2.761173749 |
| hsa_circ_0046536_CBC1 | 0.023802806 | 2.179541072 |
| hsa-circRNA2536-3_CBC1 | 0.000173151 | 6.597397758 |
| hsa-circRNA12077-7_CBC1 | 0.000279969 | 2.711042152 |
| hsa_circ_0115533_CBC1 | 0.033248501 | 2.094046119 |
| hsa_circ_0018986_CBC1 | 0.000166891 | 2.281324017 |
| hsa-circRNA4225-17_CBC1 | 0.000232153 | 3.61492286 |
| hsa_circ_0084275_CBC1 | 0.00139386 | 2.271994788 |
| hsa-circRNA11242-62_CBC1 | 8.83045E-05 | 6.483295103 |
| hsa_circ_0056380_CBC1 | 0.005110054 | 2.020563182 |
| hsa-circRNA2520-8_CBC1 | 2.9697E-05 | 16.65465944 |
| hsa_circ_0060551_CBC1 | 0.000128214 | 3.096108261 |
| hsa-circRNA15727-8_CBC1 | 0.002064246 | 6.889254931 |
| hsa_circ_0135660_CBC1 | 0.038123033 | 2.422491151 |
| hsa-circRNA15835-2_CBC1 | 0.000359102 | 2.088777496 |
| hsa_circ_0049208_CBC1 | 0.001691746 | 2.15601131 |
| hsa_circ_0022812_CBC1 | 0.002102983 | 3.503693566 |
| hsa_circ_0026190_CBC1 | 0.006727915 | 2.921223628 |
| hsa_circ_0065037_CBC1 | 0.02585649 | 3.457867788 |
| hsa-circRNA11595-41_CBC1 | 0.005997039 | 3.739118219 |
| hsa_circ_0065980_CBC1 | 0.002125454 | 3.440793626 |
| hsa_circ_0117014_CBC1 | 8.45879E-06 | 5.463817941 |
| hsa_circ_0063041_CBC1 | 5.06936E-05 | 3.688385055 |
| hsa_circ_0081406_CBC1 | 0.00042291 | 3.782665397 |
| hsa-circRNA1030-17_CBC1 | 0.000744177 | 7.30232474 |
| hsa-circRNA1790-11_CBC1 | 0.018384902 | 2.158686251 |
| hsa_circ_0063162_CBC1 | 0.000291132 | 2.775058557 |
| hsa-circRNA1203-3_CBC1 | 0.004403843 | 5.110786083 |
| hsa_circ_0018793_CBC1 | 0.00665123 | 2.14278307 |
| hsa_circ_0071992_CBC1 | 0.002883249 | 2.078389767 |
| hsa_circ_0103427_CBC1 | 5.55859E-05 | 6.2908518 |
| hsa_circ_0010016_CBC1 | 0.03243749 | 3.419664684 |
| hsa_circ_0012365_CBC1 | 0.000860648 | 4.326262363 |
| hsa_circ_0056033_CBC1 | 0.002297189 | 3.029935325 |
| hsa-circRNA8791-22_CBC1 | 2.50959E-05 | 6.582365439 |
| hsa-circRNA1496-2_CBC1 | 0.000806534 | 2.022117047 |
| hsa-circRNA5897-47_CBC1 | 0.000420996 | 2.248795239 |
| hsa_circ_0023844_CBC1 | 0.012550346 | 2.027221965 |
| hsa_circ_0107646_CBC1 | 0.000286908 | 4.055507194 |
| hsa_circ_0040885_CBC1 | 0.002624037 | 3.400697568 |
| hsa_circ_0012249_CBC1 | 0.004546152 | 3.145887673 |
| hsa_circ_0070659_CBC1 | 0.022610583 | 2.312077373 |
| hsa-circRNA10221-3_CBC1 | 0.000287656 | 3.812566424 |
| hsa_circ_0076154_CBC1 | 0.00335699 | 2.305930646 |
| hsa-circRNA6968-3_CBC1 | 0.000921057 | 3.669374004 |
| hsa_circ_0105072_CBC1 | 0.000497415 | 2.250676301 |
| hsa-circRNA8518-24_CBC1 | 0.003133563 | 3.688909614 |
| hsa_circ_0041858_CBC1 | 0.000224708 | 2.2769232 |
| hsa-circRNA6415-26_CBC1 | 0.040502698 | 2.352690402 |
| hsa-circRNA8791-33_CBC1 | 0.003227156 | 2.46089831 |
| hsa-circRNA2965-2_CBC1 | 0.000389091 | 2.100256105 |
| hsa_circ_0088935_CBC1 | 6.39138E-05 | 2.241017761 |
| hsa_circ_0096375_CBC1 | 0.005641769 | 2.68284471 |
| hsa-circRNA2719-25_CBC1 | 0.002754401 | 3.932332925 |
| hsa_circ_0087188_CBC1 | 0.000757649 | 2.243090033 |
| hsa-circRNA4049-35_CBC1 | 0.000602931 | 4.019484839 |
| hsa_circ_0044030_CBC1 | 0.001230594 | 4.291184799 |
| hsa_circ_0058988_CBC1 | 0.001997648 | 2.812461214 |
| hsa-circRNA1772-5_CBC1 | 0.000624722 | 2.586911587 |
| hsa_circ_0120430_CBC1 | 0.044226101 | 2.424167436 |
| hsa_circ_0049402_CBC1 | 2.41648E-05 | 8.243281956 |
| hsa_circ_0005837_CBC1 | 0.017036698 | 8.326254379 |
| hsa_circ_0098807_CBC1 | 0.022378875 | 2.073337429 |
| hsa-circRNA617-5_CBC1 | 0.000233344 | 3.429387198 |
| hsa_circ_0072044_CBC1 | 7.67735E-05 | 2.816115133 |
| hsa_circ_0012091_CBC1 | 0.018140457 | 2.151350232 |
| hsa-circRNA11433-27_CBC1 | 0.010620419 | 2.179295946 |
| hsa_circ_0053019_CBC1 | 0.011380656 | 4.197253861 |
| hsa_circ_0028434_CBC1 | 0.002666597 | 2.934902491 |
| hsa_circ_0028125_CBC1 | 0.023845095 | 2.750279754 |
| hsa-circRNA13990-15_CBC1 | 0.012474709 | 3.066003236 |
| hsa-circRNA5383-4_CBC1 | 0.000131379 | 9.932650308 |
| hsa_circ_0024912_CBC1 | 0.001121961 | 4.385552631 |
| hsa_circ_0064202_CBC1 | 0.00245798 | 3.937563115 |
| hsa_circ_0122736_CBC1 | 0.006936865 | 3.892896035 |
| hsa_circ_0041860_CBC1 | 0.00276094 | 2.071552613 |
| hsa_circ_0094256_CBC1 | 0.00115128 | 2.025265386 |
| hsa_circ_0041000_CBC1 | 0.008591334 | 6.723784658 |
| hsa_circ_0056717_CBC1 | 0.038407687 | 2.105810901 |
| hsa-circRNA12292-13_CBC1 | 0.001097038 | 2.182957755 |
| hsa_circ_0082202_CBC1 | 0.002426524 | 2.191768865 |
| hsa-circRNA2328-5_CBC1 | 0.006927878 | 3.168599396 |
| hsa_circ_0125039_CBC1 | 0.012036089 | 3.032974492 |
| hsa-circRNA2407-8_CBC1 | 0.002771696 | 2.146766936 |
| hsa_circ_0039093_CBC1 | 0.00038123 | 2.003794471 |
| hsa_circ_0039587_CBC1 | 0.005733279 | 2.275229444 |
| hsa_circ_0087267_CBC1 | 0.020015477 | 2.559676541 |
| hsa_circ_0034602_CBC1 | 0.002691414 | 5.940478481 |
| hsa-circRNA1257-37_CBC1 | 0.000256462 | 3.677616682 |
| hsa_circ_0032827_CBC1 | 0.018554412 | 2.765460842 |
| hsa_circ_0015975_CBC1 | 0.012592949 | 2.708759182 |
| hsa_circ_0015824_CBC1 | 0.002535921 | 8.650742457 |
| hsa_circ_0057226_CBC1 | 0.002991383 | 2.209837221 |
| hsa_circ_0025033_CBC1 | 0.003039238 | 9.174205996 |
| hsa_circ_0029131_CBC1 | 0.012785752 | 2.197445389 |
| hsa_circ_0043863_CBC1 | 0.001101229 | 2.690082917 |
| hsa-circRNA9443-22_CBC1 | 0.013660732 | 2.934305205 |
| hsa-circRNA1994-11_CBC1 | 0.003300367 | 3.061103188 |
| hsa-circRNA10677-3_CBC1 | 0.000203384 | 2.08062948 |
| hsa_circ_0064232_CBC1 | 0.000279742 | 3.996497275 |
| hsa-circRNA15649-3_CBC1 | 0.007260063 | 2.091286303 |
| hsa-circRNA10893-19_CBC1 | 0.000147026 | 4.673667769 |
| hsa-circRNA1081-4_CBC1 | 0.025879494 | 2.400913772 |
| hsa_circ_0074935_CBC1 | 0.006921664 | 2.054842239 |
| hsa_circ_0030401_CBC1 | 0.000432197 | 4.09016255 |
| hsa_circ_0090947_CBC1 | 0.00333693 | 7.728191605 |
| hsa-circRNA2719-40_CBC1 | 0.001515938 | 4.015211135 |
| hsa_circ_0047935_CBC1 | 0.017712403 | 3.397859716 |
| hsa-circRNA2328-8_CBC1 | 0.005792548 | 4.339270793 |
| hsa_circ_0081416_CBC1 | 0.001676655 | 2.000154927 |
| hsa-circRNA1231-1_CBC1 | 0.000792293 | 2.190225339 |
| hsa-circRNA3557-12_CBC1 | 0.007380278 | 2.431798493 |
| hsa_circ_0075856_CBC1 | 0.033365093 | 2.440170303 |
| hsa-circRNA2097-6_CBC1 | 0.004509888 | 3.080234202 |
| hsa_circ_0043544_CBC1 | 0.008433836 | 2.467708576 |
| hsa_circ_0061628_CBC1 | 0.005007555 | 3.477497954 |
| hsa-circRNA13165-21_CBC1 | 0.042282738 | 2.32990563 |
| hsa_circ_0056032_CBC1 | 0.001155909 | 7.992985016 |
| hsa_circ_0105637_CBC1 | 9.88944E-05 | 2.082656996 |
| hsa_circ_0097643_CBC1 | 0.000678017 | 2.204307889 |
| hsa_circ_0065354_CBC1 | 0.000990332 | 3.365132579 |
| hsa_circ_0029532_CBC1 | 0.00114406 | 2.263320974 |
| hsa_circ_0026586_CBC1 | 0.002563454 | 3.045161472 |
| hsa_circ_0081405_CBC1 | 0.00037674 | 3.497692068 |
| hsa_circ_0050715_CBC1 | 0.000214834 | 4.693802884 |
| hsa_circ_0052998_CBC1 | 0.001505822 | 4.723659126 |
| hsa_circ_0066945_CBC1 | 0.004928595 | 6.878359139 |
| hsa_circ_0099946_CBC1 | 0.000375091 | 2.645792077 |
| hsa_circ_0082519_CBC1 | 0.017565345 | 3.071490242 |
| hsa_circ_0018473_CBC1 | 0.000667662 | 2.966181243 |
| hsa-circRNA5028-27_CBC1 | 0.000143613 | 5.061083167 |
| hsa_circ_0028600_CBC1 | 0.000359028 | 3.924135704 |
| hsa_circ_0089600_CBC1 | 0.024796425 | 2.253391997 |
| hsa-circRNA2995-10_CBC1 | 0.010264034 | 2.148847794 |
| hsa_circ_0025158_CBC1 | 0.000478467 | 3.506506688 |
| hsa_circ_0111663_CBC1 | 0.000420883 | 9.284068061 |
| hsa_circ_0043149_CBC1 | 0.005867066 | 2.160414942 |
| hsa-circRNA8518-40_CBC1 | 0.003222623 | 2.066266555 |
| hsa_circ_0083736_CBC1 | 0.000365594 | 4.90651743 |
| hsa_circ_0135000_CBC1 | 0.046627614 | 2.107728268 |
| hsa-circRNA427-3_CBC1 | 0.006860902 | 3.527883484 |
| hsa_circ_0108835_CBC1 | 0.001116359 | 3.475533914 |
| hsa_circ_0105951_CBC1 | 0.00073558 | 3.001745109 |
| hsa_circ_0061630_CBC1 | 0.003272363 | 3.240535908 |
| hsa-circRNA10206-9_CBC1 | 2.51372E-06 | 3.333033315 |
| hsa-circRNA13520-4_CBC1 | 0.000151663 | 4.488934583 |
| hsa-circRNA990-2_CBC1 | 0.000447392 | 3.089604679 |
| hsa_circ_0032820_CBC1 | 0.000405311 | 4.902593717 |
| hsa_circ_0043867_CBC1 | 0.003421759 | 2.122348109 |
| hsa_circ_0047143_CBC1 | 0.013328962 | 2.32222985 |
| hsa_circ_0039741_CBC1 | 0.014522014 | 2.584150448 |
| hsa_circ_0063178_CBC1 | 0.000798477 | 2.115439113 |
| hsa_circ_0048431_CBC1 | 0.001021363 | 2.077051544 |
| hsa-circRNA8511-16_CBC1 | 0.018879031 | 5.477356288 |
| hsa-circRNA2407-12_CBC1 | 0.019632271 | 2.79074343 |
| hsa_circ_0034614_CBC1 | 0.000493233 | 6.768722814 |
| hsa_circ_0074693_CBC1 | 0.038809283 | 2.091023561 |
| hsa_circ_0057548_CBC1 | 0.00408574 | 2.865934546 |
| hsa_circ_0064007_CBC1 | 0.000378646 | 2.27423648 |
| hsa-circRNA5149-25_CBC1 | 0.00127644 | 3.337150642 |
| hsa-circRNA4165_CBC1 | 0.000241688 | 2.248508185 |
| hsa-circRNA12268-4_CBC1 | 0.004064952 | 2.030539019 |
| hsa_circ_0025171_CBC1 | 0.000428933 | 3.190072318 |
| hsa-circRNA5544-10_CBC1 | 0.046078457 | 2.479493917 |
| hsa-circRNA11372-5_CBC1 | 0.007594245 | 2.034389098 |
| hsa_circ_0088992_CBC1 | 0.000336799 | 2.758383466 |
| hsa-circRNA3215-22_CBC1 | 7.68755E-05 | 2.072088454 |
| hsa-circRNA13520-12_CBC1 | 0.021619012 | 2.818344293 |
| hsa_circ_0046522_CBC1 | 0.007202335 | 2.086879475 |
| hsa_circ_0041865_CBC1 | 0.000448168 | 2.068217954 |
| hsa_circ_0044127_CBC1 | 5.58872E-05 | 10.82596578 |
| hsa-circRNA1030-5_CBC1 | 0.000443886 | 6.819129001 |
| hsa_circ_0065032_CBC1 | 0.011749051 | 4.49493094 |
| hsa_circ_0026183_CBC1 | 8.13796E-06 | 13.00866644 |
| hsa_circ_0064253_CBC1 | 9.94309E-06 | 4.828192562 |
| hsa_circ_0103472_CBC1 | 0.003911434 | 3.13872069 |
| hsa-circRNA11981-13_CBC1 | 0.002241836 | 3.61858735 |
| hsa_circ_0043500_CBC1 | 0.01073144 | 2.042335585 |
| hsa_circ_0122731_CBC1 | 0.003605596 | 2.78949448 |
| hsa-circRNA13111-2_CBC1 | 0.002738503 | 3.036184447 |
| hsa_circ_0040577_CBC1 | 0.000353631 | 2.62557106 |
| hsa_circ_0059881_CBC1 | 0.002498196 | 2.197592009 |
| hsa-circRNA11242-10_CBC1 | 0.000511995 | 5.41108186 |
| hsa_circ_0090955_CBC1 | 0.000604248 | 10.95962885 |
| hsa_circ_0065760_CBC1 | 6.61212E-06 | 6.45798363 |
| hsa-circRNA8433-15_CBC1 | 0.00150962 | 10.43247777 |
| hsa_circ_0082513_CBC1 | 0.012726787 | 2.099497476 |
| hsa_circ_0065448_CBC1 | 0.003048691 | 2.30183204 |
| hsa_circ_0065438_CBC1 | 0.001648598 | 2.78345019 |
| hsa-circRNA12510-2_CBC1 | 0.020967075 | 2.579915231 |
| hsa-circRNA13178-6_CBC1 | 0.002102233 | 2.251064118 |
| hsa_circ_0124810_CBC1 | 0.005269832 | 3.184692225 |
| hsa-circRNA9491-5_CBC1 | 0.003477529 | 2.181940466 |
| hsa-circRNA6968-2_CBC1 | 0.000324586 | 2.973254143 |
| hsa_circ_0049216_CBC1 | 0.000331236 | 2.832569433 |
| hsa-circRNA2719-43_CBC1 | 4.88127E-05 | 4.420048685 |
| hsa_circ_0094485_CBC1 | 0.00027317 | 8.053835018 |
| hsa-circRNA13865-2_CBC1 | 0.045360659 | 2.102591582 |
| hsa_circ_0060837_CBC1 | 0.003188666 | 5.656468659 |
| hsa_circ_0038634_CBC1 | 2.09429E-05 | 12.1343877 |
| hsa_circ_0077402_CBC1 | 0.043550308 | 2.557112257 |
| hsa_circ_0077157_CBC1 | 0.000345967 | 7.63433218 |
| hsa-circRNA7445-27_CBC1 | 0.028706512 | 2.9074435 |
| hsa_circ_0065759_CBC1 | 2.66076E-05 | 6.193627287 |
| hsa_circ_0113636_CBC1 | 0.004105637 | 3.431514398 |
| hsa_circ_0068027_CBC1 | 0.015137169 | 3.203876059 |
| hsa_circ_0138679_CBC1 | 0.002361897 | 5.041333096 |
| hsa-circRNA7836-96_CBC1 | 2.39252E-05 | 2.533978768 |
| hsa_circ_0096559_CBC1 | 0.002382697 | 3.706871359 |
| hsa-circRNA5886-27_CBC1 | 6.52175E-05 | 2.601816987 |
| hsa_circ_0026742_CBC1 | 0.00739479 | 2.086958078 |
| hsa-circRNA7663-7_CBC1 | 0.001425895 | 2.457082473 |
| hsa_circ_0059725_CBC1 | 3.30342E-05 | 6.758732339 |
| hsa-circRNA5806-26_CBC1 | 0.020721397 | 2.824853856 |
| hsa-circRNA8900-5_CBC1 | 0.001413262 | 3.95242545 |
| hsa-circRNA15486-289_CBC1 | 0.003041442 | 2.555169206 |
| hsa_circ_0012293_CBC1 | 6.53755E-05 | 2.743354331 |
| hsa_circ_0063410_CBC1 | 0.009482062 | 2.268495929 |
| hsa_circ_0025722_CBC1 | 0.02967963 | 2.471032578 |
| hsa_circ_0014229_CBC1 | 0.004890824 | 2.480203162 |
| hsa_circ_0005103_CBC1 | 0.009226219 | 2.248484866 |
| hsa_circ_0029095_CBC1 | 0.007541919 | 4.812058934 |
| hsa-circRNA2520-23_CBC1 | 0.001213313 | 4.394083132 |
| hsa-circRNA3238-2_CBC1 | 0.028569384 | 2.051625588 |
| hsa_circ_0007703_CBC1 | 0.000998693 | 2.218115641 |
| hsa_circ_0057345_CBC1 | 0.009400614 | 2.632831707 |
| hsa_circ_0094591_CBC1 | 0.007190509 | 4.579776453 |
| hsa_circ_0080637_CBC1 | 0.005622352 | 2.148339863 |
| hsa-circRNA11458-6_CBC1 | 0.000295813 | 4.70846596 |
| hsa_circ_0035489_CBC1 | 1.44127E-05 | 6.712305885 |
| hsa_circ_0097466_CBC1 | 0.005081877 | 3.216090679 |
| hsa-circRNA3897-10_CBC1 | 0.000260342 | 3.421231282 |
| hsa_circ_0002662_CBC1 | 0.004295108 | 2.065824899 |
| hsa_circ_0088802_CBC1 | 0.002678692 | 2.183931528 |
| hsa_circ_0078046_CBC1 | 0.042422228 | 2.623682016 |
| hsa_circ_0095649_CBC1 | 0.007326636 | 2.599453516 |
| hsa-circRNA10312-8_CBC1 | 0.013919986 | 2.310298238 |
| hsa-circRNA7413-12_CBC1 | 0.002414666 | 2.68151231 |
| hsa_circ_0048361_CBC1 | 0.005092016 | 2.167379339 |
| hsa_circ_0064566_CBC1 | 0.000941697 | 9.982476432 |
| hsa_circ_0138114_CBC1 | 0.00048137 | 2.770106722 |
| hsa_circ_0029191_CBC1 | 0.012406994 | 2.145892148 |
| hsa-circRNA11242-20_CBC1 | 0.006000752 | 4.426739118 |
| hsa_circ_0016240_CBC1 | 0.021774933 | 2.020185644 |
| hsa_circ_0017745_CBC1 | 0.000273819 | 2.233591888 |
| hsa_circ_0126895_CBC1 | 0.039539983 | 2.74205957 |
| hsa_circ_0043172_CBC1 | 0.010980108 | 2.346910271 |
| hsa_circ_0119604_CBC1 | 0.011306182 | 2.281771988 |
| hsa-circRNA9929-18_CBC1 | 0.013846087 | 2.408478213 |
| hsa-circRNA11615-3_CBC1 | 0.002321964 | 2.671083448 |
| hsa-circRNA11180-35_CBC1 | 0.000679675 | 2.717192971 |
| hsa_circ_0040741_CBC1 | 0.00017166 | 5.561515009 |
| hsa_circ_0051653_CBC1 | 0.001867668 | 2.232912862 |
| hsa_circ_0062193_CBC1 | 0.049892094 | 2.320079555 |
| hsa_circ_0073746_CBC1 | 0.001469381 | 6.65265612 |
| hsa_circ_0021492_CBC1 | 0.000145293 | 2.031965175 |
| hsa_circ_0088308_CBC1 | 0.017115605 | 2.387296254 |
| hsa_circ_0049213_CBC1 | 0.000240248 | 3.28583003 |
| hsa_circ_0047923_CBC1 | 0.005816382 | 3.39116775 |
| hsa_circ_0084481_CBC1 | 0.022937273 | 2.704904397 |
| hsa_circ_0056040_CBC1 | 1.61904E-05 | 7.800324278 |
| hsa_circ_0136925_CBC1 | 0.03038701 | 2.184803491 |
| hsa_circ_0064229_CBC1 | 0.000984021 | 3.364518678 |
| hsa_circ_0009100_CBC1 | 3.07439E-05 | 2.953611312 |
| hsa_circ_0007405_CBC1 | 0.003590961 | 2.780128293 |
| hsa_circ_0017790_CBC1 | 0.003287344 | 7.058697077 |
| hsa_circ_0028632_CBC1 | 0.041363113 | 3.446065901 |
| hsa_circ_0089661_CBC1 | 2.41147E-05 | 3.507300591 |
| hsa-circRNA6297-9_CBC1 | 8.4725E-05 | 8.579174366 |
| hsa-circRNA10893-13_CBC1 | 0.000439723 | 3.89288135 |
| hsa_circ_0028603_CBC1 | 0.001693352 | 2.114375409 |
| hsa_circ_0002230_CBC1 | 0.028884246 | 2.052402991 |
| hsa-circRNA7602_CBC1 | 0.015576286 | 2.861686658 |
| hsa_circ_0048350_CBC1 | 0.009026173 | 2.457126944 |
| hsa_circ_0065036_CBC1 | 0.002346901 | 4.729797853 |
| hsa-circRNA10149-14_CBC1 | 7.05034E-05 | 4.652522755 |
| hsa_circ_0036928_CBC1 | 0.000453841 | 3.078592284 |
| hsa_circ_0083184_CBC1 | 0.001010338 | 4.785390902 |
| hsa_circ_0077158_CBC1 | 0.000650972 | 7.80877828 |
| hsa-circRNA11930-2_CBC1 | 0.000712502 | 3.985452329 |
| hsa_circ_0021538_CBC1 | 0.009433398 | 4.966617 |
| hsa-circRNA11458-30_CBC1 | 0.003926585 | 4.736142836 |
| hsa_circ_0139455_CBC1 | 0.00215731 | 7.557742433 |
| hsa_circ_0082515_CBC1 | 0.02284762 | 2.184733069 |
| hsa_circ_0042324_CBC1 | 0.01833936 | 2.082134905 |
| hsa_circ_0095737_CBC1 | 0.014957193 | 2.284520245 |
| hsa-circRNA3215-52_CBC1 | 0.000170969 | 2.092324563 |
| hsa-circRNA4065-29_CBC1 | 3.7115E-05 | 2.190143912 |
| hsa_circ_0086195_CBC1 | 0.000875437 | 2.044560116 |
| hsa_circ_0090954_CBC1 | 0.000570293 | 8.34160366 |
| hsa_circ_0049744_CBC1 | 0.001374968 | 2.989547334 |
| hsa-circRNA851-9_CBC1 | 0.00540999 | 2.017133768 |
| hsa_circ_0042565_CBC1 | 8.11343E-05 | 5.782879913 |
| hsa_circ_0021735_CBC1 | 0.00146028 | 2.195885316 |
| hsa-circRNA5383-6_CBC1 | 3.87023E-06 | 5.321217715 |
| hsa_circ_0072763_CBC1 | 9.52199E-05 | 7.865307055 |
| hsa-circRNA14844-6_CBC1 | 0.023955183 | 2.973353769 |
| hsa-circRNA2283-4_CBC1 | 0.006880777 | 2.060893565 |
| hsa_circ_0029526_CBC1 | 2.2347E-05 | 3.597002932 |
| hsa_circ_0007548_CBC1 | 0.000596986 | 2.405541055 |
| hsa_circ_0082510_CBC1 | 0.012966649 | 2.795940013 |
| hsa_circ_0024913_CBC1 | 0.002480572 | 2.145381739 |
| hsa_circ_0040742_CBC1 | 0.003732672 | 4.619906109 |
| hsa_circ_0088317_CBC1 | 0.003060185 | 2.130701851 |
| hsa-circRNA10541-25_CBC1 | 0.001659979 | 3.455954744 |
| hsa_circ_0021835_CBC1 | 0.010607361 | 2.104214015 |
| hsa_circ_0071654_CBC1 | 0.005066091 | 3.481270403 |
| hsa_circ_0011290_CBC1 | 0.003045083 | 2.758240038 |
| hsa_circ_0121228_CBC1 | 2.40824E-05 | 6.813447357 |
| hsa_circ_0039473_CBC1 | 0.002360603 | 2.032317414 |
| hsa_circ_0099645_CBC1 | 0.001415442 | 3.13855653 |
| hsa_circ_0034565_CBC1 | 8.81164E-05 | 5.902238812 |
| hsa_circ_0029486_CBC1 | 0.012218621 | 3.100667762 |
| hsa_circ_0064266_CBC1 | 0.011149967 | 2.381579166 |
| hsa_circ_0083899_CBC1 | 0.005672511 | 2.756937285 |
| hsa_circ_0006516_CBC1 | 0.000844923 | 2.031556219 |
| hsa_circ_0077800_CBC1 | 9.25211E-05 | 6.223060444 |
| hsa_circ_0020491_CBC1 | 3.13091E-05 | 15.43501414 |
| hsa-circRNA2520-31_CBC1 | 3.31261E-05 | 6.858659728 |
| hsa-circRNA13527-1_CBC1 | 0.001246237 | 3.162327982 |
| hsa-circRNA12269-7_CBC1 | 0.000201275 | 6.005929358 |
| hsa_circ_0092521_CBC1 | 0.034856459 | 2.621869388 |
| hsa_circ_0063045_CBC1 | 0.000202767 | 2.314755138 |
| hsa-circRNA11981-15_CBC1 | 0.000502209 | 3.045547432 |
| hsa-circRNA13850-11_CBC1 | 0.013894279 | 2.313252464 |
| hsa-circRNA4965-28_CBC1 | 0.000130558 | 2.623255994 |
| hsa_circ_0125969_CBC1 | 0.000132501 | 2.409449875 |
| hsa_circ_0042341_CBC1 | 8.41918E-05 | 5.830333827 |
| hsa_circ_0038929_CBC1 | 0.005233764 | 2.16624483 |
| hsa_circ_0048432_CBC1 | 0.010694979 | 2.175816256 |
| hsa-circRNA11993-4_CBC1 | 0.000171494 | 8.754265941 |
| hsa_circ_0041039_CBC1 | 0.000594861 | 5.17659592 |
| hsa_circ_0045478_CBC1 | 0.008952749 | 2.031214792 |
| hsa-circRNA13021-5_CBC1 | 0.001143557 | 3.681118886 |
| hsa_circ_0084361_CBC1 | 0.036799928 | 2.035427964 |
| hsa_circ_0113882_CBC1 | 0.004283625 | 2.962808388 |
| hsa-circRNA4049-10_CBC1 | 0.047108889 | 3.48034435 |
| hsa_circ_0001046_CBC1 | 1.83065E-05 | 5.90434385 |
| hsa_circ_0042792_CBC1 | 0.00472431 | 2.274892227 |
| hsa-circRNA626-11_CBC1 | 0.006818792 | 2.508392633 |
| hsa_circ_0049326_CBC1 | 0.004733945 | 2.06754519 |
| hsa-circRNA10819-1_CBC1 | 0.010938992 | 2.018329756 |
| hsa_circ_0082196_CBC1 | 0.000785312 | 2.133631306 |
| hsa-circRNA617-13_CBC1 | 0.028202758 | 2.90732623 |
| hsa_circ_0097163_CBC1 | 0.00390578 | 2.343230628 |
| hsa-circRNA9867-61_CBC1 | 0.02542806 | 2.713305017 |
| hsa_circ_0072005_CBC1 | 0.001738045 | 2.015284456 |
| hsa-circRNA10206-41_CBC1 | 4.44384E-06 | 4.174003492 |
| hsa_circ_0082505_CBC1 | 0.003503884 | 2.25318323 |
| hsa_circ_0009806_CBC1 | 0.000198424 | 2.007960404 |
| hsa_circ_0089002_CBC1 | 0.005965281 | 3.355852375 |
| hsa_circ_0095553_CBC1 | 0.001684994 | 2.487505313 |
| hsa_circ_0049092_CBC1 | 0.033912837 | 2.428604131 |
| hsa_circ_0049049_CBC1 | 0.021797676 | 2.341443067 |
| hsa_circ_0007726_CBC1 | 0.00232236 | 3.790313659 |
| hsa-circRNA8483-3_CBC1 | 0.000222179 | 2.361587866 |
| hsa_circ_0068858_CBC1 | 0.000344511 | 15.77445492 |
| hsa_circ_0085456_CBC1 | 0.023086956 | 2.864349349 |
| hsa_circ_0025259_CBC1 | 0.004699935 | 2.265353131 |
| hsa_circ_0045024_CBC1 | 0.000825827 | 2.697948591 |
| hsa_circ_0128451_CBC1 | 0.004077591 | 2.459123453 |
| hsa_circ_0110245_CBC1 | 0.045556018 | 3.046574312 |
| hsa_circ_0137507_CBC1 | 0.001369728 | 2.04692409 |
| hsa_circ_0051414_CBC1 | 0.001441756 | 2.075993197 |
| hsa_circ_0050711_CBC1 | 2.9969E-05 | 3.648148086 |
| hsa_circ_0065982_CBC1 | 0.001541836 | 3.849866543 |
| hsa-circRNA3390-9_CBC1 | 2.8235E-05 | 3.331513889 |
| hsa_circ_0084382_CBC1 | 0.009978137 | 2.19023394 |
| hsa_circ_0090645_CBC1 | 0.001471678 | 2.894486767 |
| hsa_circ_0088881_CBC1 | 0.000138918 | 5.406894651 |
| hsa-circRNA11242-47_CBC1 | 0.000286968 | 2.570633601 |
| hsa-circRNA2506-2_CBC1 | 0.000171769 | 4.845039545 |
| hsa-circRNA6265-1_CBC1 | 0.037179389 | 2.548366699 |
| hsa_circ_0000171_CBC1 | 0.002445057 | 10.89505417 |
| hsa_circ_0040003_CBC1 | 0.015805381 | 2.674388668 |
| hsa-circRNA5318-9_CBC1 | 0.011176148 | 2.038954929 |
| hsa-circRNA2407-9_CBC1 | 0.00262652 | 3.414914886 |
| hsa_circ_0039602_CBC1 | 0.005162751 | 2.11261741 |
| hsa-circRNA15229-7_CBC1 | 0.000565909 | 3.5847882 |
| hsa-circRNA10149-20_CBC1 | 1.80872E-05 | 7.53177012 |
| hsa-circRNA13715-4_CBC1 | 0.003198427 | 2.183491784 |
| hsa_circ_0080977_CBC1 | 0.001910367 | 2.649319646 |
| hsa-circRNA7663-24_CBC1 | 0.006855332 | 2.703117227 |
| hsa_circ_0082843_CBC1 | 0.004218699 | 2.889900487 |
| hsa_circ_0062270_CBC1 | 0.000439217 | 7.519208652 |
| hsa-circRNA10541-18_CBC1 | 0.002280029 | 3.123310403 |
| hsa-circRNA11772-1_CBC1 | 0.029726105 | 2.200714062 |
| hsa-circRNA2910-9_CBC1 | 0.000425988 | 10.04181734 |
| hsa_circ_0082811_CBC1 | 0.035059141 | 2.067752337 |
| hsa-circRNA7836-93_CBC1 | 0.001817607 | 2.079696534 |
| hsa_circ_0081551_CBC1 | 0.003325447 | 2.113369633 |
| hsa-circRNA2639-20_CBC1 | 0.00077742 | 2.338099389 |
| hsa-circRNA2313-11_CBC1 | 0.005453052 | 2.499052036 |
| hsa_circ_0088804_CBC1 | 0.000169057 | 2.088628964 |
| hsa_circ_0068867_CBC1 | 0.000845434 | 8.47861834 |
| hsa_circ_0106838_CBC1 | 0.032778989 | 2.052342692 |
| hsa_circ_0018037_CBC1 | 0.005030373 | 2.52819643 |
| hsa_circ_0038632_CBC1 | 0.000395829 | 8.103604435 |
| hsa_circ_0088967_CBC1 | 0.000466429 | 2.194178411 |
| hsa_circ_0029144_CBC1 | 0.023015607 | 2.286485132 |
| hsa_circ_0007935_CBC1 | 0.003536595 | 2.109620031 |
| hsa-circRNA12437-19_CBC1 | 0.005992085 | 2.038352483 |
| hsa_circ_0050041_CBC1 | 0.010051606 | 2.009440044 |
| hsa_circ_0079000_CBC1 | 4.42464E-05 | 5.001457712 |
| hsa_circ_0045603_CBC1 | 0.002886397 | 2.098129794 |
| hsa-circRNA15486-245_CBC1 | 0.041447391 | 2.165130591 |
| hsa-circRNA7257-5_CBC1 | 0.037819349 | 2.674819135 |
| hsa-circRNA13043-25_CBC1 | 0.023683229 | 3.931318408 |
| hsa-circRNA5287-1_CBC1 | 0.006156509 | 2.772987161 |
| hsa_circ_0097696_CBC1 | 0.022385405 | 2.058573724 |
| hsa_circ_0012601_CBC1 | 0.049072845 | 2.538535336 |
| hsa_circ_0127018_CBC1 | 0.030386029 | 2.080793882 |
| hsa-circRNA6411-18_CBC1 | 0.000999276 | 5.746625362 |
| hsa_circ_0029174_CBC1 | 0.002647538 | 2.003684073 |
| hsa_circ_0098264_CBC1 | 0.016125454 | 2.03121654 |
| hsa_circ_0091237_CBC1 | 0.005589977 | 4.822657961 |
| hsa_circ_0049950_CBC1 | 2.35429E-06 | 2.045638746 |
| hsa_circ_0123868_CBC1 | 0.022072648 | 4.017336531 |
| hsa_circ_0044578_CBC1 | 0.000100943 | 2.058232599 |
| hsa-circRNA8310-10_CBC1 | 0.000305021 | 2.196233309 |
| hsa_circ_0083900_CBC1 | 0.00198897 | 2.634459889 |
| hsa_circ_0018038_CBC1 | 0.002502143 | 2.098120031 |
| hsa-circRNA7543-1_CBC1 | 0.00199816 | 3.274286115 |
| hsa_circ_0073763_CBC1 | 0.027719279 | 2.170966304 |
| hsa-circRNA1926-4_CBC1 | 0.001754416 | 3.33582393 |
| hsa-circRNA5149-23_CBC1 | 0.002688934 | 3.794597582 |
| hsa_circ_0084987_CBC1 | 0.008672252 | 2.372114283 |
| hsa_circ_0025037_CBC1 | 2.49466E-05 | 3.983598505 |
| hsa-circRNA14781-10_CBC1 | 0.01872921 | 4.024430773 |
| hsa-circRNA13111-11_CBC1 | 0.000252032 | 4.242283663 |
| hsa_circ_0068019_CBC1 | 0.003005731 | 2.053834449 |
| hsa_circ_0051723_CBC1 | 0.002120749 | 6.717714942 |
| hsa_circ_0022813_CBC1 | 5.55832E-06 | 3.525217044 |
| hsa-circRNA4666-26_CBC1 | 0.004254726 | 3.366857605 |
| hsa_circ_0103444_CBC1 | 0.000845214 | 2.238411514 |
| hsa_circ_0029125_CBC1 | 0.01523122 | 2.465977518 |
| hsa_circ_0077220_CBC1 | 0.048054897 | 2.009695466 |
| hsa_circ_0038931_CBC1 | 0.006351181 | 5.306533352 |
| hsa_circ_0029524_CBC1 | 0.003180114 | 3.056913217 |
| hsa-circRNA6634-2_CBC1 | 0.020482987 | 2.395128241 |
| hsa_circ_0068321_CBC1 | 0.001066637 | 2.211773974 |
| hsa_circ_0045443_CBC1 | 0.021690222 | 2.168752355 |
| hsa-circRNA6265-8_CBC1 | 0.022032628 | 2.784341411 |
| hsa_circ_0063513_CBC1 | 0.023368068 | 2.225228367 |
| hsa_circ_0042339_CBC1 | 0.000634582 | 3.78621478 |
| hsa_circ_0074789_CBC1 | 0.039839561 | 2.045585279 |
| hsa_circ_0095561_CBC1 | 0.004377964 | 3.022446573 |
| hsa_circ_0092946_CBC1 | 0.003182442 | 2.647485969 |
| hsa_circ_0013509_CBC1 | 0.002140288 | 2.051273189 |
| hsa_circ_0011713_CBC1 | 0.007673214 | 12.02904345 |
| hsa_circ_0036083_CBC1 | 0.000344669 | 3.440271165 |
| hsa_circ_0090166_CBC1 | 0.007893672 | 2.159977332 |
| hsa_circ_0012067_CBC1 | 0.005228104 | 2.195026283 |
| hsa-circRNA11595-34_CBC1 | 0.012146101 | 2.843528268 |
| hsa-circRNA5213-13_CBC1 | 0.003029549 | 5.783482751 |
| hsa_circ_0066760_CBC1 | 0.019563624 | 3.439248606 |
| hsa_circ_0072764_CBC1 | 3.66213E-05 | 7.01293761 |
| hsa-circRNA1426-4_CBC1 | 0.004737624 | 3.658267496 |
| hsa_circ_0014863_CBC1 | 0.001547069 | 2.776564236 |
| hsa_circ_0088955_CBC1 | 0.001534086 | 2.197562422 |
| hsa-circRNA11393-13_CBC1 | 0.000193459 | 6.248847904 |
| hsa_circ_0121370_CBC1 | 0.010212346 | 2.167623393 |
| hsa_circ_0040740_CBC1 | 0.002404178 | 6.654781071 |
| hsa_circ_0029694_CBC1 | 0.004266957 | 6.644579466 |
| hsa-circRNA15229-3_CBC1 | 0.000263601 | 3.191267292 |
| hsa_circ_0036709_CBC1 | 0.002206506 | 4.902467814 |
| hsa_circ_0081411_CBC1 | 0.000263491 | 3.964911822 |
| hsa_circ_0121227_CBC1 | 5.87615E-05 | 6.829512267 |
| hsa_circ_0049217_CBC1 | 0.000617873 | 3.564858549 |
| hsa-circRNA6901-19_CBC1 | 0.03538108 | 2.551686532 |
| hsa_circ_0050181_CBC1 | 0.002810687 | 2.028393525 |
| hsa-circRNA6178-4_CBC1 | 0.014603769 | 2.775023681 |
| hsa_circ_0084474_CBC1 | 0.001716453 | 2.434522018 |
| hsa-circRNA11493-10_CBC1 | 0.000894154 | 5.764133968 |
| hsa_circ_0003555_CBC1 | 0.002958696 | 2.031429909 |
| hsa-circRNA14109-3_CBC1 | 0.01157214 | 3.042744505 |
| hsa-circRNA2033-58_CBC1 | 0.002935018 | 2.352835607 |
| hsa_circ_0105090_CBC1 | 0.002327574 | 2.691736007 |
| hsa_circ_0049193_CBC1 | 0.002066397 | 2.543326808 |
| hsa-circRNA2313-1_CBC1 | 0.006881154 | 2.279845035 |
| hsa-circRNA1649-9_CBC1 | 0.000456178 | 3.355863689 |
| hsa_circ_0111200_CBC1 | 0.009006605 | 2.023087401 |
| hsa_circ_0137794_CBC1 | 0.002576733 | 3.069600588 |
| hsa_circ_0012019_CBC1 | 1.72012E-07 | 12.48626497 |
| hsa-circRNA2520-22_CBC1 | 0.000262912 | 4.3465023 |
| hsa_circ_0003209_CBC1 | 0.000245381 | 6.237737747 |
| hsa_circ_0005368_CBC1 | 3.83422E-05 | 4.846768414 |
| hsa-circRNA15727-16_CBC1 | 0.005262528 | 8.154572759 |
| hsa-circRNA10887-2_CBC1 | 0.000446532 | 2.601545805 |
| hsa_circ_0029104_CBC1 | 0.005217137 | 2.285451317 |
| hsa_circ_0081556_CBC1 | 0.001963574 | 2.271129348 |
| hsa-circRNA8092-4_CBC1 | 0.002694141 | 6.261630376 |
| hsa_circ_0084641_CBC1 | 0.008466092 | 4.463358172 |
| hsa-circRNA2520-29_CBC1 | 0.000239624 | 8.575600575 |
| hsa_circ_0035492_CBC1 | 3.24681E-05 | 6.386727546 |
| hsa-circRNA14090_CBC1 | 0.002768795 | 8.33172919 |
| hsa_circ_0056156_CBC1 | 0.022281721 | 3.543713098 |
| hsa-circRNA3588-4_CBC1 | 0.001525919 | 4.994434902 |
| hsa_circ_0040938_CBC1 | 0.010992866 | 2.721855151 |
| hsa-circRNA2520-1_CBC1 | 0.002001308 | 4.603997038 |
| hsa_circ_0031979_CBC1 | 0.000623235 | 6.967007465 |
| hsa_circ_0028545_CBC1 | 0.00477522 | 2.233451119 |
| hsa_circ_0060208_CBC1 | 0.000174863 | 5.240548216 |
| hsa-circRNA8500-18_CBC1 | 0.00025111 | 3.712112899 |
| hsa-circRNA1030-4_CBC1 | 0.000692894 | 4.223788026 |
| hsa-circRNA10312-6_CBC1 | 0.000815158 | 3.544835966 |
| hsa_circ_0042373_CBC1 | 0.00308427 | 2.561644048 |
| hsa_circ_0019642_CBC1 | 0.038904198 | 2.098228071 |
| hsa_circ_0088989_CBC1 | 0.000168998 | 2.272322269 |
| hsa_circ_0125527_CBC1 | 0.016601841 | 2.118435028 |
| hsa-circRNA2033-108_CBC1 | 0.003380479 | 2.838099421 |
| hsa_circ_0067426_CBC1 | 0.006833208 | 2.65216984 |
| hsa-circRNA14251-13_CBC1 | 0.012179342 | 2.056948955 |
| hsa_circ_0125561_CBC1 | 0.00114004 | 2.702463337 |
| hsa-circRNA11137-6_CBC1 | 0.016868527 | 2.140802558 |
| hsa_circ_0036696_CBC1 | 0.000378172 | 3.766585634 |
| hsa_circ_0104748_CBC1 | 0.005044937 | 2.820862186 |
| hsa_circ_0100052_CBC1 | 0.002487827 | 2.783302957 |
| hsa_circ_0051240_CBC1 | 0.007873792 | 2.173232219 |
| hsa-circRNA10893-8_CBC1 | 0.000362261 | 5.492244774 |
| hsa_circ_0067171_CBC1 | 0.000759568 | 3.655469838 |
| hsa-circRNA3581-168_CBC1 | 0.000211973 | 2.609221434 |
| hsa-circRNA6777-1_CBC1 | 0.001322389 | 3.094632115 |
| hsa_circ_0054471_CBC1 | 0.002043296 | 2.016658725 |
| hsa_circ_0056031_CBC1 | 0.002340163 | 11.47147601 |
| hsa_circ_0076752_CBC1 | 0.001588934 | 2.837760175 |
| hsa-circRNA11981-46_CBC1 | 0.001609719 | 3.042953915 |
| hsa_circ_0050682_CBC1 | 4.86173E-05 | 3.693928372 |
| hsa-circRNA11175-3_CBC1 | 0.000572154 | 2.25835588 |
| hsa_circ_0070685_CBC1 | 0.001607375 | 3.476742997 |
| hsa_circ_0102373_CBC1 | 0.001431829 | 2.580243413 |
| hsa-circRNA15355-2_CBC1 | 0.006456486 | 2.67857821 |
| hsa_circ_0032091_CBC1 | 0.018078045 | 2.112200962 |
| hsa_circ_0000539_CBC1 | 0.001650091 | 8.595565351 |
| hsa_circ_0064205_CBC1 | 0.000118474 | 5.529895309 |
| hsa-circRNA7836-13_CBC1 | 3.75869E-05 | 2.104422355 |
| hsa_circ_0036701_CBC1 | 0.000790171 | 3.131044971 |
| hsa_circ_0033545_CBC1 | 0.000113771 | 2.764376814 |
| hsa_circ_0038754_CBC1 | 0.002500298 | 2.115184808 |
| hsa_circ_0126907_CBC1 | 0.028564366 | 2.109683966 |
| hsa_circ_0064234_CBC1 | 0.001156532 | 4.83148157 |
| hsa_circ_0065034_CBC1 | 0.014112151 | 2.978005398 |
| hsa_circ_0015908_CBC1 | 0.000338006 | 2.108344099 |
| hsa_circ_0075887_CBC1 | 0.01226982 | 2.008679517 |
| hsa_circ_0102385_CBC1 | 0.000434175 | 6.082233856 |
| hsa_circ_0064237_CBC1 | 0.000585268 | 5.032956082 |
| hsa_circ_0012367_CBC1 | 0.001033765 | 3.051603964 |
| hsa-circRNA7825-10_CBC1 | 0.001167699 | 2.356235097 |
| hsa-circRNA9060-20_CBC1 | 0.009577509 | 2.888760639 |
| hsa-circRNA9443-9_CBC1 | 0.000909033 | 4.260642091 |
| hsa-circRNA15355-31_CBC1 | 0.014009106 | 2.298185557 |
| hsa-circRNA2527-20_CBC1 | 0.001790113 | 7.204747206 |
| hsa-circRNA5346-55_CBC1 | 0.018451393 | 2.05878673 |
| hsa-circRNA11981-60_CBC1 | 7.3099E-05 | 2.807620521 |
| hsa_circ_0062023_CBC1 | 0.009203895 | 2.008118705 |
| hsa_circ_0101893_CBC1 | 0.007144136 | 2.508235516 |
| hsa_circ_0071538_CBC1 | 0.002900556 | 3.71042463 |
| hsa_circ_0124818_CBC1 | 0.001382429 | 2.97559726 |
| hsa-circRNA3533-3_CBC1 | 0.008463924 | 4.16899285 |
| hsa-circRNA3897-6_CBC1 | 0.00630703 | 2.860965095 |
| hsa_circ_0111962_CBC1 | 0.005919441 | 7.589401906 |
| hsa-circRNA4002-1_CBC1 | 0.000737106 | 2.085127185 |
| hsa_circ_0004887_CBC1 | 0.001298557 | 3.088757623 |
| hsa_circ_0019163_CBC1 | 0.001870316 | 7.669981499 |
| hsa_circ_0091422_CBC1 | 0.018770992 | 2.069842716 |
| hsa-circRNA13043-32_CBC1 | 0.025558967 | 3.969694102 |
| hsa_circ_0064228_CBC1 | 0.004760029 | 5.525112853 |
| hsa_circ_0005284_CBC1 | 0.000316165 | 2.588875633 |
| hsa_circ_0085467_CBC1 | 0.006570542 | 3.297480218 |
| hsa_circ_0103713_CBC1 | 0.015730486 | 3.886720819 |
| hsa-circRNA8518-52_CBC1 | 0.008916237 | 2.695038464 |
| hsa_circ_0040494_CBC1 | 0.000137352 | 2.821128549 |
| hsa_circ_0021534_CBC1 | 0.002311709 | 3.910572294 |
| hsa_circ_0049738_CBC1 | 7.61215E-06 | 3.247965607 |
| hsa_circ_0031127_CBC1 | 0.001768636 | 2.429355628 |
| hsa-circRNA13698-50_CBC1 | 0.009696012 | 6.590520151 |
| hsa_circ_0123864_CBC1 | 0.000912955 | 3.306507357 |
| hsa-circRNA15355-32_CBC1 | 0.002610671 | 3.016842654 |
| hsa-circRNA4910-10_CBC1 | 3.52711E-05 | 2.751053524 |
| hsa_circ_0083898_CBC1 | 0.001511282 | 2.722982146 |
| hsa_circ_0034566_CBC1 | 0.000312808 | 15.14931832 |
| hsa_circ_0064211_CBC1 | 0.004548489 | 3.446319981 |
| hsa_circ_0125196_CBC1 | 0.000618263 | 7.180867075 |
| hsa-circRNA8500-50_CBC1 | 0.000989579 | 3.809696147 |
| hsa_circ_0088284_CBC1 | 0.000502587 | 2.836889877 |
| hsa-circRNA7836-35_CBC1 | 0.006289129 | 3.414438226 |
| hsa-circRNA10893-20_CBC1 | 0.000162475 | 5.924968802 |
| hsa_circ_0081409_CBC1 | 0.002690719 | 3.446195635 |
| hsa_circ_0021913_CBC1 | 0.000539019 | 2.364315574 |
| hsa-circRNA14309-4_CBC1 | 0.006032255 | 2.793634946 |
| hsa_circ_0012618_CBC1 | 0.043518906 | 2.248983839 |
| hsa_circ_0025151_CBC1 | 1.85951E-05 | 4.009507761 |
| hsa_circ_0005015_CBC1 | 0.04633115 | 3.108418446 |
| hsa_circ_0102722_CBC1 | 0.013879263 | 3.851775412 |
| hsa_circ_0086752_CBC1 | 7.62523E-05 | 8.230671757 |
| hsa-circRNA3353-5_CBC1 | 0.038226173 | 3.495724909 |
| hsa_circ_0084383_CBC1 | 0.000816109 | 2.15159688 |
| hsa-circRNA427-5_CBC1 | 0.023236577 | 5.192317107 |
| hsa-circRNA8964-5_CBC1 | 0.000107774 | 2.026142712 |
| hsa_circ_0021737_CBC1 | 0.016369666 | 2.305124545 |
| hsa_circ_0031783_CBC1 | 0.01144713 | 3.625774602 |
| hsa_circ_0125863_CBC1 | 0.009820639 | 2.745378386 |
| hsa_circ_0055198_CBC1 | 0.011480122 | 2.707239841 |
| hsa_circ_0088307_CBC1 | 0.000566635 | 2.696119306 |
| hsa-circRNA15902-24_CBC1 | 0.029178991 | 3.266774401 |
| hsa_circ_0036912_CBC1 | 0.000839663 | 5.227636135 |
| hsa-circRNA11981-68_CBC1 | 0.003131071 | 2.411761484 |
| hsa_circ_0114518_CBC1 | 0.028276916 | 2.304294756 |
| hsa_circ_0106211_CBC1 | 0.012686351 | 2.739400792 |
| hsa_circ_0080640_CBC1 | 0.001514677 | 2.22407603 |
| hsa_circ_0131460_CBC1 | 0.000454245 | 2.139310282 |
| hsa_circ_0090630_CBC1 | 0.000149849 | 2.653028603 |
| hsa-circRNA3353-9_CBC1 | 0.011465715 | 3.948471723 |
| hsa-circRNA3767-4_CBC1 | 0.000728609 | 2.086435226 |
| hsa-circRNA1038-2_CBC1 | 0.013378111 | 3.967524882 |
| hsa-circRNA9154-18_CBC1 | 0.002412261 | 2.128617462 |
| hsa_circ_0121383_CBC1 | 0.003780964 | 4.072444436 |
| hsa_circ_0107804_CBC1 | 0.013153666 | 2.131807439 |
| hsa-circRNA11595-36_CBC1 | 0.00162628 | 2.787818365 |
| hsa-circRNA5149-6_CBC1 | 0.003771286 | 3.234800613 |
| hsa-circRNA11493-38_CBC1 | 0.001862543 | 5.880469168 |
| hsa-circRNA7044-10_CBC1 | 0.00073809 | 7.411255588 |
| hsa-circRNA7044-2_CBC1 | 0.001273418 | 7.372886664 |
| hsa_circ_0081188_CBC1 | 0.001590991 | 2.039436559 |
| hsa_circ_0049108_CBC1 | 0.001908522 | 3.30879482 |
| hsa_circ_0089842_CBC1 | 0.033728119 | 2.164588967 |
| hsa_circ_0079865_CBC1 | 0.000460587 | 7.051151518 |
| hsa_circ_0101456_CBC1 | 0.00099094 | 2.310955304 |
| hsa-circRNA2961-48_CBC1 | 0.001447147 | 2.13517889 |
| hsa_circ_0041031_CBC1 | 0.033122387 | 2.653948421 |
| hsa_circ_0110152_CBC1 | 0.002606832 | 3.275688762 |
| hsa_circ_0085460_CBC1 | 0.001766197 | 2.288874206 |
| hsa_circ_0016387_CBC1 | 0.030316469 | 3.77987421 |
| hsa-circRNA15598-10_CBC1 | 0.012040228 | 3.620904425 |
| hsa-circRNA2042-16_CBC1 | 0.003994074 | 2.013194725 |
| hsa_circ_0069273_CBC1 | 0.003603763 | 7.428340003 |
| hsa_circ_0026197_CBC1 | 0.016874226 | 7.291235317 |
| hsa_circ_0119183_CBC1 | 0.005087485 | 2.531014341 |
| hsa_circ_0097649_CBC1 | 0.001598062 | 3.014318801 |
| hsa_circ_0019216_CBC1 | 4.12842E-05 | 10.14633883 |
| hsa_circ_0075901_CBC1 | 0.040895966 | 2.58640503 |
| hsa_circ_0096196_CBC1 | 0.000548472 | 5.102184574 |
| hsa_circ_0113440_CBC1 | 0.002670551 | 2.003105212 |
| hsa-circRNA2536-1_CBC1 | 0.000106897 | 5.390962317 |
| hsa-circRNA8500-7_CBC1 | 0.002312067 | 3.283153469 |
| hsa_circ_0091240_CBC1 | 0.008655895 | 5.587479507 |
| hsa-circRNA2502-11_CBC1 | 0.000438807 | 2.726812306 |
| hsa_circ_0085995_CBC1 | 0.00475533 | 2.434561705 |
| hsa_circ_0040481_CBC1 | 0.000430659 | 3.562510367 |
| hsa-circRNA11180-32_CBC1 | 0.005377374 | 3.059856444 |
| hsa_circ_0111793_CBC1 | 0.039391127 | 2.877749261 |
| hsa_circ_0036871_CBC1 | 0.000862972 | 11.6583363 |
| hsa_circ_0137897_CBC1 | 0.046060103 | 2.473867105 |
| hsa_circ_0067837_CBC1 | 0.01657546 | 8.200067085 |
| hsa_circ_0027116_CBC1 | 0.0413705 | 2.371111389 |
| hsa_circ_0071652_CBC1 | 0.000768341 | 3.292206239 |
| hsa_circ_0068898_CBC1 | 0.000686089 | 3.34683781 |
| hsa-circRNA4666-21_CBC1 | 0.01208758 | 3.61396806 |
| hsa-circRNA5897-19_CBC1 | 0.005297035 | 2.297063641 |
| hsa_circ_0049093_CBC1 | 0.003028842 | 3.291560831 |
| hsa_circ_0136924_CBC1 | 0.039526088 | 2.151281386 |
| hsa-circRNA2502-16_CBC1 | 0.000178969 | 2.742051261 |
| hsa_circ_0005138_CBC1 | 0.04044273 | 2.930400239 |
| hsa_circ_0050710_CBC1 | 0.000211791 | 8.771846783 |
| hsa-circRNA4547-7_CBC1 | 5.37879E-06 | 6.461725102 |
| hsa_circ_0139350_CBC1 | 0.002502862 | 4.117187846 |
| hsa_circ_0061635_CBC1 | 0.005131073 | 2.486227302 |
| hsa-circRNA9331-4_CBC1 | 0.008674524 | 2.084583859 |
| hsa_circ_0102833_CBC1 | 1.7718E-05 | 3.376315156 |
| hsa_circ_0125971_CBC1 | 0.002443607 | 2.164550865 |
| hsa_circ_0090641_CBC1 | 0.00171557 | 2.615324278 |
| hsa_circ_0064246_CBC1 | 0.000351572 | 7.199335234 |
| hsa-circRNA4910-2_CBC1 | 0.000266641 | 4.316314916 |
| hsa_circ_0097514_CBC1 | 0.000723271 | 4.39733405 |
| hsa_circ_0064242_CBC1 | 0.015161422 | 2.074097161 |
| hsa-circRNA9989-2_CBC1 | 0.00012141 | 2.623548066 |
| hsa_circ_0063165_CBC1 | 0.000948601 | 2.532476348 |
| hsa-circRNA1649-6_CBC1 | 0.000677501 | 3.072388745 |
| hsa_circ_0056035_CBC1 | 0.000127775 | 9.74455613 |
| hsa-circRNA2329-16_CBC1 | 0.00198136 | 4.518301488 |
| hsa_circ_0059378_CBC1 | 0.021264799 | 2.055028601 |
| hsa_circ_0111109_CBC1 | 0.00031903 | 2.8060867 |
| hsa_circ_0028681_CBC1 | 0.000106525 | 7.681660194 |
| hsa-circRNA15902-38_CBC1 | 0.004870614 | 2.18833931 |
| hsa-circRNA9989-7_CBC1 | 2.54151E-06 | 3.295367601 |
| hsa_circ_0138713_CBC1 | 0.039035682 | 2.037204725 |
| hsa-circRNA14224-10_CBC1 | 0.000202049 | 2.805042438 |
| hsa-circRNA3548_CBC1 | 6.24002E-05 | 4.360346407 |
| hsa_circ_0113054_CBC1 | 0.003783148 | 2.096755807 |
| hsa_circ_0043549_CBC1 | 0.001358431 | 4.074159543 |
| hsa-circRNA9555-10_CBC1 | 0.000157389 | 2.218286566 |
| hsa_circ_0063607_CBC1 | 0.016593073 | 2.33115784 |
| hsa_circ_0046298_CBC1 | 0.001465483 | 2.303504807 |
| hsa_circ_0088311_CBC1 | 0.024662103 | 3.628304556 |
| hsa_circ_0076756_CBC1 | 0.00568663 | 3.704681934 |
| hsa_circ_0059375_CBC1 | 0.01637731 | 2.061585902 |
| hsa_circ_0043671_CBC1 | 0.008842306 | 2.013122454 |
| hsa_circ_0065758_CBC1 | 0.000101009 | 4.595091775 |
| hsa-circRNA9333-2_CBC1 | 4.84542E-05 | 12.12390696 |
| hsa_circ_0064206_CBC1 | 0.00149259 | 3.334311172 |
| hsa_circ_0085465_CBC1 | 0.044708609 | 2.597443781 |
| hsa_circ_0116156_CBC1 | 0.003586971 | 2.99542851 |
| hsa_circ_0039802_CBC1 | 0.001171571 | 2.239125577 |
| hsa-circRNA5886-42_CBC1 | 4.24505E-06 | 2.460263599 |
| hsa_circ_0050797_CBC1 | 0.012543291 | 2.090460475 |
| hsa_circ_0015175_CBC1 | 0.008316308 | 2.47719016 |
| hsa_circ_0034576_CBC1 | 0.002540735 | 2.069091063 |
| hsa_circ_0055670_CBC1 | 3.93827E-05 | 8.149796975 |
| hsa_circ_0062271_CBC1 | 0.010072717 | 2.683054919 |
| hsa_circ_0004966_CBC1 | 0.003337484 | 2.497910364 |
| hsa-circRNA2639-16_CBC1 | 0.001168307 | 3.478061965 |
| hsa-circRNA3109-14_CBC1 | 0.000554976 | 2.662470221 |
| hsa_circ_0069019_CBC1 | 0.000640179 | 2.022118072 |
| hsa_circ_0009506_CBC1 | 0.002984893 | 2.058621324 |
| hsa-circRNA7964-3_CBC1 | 0.016454646 | 2.557524145 |
| hsa_circ_0009810_CBC1 | 0.002420185 | 2.367932563 |
| hsa_circ_0016241_CBC1 | 0.002087896 | 2.218005408 |
| hsa_circ_0035045_CBC1 | 0.044331864 | 2.406687469 |
| hsa_circ_0024903_CBC1 | 0.000304419 | 2.948462433 |
| hsa-circRNA888-14_CBC1 | 0.002354516 | 3.472575921 |
| hsa_circ_0043176_CBC1 | 0.025744762 | 2.485732637 |
| hsa-circRNA10466-23_CBC1 | 0.030411451 | 2.060630225 |
| hsa-circRNA4355-4_CBC1 | 0.000335347 | 4.097803981 |
| hsa_circ_0088977_CBC1 | 8.83589E-05 | 2.71179085 |
| hsa_circ_0048387_CBC1 | 0.003243944 | 2.47645018 |
| hsa_circ_0084480_CBC1 | 0.012838219 | 3.245914052 |
| hsa-circRNA946-17_CBC1 | 0.002836223 | 4.439053961 |
| hsa_circ_0025042_CBC1 | 6.61783E-06 | 3.99516419 |
| hsa_circ_0025032_CBC1 | 0.000448541 | 3.762615781 |
| hsa_circ_0025147_CBC1 | 0.000567438 | 3.301421123 |
| hsa_circ_0015759_CBC1 | 0.001310501 | 4.82635332 |
| hsa-circRNA15902-26_CBC1 | 0.000512639 | 2.582610031 |
| hsa_circ_0102391_CBC1 | 0.000264268 | 4.526616941 |
| hsa_circ_0076151_CBC1 | 0.033800093 | 3.425638708 |
| hsa_circ_0090636_CBC1 | 0.029813499 | 4.567797762 |
| hsa_circ_0011101_CBC1 | 0.001524606 | 2.04614588 |
| hsa_circ_0089659_CBC1 | 0.000398119 | 2.708167407 |
| hsa-circRNA2097-16_CBC1 | 0.001048171 | 3.759892267 |
| hsa-circRNA15355-28_CBC1 | 0.002487643 | 2.429560436 |
| hsa_circ_0076149_CBC1 | 0.036918554 | 2.809321441 |
| hsa-circRNA15389-1_CBC1 | 0.016230038 | 2.838725101 |
| hsa_circ_0065981_CBC1 | 0.006116565 | 4.234402461 |
| hsa_circ_0053167_CBC1 | 0.008971083 | 2.615323131 |
| hsa-circRNA2502-13_CBC1 | 0.001342754 | 2.043096708 |
| hsa_circ_0006862_CBC1 | 0.007884897 | 4.568012406 |
| hsa_circ_0062534_CBC1 | 0.019879357 | 2.135612392 |
| hsa_circ_0015894_CBC1 | 0.001859067 | 2.240823523 |
| hsa_circ_0097690_CBC1 | 0.005979589 | 2.526910745 |
| hsa-circRNA2623-6_CBC1 | 0.022459559 | 3.753588772 |
| hsa_circ_0072241_CBC1 | 0.013647547 | 2.328510154 |
| hsa-circRNA7663-28_CBC1 | 0.017827022 | 2.548769761 |
| hsa-circRNA14051-5_CBC1 | 0.024554496 | 2.272139316 |
| hsa-circRNA6760-2_CBC1 | 0.000417546 | 2.076638405 |
| hsa_circ_0056028_CBC1 | 9.72322E-05 | 10.81469195 |
| hsa_circ_0013128_CBC1 | 0.021664489 | 2.135186969 |
| hsa-circRNA9240-12_CBC1 | 0.022495163 | 2.553603038 |
| hsa_circ_0012606_CBC1 | 0.000873249 | 2.574797935 |
| hsa_circ_0032161_CBC1 | 0.012677445 | 3.552045992 |
| hsa_circ_0074523_CBC1 | 0.002871328 | 2.898837075 |
| hsa-circRNA7777-22_CBC1 | 0.002134246 | 3.885901391 |
| hsa_circ_0059327_CBC1 | 0.000718755 | 4.372602147 |
| hsa_circ_0009776_CBC1 | 0.014825361 | 2.05967375 |
| hsa_circ_0095817_CBC1 | 0.010685512 | 3.103981586 |
| hsa-circRNA5936-11_CBC1 | 0.00368721 | 8.04536884 |
| hsa_circ_0012215_CBC1 | 0.002017076 | 3.425466478 |
| hsa_circ_0106001_CBC1 | 0.006115711 | 4.118448584 |
| hsa_circ_0079874_CBC1 | 0.000288004 | 6.017904803 |
| hsa_circ_0088962_CBC1 | 7.5766E-05 | 2.609319633 |
| hsa-circRNA10887-8_CBC1 | 0.002415573 | 2.443064179 |
| hsa_circ_0129999_CBC1 | 0.000542202 | 3.971532736 |
| hsa_circ_0042021_CBC1 | 0.001539581 | 2.027392533 |
| hsa_circ_0036911_CBC1 | 0.000182601 | 7.167693296 |
| hsa_circ_0036711_CBC1 | 5.21508E-05 | 4.413391073 |
| hsa_circ_0090632_CBC1 | 0.000212873 | 2.932589711 |
| hsa_circ_0050706_CBC1 | 1.40306E-06 | 2.894548089 |
| hsa_circ_0009933_CBC1 | 0.01291781 | 2.281113527 |
| hsa_circ_0007574_CBC1 | 0.011425573 | 2.226799637 |
| hsa_circ_0074580_CBC1 | 0.000982242 | 2.093766398 |
| hsa_circ_0089186_CBC1 | 0.00242254 | 2.058056526 |
| hsa_circ_0042379_CBC1 | 0.000959121 | 4.036252385 |
| hsa_circ_0050289_CBC1 | 0.003380075 | 2.617730638 |
| hsa_circ_0090167_CBC1 | 0.008032671 | 2.580983151 |
| hsa-circRNA11493-49_CBC1 | 0.000529587 | 6.326997123 |
| hsa_circ_0055216_CBC1 | 0.006202221 | 2.810968792 |
| hsa_circ_0104334_CBC1 | 0.002210658 | 3.499210747 |
| hsa_circ_0067841_CBC1 | 0.000108361 | 5.325943929 |
| hsa_circ_0068023_CBC1 | 0.004463997 | 3.565764427 |
| hsa-circRNA2520-12_CBC1 | 0.00068772 | 10.02669289 |
| hsa-circRNA264-3_CBC1 | 0.00032174 | 6.791269067 |
| hsa-circRNA7713-2_CBC1 | 0.005614149 | 4.709863395 |
| hsa_circ_0063047_CBC1 | 4.10683E-05 | 2.37122571 |
| hsa_circ_0138062_CBC1 | 0.000500885 | 2.488680088 |
| hsa_circ_0026185_CBC1 | 0.002694563 | 3.539648497 |
| hsa_circ_0029173_CBC1 | 0.001440383 | 2.738473589 |
| hsa_circ_0087795_CBC1 | 0.01249284 | 3.132698986 |
| hsa_circ_0038637_CBC1 | 5.88515E-05 | 13.9969007 |
| hsa-circRNA7393-6_CBC1 | 5.39147E-05 | 9.376340872 |
| hsa_circ_0098810_CBC1 | 0.013395114 | 2.254129805 |
| hsa-circRNA5028-6_CBC1 | 0.000264755 | 10.50567638 |
| hsa_circ_0100099_CBC1 | 0.009009723 | 3.075029272 |
| hsa_circ_0070149_CBC1 | 0.014361199 | 2.115269373 |
| hsa_circ_0140379_CBC1 | 0.003188058 | 2.689442615 |
| hsa-circRNA9443-23_CBC1 | 0.008869004 | 3.330439165 |
| hsa_circ_0129342_CBC1 | 0.004722207 | 3.679807166 |
| hsa_circ_0065386_CBC1 | 0.006824289 | 2.14634922 |
| hsa-circRNA10206-26_CBC1 | 0.000357814 | 3.421205845 |
| hsa_circ_0102234_CBC1 | 0.006569753 | 2.155519852 |
| hsa-circRNA7836-41_CBC1 | 0.000166538 | 2.964665421 |
| hsa_circ_0102372_CBC1 | 0.001713025 | 4.263546033 |
| hsa-circRNA2033-31_CBC1 | 0.002320899 | 2.895916045 |
| hsa_circ_0115786_CBC1 | 0.00071326 | 3.042893602 |
| hsa_circ_0033133_CBC1 | 0.002590845 | 3.149142231 |
| hsa_circ_0088890_CBC1 | 0.007444106 | 2.463503132 |
| hsa_circ_0064208_CBC1 | 0.001574686 | 4.144805586 |
| hsa_circ_0001453_CBC1 | 0.007472082 | 3.122730951 |
| hsa_circ_0084477_CBC1 | 0.001442038 | 2.336380092 |
| hsa_circ_0024914_CBC1 | 0.000207234 | 2.81263948 |
| hsa_circ_0021907_CBC1 | 0.000573882 | 2.168303782 |
| hsa_circ_0052999_CBC1 | 0.000493597 | 3.825572109 |
| hsa_circ_0011712_CBC1 | 7.04958E-05 | 7.370633721 |
| hsa_circ_0007906_CBC1 | 0.000146423 | 2.981747693 |
| hsa_circ_0084264_CBC1 | 0.007530171 | 2.06179197 |
| hsa_circ_0088934_CBC1 | 0.00049576 | 2.39430321 |
| hsa_circ_0037538_CBC1 | 0.00123067 | 6.982882328 |
| hsa_circ_0006549_CBC1 | 0.02472438 | 3.067639099 |
| hsa_circ_0040580_CBC1 | 0.000296019 | 3.226737737 |
| hsa_circ_0090959_CBC1 | 0.001583114 | 6.660624946 |
| hsa-circRNA14838-128_CBC1 | 0.002511378 | 2.243229142 |
| hsa-circRNA2033-128_CBC1 | 0.016128432 | 2.23058737 |
| hsa-circRNA2719-24_CBC1 | 0.003931002 | 4.900265377 |
| hsa_circ_0048978_CBC1 | 0.000238423 | 2.401922067 |
| hsa_circ_0052585_CBC1 | 0.000141963 | 5.186679495 |
| hsa-circRNA10541-31_CBC1 | 0.003847543 | 3.838120895 |
| hsa_circ_0073985_CBC1 | 0.048356239 | 2.096192006 |
| hsa_circ_0001388_CBC1 | 0.000140518 | 2.544707111 |
| hsa_circ_0086150_CBC1 | 0.02083302 | 3.341772607 |
| hsa_circ_0114919_CBC1 | 0.043211827 | 2.571815737 |
| hsa-circRNA5897-21_CBC1 | 0.004886726 | 2.074345391 |
| hsa_circ_0007453_CBC1 | 9.52227E-05 | 2.521151562 |
| hsa_circ_0036921_CBC1 | 9.15609E-05 | 5.989207447 |
| hsa_circ_0069816_CBC1 | 0.037557989 | 2.087988029 |
| hsa-circRNA7393-1_CBC1 | 0.000515177 | 10.8166256 |
| hsa_circ_0035047_CBC1 | 0.019250789 | 2.704156699 |
| hsa_circ_0034563_CBC1 | 0.000112067 | 6.648011613 |
| hsa-circRNA1790-14_CBC1 | 0.000430534 | 10.56721865 |
| hsa-circRNA9996-5_CBC1 | 0.033602903 | 2.846598573 |
| hsa_circ_0019164_CBC1 | 0.000161777 | 11.48987289 |
| hsa_circ_0025169_CBC1 | 0.000310873 | 3.106524433 |
| hsa_circ_0059674_CBC1 | 0.007538795 | 3.120656714 |
| hsa_circ_0111959_CBC1 | 0.000930901 | 7.34703549 |
| hsa_circ_0000409_CBC1 | 0.01769142 | 2.190778507 |
| hsa-circRNA7836-21_CBC1 | 0.004542821 | 2.206677332 |
| hsa-circRNA15450-2_CBC1 | 0.002214709 | 9.160195744 |
| hsa_circ_0068335_CBC1 | 0.000588505 | 3.072695171 |
| hsa-circRNA4965-20_CBC1 | 0.000135753 | 2.905752685 |
| hsa-circRNA3109-13_CBC1 | 0.000304332 | 4.126116147 |
| hsa-circRNA14257-13_CBC1 | 0.019384152 | 2.527943949 |
| hsa_circ_0008854_CBC1 | 0.001978576 | 4.814507473 |
| hsa_circ_0090945_CBC1 | 0.000165747 | 3.160117229 |
| hsa_circ_0028707_CBC1 | 0.000237106 | 8.430218026 |
| hsa-circRNA10457-6_CBC1 | 0.008487107 | 2.17784284 |
| hsa_circ_0065353_CBC1 | 0.019425735 | 3.956197866 |
| hsa_circ_0025162_CBC1 | 4.10614E-05 | 3.275028258 |
| hsa_circ_0111110_CBC1 | 0.00102326 | 2.269163847 |
| hsa_circ_0022129_CBC1 | 0.002396967 | 2.434277785 |
| hsa_circ_0002597_CBC1 | 0.000193823 | 7.926309093 |
| hsa_circ_0137830_CBC1 | 0.021409855 | 2.070096469 |
| hsa_circ_0081390_CBC1 | 0.041648835 | 2.809614826 |
| hsa_circ_0088901_CBC1 | 0.009161711 | 3.376091515 |
| hsa_circ_0028593_CBC1 | 0.002513373 | 2.355278629 |
| hsa-circRNA2033-16_CBC1 | 0.002095706 | 2.511845145 |
| hsa_circ_0016238_CBC1 | 0.000688025 | 2.543266237 |
| hsa_circ_0001135_CBC1 | 1.15273E-05 | 6.395438918 |
| hsa_circ_0068020_CBC1 | 0.000506464 | 3.684048165 |
| hsa_circ_0060463_CBC1 | 0.000439097 | 3.848850796 |
| hsa_circ_0070688_CBC1 | 0.018337329 | 2.35453337 |
| hsa_circ_0090302_CBC1 | 0.016890573 | 3.028556485 |
| hsa-circRNA12944-3_CBC1 | 0.00785022 | 2.993530397 |
| hsa-circRNA2502-12_CBC1 | 0.000130403 | 4.411615019 |
| hsa_circ_0043548_CBC1 | 0.001045055 | 4.616172601 |
| hsa_circ_0136406_CBC1 | 0.007972881 | 2.636012816 |
| hsa_circ_0093082_CBC1 | 0.002468414 | 2.262151095 |
| hsa_circ_0056024_CBC1 | 0.014893026 | 7.430961946 |
| hsa_circ_0064430_CBC1 | 6.43705E-05 | 2.155699543 |
| hsa_circ_0036926_CBC1 | 0.000133367 | 6.483920449 |
| hsa_circ_0087596_CBC1 | 0.000553663 | 2.457314467 |
| hsa_circ_0007034_CBC1 | 0.001701212 | 2.021685407 |
| hsa_circ_0061636_CBC1 | 0.004987348 | 2.798820759 |
| hsa_circ_0075989_CBC1 | 0.009932846 | 2.120149512 |
| hsa_circ_0084088_CBC1 | 0.010253327 | 2.090796644 |
| hsa_circ_0074792_CBC1 | 0.00800872 | 2.067457317 |
| hsa-circRNA14433-3_CBC1 | 0.007439511 | 2.290565774 |
| hsa-circRNA11493-48_CBC1 | 0.001137063 | 6.891857927 |
| hsa-circRNA8885-17_CBC1 | 0.000104019 | 8.832963136 |
| hsa_circ_0003161_CBC1 | 0.002621344 | 2.240428082 |
| hsa_circ_0037531_CBC1 | 0.005056051 | 7.559230845 |
| hsa_circ_0044832_CBC1 | 0.000445321 | 3.035700911 |
| hsa-circRNA1705_CBC1 | 0.021355831 | 2.525953792 |
| hsa-circRNA14645-4_CBC1 | 0.007801076 | 2.17560064 |
| hsa_circ_0110328_CBC1 | 0.001722159 | 4.124280001 |
| hsa_circ_0051654_CBC1 | 7.28069E-05 | 2.0313111 |
| hsa_circ_0010306_CBC1 | 0.003124159 | 2.044910936 |
| hsa-circRNA6947-2_CBC1 | 0.020501871 | 2.496779693 |
| hsa-circRNA15389-16_CBC1 | 0.000681163 | 5.517805349 |
| hsa_circ_0044126_CBC1 | 0.00065762 | 11.54692676 |
| hsa_circ_0056027_CBC1 | 0.000922872 | 16.27979807 |
| hsa_circ_0082003_CBC1 | 0.002442936 | 2.112805128 |
| hsa_circ_0081397_CBC1 | 0.018647072 | 3.497623479 |
| hsa_circ_0110447_CBC1 | 0.005096573 | 2.260187792 |
| hsa_circ_0039960_CBC1 | 0.008170966 | 2.118700873 |
| hsa_circ_0139548_CBC1 | 0.020510033 | 2.310604331 |
| hsa_circ_0027681_CBC1 | 0.012996126 | 2.044359733 |
| hsa-circRNA12057-5_CBC1 | 0.007128518 | 6.116888741 |
| hsa_circ_0133655_CBC1 | 0.005649977 | 2.148810643 |
| hsa_circ_0120406_CBC1 | 0.006404257 | 4.387495134 |
| hsa_circ_0103035_CBC1 | 0.006870931 | 2.142765287 |
| hsa_circ_0036702_CBC1 | 0.002051311 | 3.159927946 |
| hsa_circ_0068322_CBC1 | 7.55484E-05 | 2.113904888 |
| hsa-circRNA3215-23_CBC1 | 2.25122E-05 | 2.068845608 |
| hsa-circRNA1030-13_CBC1 | 0.000795591 | 5.052378183 |
| hsa-circRNA711-2_CBC1 | 0.000688019 | 2.066244595 |
| hsa-circRNA2033-127_CBC1 | 0.000338058 | 2.321176163 |
| hsa_circ_0007042_CBC1 | 0.003224735 | 2.744333698 |
| hsa_circ_0044701_CBC1 | 0.001114297 | 2.195345069 |
| hsa_circ_0014752_CBC1 | 0.017223968 | 2.250816173 |
| hsa_circ_0134631_CBC1 | 0.035140776 | 2.623505936 |
| hsa-circRNA7787-5_CBC1 | 0.022484237 | 3.37145546 |
| hsa_circ_0014748_CBC1 | 3.95437E-05 | 4.644797987 |
| hsa-circRNA12453-7_CBC1 | 0.001404173 | 3.734586097 |
| hsa_circ_0024728_CBC1 | 0.009047178 | 2.358461288 |
| hsa-circRNA9996-14_CBC1 | 0.048341646 | 3.315574589 |
| hsa_circ_0009489_CBC1 | 0.003324105 | 2.947907671 |
| hsa_circ_0047949_CBC1 | 0.042201409 | 2.763306086 |
| hsa_circ_0076728_CBC1 | 0.013046318 | 7.241493996 |
| hsa-circRNA1594-3_CBC1 | 0.020701539 | 2.443728102 |
| hsa_circ_0028702_CBC1 | 3.94599E-06 | 5.686485224 |
| hsa-circRNA11711-38_CBC1 | 0.001588831 | 2.113424599 |
| hsa_circ_0082842_CBC1 | 0.006724554 | 2.680491133 |
| hsa_circ_0095556_CBC1 | 0.001074272 | 3.415261529 |
| hsa_circ_0039140_CBC1 | 0.028135879 | 2.08581769 |
| hsa_circ_0020487_CBC1 | 3.98113E-05 | 17.83199451 |
| hsa_circ_0025791_CBC1 | 0.001257502 | 4.788529264 |
| hsa_circ_0034612_CBC1 | 0.005641481 | 7.371689273 |
| hsa_circ_0034603_CBC1 | 0.002452417 | 6.284042285 |
| hsa-circRNA15435-2_CBC1 | 0.001127769 | 2.342134489 |
| hsa_circ_0065385_CBC1 | 0.00010657 | 3.329402184 |
| hsa_circ_0063603_CBC1 | 0.007205714 | 2.233207874 |
| hsa_circ_0082516_CBC1 | 0.034899732 | 2.551150221 |
| hsa_circ_0046127_CBC1 | 0.003035448 | 2.051521078 |
| hsa_circ_0025661_CBC1 | 0.001730943 | 2.119777486 |
| hsa_circ_0016707_CBC1 | 0.044184958 | 2.233691798 |
| hsa_circ_0126240_CBC1 | 0.04501048 | 2.070645592 |
| hsa_circ_0087794_CBC1 | 0.006829853 | 2.899013822 |
| hsa_circ_0034301_CBC1 | 0.000189123 | 3.373488191 |
| hsa_circ_0082533_CBC1 | 0.013577728 | 2.110519629 |
| hsa_circ_0005473_CBC1 | 0.000271919 | 2.077822723 |
| hsa_circ_0013324_CBC1 | 0.019477427 | 2.576622498 |
| hsa_circ_0071539_CBC1 | 0.004211219 | 3.197369637 |
| hsa_circ_0029518_CBC1 | 0.000396417 | 2.915254389 |
| hsa_circ_0041888_CBC1 | 0.000311014 | 2.221879256 |
| hsa-circRNA9443-8_CBC1 | 0.002368856 | 4.25257443 |
| hsa_circ_0034577_CBC1 | 0.002548237 | 2.356912238 |
| hsa_circ_0049404_CBC1 | 1.30437E-05 | 7.851422659 |
| hsa-circRNA14645-3_CBC1 | 0.00133198 | 2.069639312 |
| hsa-circRNA1030-20_CBC1 | 0.000692281 | 7.690940952 |
| hsa_circ_0034695_CBC1 | 0.004106119 | 11.31042282 |
| hsa_circ_0138299_CBC1 | 0.002055291 | 2.274215223 |
| hsa_circ_0088939_CBC1 | 1.25656E-05 | 2.671757806 |
| hsa_circ_0121452_CBC1 | 0.00072359 | 3.041274611 |
| hsa_circ_0062834_CBC1 | 0.001414418 | 2.229212788 |
| hsa_circ_0056036_CBC1 | 4.60891E-05 | 8.698222555 |
| hsa_circ_0036085_CBC1 | 0.00227756 | 4.647381573 |
| hsa_circ_0052584_CBC1 | 0.002082041 | 6.654589869 |
| hsa_circ_0030367_CBC1 | 0.000736701 | 3.576453073 |
| hsa-circRNA11838-48_CBC1 | 0.033872677 | 2.019131697 |
| hsa-circRNA8881-22_CBC1 | 0.000132902 | 9.608846767 |
| hsa_circ_0050680_CBC1 | 0.001076193 | 3.609724876 |
| hsa_circ_0077409_CBC1 | 0.029083196 | 3.395410045 |
| hsa-circRNA10149-29_CBC1 | 0.000664148 | 8.254060005 |
| hsa-circRNA10441-60_CBC1 | 0.008646435 | 4.581035954 |
| hsa_circ_0137508_CBC1 | 0.004377561 | 3.223027954 |
| hsa_circ_0139550_CBC1 | 0.011261994 | 2.023929438 |
| hsa_circ_0086766_CBC1 | 0.008550402 | 2.359671319 |
| hsa-circRNA858-21_CBC1 | 0.004410955 | 4.399305442 |
| hsa_circ_0034644_CBC1 | 0.002048047 | 2.261070008 |
| hsa-circRNA15007_CBC1 | 0.001038927 | 2.221435367 |
| hsa-circRNA2527-15_CBC1 | 0.002841134 | 8.14943028 |
| hsa_circ_0043525_CBC1 | 0.030470003 | 3.362522968 |
| hsa_circ_0034607_CBC1 | 0.006850008 | 4.421675007 |
| hsa-circRNA3795-5_CBC1 | 0.004143847 | 3.502483967 |
| hsa_circ_0088997_CBC1 | 0.000173287 | 3.078856262 |
| hsa_circ_0004085_CBC1 | 0.005372967 | 2.175130184 |
| hsa_circ_0018033_CBC1 | 0.0013284 | 3.471062076 |
| hsa_circ_0005696_CBC1 | 0.01357866 | 2.377142828 |
| hsa-circRNA3767-1_CBC1 | 0.000657278 | 2.401436132 |
| hsa_circ_0140380_CBC1 | 0.009574017 | 2.495233427 |
| hsa_circ_0103600_CBC1 | 0.020435342 | 2.433154093 |
| hsa_circ_0001047_CBC1 | 2.70642E-06 | 7.086403561 |
| hsa_circ_0070812_CBC1 | 0.003928901 | 4.032128892 |
| hsa_circ_0018035_CBC1 | 0.004949048 | 2.488867439 |
| hsa_circ_0005460_CBC1 | 0.003475192 | 2.33867765 |
| hsa-circRNA733-4_CBC1 | 0.007578403 | 4.874544478 |
| hsa_circ_0068993_CBC1 | 0.000638762 | 2.012537941 |
| hsa-circRNA8092-14_CBC1 | 0.000870364 | 5.297258845 |
| hsa-circRNA12177-2_CBC1 | 0.004899005 | 3.337160841 |
| hsa_circ_0039152_CBC1 | 0.004888018 | 2.107685899 |
| hsa-circRNA4910-9_CBC1 | 4.0665E-05 | 4.661132974 |
| hsa_circ_0059728_CBC1 | 9.93039E-05 | 6.295944591 |
| hsa_circ_0081395_CBC1 | 3.4801E-08 | 5.549418742 |
| hsa_circ_0002766_CBC1 | 0.0041092 | 4.942285231 |
| hsa_circ_0110307_CBC1 | 0.022218762 | 2.234833345 |
| hsa_circ_0076093_CBC1 | 0.01285092 | 2.400466236 |
| hsa_circ_0104843_CBC1 | 0.002656488 | 5.982925079 |
| hsa_circ_0087031_CBC1 | 0.003047311 | 2.595552284 |
| hsa-circRNA15598-9_CBC1 | 0.017207606 | 2.5609552 |
| hsa_circ_0023042_CBC1 | 0.014163165 | 2.503621193 |
| hsa_circ_0098261_CBC1 | 0.019755001 | 2.241493065 |
| hsa_circ_0098852_CBC1 | 0.004240346 | 2.681536989 |
| hsa-circRNA1760-5_CBC1 | 0.000320054 | 3.029187385 |
| hsa_circ_0100813_CBC1 | 0.000124527 | 8.877777143 |
| hsa_circ_0135005_CBC1 | 0.04472045 | 2.801176013 |
| hsa_circ_0024905_CBC1 | 0.000369617 | 6.308758199 |
| hsa_circ_0097577_CBC1 | 0.009505509 | 2.805548877 |
| hsa_circ_0071680_CBC1 | 0.000226552 | 6.887417185 |
| hsa-circRNA15389-2_CBC1 | 0.009881821 | 4.413304002 |
| hsa_circ_0112876_CBC1 | 0.004353735 | 2.475790037 |
| hsa_circ_0133542_CBC1 | 0.002359623 | 2.825253557 |
| hsa_circ_0138060_CBC1 | 0.001284844 | 2.136224473 |
| hsa-circRNA2520-19_CBC1 | 0.000118087 | 11.40985714 |
| hsa_circ_0033138_CBC1 | 0.025095383 | 2.244275062 |
| hsa_circ_0041885_CBC1 | 0.00084129 | 2.200987494 |
| hsa_circ_0049114_CBC1 | 0.011970614 | 5.646951563 |
| hsa_circ_0133062_CBC1 | 0.048042618 | 2.000641022 |
| hsa_circ_0038053_CBC1 | 0.002734841 | 2.266420811 |
| hsa-circRNA13330-7_CBC1 | 0.005194249 | 3.544835769 |
| hsa-circRNA11981-50_CBC1 | 0.000210713 | 3.635960705 |
| hsa_circ_0028453_CBC1 | 0.029873118 | 2.395540521 |
| hsa_circ_0136190_CBC1 | 0.031032166 | 2.63403336 |
| hsa-circRNA9789-3_CBC1 | 0.0058435 | 3.040312227 |
| hsa_circ_0021535_CBC1 | 0.001745473 | 8.017028754 |
| hsa_circ_0008122_CBC1 | 0.028116391 | 2.364804898 |
| hsa-circRNA3215-19_CBC1 | 0.005201883 | 2.292293187 |
| hsa_circ_0009258_CBC1 | 0.030460552 | 2.134610909 |
| hsa_circ_0018526_CBC1 | 0.007889827 | 2.940020098 |
| hsa_circ_0005428_CBC1 | 0.003120281 | 3.37683731 |
| hsa_circ_0068320_CBC1 | 0.000898308 | 2.267311719 |
| hsa-circRNA1257-45_CBC1 | 0.000347243 | 2.792745943 |
| hsa_circ_0003902_CBC1 | 4.91868E-05 | 7.97390086 |
| hsa-circRNA8500-53_CBC1 | 0.00106214 | 3.653837239 |
| hsa-circRNA7275-45_CBC1 | 0.014778384 | 2.705963928 |
| hsa_circ_0026192_CBC1 | 0.003568863 | 2.866804913 |
| hsa-circRNA8881-21_CBC1 | 0.002355952 | 26.66777525 |
| hsa_circ_0044129_CBC1 | 3.15312E-05 | 14.22583693 |
| hsa-circRNA10810-7_CBC1 | 0.003798491 | 2.136518866 |
| hsa_circ_0000050_CBC1 | 0.003739055 | 2.016780412 |
| hsa_circ_0074522_CBC1 | 0.000510951 | 2.365445491 |
| hsa_circ_0076303_CBC1 | 0.026225034 | 2.045713052 |
| hsa-circRNA8371-2_CBC1 | 0.032363379 | 2.687627595 |
| hsa_circ_0032224_CBC1 | 0.035761977 | 2.003424396 |
| hsa_circ_0112734_CBC1 | 0.029103398 | 3.482174474 |
| hsa_circ_0046126_CBC1 | 0.000328178 | 2.295676786 |
| hsa_circ_0115952_CBC1 | 0.001539327 | 2.545243862 |
| hsa-circRNA1649-7_CBC1 | 0.000338359 | 4.620614195 |
| hsa_circ_0002109_CBC1 | 0.002365839 | 6.259682819 |
| hsa_circ_0049226_CBC1 | 0.001644892 | 2.844609305 |
| hsa_circ_0063038_CBC1 | 0.003278347 | 3.12722536 |
| hsa_circ_0068024_CBC1 | 0.001592087 | 2.874762563 |
| hsa-circRNA15991-3_CBC1 | 0.001603067 | 2.109116167 |
| hsa_circ_0097655_CBC1 | 0.002603176 | 4.787146155 |
| hsa_circ_0029138_CBC1 | 0.001772769 | 2.981468894 |
| hsa_circ_0079879_CBC1 | 0.000898698 | 8.55199451 |
| hsa_circ_0092088_CBC1 | 0.00023538 | 2.547694731 |
| hsa-circRNA9929-3_CBC1 | 0.001921068 | 2.933835805 |
| hsa_circ_0047537_CBC1 | 0.002288595 | 2.236081254 |
| hsa-circRNA11595-4_CBC1 | 0.001835266 | 2.839578308 |
| hsa-circRNA8500-22_CBC1 | 0.004205049 | 3.814245557 |
| hsa_circ_0117984_CBC1 | 0.000638067 | 5.600835205 |
| hsa-circRNA7836-45_CBC1 | 0.00040547 | 2.036853289 |
| hsa_circ_0010974_CBC1 | 0.000487284 | 2.095205809 |
| hsa-circRNA4225-18_CBC1 | 0.012014416 | 2.35504115 |
| hsa-circRNA13111-12_CBC1 | 0.000284656 | 4.656848159 |
| hsa-circRNA15435-4_CBC1 | 0.001085759 | 2.160658803 |
| hsa_circ_0079878_CBC1 | 4.89254E-05 | 8.334384277 |
| hsa_circ_0049401_CBC1 | 4.85929E-05 | 6.326520095 |
| hsa_circ_0047936_CBC1 | 0.002863868 | 2.23989624 |
| hsa_circ_0028700_CBC1 | 0.000374396 | 4.842318106 |
| hsa_circ_0025720_CBC1 | 0.007770002 | 2.148123537 |
| hsa_circ_0006091_CBC1 | 0.002408356 | 2.087488853 |
| hsa_circ_0046673_CBC1 | 0.000455519 | 4.879647845 |
| hsa-circRNA7044-5_CBC1 | 2.70529E-05 | 7.458336655 |
| hsa-circRNA626-25_CBC1 | 0.0027749 | 2.110560455 |
| hsa_circ_0090625_CBC1 | 0.039657352 | 2.084594945 |
| hsa-circRNA5796-1_CBC1 | 0.000540874 | 6.632770984 |
| hsa_circ_0087595_CBC1 | 6.05051E-05 | 2.799307742 |
| hsa-circRNA13850-3_CBC1 | 0.003245292 | 2.85575517 |
| hsa_circ_0042119_CBC1 | 0.010726535 | 2.457384264 |
| hsa_circ_0136187_CBC1 | 0.036178689 | 3.606040749 |
| hsa_circ_0098813_CBC1 | 0.013173673 | 2.489466686 |
| hsa_circ_0037811_CBC1 | 0.000376186 | 2.930179343 |
| hsa_circ_0064961_CBC1 | 0.00065567 | 2.009935077 |
| hsa-circRNA7044-16_CBC1 | 0.000335216 | 8.32829328 |
| hsa_circ_0080979_CBC1 | 0.047104078 | 2.348051157 |
| hsa_circ_0048412_CBC1 | 0.005145146 | 4.627736131 |
| hsa_circ_0136193_CBC1 | 0.001024852 | 7.019081171 |
| hsa_circ_0024161_CBC1 | 0.002296204 | 2.379216072 |
| hsa-circRNA2520-11_CBC1 | 3.23076E-05 | 9.298733391 |
| hsa_circ_0005061_CBC1 | 1.08051E-05 | 2.007869561 |
| hsa_circ_0050807_CBC1 | 0.000222621 | 2.006338629 |
| hsa-circRNA13041-20_CBC1 | 0.013125797 | 2.288328143 |
| hsa-circRNA8621-6_CBC1 | 0.000264223 | 2.945679132 |
| hsa_circ_0032895_CBC1 | 0.005289714 | 2.797642678 |
| hsa_circ_0072694_CBC1 | 0.044802973 | 3.528190432 |
| hsa_circ_0017785_CBC1 | 0.034170054 | 3.613704674 |
| hsa_circ_0012322_CBC1 | 0.013451048 | 2.26735715 |
| hsa_circ_0114950_CBC1 | 0.019304875 | 2.020436825 |
| hsa_circ_0028690_CBC1 | 0.001404617 | 5.728986275 |
| hsa-circRNA3568-2_CBC1 | 0.000578824 | 2.335707213 |
| hsa_circ_0135661_CBC1 | 0.002263146 | 4.144357028 |
| hsa_circ_0002792_CBC1 | 0.026172652 | 2.560337122 |
| hsa-circRNA2729-2_CBC1 | 0.010096689 | 3.482678181 |
| hsa_circ_0011593_CBC1 | 0.016594896 | 2.92660121 |
| hsa_circ_0089660_CBC1 | 0.000157345 | 3.056490435 |
| hsa-circRNA12677-147_CBC1 | 1.71929E-06 | 7.270943183 |
| hsa-circRNA7257-14_CBC1 | 0.007703874 | 2.454389428 |
| hsa-circRNA4965-31_CBC1 | 3.15768E-05 | 6.676117682 |
| hsa-circRNA14994-11_CBC1 | 0.000340464 | 2.521523499 |
| hsa_circ_0095554_CBC1 | 0.001262744 | 3.210957366 |
| hsa-circRNA10691-3_CBC1 | 0.00217589 | 3.109737867 |
| hsa_circ_0098757_CBC1 | 0.014454655 | 2.309158392 |
| hsa_circ_0022086_CBC1 | 0.002973516 | 2.976848506 |
| hsa_circ_0042337_CBC1 | 0.002846204 | 2.196162248 |
| hsa_circ_0113466_CBC1 | 0.004082207 | 3.479760425 |
| hsa-circRNA14728_CBC1 | 0.004372946 | 2.845822965 |
| hsa_circ_0136184_CBC1 | 0.000403486 | 3.112727304 |
| hsa_circ_0049026_CBC1 | 0.043836013 | 2.071906978 |
| hsa-circRNA421_CBC1 | 0.003093471 | 2.288065902 |
| hsa_circ_0036913_CBC1 | 2.38642E-05 | 6.580084558 |
| hsa_circ_0018970_CBC1 | 0.000670044 | 2.170889845 |
| hsa_circ_0012167_CBC1 | 0.000105886 | 5.76929119 |
| hsa_circ_0036917_CBC1 | 2.77153E-05 | 5.609036263 |
| hsa_circ_0043546_CBC1 | 0.000705575 | 4.222236707 |
| hsa_circ_0119085_CBC1 | 0.025198178 | 2.782429907 |
| hsa_circ_0002892_CBC1 | 2.34133E-05 | 2.347928565 |
| hsa-circRNA6200-16_CBC1 | 0.000708325 | 4.456535727 |
| hsa-circRNA2033-82_CBC1 | 0.000905485 | 3.599445488 |
| hsa_circ_0139657_CBC1 | 0.006489731 | 3.393569376 |
| hsa-circRNA3977-10_CBC1 | 0.002420056 | 3.502252021 |
| hsa_circ_0068855_CBC1 | 0.000133292 | 7.933170836 |
| hsa_circ_0015831_CBC1 | 0.003697641 | 6.112578447 |
| hsa-circRNA1623-4_CBC1 | 0.007124259 | 2.431014576 |
| hsa_circ_0086139_CBC1 | 0.001324795 | 3.697227216 |
| hsa-circRNA813-2_CBC1 | 0.02103147 | 2.91043205 |
| hsa_circ_0024794_CBC1 | 0.002953766 | 2.058150356 |
| hsa_circ_0093449_CBC1 | 0.001700663 | 3.148206595 |
| hsa_circ_0095358_CBC1 | 0.009411916 | 2.530001449 |
| hsa_circ_0138111_CBC1 | 0.000181707 | 3.427523282 |
| hsa-circRNA2536-11_CBC1 | 0.000439995 | 3.359587374 |
| hsa_circ_0022814_CBC1 | 0.000745593 | 5.97775542 |
| hsa-circRNA13196-2_CBC1 | 0.045555202 | 2.175778293 |
| hsa-circRNA2033-59_CBC1 | 0.009955615 | 3.03995313 |
| hsa_circ_0041994_CBC1 | 7.3538E-07 | 6.848425941 |
| hsa_circ_0025448_CBC1 | 0.000819038 | 2.828999813 |
| hsa-circRNA1030-15_CBC1 | 0.000687398 | 11.47333763 |
| hsa-circRNA9249_CBC1 | 0.002355286 | 3.960355153 |
| hsa_circ_0075886_CBC1 | 0.001294191 | 6.168345182 |
| hsa-circRNA3015-4_CBC1 | 1.07856E-05 | 2.314548349 |
| hsa_circ_0123359_CBC1 | 0.004435266 | 4.41974138 |
| hsa_circ_0122580_CBC1 | 0.000649854 | 6.315946149 |
| hsa_circ_0065977_CBC1 | 0.002586769 | 2.791845875 |
| hsa_circ_0068025_CBC1 | 0.007860963 | 3.514455373 |
| hsa_circ_0087512_CBC1 | 0.00159047 | 5.217026693 |
| hsa_circ_0036700_CBC1 | 0.00109161 | 4.412484696 |
| hsa_circ_0079868_CBC1 | 0.001224854 | 6.372710787 |
| hsa_circ_0010971_CBC1 | 0.000504579 | 2.103157795 |
| hsa_circ_0134223_CBC1 | 0.000230593 | 6.753139481 |
| hsa_circ_0050696_CBC1 | 1.16318E-05 | 4.499072362 |
| hsa-circRNA13698-27_CBC1 | 0.001691245 | 9.354608715 |
| hsa-circRNA9154-39_CBC1 | 0.023997743 | 2.253304629 |
| hsa_circ_0059404_CBC1 | 0.042272493 | 2.254325081 |
| hsa-circRNA3767-8_CBC1 | 0.016301297 | 2.380821651 |
| hsa_circ_0043947_CBC1 | 3.40402E-05 | 3.632696357 |
| hsa_circ_0038392_CBC1 | 0.000310756 | 2.295911859 |
| hsa_circ_0131457_CBC1 | 0.000654937 | 2.223561248 |
| hsa-circRNA13998-25_CBC1 | 0.017895268 | 2.033034914 |
| hsa_circ_0002760_CBC1 | 0.001622787 | 2.198091204 |
| hsa_circ_0132370_CBC1 | 0.003576135 | 11.11897433 |
| hsa_circ_0050705_CBC1 | 5.06637E-07 | 12.79518655 |
| hsa-circRNA11493-25_CBC1 | 0.000765642 | 4.016793319 |
| hsa-circRNA7275-21_CBC1 | 0.0031017 | 2.268021683 |
| hsa-circRNA7663-2_CBC1 | 0.031084514 | 2.776214966 |
| hsa_circ_0008946_CBC1 | 0.000104788 | 7.591555472 |
| hsa_circ_0124008_CBC1 | 0.008128662 | 2.549054768 |
| hsa_circ_0019162_CBC1 | 0.004391004 | 3.619670406 |
| hsa_circ_0024105_CBC1 | 0.000637271 | 2.421995178 |
| hsa_circ_0082573_CBC1 | 0.005431297 | 2.103616602 |
| hsa_circ_0047090_CBC1 | 0.008020038 | 2.56102387 |
| hsa_circ_0022856_CBC1 | 0.038249286 | 2.141265558 |
| hsa_circ_0034303_CBC1 | 0.022946792 | 2.529182393 |
| hsa_circ_0036180_CBC1 | 0.000410925 | 2.159495579 |
| hsa_circ_0131893_CBC1 | 0.021909825 | 4.247808928 |
| hsa-circRNA8511-9_CBC1 | 0.005363983 | 4.428787354 |
| hsa_circ_0051666_CBC1 | 0.009032445 | 3.225722868 |
| hsa_circ_0017754_CBC1 | 0.001769492 | 2.266115751 |
| hsa_circ_0053382_CBC1 | 0.000379065 | 4.897108161 |
| hsa_circ_0065423_CBC1 | 4.6468E-05 | 2.219476812 |
| hsa_circ_0036000_CBC1 | 0.014828884 | 2.463603678 |
| hsa_circ_0053201_CBC1 | 0.046159854 | 2.005899481 |
| hsa_circ_0016377_CBC1 | 0.006813424 | 6.052982032 |
| hsa_circ_0061547_CBC1 | 0.004762287 | 2.33892124 |
| hsa_circ_0074784_CBC1 | 0.005285063 | 2.243975723 |
| hsa_circ_0063032_CBC1 | 0.00059263 | 3.491345139 |
| hsa_circ_0061632_CBC1 | 0.001393867 | 5.522885085 |
| hsa_circ_0017488_CBC1 | 0.000318602 | 2.095309735 |
| hsa-circRNA11242-7_CBC1 | 0.000221527 | 4.503783489 |
| hsa_circ_0098802_CBC1 | 0.020307706 | 2.424117083 |
| hsa_circ_0063522_CBC1 | 0.00055975 | 2.048803269 |
| hsa-circRNA16246-1_CBC1 | 0.026397767 | 4.4163485 |
| hsa_circ_0014747_CBC1 | 0.000569425 | 6.87581998 |
| hsa_circ_0030370_CBC1 | 0.004811506 | 3.363119007 |
| hsa-circRNA10818-3_CBC1 | 0.014637037 | 2.054392719 |
| hsa_circ_0094365_CBC1 | 0.000902587 | 3.48942292 |
| hsa_circ_0004519_CBC1 | 0.001355433 | 2.205597693 |
| hsa_circ_0059711_CBC1 | 3.25627E-05 | 8.625925006 |
| hsa_circ_0043543_CBC1 | 0.000173214 | 5.497677063 |
| hsa_circ_0076753_CBC1 | 0.006117686 | 2.793352135 |
| hsa_circ_0125329_CBC1 | 0.001569897 | 2.008359078 |
| hsa-circRNA6231_CBC1 | 0.001894722 | 3.328557594 |
| hsa-circRNA15511-9_CBC1 | 0.028266944 | 2.439866126 |
| hsa_circ_0049187_CBC1 | 0.000871212 | 2.682879826 |
| hsa-circRNA2886-8_CBC1 | 0.005625572 | 2.387424895 |
| hsa_circ_0012381_CBC1 | 0.001643038 | 3.801566719 |
| hsa_circ_0090953_CBC1 | 0.047199897 | 4.691044936 |
| hsa_circ_0027446_CBC1 | 0.008701786 | 2.411927042 |
| hsa_circ_0039236_CBC1 | 0.015434148 | 2.946686393 |
| hsa_circ_0094257_CBC1 | 0.002046631 | 2.017744345 |
| hsa-circRNA13698-28_CBC1 | 0.008077248 | 8.892071364 |
| hsa_circ_0136264_CBC1 | 0.002855547 | 2.019328899 |
| hsa_circ_0050716_CBC1 | 0.019714489 | 2.171870698 |
| hsa-circRNA8870-8_CBC1 | 0.0070718 | 2.664117819 |
| hsa_circ_0029527_CBC1 | 0.000818067 | 6.859719889 |
| hsa_circ_0060361_CBC1 | 3.00425E-06 | 10.31637937 |
| hsa-circRNA1396-4_CBC1 | 0.010553489 | 3.357384943 |
| hsa-circRNA10440-1_CBC1 | 0.002119319 | 3.125066492 |
| hsa-circRNA2033-24_CBC1 | 0.001515848 | 3.323277136 |
| hsa_circ_0082836_CBC1 | 0.010171321 | 2.145673167 |
| hsa_circ_0005352_CBC1 | 0.002355309 | 3.614581194 |
| hsa_circ_0095565_CBC1 | 0.008344927 | 2.118639564 |
| hsa_circ_0085035_CBC1 | 0.000428841 | 3.607008997 |
| hsa-circRNA10541-22_CBC1 | 0.000712234 | 3.300044162 |
| hsa_circ_0015819_CBC1 | 0.001566469 | 10.49586765 |
| hsa_circ_0041886_CBC1 | 0.007208814 | 2.1941034 |
| hsa_circ_0017789_CBC1 | 0.004475808 | 8.620547718 |
| hsa_circ_0066607_CBC1 | 0.005807712 | 3.983227684 |
| hsa_circ_0017553_CBC1 | 0.007381308 | 2.164229391 |
| hsa_circ_0039193_CBC1 | 0.000163789 | 13.09580465 |
| hsa_circ_0039460_CBC1 | 0.004881653 | 2.514137505 |
| hsa_circ_0031771_CBC1 | 0.011161229 | 4.621927141 |
| hsa_circ_0082844_CBC1 | 0.001329752 | 2.614346724 |
| hsa_circ_0004878_CBC1 | 0.014812852 | 2.347876568 |
| hsa_circ_0009847_CBC1 | 0.00369642 | 2.157657373 |
| hsa-circRNA7275-28_CBC1 | 0.021905061 | 2.097141236 |
| hsa_circ_0013389_CBC1 | 0.025114133 | 2.393054453 |
| hsa_circ_0081391_CBC1 | 0.000260829 | 2.39180913 |
| hsa_circ_0001268_CBC1 | 0.000410731 | 4.202688226 |
| hsa-circRNA4065-1_CBC1 | 0.000534554 | 2.152725646 |
| hsa_circ_0031777_CBC1 | 0.005059399 | 2.700088751 |
| hsa_circ_0106134_CBC1 | 0.001858429 | 2.029907994 |
| hsa_circ_0022804_CBC1 | 0.000547782 | 3.385966718 |
| hsa-circRNA6411-13_CBC1 | 0.007821904 | 7.114306781 |
| hsa_circ_0090623_CBC1 | 0.000954202 | 3.592605151 |
| hsa_circ_0085468_CBC1 | 0.004090537 | 5.607109107 |
| hsa_circ_0070684_CBC1 | 0.019312683 | 2.960468663 |
| hsa-circRNA12269-24_CBC1 | 9.05181E-05 | 5.871105204 |
| hsa_circ_0101385_CBC1 | 0.024120172 | 2.566396962 |
| hsa-circRNA1231-4_CBC1 | 0.000122361 | 2.169758804 |
| hsa_circ_0062265_CBC1 | 0.004028112 | 5.890093006 |
| hsa-circRNA4225-11_CBC1 | 0.000565791 | 2.92550681 |
| hsa_circ_0040694_CBC1 | 0.002944808 | 2.147267555 |
| hsa_circ_0043169_CBC1 | 0.001990188 | 2.152488339 |
| hsa_circ_0093986_CBC1 | 0.032676391 | 2.237060199 |
| hsa-circRNA1036-9_CBC1 | 0.024106976 | 2.805912331 |
| hsa_circ_0024910_CBC1 | 0.023310486 | 3.812527048 |
| hsa_circ_0008229_CBC1 | 0.000651033 | 9.734701941 |
| hsa_circ_0123912_CBC1 | 0.006212451 | 2.032970911 |
| hsa_circ_0093980_CBC1 | 0.01540484 | 2.427244072 |
| hsa_circ_0028682_CBC1 | 9.8746E-05 | 8.500775623 |
| hsa_circ_0050796_CBC1 | 0.00528296 | 2.702944534 |
| hsa-circRNA7663-16_CBC1 | 0.005431949 | 3.88491585 |
| hsa-circRNA2033-134_CBC1 | 0.001385368 | 2.396544218 |
| hsa_circ_0017551_CBC1 | 0.001895834 | 3.111409117 |
| hsa_circ_0137512_CBC1 | 0.000492287 | 6.479345119 |
| hsa-circRNA15902-1_CBC1 | 0.001106025 | 2.298304604 |
| hsa_circ_0051725_CBC1 | 0.000583662 | 7.544609396 |
| hsa_circ_0036910_CBC1 | 0.006474954 | 2.26233611 |
| hsa_circ_0036920_CBC1 | 0.000256219 | 6.267225528 |
| hsa-circRNA8054-5_CBC1 | 0.000353035 | 3.085632427 |
| hsa_circ_0021480_CBC1 | 0.002357846 | 2.119557581 |
| hsa_circ_0008843_CBC1 | 0.003388892 | 2.77781762 |
| hsa_circ_0134741_CBC1 | 0.00019059 | 2.912941299 |
| hsa_circ_0025149_CBC1 | 0.000186087 | 3.769991526 |
| hsa_circ_0049797_CBC1 | 0.000694509 | 2.31659321 |
| hsa_circ_0001592_CBC1 | 0.006164828 | 2.284020258 |
| hsa_circ_0092321_CBC1 | 0.003083805 | 6.803517995 |
| hsa-circRNA12269-6_CBC1 | 0.005666657 | 7.455835751 |
| hsa_circ_0014763_CBC1 | 0.000240627 | 3.415759867 |
| hsa-circRNA5886-34_CBC1 | 0.00022725 | 3.14659185 |
| hsa_circ_0088280_CBC1 | 0.041430982 | 2.046690406 |
| hsa_circ_0008982_CBC1 | 0.002048243 | 2.015903174 |
| hsa_circ_0062445_CBC1 | 0.047241892 | 2.087920336 |
| hsa_circ_0049218_CBC1 | 0.000668105 | 2.862420316 |
| hsa_circ_0070554_CBC1 | 0.002385814 | 5.696105871 |
| hsa_circ_0122583_CBC1 | 0.000668848 | 4.385493543 |
| hsa_circ_0058366_CBC1 | 0.008529061 | 2.110514655 |
| hsa_circ_0134384_CBC1 | 0.000958165 | 2.200462855 |
| hsa_circ_0011478_CBC1 | 0.001714036 | 2.197495168 |
| hsa-circRNA2527-8_CBC1 | 0.001841904 | 4.743185673 |
| hsa_circ_0074099_CBC1 | 0.003934027 | 8.940192299 |
| hsa_circ_0044049_CBC1 | 0.009967111 | 2.132305081 |
| hsa_circ_0136766_CBC1 | 0.012036767 | 3.950845762 |
| hsa_circ_0035488_CBC1 | 0.000668858 | 6.251698376 |
| hsa_circ_0003973_CBC1 | 0.019243605 | 2.196983582 |
| hsa_circ_0024920_CBC1 | 0.001121235 | 3.663398581 |
| hsa-circRNA9867-17_CBC1 | 0.003201413 | 2.246865971 |
| hsa_circ_0059733_CBC1 | 0.004378546 | 7.984111026 |
| hsa-circRNA2599-10_CBC1 | 0.000110346 | 4.231755331 |
| hsa-circRNA13348-4_CBC1 | 0.030566078 | 2.788732523 |
| hsa_circ_0074841_CBC1 | 0.000786697 | 7.47610196 |
| hsa_circ_0004172_CBC1 | 0.000277875 | 2.350712808 |
| hsa_circ_0070508_CBC1 | 0.048293552 | 2.55853727 |
| hsa_circ_0102383_CBC1 | 0.002183071 | 8.962722144 |
| hsa_circ_0084478_CBC1 | 0.014525647 | 2.023066238 |
| hsa_circ_0056038_CBC1 | 0.000106906 | 7.976223842 |
| hsa_circ_0109680_CBC1 | 0.006844443 | 2.084283781 |
| hsa-circRNA9910-3_CBC1 | 0.012680739 | 2.437673299 |
| hsa_circ_0106911_CBC1 | 0.001996658 | 4.813972836 |
| hsa_circ_0032903_CBC1 | 0.000154031 | 3.551834408 |
| hsa_circ_0059726_CBC1 | 3.07064E-05 | 7.386456698 |
| hsa-circRNA8518-39_CBC1 | 0.002140911 | 2.511258503 |
| hsa-circRNA1988-3_CBC1 | 0.012049948 | 2.777986607 |
| hsa_circ_0110334_CBC1 | 0.035255039 | 3.648202181 |
| hsa_circ_0071684_CBC1 | 0.000140632 | 5.649923885 |
| hsa_circ_0088314_CBC1 | 3.4767E-05 | 3.854374579 |
| hsa_circ_0041856_CBC1 | 0.002484884 | 2.202038874 |
| hsa_circ_0029088_CBC1 | 0.004952262 | 2.860686968 |
| hsa_circ_0068859_CBC1 | 0.000207866 | 3.2534472 |
| hsa_circ_0060451_CBC1 | 0.00057688 | 7.35321045 |
| hsa_circ_0133284_CBC1 | 0.005464852 | 2.000927843 |
| hsa_circ_0049223_CBC1 | 0.000584375 | 2.617585132 |
| hsa-circRNA12057-3_CBC1 | 7.31304E-05 | 2.764288963 |
| hsa-circRNA247-11_CBC1 | 6.67584E-05 | 7.465627094 |
| hsa-circRNA2329-24_CBC1 | 0.00018846 | 9.159987003 |
| hsa_circ_0083901_CBC1 | 0.004649636 | 2.276605143 |
| hsa_circ_0019166_CBC1 | 0.000215757 | 7.003648626 |
| hsa_circ_0093978_CBC1 | 0.004289216 | 3.687865794 |
| hsa-circRNA1257-26_CBC1 | 0.000839104 | 3.966979643 |
| hsa-circRNA4771-3_CBC1 | 0.005055007 | 2.064466074 |
| hsa-circRNA10466-29_CBC1 | 0.016939599 | 2.289496798 |
| hsa-circRNA13043-31_CBC1 | 0.019321931 | 4.670210219 |
| hsa_circ_0091591_CBC1 | 0.003944417 | 2.094233354 |
| hsa-circRNA1203-21_CBC1 | 0.001429522 | 2.161074338 |
| hsa_circ_0025660_CBC1 | 0.00266294 | 2.301134681 |
| hsa_circ_0097847_CBC1 | 0.000368212 | 2.871941802 |
| hsa-circRNA8518-32_CBC1 | 0.047925133 | 2.368801257 |
| hsa_circ_0013391_CBC1 | 0.015727589 | 2.895848922 |
| hsa_circ_0050717_CBC1 | 0.003562059 | 5.770628308 |
| hsa_circ_0025449_CBC1 | 0.001952596 | 4.81512189 |
| hsa_circ_0066751_CBC1 | 0.001116958 | 6.27967155 |
| hsa-circRNA14229-6_CBC1 | 0.023873005 | 3.066051145 |
| hsa_circ_0077260_CBC1 | 0.007167872 | 4.102743969 |
| hsa_circ_0123014_CBC1 | 0.006709195 | 2.287195628 |
| hsa_circ_0059414_CBC1 | 0.020191378 | 2.205882564 |
| hsa_circ_0040488_CBC1 | 0.005970612 | 2.643866796 |
| hsa-circRNA11049-11_CBC1 | 0.001347362 | 2.191007275 |
| hsa_circ_0072758_CBC1 | 1.01269E-05 | 6.248125192 |
| hsa-circRNA7663-23_CBC1 | 0.010225908 | 2.878860919 |
| hsa_circ_0039129_CBC1 | 0.002169945 | 4.044256743 |
| hsa_circ_0050704_CBC1 | 6.21474E-05 | 8.682276174 |
| hsa_circ_0025148_CBC1 | 0.000470623 | 4.078483839 |
| hsa-circRNA2329-31_CBC1 | 0.000826768 | 7.334449944 |
| hsa_circ_0067427_CBC1 | 0.000314982 | 2.676296035 |
| hsa_circ_0051718_CBC1 | 0.000187162 | 5.890041337 |
| hsa-circRNA708-1_CBC1 | 0.000110511 | 8.776044869 |
| hsa-circRNA3390-6_CBC1 | 0.009947468 | 4.628197113 |
| hsa_circ_0080972_CBC1 | 0.027038062 | 2.444074623 |
| hsa-circRNA6673_CBC1 | 0.024943431 | 5.53371754 |
| hsa_circ_0097454_CBC1 | 0.018765362 | 2.065499116 |
| hsa_circ_0076098_CBC1 | 0.005721358 | 2.207965978 |
| hsa_circ_0024727_CBC1 | 0.002189701 | 2.376305817 |
| hsa_circ_0045015_CBC1 | 0.016912819 | 2.082214524 |
| hsa_circ_0032234_CBC1 | 0.026209743 | 2.143918791 |
| hsa_circ_0036263_CBC1 | 0.014394085 | 2.757584759 |
| hsa_circ_0036705_CBC1 | 0.001821348 | 4.38578887 |
| hsa-circRNA6634-4_CBC1 | 0.005803241 | 3.492866243 |
| hsa_circ_0083735_CBC1 | 6.0067E-05 | 9.388270011 |
| hsa_circ_0107333_CBC1 | 0.001595275 | 2.48649471 |
| hsa_circ_0053002_CBC1 | 0.000160623 | 4.273212663 |
| hsa_circ_0074307_CBC1 | 0.000855084 | 2.583050759 |
| hsa_circ_0068028_CBC1 | 0.008744669 | 3.354062423 |
| hsa_circ_0049210_CBC1 | 0.000741136 | 2.853033801 |
| hsa-circRNA5427-80_CBC1 | 0.00039216 | 6.781616462 |
| hsa_circ_0063520_CBC1 | 8.81981E-05 | 2.229674295 |
| hsa-circRNA10206-52_CBC1 | 0.0002805 | 2.124819302 |
| hsa_circ_0040871_CBC1 | 0.00011053 | 2.016150841 |
| hsa_circ_0007496_CBC1 | 0.002094368 | 4.159889195 |
| hsa_circ_0124249_CBC1 | 0.020765931 | 2.13031282 |
| hsa_circ_0081476_CBC1 | 0.000306235 | 2.023981471 |
| hsa_circ_0038705_CBC1 | 0.02985341 | 2.376639037 |
| hsa_circ_0036699_CBC1 | 0.002104923 | 6.086312164 |
| hsa-circRNA12558-11_CBC1 | 0.008528091 | 2.059691062 |
| hsa_circ_0065029_CBC1 | 0.004016392 | 4.216502323 |
| hsa_circ_0002124_CBC1 | 0.001452783 | 2.133356953 |
| hsa-circRNA3792-13_CBC1 | 0.015442822 | 2.474158742 |
| hsa_circ_0080554_CBC1 | 2.2701E-05 | 2.195233437 |
| hsa_circ_0128092_CBC1 | 0.000278862 | 2.067390198 |
| hsa_circ_0026589_CBC1 | 0.001856083 | 3.648793023 |
| hsa_circ_0009107_CBC1 | 0.010064394 | 4.024540566 |
| hsa_circ_0116913_CBC1 | 0.000202168 | 7.503396869 |
| hsa-circRNA15162-60_CBC1 | 0.00114327 | 3.054772473 |
| hsa_circ_0040477_CBC1 | 0.006431939 | 4.510636151 |
| hsa_circ_0029134_CBC1 | 0.012449648 | 2.424058894 |
| hsa_circ_0022687_CBC1 | 0.00349413 | 2.20526528 |
| hsa_circ_0088956_CBC1 | 0.000946017 | 2.072935231 |
| hsa-circRNA9789-19_CBC1 | 0.003456928 | 3.942392831 |
| hsa_circ_0036922_CBC1 | 0.000134644 | 6.904259604 |
| hsa_circ_0138115_CBC1 | 0.000681445 | 3.497986363 |
| hsa_circ_0028694_CBC1 | 1.66503E-05 | 5.443846618 |
| hsa-circRNA8621-2_CBC1 | 0.001917082 | 5.224588207 |
| hsa-circRNA7393-11_CBC1 | 0.000101088 | 8.218480073 |
| hsa_circ_0140006_CBC1 | 0.002145703 | 4.001594679 |
| hsa_circ_0107705_CBC1 | 0.009829461 | 2.273975711 |
| hsa_circ_0097919_CBC1 | 5.56237E-05 | 3.938209385 |
| hsa-circRNA3279-17_CBC1 | 3.77572E-05 | 2.88250949 |
| hsa-circRNA1008-12_CBC1 | 0.000142971 | 2.347853222 |
| hsa_circ_0045598_CBC1 | 0.000216348 | 2.073742444 |
| hsa_circ_0001495_CBC1 | 0.002611085 | 3.642525662 |
| hsa_circ_0133167_CBC1 | 0.038968362 | 2.915477204 |
| hsa_circ_0065756_CBC1 | 0.000115517 | 4.464811879 |
| hsa-circRNA1649-21_CBC1 | 0.001869464 | 3.136492907 |
| hsa_circ_0036084_CBC1 | 0.000439093 | 4.179086616 |
| hsa_circ_0076744_CBC1 | 0.014970944 | 2.168135257 |
| hsa_circ_0018489_CBC1 | 0.003880698 | 2.425336566 |
| hsa_circ_0102723_CBC1 | 0.013140956 | 3.235243394 |
| hsa_circ_0081417_CBC1 | 3.36265E-06 | 3.889436316 |
| hsa-circRNA12677-33_CBC1 | 0.000400242 | 7.861147694 |
| hsa_circ_0063688_CBC1 | 0.005976143 | 2.538709192 |
| hsa-circRNA11493-20_CBC1 | 0.00769721 | 5.094636989 |
| hsa_circ_0109001_CBC1 | 0.001294892 | 2.748010647 |
| hsa_circ_0041875_CBC1 | 0.000522109 | 2.332985173 |
| hsa_circ_0041044_CBC1 | 0.004761328 | 3.215982549 |
| hsa_circ_0041062_CBC1 | 0.046232159 | 2.742777368 |
| hsa_circ_0042567_CBC1 | 3.87311E-05 | 9.54522155 |
| hsa_circ_0094367_CBC1 | 0.000867681 | 8.627871095 |
| hsa-circRNA2973-2_CBC1 | 0.001302324 | 2.049226359 |
| hsa_circ_0022808_CBC1 | 0.002043596 | 2.384658442 |
| hsa_circ_0065411_CBC1 | 0.012625213 | 2.064329629 |
| hsa-circRNA5936-2_CBC1 | 0.001631803 | 5.657669723 |
| hsa_circ_0088986_CBC1 | 0.000488695 | 2.176374712 |
| hsa_circ_0049798_CBC1 | 0.003223511 | 2.38012983 |
| hsa-circRNA1721-36_CBC1 | 0.019602548 | 2.710170877 |
| hsa_circ_0034605_CBC1 | 0.019343005 | 5.579590154 |
| hsa-circRNA13465-5_CBC1 | 6.00681E-05 | 7.135254608 |
| hsa-circRNA13111-10_CBC1 | 6.40319E-05 | 8.392799494 |
| hsa_circ_0039108_CBC1 | 0.003600933 | 2.082185851 |
| hsa-circRNA6265-7_CBC1 | 0.017556615 | 2.761232857 |
| hsa_circ_0042569_CBC1 | 2.4014E-05 | 7.672253553 |
| hsa-circRNA5806-9_CBC1 | 0.00057881 | 4.899285709 |
| hsa_circ_0070690_CBC1 | 0.019292579 | 2.92645739 |
| hsa_circ_0028696_CBC1 | 4.21884E-05 | 5.690585382 |
| hsa_circ_0016383_CBC1 | 0.003057535 | 6.460349235 |
| hsa_circ_0013659_CBC1 | 0.024306497 | 2.068464292 |
| hsa_circ_0074091_CBC1 | 1.68465E-05 | 10.765288 |
| hsa_circ_0034573_CBC1 | 0.000796402 | 11.61226141 |
| hsa_circ_0052580_CBC1 | 0.002455757 | 2.064098321 |
| hsa_circ_0032023_CBC1 | 0.000274839 | 6.820824189 |
| hsa_circ_0036899_CBC1 | 0.000393567 | 2.011448705 |
| hsa-circRNA10206-17_CBC1 | 0.00623909 | 3.918701757 |
| hsa-circRNA2741-7_CBC1 | 0.005121199 | 2.262329044 |
| hsa-circRNA2536-17_CBC1 | 0.000178254 | 7.946581729 |
| hsa_circ_0027579_CBC1 | 0.002050266 | 2.603931245 |
| hsa_circ_0029529_CBC1 | 0.000439316 | 3.131320292 |
| hsa_circ_0075801_CBC1 | 0.008179556 | 2.096084663 |
| hsa_circ_0133661_CBC1 | 0.000811016 | 2.505231134 |
| hsa-circRNA836-7_CBC1 | 0.004926599 | 2.778039329 |
| hsa-circRNA11981-29_CBC1 | 0.000659783 | 3.666557192 |
| hsa_circ_0059714_CBC1 | 0.015358376 | 5.582631091 |
| hsa_circ_0135576_CBC1 | 0.031655262 | 2.380286529 |
| hsa_circ_0007837_CBC1 | 0.040902251 | 2.168026801 |
| hsa_circ_0012520_CBC1 | 0.003915742 | 5.548322407 |
| hsa-circRNA8881-24_CBC1 | 0.000294657 | 9.968215803 |
| hsa_circ_0090301_CBC1 | 0.013861108 | 2.373062135 |
| hsa_circ_0137511_CBC1 | 0.015865786 | 3.187156995 |
| hsa_circ_0058981_CBC1 | 0.004024499 | 6.859473307 |
| hsa_circ_0105327_CBC1 | 0.043673613 | 2.115325658 |
| hsa-circRNA7663-33_CBC1 | 0.017814116 | 2.899336899 |
| hsa_circ_0090960_CBC1 | 2.13745E-05 | 6.09184901 |
| hsa_circ_0028699_CBC1 | 0.001040167 | 2.440537096 |
| hsa_circ_0088993_CBC1 | 0.00112994 | 2.013794273 |
| hsa-circRNA7836-101_CBC1 | 0.001279168 | 3.043224553 |
| hsa_circ_0043152_CBC1 | 0.004162429 | 2.209014762 |
| hsa-circRNA667-1_CBC1 | 0.026884791 | 2.682591798 |
| hsa_circ_0102406_CBC1 | 8.3964E-05 | 3.660233868 |
| hsa_circ_0074525_CBC1 | 1.7684E-05 | 2.409159365 |
| hsa_circ_0032823_CBC1 | 0.03328242 | 3.08278289 |
| hsa_circ_0050097_CBC1 | 0.004481427 | 2.036721121 |
| hsa_circ_0081658_CBC1 | 0.000528078 | 2.499363943 |
| hsa_circ_0133408_CBC1 | 0.007155345 | 2.456167511 |
| hsa_circ_0103426_CBC1 | 0.000254925 | 9.189694841 |
| hsa_circ_0015821_CBC1 | 8.37226E-05 | 13.77402949 |
| hsa_circ_0109114_CBC1 | 0.022941279 | 2.112331286 |
| hsa_circ_0081245_CBC1 | 0.025150271 | 2.381245978 |
| hsa_circ_0137831_CBC1 | 0.013655988 | 2.308300291 |
| hsa_circ_0062017_CBC1 | 0.010891462 | 3.199851704 |
| hsa-circRNA8518-43_CBC1 | 0.035895578 | 2.308893679 |
| hsa-circRNA8433-17_CBC1 | 0.010749909 | 8.472748765 |
| hsa_circ_0061637_CBC1 | 0.002126986 | 3.094677153 |
| hsa_circ_0034569_CBC1 | 0.000318777 | 6.129394768 |
| hsa-circRNA7585-1_CBC1 | 0.010838743 | 2.005156503 |
| hsa_circ_0068317_CBC1 | 0.001390385 | 2.720765864 |
| hsa_circ_0070103_CBC1 | 0.000903962 | 2.446314868 |
| hsa-circRNA11458-15_CBC1 | 0.002445742 | 10.87617989 |
| hsa_circ_0074303_CBC1 | 0.001177291 | 2.232098237 |
| hsa_circ_0115131_CBC1 | 0.023473134 | 4.124459615 |
| hsa_circ_0039708_CBC1 | 0.037955679 | 2.067334001 |
| hsa-circRNA1008-10_CBC1 | 0.004790717 | 4.596628315 |
| hsa_circ_0028698_CBC1 | 0.007957652 | 5.471363514 |
| hsa-circRNA9491-12_CBC1 | 0.00239402 | 2.617587359 |
| hsa-circRNA1733-22_CBC1 | 0.006151829 | 2.060478515 |
| hsa_circ_0111195_CBC1 | 0.03400927 | 2.262889955 |
| hsa_circ_0004156_CBC1 | 0.000135853 | 2.212991308 |
| hsa-circRNA14433-13_CBC1 | 0.009780242 | 3.133275788 |
| hsa_circ_0071584_CBC1 | 0.016727828 | 2.412781546 |
| hsa_circ_0024915_CBC1 | 0.00050565 | 3.614954092 |
| hsa_circ_0110330_CBC1 | 0.000433707 | 2.713450036 |
| hsa_circ_0029093_CBC1 | 0.006323257 | 3.931679584 |
| hsa-circRNA1021-3_CBC1 | 0.000607877 | 6.056338179 |
| hsa-circRNA4209-5_CBC1 | 0.0002087 | 2.127691377 |
| hsa_circ_0097646_CBC1 | 0.00177373 | 3.487243595 |
| hsa_circ_0041873_CBC1 | 0.000163518 | 2.180860483 |
| hsa_circ_0115126_CBC1 | 0.034187365 | 3.565489526 |
| hsa-circRNA4049-54_CBC1 | 0.014220451 | 6.884885634 |
| hsa_circ_0071682_CBC1 | 0.003157125 | 9.023142232 |
| hsa_circ_0067838_CBC1 | 0.000858315 | 4.33542247 |
| hsa-circRNA9572-2_CBC1 | 9.66844E-05 | 3.854251987 |
| hsa_circ_0002731_CBC1 | 0.013444764 | 2.929506405 |
| hsa_circ_0115276_CBC1 | 0.046709078 | 2.049752331 |
| hsa-circRNA5149-8_CBC1 | 0.003172129 | 3.467713739 |
| hsa_circ_0137834_CBC1 | 0.032462341 | 2.575800064 |
| hsa_circ_0072128_CBC1 | 0.007776087 | 2.319189383 |
| hsa_circ_0074313_CBC1 | 0.001534859 | 2.145653939 |
| hsa-circRNA10868-2_CBC1 | 0.039151128 | 2.012291883 |
| hsa-circRNA8500-45_CBC1 | 0.001651589 | 3.802082574 |
| hsa_circ_0026184_CBC1 | 0.001428502 | 4.383162 |
| hsa-circRNA3795-9_CBC1 | 0.005290854 | 4.983527619 |
| hsa_circ_0041045_CBC1 | 0.00584438 | 3.090702861 |
| hsa_circ_0000675_CBC1 | 0.002214875 | 2.190096896 |
| hsa_circ_0106816_CBC1 | 0.00023704 | 3.098103485 |
| hsa_circ_0045417_CBC1 | 0.033463444 | 4.004746214 |
| hsa_circ_0058400_CBC1 | 0.010670297 | 2.69469857 |
| hsa_circ_0112733_CBC1 | 0.021885079 | 3.254933804 |
| hsa_circ_0115169_CBC1 | 0.000305334 | 2.540561834 |
| hsa_circ_0024919_CBC1 | 0.002608903 | 5.282764428 |
| hsa_circ_0015905_CBC1 | 0.003692398 | 2.057017442 |
| hsa_circ_0082496_CBC1 | 0.001220851 | 2.278141216 |
| hsa_circ_0079870_CBC1 | 0.000187905 | 5.497137851 |
| hsa_circ_0095895_CBC1 | 0.008907279 | 2.165065265 |
| hsa_circ_0108998_CBC1 | 0.002583287 | 2.81179715 |
| hsa-circRNA5658-1_CBC1 | 0.040986437 | 2.83644107 |
| hsa-circRNA5213-3_CBC1 | 0.000868693 | 8.584407751 |
| hsa-circRNA4307-1_CBC1 | 0.000517047 | 2.206606123 |
| hsa_circ_0038926_CBC1 | 7.92253E-05 | 5.815619548 |
| hsa_circ_0079036_CBC1 | 0.002237599 | 2.231343863 |
| hsa_circ_0076757_CBC1 | 0.036795126 | 2.270245282 |
| hsa_circ_0050718_CBC1 | 0.001173116 | 3.209655297 |
| hsa-circRNA8296-4_CBC1 | 0.001868702 | 2.586116148 |
| hsa-circRNA11493-15_CBC1 | 0.013036508 | 2.296745311 |
| hsa-circRNA9867-118_CBC1 | 0.019300467 | 2.428205395 |
| hsa-circRNA9146-1_CBC1 | 0.009674295 | 2.646503637 |
| hsa-circRNA626-2_CBC1 | 0.004749605 | 2.658958928 |
| hsa_circ_0102386_CBC1 | 0.004973027 | 2.925605842 |
| hsa_circ_0051715_CBC1 | 4.8366E-06 | 2.877034988 |
| hsa_circ_0088958_CBC1 | 0.001586863 | 2.864708954 |
| hsa_circ_0028686_CBC1 | 5.48737E-05 | 6.127224565 |
| hsa_circ_0125625_CBC1 | 0.004638371 | 3.377861599 |
| hsa_circ_0045419_CBC1 | 0.000207482 | 2.291620858 |
| hsa_circ_0042104_CBC1 | 0.00107078 | 3.488757773 |
| hsa_circ_0137776_CBC1 | 0.014416926 | 2.574898922 |
| hsa_circ_0088970_CBC1 | 0.000229117 | 2.068296952 |
| hsa_circ_0069018_CBC1 | 0.034407786 | 2.167655927 |
| hsa_circ_0051716_CBC1 | 1.59107E-05 | 4.757008874 |
| hsa_circ_0012615_CBC1 | 0.003511848 | 2.108991634 |
| hsa-circRNA7257-15_CBC1 | 0.014128379 | 2.563457782 |
| hsa_circ_0043952_CBC1 | 0.000182893 | 6.275571261 |
| hsa_circ_0063163_CBC1 | 0.000699409 | 3.151738547 |
| hsa-circRNA5149-21_CBC1 | 0.002575599 | 3.424400022 |
| hsa_circ_0093348_CBC1 | 0.00201297 | 2.181032063 |
| hsa_circ_0136453_CBC1 | 0.002564972 | 2.460055099 |
| hsa_circ_0003299_CBC1 | 0.030923223 | 4.140055164 |
| hsa_circ_0002931_CBC1 | 0.017846007 | 2.022686838 |
| hsa_circ_0025153_CBC1 | 7.0428E-05 | 2.849461031 |
| hsa_circ_0028678_CBC1 | 2.18662E-05 | 7.583581772 |
| hsa-circRNA13043-24_CBC1 | 0.036207913 | 3.616019063 |
| hsa_circ_0012377_CBC1 | 0.000902533 | 3.785810241 |
| hsa_circ_0120417_CBC1 | 0.005488148 | 2.508331528 |
| hsa_circ_0087037_CBC1 | 0.004429347 | 3.116935894 |
| hsa_circ_0032158_CBC1 | 0.000538394 | 3.802579536 |
| hsa_circ_0068868_CBC1 | 0.000525695 | 7.443913479 |
| hsa_circ_0122732_CBC1 | 0.000902611 | 2.52275918 |
| hsa-circRNA7400-2_CBC1 | 0.046164698 | 2.128283819 |
| hsa-circRNA15906-8_CBC1 | 4.15391E-06 | 2.934719513 |
| hsa_circ_0027923_CBC1 | 0.001012948 | 3.496793544 |
| hsa_circ_0047924_CBC1 | 0.001367682 | 3.747827752 |
| hsa_circ_0093454_CBC1 | 0.001944113 | 2.832809507 |
| hsa_circ_0038932_CBC1 | 5.55201E-05 | 3.821461812 |
| hsa_circ_0028695_CBC1 | 6.15064E-05 | 6.918672957 |
| hsa_circ_0035398_CBC1 | 0.000561696 | 2.203146149 |
| hsa-circRNA2536-23_CBC1 | 0.000413421 | 6.249091996 |
| hsa-circRNA11793-3_CBC1 | 1.11718E-05 | 4.542828636 |
| hsa-circRNA1089-3_CBC1 | 0.000190709 | 3.833031145 |
| hsa_circ_0016379_CBC1 | 0.002932717 | 5.78151724 |
| hsa-circRNA9443-16_CBC1 | 0.000455961 | 7.131761511 |
| hsa-circRNA8092-25_CBC1 | 0.001722965 | 5.816598004 |
| hsa-circRNA15389-4_CBC1 | 0.002260832 | 7.473697421 |
| hsa_circ_0045021_CBC1 | 0.002432751 | 2.391328469 |
| hsa_circ_0101916_CBC1 | 0.010783951 | 3.841864893 |
| hsa_circ_0074844_CBC1 | 0.000231029 | 4.415661013 |
| hsa_circ_0065978_CBC1 | 0.020507073 | 5.71369605 |
| hsa-circRNA2049-4_CBC1 | 0.049075509 | 3.014801405 |
| hsa_circ_0029538_CBC1 | 0.005039308 | 3.461842112 |
| hsa_circ_0043656_CBC1 | 0.013157406 | 2.192376934 |
| hsa_circ_0041004_CBC1 | 0.000210981 | 2.761135362 |
| hsa-circRNA946-14_CBC1 | 0.001577794 | 2.424875251 |
| hsa_circ_0072692_CBC1 | 0.03145875 | 2.878530442 |
| hsa_circ_0040478_CBC1 | 0.01396966 | 3.315602524 |
| hsa-circRNA14309-3_CBC1 | 0.001388512 | 4.009897301 |
| hsa_circ_0102159_CBC1 | 0.01358251 | 2.563373424 |
| hsa_circ_0044125_CBC1 | 1.1578E-05 | 7.930220855 |
| hsa_circ_0045484_CBC1 | 2.28877E-05 | 2.86582108 |
| hsa_circ_0066752_CBC1 | 0.001459588 | 7.635925262 |
| hsa-circRNA5718-7_CBC1 | 0.010995084 | 2.821331453 |
| hsa_circ_0102382_CBC1 | 0.00188324 | 9.325986708 |
| hsa_circ_0039017_CBC1 | 0.03903325 | 2.445304428 |
| hsa_circ_0069551_CBC1 | 0.002196473 | 5.101886244 |
| hsa-circRNA5654-3_CBC1 | 0.007187202 | 2.134461 |
| hsa-circRNA9491-17_CBC1 | 2.44339E-05 | 2.043101213 |
| hsa-circRNA3215-45_CBC1 | 0.000130254 | 2.18055042 |
| hsa_circ_0059707_CBC1 | 0.015562722 | 2.135093619 |
| hsa_circ_0032024_CBC1 | 4.19615E-05 | 10.10246276 |
| hsa-circRNA708-2_CBC1 | 0.000925342 | 6.712533942 |
| hsa-circRNA1396-20_CBC1 | 0.003632419 | 3.02827651 |
| hsa_circ_0138108_CBC1 | 0.001066045 | 2.024972779 |
| hsa-circRNA11242-97_CBC1 | 0.027709302 | 2.921868808 |
| hsa-circRNA9660-7_CBC1 | 0.045323823 | 3.133130411 |
| hsa_circ_0086832_CBC1 | 0.000158677 | 6.609458574 |
| hsa_circ_0061218_CBC1 | 0.02992355 | 2.729672189 |
| hsa_circ_0025260_CBC1 | 0.001909444 | 2.313030485 |
| hsa_circ_0038192_CBC1 | 0.007056061 | 2.247597871 |
| hsa-circRNA3231-2_CBC1 | 2.1243E-05 | 2.522716753 |
| hsa_circ_0013305_CBC1 | 0.030445059 | 2.996386945 |
| hsa_circ_0032937_CBC1 | 0.011171958 | 3.613232393 |
| hsa_circ_0088932_CBC1 | 0.000710779 | 2.061908049 |
| hsa_circ_0036923_CBC1 | 0.000162309 | 5.991481839 |
| hsa_circ_0020492_CBC1 | 0.000376878 | 23.12343975 |
| hsa_circ_0065031_CBC1 | 0.000634728 | 3.06662307 |
| hsa-circRNA1772-8_CBC1 | 0.002404558 | 2.481272558 |
| hsa-circRNA8362-1_CBC1 | 0.000911263 | 3.042042146 |
| hsa_circ_0060318_CBC1 | 0.000738097 | 2.723359705 |
| hsa-circRNA6452_CBC1 | 2.56415E-06 | 13.44408359 |
| hsa_circ_0008471_CBC1 | 0.031129296 | 3.959692948 |
| hsa_circ_0139456_CBC1 | 0.002441601 | 7.231322027 |
| hsa_circ_0040615_CBC1 | 0.001632194 | 3.271996749 |
| hsa-circRNA11433-11_CBC1 | 0.000587373 | 2.039448758 |
| hsa-circRNA3880-4_CBC1 | 0.000143607 | 3.426869242 |
| hsa_circ_0006367_CBC1 | 0.01260226 | 4.578777812 |
| hsa_circ_0066935_CBC1 | 0.002282451 | 8.262773829 |
| hsa-circRNA15781-16_CBC1 | 0.003112674 | 2.009110661 |
| hsa-circRNA743-6_CBC1 | 0.000648645 | 4.830983365 |
| hsa_circ_0068115_CBC1 | 0.047697278 | 2.04831224 |
| hsa-circRNA8518-14_CBC1 | 0.00653166 | 2.1184079 |
| hsa_circ_0102359_CBC1 | 0.023674279 | 3.581642653 |
| hsa-circRNA2520-15_CBC1 | 0.00079845 | 7.887719127 |
| hsa-circRNA2639-10_CBC1 | 0.00641705 | 3.301367936 |
| hsa-circRNA5308-13_CBC1 | 1.15176E-05 | 4.206113897 |
| hsa_circ_0029533_CBC1 | 0.000953662 | 2.596913045 |
| hsa_circ_0084485_CBC1 | 0.041886115 | 3.369251486 |
| hsa-circRNA2719-34_CBC1 | 0.000559991 | 3.981210196 |
| hsa-circRNA14781-24_CBC1 | 0.010845604 | 3.040326045 |
| hsa_circ_0050633_CBC1 | 0.025585489 | 2.113659258 |
| hsa-circRNA14439-4_CBC1 | 0.004749855 | 12.25029112 |
| hsa_circ_0094304_CBC1 | 0.001240255 | 3.028577387 |
| hsa_circ_0100051_CBC1 | 0.006715744 | 5.08683221 |
| hsa-circRNA13528-9_CBC1 | 0.002764974 | 2.686390764 |
| hsa_circ_0012245_CBC1 | 0.028202036 | 2.005325646 |
| hsa-circRNA13990-41_CBC1 | 0.010021285 | 2.210218528 |
| hsa_circ_0028684_CBC1 | 5.40954E-05 | 8.316936988 |
| hsa_circ_0066701_CBC1 | 0.028311725 | 2.888562176 |
| hsa_circ_0048410_CBC1 | 0.000359674 | 2.08117243 |
| hsa_circ_0095562_CBC1 | 0.000647243 | 2.467079124 |
| hsa_circ_0036055_CBC1 | 0.005196198 | 2.056860798 |
| hsa_circ_0134644_CBC1 | 0.005178983 | 2.131027048 |
| hsa_circ_0120412_CBC1 | 0.00891361 | 2.61814921 |
| hsa_circ_0039103_CBC1 | 0.000630341 | 3.341905903 |
| hsa-circRNA16274-11_CBC1 | 0.009112717 | 3.46913212 |
| hsa-circRNA7044-14_CBC1 | 9.10477E-05 | 9.082617502 |
| hsa-circRNA15486-291_CBC1 | 0.022739562 | 2.160464476 |
| hsa_circ_0088929_CBC1 | 0.001107943 | 2.943652765 |
| hsa_circ_0066942_CBC1 | 0.010028737 | 4.963321315 |
| hsa_circ_0069665_CBC1 | 0.007238679 | 2.596713107 |
| hsa_circ_0084472_CBC1 | 0.007997647 | 2.350233108 |
| hsa-circRNA14257-19_CBC1 | 0.000222742 | 2.516534443 |
| hsa-circRNA6436-4_CBC1 | 0.006648812 | 2.26092963 |
| hsa-circRNA10312-22_CBC1 | 0.000896508 | 3.550345934 |
| hsa_circ_0081187_CBC1 | 0.039297955 | 2.437829528 |
| hsa_circ_0028676_CBC1 | 2.27338E-05 | 6.1195625 |
| hsa_circ_0080549_CBC1 | 0.000701689 | 2.402996014 |
| hsa-circRNA1291-16_CBC1 | 0.028509901 | 2.354469255 |
| hsa_circ_0068891_CBC1 | 0.002914266 | 2.26091219 |
| hsa_circ_0042571_CBC1 | 0.000236101 | 7.38781396 |
| hsa_circ_0018039_CBC1 | 0.007182565 | 2.278864451 |
| hsa_circ_0018032_CBC1 | 0.001171333 | 4.080561173 |
| hsa_circ_0015829_CBC1 | 0.003238693 | 11.45560762 |
| hsa_circ_0089001_CBC1 | 0.007198857 | 3.130236352 |
| hsa_circ_0012610_CBC1 | 0.006246706 | 2.204619222 |
| hsa-circRNA9789-13_CBC1 | 0.000676017 | 3.571497639 |
| hsa-circRNA5886-39_CBC1 | 0.001307973 | 2.296087822 |
| hsa-circRNA5806-24_CBC1 | 0.019432132 | 2.866061711 |
| hsa-circRNA13359-31_CBC1 | 0.001015192 | 2.301030936 |
| hsa_circ_0101892_CBC1 | 0.002284635 | 2.4433203 |
| hsa-circRNA1772-7_CBC1 | 0.008433229 | 2.099887832 |
| hsa-circRNA5427-42_CBC1 | 0.000137085 | 5.372078512 |
| hsa_circ_0012522_CBC1 | 0.0016543 | 8.032403302 |
| hsa-circRNA3215-13_CBC1 | 9.0283E-05 | 2.309660812 |
| hsa-circRNA1996-1_CBC1 | 0.002429422 | 2.390579176 |
| hsa-circRNA2719-11_CBC1 | 0.000208445 | 4.174387433 |
| hsa-circRNA2155-1_CBC1 | 0.005619452 | 5.570058029 |
| hsa_circ_0076246_CBC1 | 0.002024 | 3.571555051 |
| hsa_circ_0102229_CBC1 | 0.001695381 | 2.242029214 |
| hsa_circ_0029507_CBC1 | 0.036047345 | 2.701704386 |
| hsa-circRNA1994-7_CBC1 | 0.001317967 | 2.551353088 |
| hsa_circ_0076740_CBC1 | 0.008184519 | 2.715629053 |
| hsa_circ_0015977_CBC1 | 0.01787839 | 3.1139285 |
| hsa_circ_0053202_CBC1 | 0.012925958 | 2.7445549 |
| hsa_circ_0050794_CBC1 | 0.010987913 | 2.116171412 |
| hsa_circ_0040739_CBC1 | 0.014504835 | 5.559566943 |
| hsa_circ_0095891_CBC1 | 0.015496285 | 2.216500616 |
| hsa_circ_0064218_CBC1 | 0.001497009 | 6.570252916 |
| hsa-circRNA5831-24_CBC1 | 0.000291336 | 2.195158165 |
| hsa_circ_0069282_CBC1 | 0.014853381 | 6.254944837 |
| hsa-circRNA5535-1_CBC1 | 0.000792783 | 4.264249205 |
| hsa_circ_0079871_CBC1 | 0.000284363 | 6.390432207 |
| hsa_circ_0049611_CBC1 | 0.000536412 | 2.349027572 |
| hsa_circ_0090629_CBC1 | 0.000371228 | 3.200490591 |
| hsa-circRNA6777-7_CBC1 | 0.001393893 | 3.226552695 |
| hsa-circRNA9929-31_CBC1 | 0.000379412 | 5.202031957 |
| hsa_circ_0115134_CBC1 | 0.010163541 | 4.238728718 |
| hsa_circ_0051980_CBC1 | 0.030099879 | 2.259580657 |
| hsa_circ_0093984_CBC1 | 0.035783734 | 2.924639179 |
| hsa-circRNA13041-4_CBC1 | 0.008410405 | 4.105594594 |
| hsa_circ_0032200_CBC1 | 0.012905201 | 2.125203165 |
| hsa_circ_0111623_CBC1 | 0.000732371 | 7.096930385 |
| hsa_circ_0064009_CBC1 | 7.72857E-06 | 2.251782684 |
| hsa_circ_0138110_CBC1 | 0.003493319 | 2.148419722 |
| hsa_circ_0139037_CBC1 | 0.006023769 | 2.734881575 |
| hsa_circ_0011005_CBC1 | 0.000714726 | 2.301103874 |
| hsa_circ_0104375_CBC1 | 0.005593681 | 5.350495854 |
| hsa-circRNA14645-7_CBC1 | 0.000808663 | 2.279831703 |
| hsa_circ_0029098_CBC1 | 0.000618106 | 4.174334318 |
| hsa-circRNA11521-9_CBC1 | 0.001258235 | 6.552181654 |
| hsa-circRNA2329-17_CBC1 | 0.000529972 | 3.765384584 |
| hsa_circ_0076751_CBC1 | 0.00418557 | 3.374169215 |
| hsa_circ_0088268_CBC1 | 0.017527968 | 2.256729786 |
| hsa_circ_0105093_CBC1 | 0.000881095 | 2.728496053 |
| hsa_circ_0078978_CBC1 | 0.001069495 | 2.530248264 |
| hsa-circRNA3512-8_CBC1 | 0.009633548 | 2.195866368 |
| hsa_circ_0021728_CBC1 | 0.012715684 | 2.162564294 |
| hsa_circ_0035491_CBC1 | 0.000252582 | 5.81041376 |
| hsa-circRNA7402-2_CBC1 | 0.000462689 | 7.020997705 |
| hsa-circRNA1039_CBC1 | 0.036772982 | 3.350085313 |
| hsa-circRNA12677-110_CBC1 | 0.000267306 | 6.977089798 |
| hsa_circ_0076742_CBC1 | 0.013139578 | 2.557032175 |
| hsa_circ_0064258_CBC1 | 0.006849912 | 6.876154158 |
| hsa_circ_0020949_CBC1 | 0.001271892 | 2.478817616 |
| hsa-circRNA9789-4_CBC1 | 0.000658014 | 5.359016437 |
| hsa-circRNA12516-19_CBC1 | 0.001394141 | 2.376608472 |
| hsa_circ_0036707_CBC1 | 0.000293012 | 4.683716341 |
| hsa_circ_0060467_CBC1 | 0.000301296 | 8.882805302 |
| hsa_circ_0028703_CBC1 | 0.000481543 | 9.665911279 |
| hsa-circRNA1034-2_CBC1 | 0.00206244 | 6.943083032 |
| hsa_circ_0065396_CBC1 | 0.003892369 | 2.32103392 |
| hsa-circRNA7970-55_CBC1 | 0.006030816 | 2.673411566 |
| hsa-circRNA10149-32_CBC1 | 1.74625E-05 | 5.706483777 |
| hsa_circ_0042568_CBC1 | 1.36167E-05 | 7.678822237 |
| hsa_circ_0024953_CBC1 | 0.004561284 | 5.018009794 |
| hsa_circ_0026189_CBC1 | 0.001570896 | 3.918704466 |
| hsa_circ_0139034_CBC1 | 0.04733675 | 2.348217744 |
| hsa-circRNA7663-1_CBC1 | 0.001074552 | 3.403505926 |
| hsa_circ_0045604_CBC1 | 0.000748664 | 2.246716208 |
| hsa-circRNA6107-18_CBC1 | 0.020485706 | 2.519427742 |
| hsa_circ_0123161_CBC1 | 0.027018076 | 2.052097226 |
| hsa_circ_0088981_CBC1 | 0.001785437 | 2.200713273 |
| hsa_circ_0139910_CBC1 | 0.006704035 | 3.07587953 |
| hsa_circ_0093827_CBC1 | 0.000123285 | 6.199928793 |
| hsa_circ_0049675_CBC1 | 4.41203E-05 | 4.768461771 |
| hsa_circ_0028692_CBC1 | 1.42348E-05 | 7.892828793 |
| hsa_circ_0049191_CBC1 | 0.000301134 | 2.881300254 |
| hsa_circ_0062568_CBC1 | 0.009208915 | 2.167903611 |
| hsa_circ_0098735_CBC1 | 0.012747655 | 2.21890339 |
| hsa_circ_0032163_CBC1 | 0.014882744 | 7.45546814 |
| hsa-circRNA11773-18_CBC1 | 0.001802043 | 2.326839116 |
| hsa-circRNA12580-4_CBC1 | 0.006206431 | 3.04295799 |
| hsa-circRNA4965-36_CBC1 | 0.00016225 | 2.729606162 |
| hsa-circRNA2719-18_CBC1 | 0.006196079 | 3.760478833 |
| hsa-circRNA5332_CBC1 | 8.73302E-05 | 10.09736259 |
| hsa-circRNA10466-35_CBC1 | 0.012337756 | 2.143756536 |
| hsa_circ_0024916_CBC1 | 0.000136253 | 4.153035629 |
| hsa_circ_0135004_CBC1 | 0.023041081 | 2.314445658 |
| hsa-circRNA2329-26_CBC1 | 0.001365816 | 3.859526095 |
| hsa-circRNA2329-8_CBC1 | 0.001361083 | 2.570793077 |
| hsa_circ_0119191_CBC1 | 7.64468E-05 | 2.558839407 |
| hsa-circRNA5076-3_CBC1 | 0.03011143 | 2.040500388 |
| hsa_circ_0052810_CBC1 | 0.008151552 | 3.96263697 |
| hsa_circ_0087032_CBC1 | 0.001185025 | 4.327976371 |
| hsa_circ_0094482_CBC1 | 0.015306373 | 4.943440826 |
| hsa_circ_0080956_CBC1 | 0.018069573 | 2.077272732 |
| hsa-circRNA4547-1_CBC1 | 0.000194249 | 10.29534909 |
| hsa_circ_0048605_CBC1 | 0.001358844 | 2.479235008 |
| hsa_circ_0006898_CBC1 | 0.001290092 | 2.96896239 |
| hsa-circRNA2527-3_CBC1 | 0.000924922 | 8.15799367 |
| hsa_circ_0049203_CBC1 | 0.00032564 | 4.304602564 |
| hsa-circRNA13851-1_CBC1 | 0.004484758 | 3.584030866 |
| hsa_circ_0021540_CBC1 | 0.002211794 | 6.462472585 |
| hsa-circRNA15725-58_CBC1 | 0.002479485 | 2.036959484 |
| hsa-circRNA277-1_CBC1 | 0.04421848 | 4.312668607 |
| hsa_circ_0040480_CBC1 | 0.000898104 | 2.747912886 |
| hsa_circ_0034692_CBC1 | 9.27958E-05 | 4.823199814 |
| hsa_circ_0066141_CBC1 | 0.008101556 | 3.202343386 |
| hsa_circ_0038930_CBC1 | 6.05776E-05 | 5.138840478 |
| hsa-circRNA2536-2_CBC1 | 0.002462025 | 3.824725331 |
| hsa-circRNA743-2_CBC1 | 0.000587035 | 6.199083187 |
| hsa-circRNA15162-5_CBC1 | 0.000971317 | 2.15157765 |
| hsa_circ_0084479_CBC1 | 0.001883924 | 4.239383674 |
| hsa_circ_0111659_CBC1 | 0.001242692 | 17.66371213 |
| hsa_circ_0082506_CBC1 | 0.001832845 | 2.492321409 |
| hsa-circRNA8500-13_CBC1 | 0.003031782 | 6.404872898 |
| hsa-circRNA2639-15_CBC1 | 0.005654833 | 3.511890817 |
| hsa_circ_0040613_CBC1 | 0.042776319 | 2.380377758 |
| hsa_circ_0000432_CBC1 | 0.014339092 | 3.327556314 |
| hsa_circ_0034304_CBC1 | 0.004729057 | 6.89921218 |
| hsa_circ_0015828_CBC1 | 0.000220854 | 11.10279332 |
| hsa-circRNA7601-23_CBC1 | 0.000441299 | 2.283687366 |
| hsa-circRNA5149-24_CBC1 | 0.008459222 | 5.024223347 |
| hsa_circ_0031036_CBC1 | 0.00242667 | 2.043824287 |
| hsa-circRNA8092-2_CBC1 | 0.003710504 | 7.042217947 |
| hsa_circ_0005439_CBC1 | 0.004381127 | 5.621681219 |
| hsa-circRNA9491-3_CBC1 | 0.000808406 | 2.089212007 |
| hsa-circRNA11180-39_CBC1 | 0.000111259 | 3.257645669 |
| hsa-circRNA5427-4_CBC1 | 0.001396827 | 5.251840036 |
| hsa_circ_0086014_CBC1 | 0.003368861 | 2.016211678 |
| hsa_circ_0032170_CBC1 | 9.73454E-05 | 3.246987869 |
| hsa-circRNA7970-22_CBC1 | 0.016359558 | 2.307226004 |
| hsa_circ_0029123_CBC1 | 0.001948409 | 2.50095797 |
| hsa_circ_0113635_CBC1 | 0.001479114 | 2.16852804 |
| hsa-circRNA6968-5_CBC1 | 0.001066684 | 3.706430104 |
| hsa-circRNA9491-1_CBC1 | 0.002054315 | 2.193243828 |
| hsa-circRNA15229-9_CBC1 | 0.000833786 | 2.808267144 |
| hsa_circ_0072765_CBC1 | 0.000807758 | 6.21390766 |
| hsa-circRNA3353-3_CBC1 | 0.03320517 | 3.453367841 |
| hsa_circ_0127797_CBC1 | 0.000593001 | 7.438841236 |
| hsa_circ_0038191_CBC1 | 0.007856977 | 2.740389009 |
| hsa_circ_0041001_CBC1 | 0.000525669 | 4.267722848 |
| hsa-circRNA2916-3_CBC1 | 2.27333E-05 | 2.687823734 |
| hsa-circRNA11521-16_CBC1 | 0.000139045 | 7.447658004 |
| hsa-circRNA3390-5_CBC1 | 0.000306746 | 2.319374659 |
| hsa-circRNA3458-3_CBC1 | 0.000111548 | 2.280089413 |
| hsa_circ_0020209_CBC1 | 0.000830016 | 3.378339564 |
| hsa_circ_0032093_CBC1 | 0.007738524 | 2.368290307 |
| hsa-circRNA5149-14_CBC1 | 0.002065 | 3.190796725 |
| hsa_circ_0079099_CBC1 | 0.000295028 | 2.235416966 |
| hsa_circ_0006361_CBC1 | 0.046590581 | 2.695699976 |
| hsa_circ_0001893_CBC1 | 0.000464397 | 2.908414718 |
| hsa_circ_0071677_CBC1 | 5.88175E-05 | 5.457262323 |
| hsa_circ_0050684_CBC1 | 0.034673133 | 2.544852861 |
| hsa_circ_0060466_CBC1 | 5.74722E-05 | 5.304297903 |
| hsa_circ_0039341_CBC1 | 0.004868635 | 2.013349503 |
| hsa_circ_0058261_CBC1 | 0.019221707 | 2.254950219 |
| hsa-circRNA1988-2_CBC1 | 0.007023038 | 3.028310846 |
| hsa-circRNA14309-8_CBC1 | 0.000798967 | 6.295457963 |
| hsa_circ_0010023_CBC1 | 0.020996366 | 2.854750517 |
| hsa_circ_0029086_CBC1 | 0.022247648 | 2.531175518 |
| hsa_circ_0098767_CBC1 | 0.000381968 | 2.808668213 |
| hsa_circ_0059675_CBC1 | 0.006608041 | 3.037702859 |
| hsa-circRNA8885-15_CBC1 | 0.000692522 | 9.036761599 |
| hsa-circRNA10494_CBC1 | 0.028089412 | 3.189330483 |
| hsa_circ_0021496_CBC1 | 0.000755653 | 4.256332275 |
| hsa_circ_0109095_CBC1 | 5.6152E-05 | 2.539529228 |
| hsa_circ_0072574_CBC1 | 0.003931571 | 2.138950992 |
| hsa_circ_0016381_CBC1 | 0.005561913 | 5.305708403 |
| hsa-circRNA2719-39_CBC1 | 0.004217944 | 3.383515768 |
| hsa_circ_0015174_CBC1 | 0.015985528 | 2.13864544 |
| hsa_circ_0087030_CBC1 | 0.000857056 | 2.242657864 |
| hsa_circ_0011103_CBC1 | 0.02133642 | 3.106392435 |
| hsa_circ_0025663_CBC1 | 0.000871687 | 2.113813698 |
| hsa_circ_0002952_CBC1 | 5.3506E-05 | 4.847482866 |
| hsa_circ_0135613_CBC1 | 0.008477537 | 6.083808242 |
| hsa_circ_0029517_CBC1 | 0.001254322 | 3.461878763 |
| hsa_circ_0004163_CBC1 | 1.5048E-05 | 5.300276901 |
| hsa-circRNA7777-10_CBC1 | 0.012671226 | 3.319925222 |
| hsa_circ_0079470_CBC1 | 0.012024187 | 2.363667901 |
| hsa_circ_0063040_CBC1 | 0.006042235 | 6.199033723 |
| hsa_circ_0002324_CBC1 | 0.019963411 | 2.026695247 |
| hsa-circRNA11396-97_CBC1 | 0.01053951 | 2.024450292 |
| hsa_circ_0018524_CBC1 | 0.001339086 | 2.850040931 |
| hsa_circ_0137505_CBC1 | 0.002578822 | 2.408608599 |
| hsa-circRNA8500-17_CBC1 | 0.009106145 | 4.876953527 |
| hsa_circ_0003165_CBC1 | 0.037197848 | 2.137011231 |
| hsa_circ_0076153_CBC1 | 0.001592088 | 2.507022246 |
| hsa_circ_0105879_CBC1 | 0.020635235 | 2.012111404 |
| hsa-circRNA1437_CBC1 | 0.036313341 | 2.631549935 |
| hsa-circRNA8518-10_CBC1 | 0.011609513 | 2.238130999 |
| hsa_circ_0111664_CBC1 | 0.001289738 | 5.252538689 |
| hsa-circRNA1128-5_CBC1 | 0.001619767 | 2.56251746 |
| hsa_circ_0025152_CBC1 | 0.000281523 | 2.719559102 |
| hsa-circRNA6178-13_CBC1 | 0.011680038 | 2.104555024 |
| hsa-circRNA2520-17_CBC1 | 0.000194683 | 7.201075777 |
| hsa_circ_0032893_CBC1 | 0.000373194 | 2.446112311 |
| hsa-circRNA11332-4_CBC1 | 0.002517058 | 2.821769138 |
| hsa_circ_0049792_CBC1 | 0.024278357 | 2.779915448 |
| hsa-circRNA9158-13_CBC1 | 0.025673473 | 2.454594294 |
| hsa_circ_0077410_CBC1 | 0.022408362 | 3.754802956 |
| hsa_circ_0090165_CBC1 | 0.010901047 | 2.51795066 |
| hsa_circ_0097848_CBC1 | 0.001724637 | 2.586989282 |
| hsa_circ_0090158_CBC1 | 0.005135632 | 3.195609802 |
| hsa_circ_0121833_CBC1 | 0.002721373 | 2.507790955 |
| hsa_circ_0028449_CBC1 | 0.032960776 | 2.24541533 |
| hsa_circ_0026323_CBC1 | 9.8686E-05 | 2.61760082 |
| hsa_circ_0082514_CBC1 | 0.033284538 | 2.052591932 |
| hsa_circ_0027111_CBC1 | 0.03800056 | 3.617700608 |
| hsa_circ_0115788_CBC1 | 0.036183656 | 2.361553312 |
| hsa_circ_0064268_CBC1 | 0.000885614 | 4.050833443 |
| hsa-circRNA5213-18_CBC1 | 0.001678921 | 4.552509245 |
| hsa_circ_0032308_CBC1 | 0.017758744 | 2.04839132 |
| hsa_circ_0080942_CBC1 | 0.03756437 | 2.787046005 |
| hsa_circ_0045805_CBC1 | 0.001525246 | 2.999225882 |
| hsa-circRNA9146-2_CBC1 | 0.005710591 | 2.026570606 |
| hsa_circ_0085469_CBC1 | 0.00279635 | 3.932966791 |
| hsa_circ_0069009_CBC1 | 0.01104533 | 2.183382687 |
| hsa_circ_0093974_CBC1 | 0.039476563 | 2.326878816 |
| hsa_circ_0026612_CBC1 | 0.002257568 | 2.530419348 |
| hsa-circRNA5059-43_CBC1 | 0.00787358 | 2.288908194 |
| hsa-circRNA8500-43_CBC1 | 0.000348603 | 3.855580218 |
| hsa_circ_0051714_CBC1 | 0.010430488 | 2.855480295 |
| hsa-circRNA9154-65_CBC1 | 0.005154482 | 2.129058083 |
| hsa_circ_0016456_CBC1 | 0.016065069 | 6.584139278 |
| hsa_circ_0031613_CBC1 | 0.007576922 | 2.535634069 |
| hsa_circ_0046269_CBC1 | 0.039950458 | 2.281171368 |
| hsa_circ_0024952_CBC1 | 0.00614023 | 2.451148481 |
| hsa_circ_0133833_CBC1 | 0.000555481 | 4.55813275 |
| hsa_circ_0020673_CBC1 | 0.004083252 | 2.023258185 |
| hsa_circ_0026590_CBC1 | 0.007960914 | 5.529461083 |
| hsa_circ_0049204_CBC1 | 0.000192823 | 4.053452639 |
| hsa_circ_0049207_CBC1 | 0.003123894 | 3.009646411 |
| hsa-circRNA2730-4_CBC1 | 0.00552509 | 9.000647264 |
| hsa-circRNA5884-5_CBC1 | 0.000108945 | 2.638133816 |
| hsa_circ_0017494_CBC1 | 0.00178902 | 2.010953543 |
| hsa_circ_0114797_CBC1 | 0.016015506 | 2.273699727 |
| hsa_circ_0003477_CBC1 | 0.001614531 | 8.458488739 |
| hsa_circ_0051724_CBC1 | 2.20937E-05 | 3.865658765 |
| hsa_circ_0069664_CBC1 | 0.019425951 | 3.568017938 |
| hsa_circ_0003256_CBC1 | 3.27198E-06 | 8.241775503 |
| hsa_circ_0034564_CBC1 | 3.38299E-05 | 11.37133554 |
| hsa_circ_0072008_CBC1 | 0.000769908 | 2.07337678 |
| hsa_circ_0034333_CBC1 | 0.007681533 | 3.914225731 |
| hsa_circ_0036927_CBC1 | 0.000178459 | 5.487695649 |
| hsa-circRNA11493-2_CBC1 | 0.035088227 | 2.804632871 |
| hsa_circ_0028693_CBC1 | 3.19756E-05 | 7.927658416 |
| hsa_circ_0089144_CBC1 | 0.01008722 | 2.406960596 |
| hsa-circRNA2033-124_CBC1 | 0.00428454 | 2.515144608 |
| hsa_circ_0080063_CBC1 | 0.005564364 | 2.252747544 |
| hsa_circ_0019165_CBC1 | 0.000780181 | 6.966047921 |
| hsa-circRNA10221-1_CBC1 | 0.000853374 | 4.456825033 |
| hsa_circ_0059730_CBC1 | 3.10323E-06 | 6.90270407 |
| hsa-circRNA4547-13_CBC1 | 2.97002E-05 | 7.52100034 |
| hsa-circRNA7566-17_CBC1 | 0.005038102 | 2.475447163 |
| hsa_circ_0011211_CBC1 | 0.001849232 | 3.172370967 |
| hsa_circ_0021895_CBC1 | 0.04022122 | 2.1490501 |
| hsa_circ_0081554_CBC1 | 0.000255501 | 2.141144868 |
| hsa_circ_0048678_CBC1 | 0.004954733 | 3.23569025 |
| hsa-circRNA2719-38_CBC1 | 0.000305525 | 3.207820155 |
| hsa_circ_0041732_CBC1 | 0.002328755 | 5.763611402 |
| hsa_circ_0086193_CBC1 | 0.032260079 | 2.129768626 |
| hsa_circ_0010021_CBC1 | 0.029858156 | 3.30404316 |
| hsa_circ_0029186_CBC1 | 0.015383031 | 2.005805545 |
| hsa-circRNA8092-13_CBC1 | 0.003489306 | 2.227515617 |
| hsa_circ_0007985_CBC1 | 0.00011469 | 2.08818171 |
| hsa_circ_0118101_CBC1 | 0.00222747 | 2.457553967 |
| hsa_circ_0079877_CBC1 | 0.000354885 | 11.7282949 |
| hsa-circRNA13753-17_CBC1 | 0.014475114 | 2.092520956 |
| hsa-circRNA7044-13_CBC1 | 0.001207567 | 6.837932383 |
| hsa_circ_0009804_CBC1 | 0.005424386 | 2.309882857 |
| hsa_circ_0096111_CBC1 | 0.00140759 | 2.113046809 |
| hsa_circ_0025146_CBC1 | 6.18367E-05 | 4.161304505 |
| hsa_circ_0097693_CBC1 | 0.015321008 | 2.022118204 |
| hsa_circ_0071530_CBC1 | 0.042706367 | 2.282321775 |
| hsa_circ_0080638_CBC1 | 0.00917036 | 2.051997376 |
| hsa_circ_0013725_CBC1 | 0.015268174 | 2.542380757 |
| hsa_circ_0024907_CBC1 | 0.004263194 | 3.705473562 |
| hsa_circ_0090297_CBC1 | 0.028368189 | 2.210909522 |
| hsa_circ_0086747_CBC1 | 7.94326E-07 | 15.36183129 |
| hsa-circRNA8268-5_CBC1 | 0.028891572 | 2.135472918 |
| hsa-circRNA10144-6_CBC1 | 0.025885007 | 2.504886906 |
| hsa-circRNA14781-25_CBC1 | 0.009882296 | 2.756689715 |
| hsa_circ_0042570_CBC1 | 0.000785728 | 2.330674334 |
| hsa_circ_0061629_CBC1 | 0.004891234 | 2.643491939 |
| hsa-circRNA9929-27_CBC1 | 0.000124764 | 5.675365094 |
| hsa-circRNA8391-10_CBC1 | 0.000778627 | 2.226235653 |
| hsa_circ_0003517_CBC1 | 0.001493646 | 3.112020446 |
| hsa_circ_0064222_CBC1 | 0.001363101 | 4.596498108 |
| hsa_circ_0027117_CBC1 | 0.000379486 | 2.785365514 |
| hsa_circ_0023007_CBC1 | 0.0084491 | 3.175845542 |
| hsa-circRNA3507-3_CBC1 | 0.000411352 | 2.378959533 |
| hsa_circ_0025271_CBC1 | 0.007208844 | 2.695878455 |
| hsa_circ_0060362_CBC1 | 0.000891287 | 6.531471888 |
| hsa_circ_0106878_CBC1 | 0.004743041 | 2.161050329 |
| hsa_circ_0088999_CBC1 | 0.001088295 | 2.901679692 |
| hsa_circ_0024911_CBC1 | 0.000372637 | 3.379794101 |
| hsa_circ_0006143_CBC1 | 0.001650115 | 5.328020831 |
| hsa_circ_0056000_CBC1 | 0.005479368 | 4.777409606 |
| hsa_circ_0052587_CBC1 | 0.017209676 | 2.563764736 |
| hsa_circ_0030281_CBC1 | 0.008947063 | 3.109651761 |
| hsa_circ_0098808_CBC1 | 0.004179454 | 2.160196102 |
| hsa_circ_0049201_CBC1 | 0.000333998 | 3.832782968 |
| hsa_circ_0072451_CBC1 | 0.030930036 | 2.148035938 |
| hsa-circRNA5427-6_CBC1 | 0.000307143 | 5.377232993 |
| hsa_circ_0111624_CBC1 | 0.001329386 | 14.28567117 |
| hsa-circRNA14844-19_CBC1 | 0.04362718 | 2.933251506 |
| hsa_circ_0029541_CBC1 | 0.000348424 | 3.978906291 |
| hsa-circRNA11838-30_CBC1 | 0.027617764 | 2.702731254 |
| hsa_circ_0102374_CBC1 | 0.007450517 | 2.135958794 |
| hsa_circ_0039713_CBC1 | 0.027423887 | 2.205049633 |
| hsa_circ_0045018_CBC1 | 0.002661956 | 2.488747899 |
| hsa_circ_0100756_CBC1 | 0.010551506 | 3.309803162 |
| hsa-circRNA2639-3_CBC1 | 0.000693979 | 3.37806427 |
| hsa_circ_0035996_CBC1 | 0.001834714 | 3.997384726 |
| hsa_circ_0029502_CBC1 | 0.000448485 | 2.925231058 |
| hsa_circ_0067651_CBC1 | 0.00999689 | 2.813409475 |
| hsa_circ_0015772_CBC1 | 0.000434544 | 10.40960349 |
| hsa_circ_0029540_CBC1 | 0.000113878 | 4.915974674 |
| hsa_circ_0084394_CBC1 | 0.000392781 | 2.134389537 |
| hsa-circRNA14844-28_CBC1 | 0.047445141 | 2.067830744 |
| hsa_circ_0003913_CBC1 | 0.042264957 | 2.592918373 |
| hsa_circ_0032899_CBC1 | 5.41559E-05 | 2.389540528 |
| hsa_circ_0006767_CBC1 | 8.8119E-05 | 4.067799483 |
| hsa-circRNA5383-7_CBC1 | 2.87159E-05 | 12.86567856 |
| hsa-circRNA10187-35_CBC1 | 0.00305585 | 2.429942593 |
| hsa-circRNA11493-16_CBC1 | 0.003109257 | 4.802736069 |
| hsa_circ_0133042_CBC1 | 0.000971925 | 3.85242655 |
| hsa_circ_0084550_CBC1 | 0.004485162 | 2.284180664 |
| hsa-circRNA4547-11_CBC1 | 3.76012E-05 | 9.143622274 |
| hsa_circ_0043657_CBC1 | 0.005058919 | 2.013790297 |
| hsa_circ_0047932_CBC1 | 0.002475077 | 2.280392161 |
| hsa_circ_0018492_CBC1 | 0.000539633 | 3.396834157 |
| hsa_circ_0080556_CBC1 | 5.88736E-05 | 2.467027965 |
| hsa_circ_0014764_CBC1 | 0.000535873 | 5.867410156 |
| hsa_circ_0102388_CBC1 | 0.000678821 | 4.410037544 |
| hsa_circ_0084066_CBC1 | 0.000256692 | 4.84241936 |
| hsa_circ_0091717_CBC1 | 0.00305584 | 3.483514407 |
| hsa_circ_0046097_CBC1 | 0.002079432 | 2.228123048 |
| hsa_circ_0139911_CBC1 | 0.031875887 | 2.168008608 |
| hsa-circRNA1623-8_CBC1 | 0.033928327 | 2.030413093 |
| hsa-circRNA12701-34_CBC1 | 0.006792696 | 2.033201357 |
| hsa-circRNA15933-4_CBC1 | 0.000743613 | 2.047762767 |
| hsa-circRNA12057-4_CBC1 | 0.001872528 | 2.509515224 |
| hsa_circ_0039233_CBC1 | 0.003451094 | 3.827284853 |
| hsa_circ_0042120_CBC1 | 0.000240369 | 2.352541241 |
| hsa_circ_0026201_CBC1 | 0.000291682 | 4.708301397 |
| hsa_circ_0008713_CBC1 | 0.002364682 | 2.372831386 |
| hsa_circ_0063046_CBC1 | 0.00155594 | 2.690989305 |
| hsa_circ_0100701_CBC1 | 0.001529418 | 5.120071474 |
| hsa_circ_0024918_CBC1 | 0.001055576 | 3.61637141 |
| hsa-circRNA11981-30_CBC1 | 0.004382049 | 2.989472313 |
| hsa-circRNA8303-3_CBC1 | 0.032858828 | 3.037277629 |
| hsa_circ_0043163_CBC1 | 0.014008618 | 2.083795651 |
| hsa_circ_0138099_CBC1 | 0.001604204 | 2.039323464 |
| hsa_circ_0079657_CBC1 | 0.042751573 | 2.575761439 |
| hsa_circ_0102721_CBC1 | 0.001255191 | 3.839041493 |
| hsa_circ_0120429_CBC1 | 0.023396629 | 2.236004901 |
| hsa_circ_0044404_CBC1 | 0.002362595 | 2.845297676 |
| hsa_circ_0041891_CBC1 | 0.000366686 | 2.53708062 |
| hsa-circRNA8518-7_CBC1 | 0.002971928 | 2.081163293 |
| hsa_circ_0067842_CBC1 | 0.000842071 | 4.896636229 |
| hsa_circ_0063521_CBC1 | 0.000823385 | 2.034965863 |
| hsa_circ_0062590_CBC1 | 0.021237994 | 2.003413059 |
| hsa_circ_0079876_CBC1 | 0.000427574 | 6.89148124 |
| hsa_circ_0061548_CBC1 | 0.007242195 | 2.122316563 |
| hsa_circ_0052030_CBC1 | 0.024196503 | 2.59692659 |
| hsa-circRNA5535-26_CBC1 | 0.007412319 | 4.375880582 |
| hsa-circRNA7836-1_CBC1 | 0.012040798 | 2.141290799 |
| hsa_circ_0034332_CBC1 | 0.000555001 | 3.237055478 |
| hsa_circ_0109766_CBC1 | 2.41492E-05 | 4.573402918 |
| hsa-circRNA3390-4_CBC1 | 0.001230333 | 2.700634149 |
| hsa-circRNA9451-2_CBC1 | 0.005131772 | 2.296297793 |
| hsa_circ_0041882_CBC1 | 0.001070171 | 2.283079697 |
| hsa-circRNA626-10_CBC1 | 0.022652882 | 3.729324117 |
| hsa-circRNA11493-21_CBC1 | 0.033025129 | 2.335109038 |
| hsa_circ_0051817_CBC1 | 0.025336275 | 3.477707601 |
| hsa_circ_0044704_CBC1 | 0.027298255 | 2.025593461 |
| hsa_circ_0048457_CBC1 | 0.000835878 | 2.034101243 |
| hsa-circRNA2407-7_CBC1 | 0.000735547 | 3.103477062 |
| hsa_circ_0047202_CBC1 | 0.03469147 | 2.628368524 |
| hsa_circ_0012323_CBC1 | 7.04731E-05 | 13.4194719 |
| hsa-circRNA3221-2_CBC1 | 0.000266482 | 6.120329126 |
| hsa-circRNA9556_CBC1 | 0.013634169 | 2.070081832 |
| hsa_circ_0122735_CBC1 | 0.000354118 | 2.523667087 |
| hsa-circRNA4065-4_CBC1 | 7.73063E-06 | 2.128486957 |
| hsa_circ_0085462_CBC1 | 0.001845284 | 6.393588297 |
| hsa_circ_0022421_CBC1 | 0.001378143 | 3.117955421 |
| hsa_circ_0082526_CBC1 | 0.012746063 | 2.622521471 |
| hsa_circ_0084476_CBC1 | 0.01358842 | 3.058598485 |
| hsa_circ_0064239_CBC1 | 0.000118752 | 5.686500754 |
| hsa-circRNA5308-22_CBC1 | 7.69992E-05 | 4.148460726 |
| hsa-circRNA9154-53_CBC1 | 0.006714286 | 2.506810336 |
| hsa_circ_0101886_CBC1 | 0.013590835 | 3.545212682 |
| hsa_circ_0050701_CBC1 | 5.17582E-05 | 13.20645432 |
| hsa_circ_0021494_CBC1 | 0.00146434 | 2.456628806 |
| hsa-circRNA5308-31_CBC1 | 0.000353593 | 3.003012627 |
| hsa_circ_0028446_CBC1 | 0.026502423 | 2.665026213 |
| hsa_circ_0051840_CBC1 | 0.002149729 | 2.026519049 |
| hsa_circ_0105104_CBC1 | 0.000581845 | 2.394581825 |
| hsa-circRNA1649-13_CBC1 | 0.000336388 | 3.398671299 |
| hsa_circ_0045874_CBC1 | 0.010635318 | 2.056381405 |
| hsa_circ_0088665_CBC1 | 0.027543603 | 2.839938523 |
| hsa_circ_0085474_CBC1 | 0.006647436 | 3.872237416 |
| hsa_circ_0020494_CBC1 | 0.000491269 | 12.05955712 |
| hsa_circ_0108155_CBC1 | 0.006537516 | 5.051879204 |
| hsa-circRNA2033-8_CBC1 | 0.006343419 | 2.72060641 |
| hsa_circ_0066939_CBC1 | 0.002037981 | 8.639370264 |
| hsa-circRNA11493-31_CBC1 | 0.001490458 | 6.985179447 |
| hsa_circ_0081665_CBC1 | 0.000126928 | 2.080498435 |
| hsa-circRNA1448-10_CBC1 | 0.017209325 | 2.448482812 |
| hsa_circ_0022755_CBC1 | 1.97544E-05 | 6.197080494 |
| hsa-circRNA10893-25_CBC1 | 1.32412E-05 | 2.682960542 |
| hsa-circRNA1030-16_CBC1 | 0.002828242 | 5.948211753 |
| hsa_circ_0112925_CBC1 | 0.001174476 | 2.120227472 |
| hsa-circRNA10206-34_CBC1 | 0.000793939 | 4.166836179 |
| hsa-circRNA2520-30_CBC1 | 0.000242439 | 6.820694615 |
| hsa_circ_0088960_CBC1 | 0.001129553 | 2.306694278 |
| hsa_circ_0012198_CBC1 | 0.004491985 | 2.619363372 |
| hsa-circRNA8968-6_CBC1 | 0.00842135 | 2.512666335 |
| hsa_circ_0024908_CBC1 | 5.0364E-05 | 4.139519996 |
| hsa-circRNA2805-2_CBC1 | 3.09558E-05 | 6.723375106 |
| hsa_circ_0121373_CBC1 | 0.021681002 | 2.220598643 |
| hsa_circ_0117622_CBC1 | 0.03237985 | 2.272707355 |
| hsa_circ_0041043_CBC1 | 0.015646486 | 3.357888642 |
| hsa_circ_0034643_CBC1 | 0.001411477 | 2.191350095 |
| hsa_circ_0090142_CBC1 | 0.025140925 | 3.244657934 |
| hsa-circRNA15520-4_CBC1 | 0.002575116 | 3.200439363 |
| hsa-circRNA13043-21_CBC1 | 0.02547438 | 2.273077638 |
| hsa_circ_0127832_CBC1 | 0.004569293 | 2.642688294 |
| hsa_circ_0088980_CBC1 | 3.58427E-05 | 2.091732203 |
| hsa_circ_0064254_CBC1 | 0.000136161 | 6.939845014 |
| hsa-circRNA626-12_CBC1 | 0.000856256 | 2.25800529 |
| hsa_circ_0029929_CBC1 | 0.000528235 | 4.154113436 |
| hsa-circRNA6939-4_CBC1 | 0.006170091 | 2.822059143 |
| hsa-circRNA2520-5_CBC1 | 0.00014108 | 6.868919254 |
| hsa_circ_0059723_CBC1 | 0.000129289 | 6.811920783 |
| hsa_circ_0122734_CBC1 | 0.016483162 | 2.577377428 |
| hsa_circ_0109291_CBC1 | 0.014070397 | 2.897861066 |
| hsa_circ_0072762_CBC1 | 0.000456639 | 6.263791841 |
| hsa_circ_0131324_CBC1 | 0.030291734 | 2.108843787 |
| hsa-circRNA3458-11_CBC1 | 0.000225758 | 2.700710666 |
| hsa_circ_0099005_CBC1 | 0.000278827 | 3.836177522 |
| hsa_circ_0051726_CBC1 | 4.40101E-05 | 3.716773401 |
| hsa-circRNA11458-8_CBC1 | 0.000394992 | 4.898054269 |
| hsa_circ_0036869_CBC1 | 0.009938031 | 4.591401755 |
| hsa_circ_0135150_CBC1 | 0.013507795 | 2.807778076 |
| hsa-circRNA946-1_CBC1 | 0.001997693 | 4.153694263 |
| hsa_circ_0137799_CBC1 | 0.001283371 | 2.028631492 |
| hsa_circ_0108154_CBC1 | 0.004131392 | 7.14597416 |
| hsa-circRNA11595-22_CBC1 | 0.004591444 | 3.12504665 |
| hsa_circ_0043537_CBC1 | 0.000226187 | 8.805122434 |
| hsa_circ_0028688_CBC1 | 2.90579E-05 | 6.674920011 |
| hsa_circ_0014754_CBC1 | 0.000526831 | 7.653986463 |
| hsa-circRNA12453-8_CBC1 | 0.000672905 | 4.419023972 |
| hsa_circ_0045421_CBC1 | 0.024130601 | 2.080174564 |
| hsa-circRNA11493-50_CBC1 | 0.000372101 | 4.418916027 |
| hsa_circ_0082536_CBC1 | 0.011918031 | 2.144670932 |
| hsa_circ_0109000_CBC1 | 0.003551641 | 2.652138487 |
| hsa-circRNA1008-15_CBC1 | 0.000300479 | 3.324116594 |
| hsa_circ_0061305_CBC1 | 0.0135301 | 3.318243577 |
| hsa_circ_0088931_CBC1 | 4.82787E-05 | 2.526480861 |
| hsa_circ_0008324_CBC1 | 0.007630905 | 2.560097933 |
| hsa_circ_0112245_CBC1 | 0.0029653 | 2.029636309 |
| hsa_circ_0043948_CBC1 | 0.000696452 | 8.771299574 |
| hsa_circ_0019074_CBC1 | 0.004569613 | 4.631181468 |
| hsa_circ_0133657_CBC1 | 0.012884273 | 2.28651858 |
| hsa_circ_0024909_CBC1 | 0.000162304 | 6.814279918 |
| hsa_circ_0076203_CBC1 | 0.004136327 | 2.14139228 |
| hsa_circ_0072000_CBC1 | 0.009320668 | 2.420090318 |
| hsa_circ_0037329_CBC1 | 0.008656474 | 2.177690921 |
| hsa-circRNA118-3_CBC1 | 0.001218513 | 2.06887326 |
| hsa_circ_0007944_CBC1 | 2.24995E-05 | 10.40401482 |
| hsa_circ_0128650_CBC1 | 8.48289E-05 | 5.609140484 |
| hsa_circ_0102393_CBC1 | 1.33434E-05 | 3.879226512 |
| hsa_circ_0014756_CBC1 | 0.000178702 | 9.219092263 |
| hsa_circ_0050017_CBC1 | 0.016665269 | 2.234245814 |
| hsa_circ_0008896_CBC1 | 0.002005851 | 2.390851882 |
| hsa-circRNA851-49_CBC1 | 0.003568812 | 2.16512247 |
| hsa_circ_0113639_CBC1 | 0.006675605 | 2.21497209 |
| hsa-circRNA2719-17_CBC1 | 0.002784336 | 3.763206286 |
| hsa_circ_0074321_CBC1 | 0.00056873 | 2.118034072 |
| hsa_circ_0012523_CBC1 | 0.005002906 | 5.446488263 |
| hsa-circRNA626-6_CBC1 | 0.001857171 | 2.49894474 |
| hsa-circRNA2033-87_CBC1 | 0.004292516 | 3.659034505 |
| hsa_circ_0056373_CBC1 | 0.004150187 | 2.437206206 |
| hsa_circ_0043536_CBC1 | 0.004731788 | 7.387633687 |
| hsa_circ_0105639_CBC1 | 0.001890628 | 2.417441091 |
| hsa-circRNA5886-26_CBC1 | 0.00592896 | 2.007285109 |
| hsa_circ_0098318_CBC1 | 1.16277E-05 | 3.891792543 |
| hsa_circ_0038733_CBC1 | 0.000942461 | 2.404828002 |
| hsa_circ_0026188_CBC1 | 0.002122746 | 2.743932172 |
| hsa_circ_0053160_CBC1 | 0.004509539 | 2.19614909 |
| hsa_circ_0015565_CBC1 | 0.030875356 | 2.770218424 |
| hsa_circ_0010014_CBC1 | 0.023715093 | 2.809472278 |
| hsa_circ_0034567_CBC1 | 0.000943448 | 6.477075285 |
| hsa_circ_0061844_CBC1 | 0.034090287 | 2.058042337 |
| hsa-circRNA12871-14_CBC1 | 0.047551316 | 2.289255658 |
| hsa_circ_0128159_CBC1 | 0.001280753 | 2.398439424 |
| hsa_circ_0059719_CBC1 | 0.002440202 | 7.981036738 |
| hsa_circ_0025038_CBC1 | 3.03511E-07 | 3.454709877 |
| hsa-circRNA11372-9_CBC1 | 0.022571608 | 5.161033072 |
| hsa_circ_0026743_CBC1 | 0.006255626 | 2.029794677 |
| hsa-circRNA11180-15_CBC1 | 2.14184E-05 | 2.881004246 |
| hsa_circ_0015910_CBC1 | 0.004370017 | 2.039011947 |
| hsa_circ_0034691_CBC1 | 2.80053E-05 | 6.138565801 |
| hsa_circ_0046974_CBC1 | 0.017927175 | 2.134463747 |
| hsa_circ_0064249_CBC1 | 0.001190662 | 3.967386557 |
| hsa_circ_0016362_CBC1 | 0.000207247 | 9.722023226 |
| hsa-circRNA2866-153_CBC1 | 0.00370395 | 2.003969821 |
| hsa_circ_0016388_CBC1 | 0.003399783 | 5.475695752 |
| hsa-circRNA5535-6_CBC1 | 0.000853852 | 3.582637946 |
| hsa_circ_0018971_CBC1 | 0.001275597 | 2.044188912 |
| hsa_circ_0024726_CBC1 | 0.029621028 | 2.628236636 |
| hsa-circRNA14363-14_CBC1 | 0.001435228 | 2.003931292 |
| hsa_circ_0097685_CBC1 | 0.0001323 | 2.403637719 |
| hsa_circ_0070121_CBC1 | 0.017544759 | 2.12707608 |
| hsa-circRNA15598-13_CBC1 | 0.006444858 | 3.994727809 |
| hsa_circ_0012162_CBC1 | 1.88996E-05 | 3.328195226 |
| hsa_circ_0024104_CBC1 | 1.12109E-06 | 2.635319278 |
| hsa_circ_0043957_CBC1 | 0.025934988 | 2.28095867 |
| hsa-circRNA2528-3_CBC1 | 0.001061156 | 4.381562648 |
| hsa-circRNA14089-4_CBC1 | 0.002237113 | 3.320105627 |
| hsa_circ_0049211_CBC1 | 0.001990024 | 2.516502558 |
| hsa_circ_0086484_CBC1 | 0.008727951 | 2.697301717 |
| hsa_circ_0085470_CBC1 | 0.002602987 | 4.509513381 |
| hsa_circ_0040497_CBC1 | 0.000435839 | 2.542974253 |
| hsa_circ_0095568_CBC1 | 0.000584287 | 2.438651314 |
| hsa_circ_0002296_CBC1 | 0.003708516 | 2.289133087 |
| hsa_circ_0034571_CBC1 | 1.43456E-05 | 13.75612174 |
| hsa_circ_0002645_CBC1 | 6.86676E-05 | 11.22730109 |
| hsa-circRNA1772-4_CBC1 | 0.002190402 | 3.400090862 |
| hsa_circ_0043978_CBC1 | 0.006984185 | 2.173100324 |
| hsa_circ_0128250_CBC1 | 0.012339921 | 2.223886051 |
| hsa_circ_0098773_CBC1 | 1.7037E-05 | 3.112156314 |
| hsa-circRNA2329-27_CBC1 | 0.040006387 | 2.328058269 |
| hsa_circ_0012157_CBC1 | 0.000652208 | 5.089564834 |
| hsa_circ_0107330_CBC1 | 0.009499778 | 3.099241657 |
| hsa-circRNA2313-4_CBC1 | 0.001497424 | 2.225299783 |
| hsa_circ_0064221_CBC1 | 0.004609991 | 2.607264093 |
| hsa_circ_0016384_CBC1 | 0.006584435 | 5.153921858 |
| hsa-circRNA5535-24_CBC1 | 0.004752566 | 3.573550031 |
| hsa-circRNA427-19_CBC1 | 0.002958956 | 2.700549126 |
| hsa-circRNA7836-47_CBC1 | 0.001311625 | 2.03424621 |
| hsa_circ_0040579_CBC1 | 9.7714E-05 | 3.42641036 |
| hsa_circ_0095265_CBC1 | 0.000107458 | 2.275123724 |
| hsa_circ_0082527_CBC1 | 0.001647102 | 2.131193725 |
| hsa-circRNA2424-25_CBC1 | 0.004100129 | 2.202951692 |
| hsa_circ_0046705_CBC1 | 0.000914768 | 4.604262946 |
| hsa-circRNA14248-32_CBC1 | 0.003302707 | 2.221944488 |
| hsa_circ_0019075_CBC1 | 0.015210916 | 3.403222431 |
| hsa-circRNA946-12_CBC1 | 0.008845028 | 2.62383197 |
| hsa-circRNA4547-19_CBC1 | 0.00241164 | 2.945546348 |
| hsa_circ_0088974_CBC1 | 0.000259554 | 2.351893993 |
| hsa_circ_0074314_CBC1 | 0.002418486 | 2.09095266 |
| hsa_circ_0115146_CBC1 | 0.026488435 | 2.732832929 |
| hsa_circ_0122581_CBC1 | 0.000488304 | 4.921094656 |
| hsa_circ_0006217_CBC1 | 0.00632148 | 5.598332147 |
| hsa_circ_0097716_CBC1 | 0.013669216 | 2.235122051 |
| hsa-circRNA16136-9_CBC1 | 0.016842383 | 2.006428937 |
| hsa-circRNA5149-12_CBC1 | 0.009381518 | 4.157957822 |
| hsa-circRNA5213-5_CBC1 | 0.002266389 | 6.183891978 |
| hsa_circ_0079864_CBC1 | 5.2702E-05 | 7.128974485 |
| hsa_circ_0094481_CBC1 | 0.001416062 | 7.888553059 |
| hsa-circRNA8885-13_CBC1 | 0.004146566 | 5.38997448 |
| hsa_circ_0026145_CBC1 | 0.000160421 | 8.090476653 |
| hsa_circ_0020742_CBC1 | 0.008748785 | 2.017172528 |
| hsa_circ_0014749_CBC1 | 0.00521213 | 7.650320495 |
| hsa_circ_0086750_CBC1 | 0.000307729 | 6.642283343 |
| hsa_circ_0002838_CBC1 | 0.001451148 | 4.924928275 |
| hsa-circRNA10893-4_CBC1 | 0.000258772 | 3.995479737 |
| hsa_circ_0111695_CBC1 | 0.001682898 | 2.113211418 |
| hsa_circ_0004333_CBC1 | 0.004813512 | 2.44276073 |
| hsa-circRNA7836-79_CBC1 | 0.002350362 | 2.224009805 |
| hsa_circ_0018508_CBC1 | 0.021447751 | 2.576317357 |
| hsa_circ_0063164_CBC1 | 0.000635147 | 2.608099101 |
| hsa_circ_0026956_CBC1 | 0.00116588 | 2.008692873 |
| hsa_circ_0038061_CBC1 | 0.008671945 | 2.770461149 |
| hsa-circRNA13698-3_CBC1 | 0.004252134 | 5.490651673 |
| hsa_circ_0063037_CBC1 | 0.000655321 | 2.902657136 |
| hsa_circ_0059873_CBC1 | 0.001540885 | 2.952485658 |
| hsa_circ_0083897_CBC1 | 0.010069391 | 2.508554536 |
| hsa-circRNA10887-9_CBC1 | 0.000608051 | 2.495209212 |
| hsa_circ_0012362_CBC1 | 0.002667407 | 4.967492736 |
| hsa_circ_0064231_CBC1 | 0.006723477 | 2.797685363 |
| hsa_circ_0047947_CBC1 | 0.0001061 | 3.969152446 |
| hsa_circ_0138113_CBC1 | 0.01893674 | 2.772907175 |
| hsa_circ_0041868_CBC1 | 0.000365974 | 2.066187613 |
| hsa_circ_0060216_CBC1 | 0.000551008 | 2.418202125 |
| hsa_circ_0005467_CBC1 | 0.000399703 | 3.065366874 |
| hsa_circ_0064204_CBC1 | 0.000182609 | 3.968193313 |
| hsa_circ_0076029_CBC1 | 0.002627758 | 2.125704661 |
| hsa_circ_0048606_CBC1 | 0.005038495 | 2.149097444 |
| hsa_circ_0010019_CBC1 | 0.025418054 | 2.932208846 |
| hsa_circ_0064226_CBC1 | 0.000697781 | 6.56854146 |
| hsa-circRNA2298-2_CBC1 | 0.000187247 | 6.179543559 |
| hsa-circRNA9222-2_CBC1 | 0.018866258 | 3.180933352 |
| hsa_circ_0082296_CBC1 | 0.018549467 | 2.270077894 |
| hsa-circRNA11981-65_CBC1 | 0.000441509 | 2.428819426 |
| hsa_circ_0088972_CBC1 | 0.001346775 | 2.585112322 |
| hsa_circ_0101919_CBC1 | 0.005690264 | 6.239534106 |
| hsa_circ_0034694_CBC1 | 2.656E-06 | 5.133720099 |
| hsa_circ_0044395_CBC1 | 0.009832911 | 2.478685141 |
| hsa_circ_0059721_CBC1 | 5.8368E-06 | 6.342276392 |
| hsa_circ_0010024_CBC1 | 0.039889561 | 2.972586767 |
| hsa-circRNA10312-26_CBC1 | 0.000133452 | 3.607748542 |
| hsa_circ_0032164_CBC1 | 0.003351199 | 3.712971836 |
| hsa_circ_0062263_CBC1 | 0.006166556 | 3.700925919 |
| hsa-circRNA1649-38_CBC1 | 4.21736E-05 | 2.549792262 |
| hsa_circ_0093453_CBC1 | 0.003525217 | 2.670429564 |
| hsa_circ_0094370_CBC1 | 0.004422909 | 4.76649001 |
| hsa-circRNA8791-2_CBC1 | 0.000175681 | 7.23453618 |
| hsa_circ_0088959_CBC1 | 0.001044446 | 2.691595393 |
| hsa_circ_0127798_CBC1 | 0.000773921 | 6.792765349 |
| hsa-circRNA9622-8_CBC1 | 0.010544671 | 2.132687448 |
| hsa_circ_0029537_CBC1 | 0.000834561 | 4.673314327 |
| hsa-circRNA8500-5_CBC1 | 0.001695235 | 3.340982878 |
| hsa_circ_0056155_CBC1 | 0.049960135 | 2.555084194 |
| hsa_circ_0059068_CBC1 | 0.042201152 | 2.595283656 |
| hsa_circ_0076247_CBC1 | 2.2764E-05 | 3.850637254 |
| hsa_circ_0063999_CBC1 | 0.010571283 | 2.064586806 |
| hsa-circRNA7831-9_CBC1 | 0.004434621 | 2.149232951 |
| hsa_circ_0026814_CBC1 | 0.004519726 | 2.061798196 |
| hsa_circ_0096291_CBC1 | 0.003812452 | 2.042907789 |
| hsa_circ_0053001_CBC1 | 0.001166367 | 3.620457806 |
| hsa-circRNA15355-20_CBC1 | 0.001107102 | 2.378773042 |
| hsa_circ_0017976_CBC1 | 0.000694274 | 2.406270074 |
| hsa_circ_0032172_CBC1 | 0.000145894 | 4.291251913 |
| hsa_circ_0128078_CBC1 | 3.8146E-05 | 10.41104803 |
| hsa_circ_0097849_CBC1 | 0.020854027 | 2.194009238 |
| hsa_circ_0043541_CBC1 | 0.00214032 | 3.929296483 |
| hsa_circ_0063793_CBC1 | 0.000147383 | 5.030013651 |
| hsa_circ_0110333_CBC1 | 0.005717266 | 3.852460112 |
| hsa_circ_0049224_CBC1 | 0.000761604 | 2.504555933 |
| hsa_circ_0005930_CBC1 | 0.002018099 | 3.355121535 |
| hsa_circ_0049103_CBC1 | 0.017344615 | 3.12358194 |
| hsa-circRNA2313-18_CBC1 | 0.003112306 | 2.279471452 |
| hsa_circ_0037519_CBC1 | 0.000133727 | 8.143679978 |
| hsa_circ_0134619_CBC1 | 0.000997913 | 2.282673596 |
| hsa-circRNA470-25_CBC1 | 0.005829917 | 3.170474497 |
| hsa_circ_0023588_CBC1 | 0.020235215 | 2.091897822 |
| hsa_circ_0095558_CBC1 | 0.007212987 | 2.282359754 |
| hsa_circ_0074945_CBC1 | 0.047552373 | 2.324185278 |
| hsa_circ_0021731_CBC1 | 0.002370143 | 3.538618785 |
| hsa-circRNA13986-47_CBC1 | 0.046499432 | 2.064671257 |
| hsa_circ_0088421_CBC1 | 0.026097157 | 2.657375603 |
| hsa-circRNA5426-2_CBC1 | 0.01618654 | 3.270007833 |
| hsa_circ_0097054_CBC1 | 0.000158152 | 4.455793297 |
| hsa_circ_0125980_CBC1 | 0.000348012 | 3.38914031 |
| hsa-circRNA264-16_CBC1 | 0.009883095 | 5.113233061 |
| hsa-circRNA8500-26_CBC1 | 0.00376948 | 6.483599364 |
| hsa_circ_0068872_CBC1 | 0.038702025 | 2.688667806 |
| hsa-circRNA3458-6_CBC1 | 0.000131991 | 3.76787751 |
| hsa_circ_0029087_CBC1 | 0.001267218 | 2.81298732 |
| hsa-circRNA11966-60_CBC1 | 0.006087051 | 3.296845914 |
| hsa_circ_0007051_CBC1 | 2.68621E-06 | 9.847504613 |
| hsa_circ_0093979_CBC1 | 0.034480625 | 2.245693009 |
| hsa_circ_0048674_CBC1 | 0.005854495 | 3.517383554 |
| hsa_circ_0036739_CBC1 | 0.000404678 | 2.084375185 |
| hsa_circ_0076729_CBC1 | 0.047528131 | 3.621549711 |
| hsa_circ_0052013_CBC1 | 0.000679657 | 2.467047306 |
| hsa-circRNA11838-12_CBC1 | 0.023485068 | 2.144800936 |
| hsa_circ_0025159_CBC1 | 0.005969616 | 3.220426348 |
| hsa_circ_0123865_CBC1 | 0.002447301 | 2.54285009 |
| hsa-circRNA3767-6_CBC1 | 0.001091371 | 2.363228647 |
| hsa_circ_0067839_CBC1 | 0.00139807 | 4.234035157 |
| hsa_circ_0071685_CBC1 | 3.98732E-05 | 7.286212118 |
| hsa_circ_0049206_CBC1 | 0.000578108 | 2.780860143 |
| hsa_circ_0049212_CBC1 | 0.000524337 | 5.607590079 |
| hsa_circ_0012382_CBC1 | 0.007602124 | 4.612047731 |
| hsa_circ_0087796_CBC1 | 0.019435077 | 2.083652169 |
| hsa-circRNA3556-12_CBC1 | 0.044106922 | 2.306999851 |
| hsa_circ_0022810_CBC1 | 0.005810901 | 2.55666268 |
| hsa_circ_0060212_CBC1 | 0.000173551 | 2.462232979 |
| hsa-circRNA10312-11_CBC1 | 0.015111738 | 2.216231003 |
| hsa-circRNA14844-1_CBC1 | 0.04885563 | 2.006985877 |
| hsa_circ_0102873_CBC1 | 0.037113075 | 2.558845153 |
| hsa_circ_0051728_CBC1 | 9.46328E-06 | 6.02082635 |
| hsa_circ_0040496_CBC1 | 0.001656801 | 2.897006343 |
| hsa-circRNA1257-15_CBC1 | 0.049904904 | 2.154524049 |
| hsa_circ_0024954_CBC1 | 0.027315744 | 2.538035898 |
| hsa-circRNA13043-34_CBC1 | 0.022000209 | 3.473534114 |
| hsa-circRNA11493-14_CBC1 | 0.001644515 | 4.856443812 |
| hsa_circ_0084231_CBC1 | 0.006563926 | 3.102873883 |
| hsa_circ_0018327_CBC1 | 0.012641485 | 2.283928309 |
| hsa_circ_0061304_CBC1 | 0.047019484 | 2.856028628 |
| hsa-circRNA10541-7_CBC1 | 0.024967823 | 2.706259277 |
| hsa_circ_0002499_CBC1 | 0.004504677 | 4.278668014 |
| hsa_circ_0040583_CBC1 | 0.002031186 | 3.318635421 |
| hsa-circRNA11084-1_CBC1 | 0.007689637 | 8.270126339 |
| hsa-circRNA4547-6_CBC1 | 3.28525E-05 | 7.912949317 |
| hsa_circ_0036726_CBC1 | 0.002092302 | 4.30698055 |
| hsa_circ_0106817_CBC1 | 0.000233909 | 5.124563101 |
| hsa-circRNA9333-12_CBC1 | 0.00020442 | 6.783483399 |
| hsa_circ_0000101_CBC1 | 0.000975426 | 2.247940604 |
| hsa_circ_0005879_CBC1 | 0.003271886 | 2.72037798 |
| hsa_circ_0012163_CBC1 | 0.000290062 | 4.850339702 |
| hsa-circRNA1391-16_CBC1 | 0.044543958 | 2.037422048 |
| hsa-circRNA391-16_CBC1 | 0.022212782 | 3.431189836 |
| hsa-circRNA8276-8_CBC1 | 0.007346226 | 2.87703435 |
| hsa_circ_0047943_CBC1 | 0.000360832 | 4.412363319 |
| hsa-circRNA10441-31_CBC1 | 0.007446012 | 2.606022864 |
| hsa_circ_0105166_CBC1 | 0.019660218 | 2.15252936 |
| hsa_circ_0085476_CBC1 | 0.004703434 | 4.607930515 |
| hsa-circRNA13698-7_CBC1 | 0.001261905 | 9.716755237 |
| hsa_circ_0138106_CBC1 | 0.000117057 | 2.053922885 |
| hsa_circ_0131599_CBC1 | 0.006870544 | 2.282956959 |
| hsa-circRNA2599-11_CBC1 | 4.93725E-05 | 4.812274617 |
| hsa_circ_0038920_CBC1 | 0.007125085 | 2.082306335 |
| hsa_circ_0065079_CBC1 | 0.007091653 | 2.353340847 |
| hsa_circ_0031789_CBC1 | 0.025774409 | 2.347489011 |
| hsa_circ_0088422_CBC1 | 0.040100825 | 2.653476469 |
| hsa_circ_0060949_CBC1 | 0.000717876 | 3.959816028 |
| hsa-circRNA270-2_CBC1 | 0.009465737 | 2.062023662 |
| hsa-circRNA15162-30_CBC1 | 0.005474122 | 2.600889601 |
| hsa_circ_0130713_CBC1 | 0.02470172 | 2.206809827 |
| hsa_circ_0026194_CBC1 | 0.000247655 | 4.162365733 |
| hsa_circ_0023524_CBC1 | 0.03364617 | 2.195490753 |
| hsa_circ_0126893_CBC1 | 0.030592117 | 2.477254956 |
| hsa-circRNA11242-73_CBC1 | 0.019825758 | 3.06337191 |
| hsa_circ_0026369_CBC1 | 0.004737295 | 2.319030915 |
| hsa_circ_0102233_CBC1 | 0.001934422 | 2.409752518 |
| hsa_circ_0028691_CBC1 | 0.000274492 | 6.907250409 |
| hsa-circRNA11838-21_CBC1 | 0.010140753 | 2.261190761 |
| hsa-circRNA9158-3_CBC1 | 0.041025624 | 2.508244571 |
| hsa-circRNA2502-14_CBC1 | 0.0004107 | 4.356656737 |
| hsa-circRNA3390-10_CBC1 | 1.51128E-05 | 3.761285278 |
| hsa-circRNA11588-17_CBC1 | 0.020589755 | 2.048079015 |
| hsa-circRNA10466-10_CBC1 | 0.033610656 | 2.32440196 |
| hsa_circ_0136684_CBC1 | 0.002115925 | 2.236928419 |
| hsa-circRNA9240-9_CBC1 | 0.003797087 | 2.307230338 |
| hsa_circ_0060465_CBC1 | 0.000387841 | 3.320705063 |
| hsa_circ_0114859_CBC1 | 0.0172471 | 3.150232828 |
| hsa-circRNA13111-4_CBC1 | 0.000707362 | 3.245400852 |
| hsa_circ_0040489_CBC1 | 0.005766882 | 2.257973511 |
| hsa_circ_0040105_CBC1 | 0.018027273 | 2.018883496 |
| hsa_circ_0063179_CBC1 | 0.005913188 | 2.024096469 |
| hsa_circ_0001594_CBC1 | 0.001397875 | 3.338869279 |
| hsa_circ_0028505_CBC1 | 0.007093318 | 2.070204011 |
| hsa_circ_0086371_CBC1 | 0.001829747 | 2.139442497 |
| hsa_circ_0004252_CBC1 | 0.000607912 | 5.599472959 |
| hsa-circRNA2910-4_CBC1 | 3.65525E-06 | 9.105416122 |
| hsa_circ_0121832_CBC1 | 0.012920105 | 3.006131405 |
| hsa_circ_0045013_CBC1 | 0.014130922 | 3.210897773 |
| hsa_circ_0034693_CBC1 | 0.000142185 | 4.214353525 |
| hsa_circ_0073949_CBC1 | 0.004226272 | 3.046380012 |
| hsa-circRNA617-17_CBC1 | 0.013467301 | 2.096472829 |
| hsa_circ_0024568_CBC1 | 0.001028129 | 2.298146087 |
| hsa_circ_0137803_CBC1 | 0.000463288 | 2.063441765 |
| hsa_circ_0101890_CBC1 | 0.003666443 | 2.362189781 |
| hsa_circ_0074842_CBC1 | 0.000166415 | 6.773939124 |
| hsa_circ_0041054_CBC1 | 0.001626472 | 5.653875257 |
| hsa_circ_0104385_CBC1 | 0.020296388 | 2.04992006 |
| hsa_circ_0028598_CBC1 | 0.000140438 | 4.219889844 |
| hsa_circ_0115534_CBC1 | 0.011763136 | 3.510195326 |
| hsa-circRNA2033-103_CBC1 | 0.002571711 | 2.65811221 |
| hsa_circ_0026925_CBC1 | 0.007414882 | 2.115469353 |
| hsa_circ_0080980_CBC1 | 0.005945557 | 2.592896471 |
| hsa_circ_0038747_CBC1 | 0.015888073 | 2.009868472 |
| hsa_circ_0022420_CBC1 | 0.000188007 | 4.751377223 |
| hsa_circ_0111662_CBC1 | 0.017295447 | 2.328177152 |
| hsa_circ_0121606_CBC1 | 0.003028109 | 9.681227467 |
| hsa-circRNA3568-3_CBC1 | 0.000871936 | 3.662746977 |
| hsa_circ_0026141_CBC1 | 6.68638E-05 | 20.18969727 |
| hsa_circ_0087033_CBC1 | 0.006541048 | 2.719313523 |
| hsa_circ_0026616_CBC1 | 0.000498103 | 2.418903213 |
| hsa_circ_0066513_CBC1 | 0.040209974 | 2.000102637 |
| hsa-circRNA3066-3_CBC1 | 0.000223943 | 2.099827673 |
| hsa_circ_0013384_CBC1 | 0.000266491 | 2.74841174 |
| hsa-circRNA13048-4_CBC1 | 0.042679088 | 2.337725657 |
| hsa_circ_0032902_CBC1 | 0.001208197 | 2.199833581 |
| hsa-circRNA8518-38_CBC1 | 0.003139005 | 2.058109742 |
| hsa-circRNA5287-2_CBC1 | 0.004637855 | 2.236176029 |
| hsa-circRNA2719-31_CBC1 | 0.005175085 | 6.739868087 |
| hsa_circ_0076629_CBC1 | 0.012463701 | 2.18513063 |
| hsa_circ_0003139_CBC1 | 0.000746184 | 3.730714665 |
| hsa_circ_0099416_CBC1 | 0.048933692 | 2.055485602 |
| hsa_circ_0110377_CBC1 | 3.78007E-06 | 5.67759701 |
| hsa_circ_0039237_CBC1 | 0.010901846 | 2.974975046 |
| hsa_circ_0102829_CBC1 | 0.024130197 | 2.094471752 |
| hsa_circ_0027113_CBC1 | 0.018529614 | 2.699636672 |
| hsa_circ_0081414_CBC1 | 2.97618E-05 | 3.807653869 |
| hsa_circ_0046535_CBC1 | 0.00619781 | 2.011785992 |
| hsa-circRNA7275-77_CBC1 | 0.002218606 | 2.6503865 |
| hsa_circ_0032011_CBC1 | 0.025667484 | 2.139620056 |
| hsa_circ_0005877_CBC1 | 0.000351168 | 3.155805032 |
| hsa_circ_0102761_CBC1 | 0.00780665 | 3.979715975 |
| hsa_circ_0135663_CBC1 | 0.001922192 | 3.42973026 |
| hsa_circ_0000402_CBC1 | 0.011780616 | 5.66812055 |
| hsa_circ_0083188_CBC1 | 0.006314061 | 3.574396397 |
| hsa_circ_0135937_CBC1 | 0.047945239 | 2.225828979 |
| hsa_circ_0004884_CBC1 | 0.003164879 | 2.230687506 |
| hsa_circ_0058982_CBC1 | 0.010828053 | 2.194956034 |
| hsa_circ_0111886_CBC1 | 0.00535729 | 4.411256044 |
| hsa_circ_0068268_CBC1 | 0.02190328 | 2.141492554 |
| hsa_circ_0049242_CBC1 | 0.000230226 | 2.401465471 |
| hsa_circ_0034597_CBC1 | 0.003879721 | 12.14885194 |
| hsa-circRNA13698-14_CBC1 | 0.001526093 | 6.070526675 |
| hsa-circRNA13465-3_CBC1 | 0.002850402 | 6.941989373 |
| hsa_circ_0059329_CBC1 | 0.000186004 | 3.167055591 |
| hsa_circ_0088983_CBC1 | 0.003974553 | 2.048773423 |
| hsa_circ_0079867_CBC1 | 0.000364133 | 6.257269125 |
| hsa-circRNA4547-3_CBC1 | 0.000619753 | 7.606556486 |
| hsa-circRNA1594-2_CBC1 | 0.037721959 | 2.197188594 |
| hsa_circ_0000661_CBC1 | 0.000570099 | 3.753763619 |
| hsa_circ_0021485_CBC1 | 0.00080946 | 2.36514944 |
| hsa-circRNA11084-3_CBC1 | 0.000139165 | 11.31842732 |
| hsa_circ_0091239_CBC1 | 0.001608035 | 5.198065752 |
| hsa_circ_0001356_CBC1 | 0.001104411 | 4.604537359 |
| hsa_circ_0088310_CBC1 | 0.001208201 | 2.432143545 |
| hsa_circ_0043156_CBC1 | 0.002001517 | 2.200303669 |
| hsa_circ_0063043_CBC1 | 4.63744E-05 | 4.836655996 |
| hsa_circ_0034579_CBC1 | 0.000313768 | 2.086969855 |
| hsa_circ_0081258_CBC1 | 0.008529551 | 2.036357797 |
| hsa-circRNA10206-35_CBC1 | 0.023873238 | 2.002346669 |
| hsa_circ_0106532_CBC1 | 0.009367989 | 4.371266906 |
| hsa_circ_0117992_CBC1 | 0.026590595 | 2.061004136 |
| hsa-circRNA11393-14_CBC1 | 0.000107019 | 9.987598043 |
| hsa_circ_0043869_CBC1 | 0.003439777 | 2.025790925 |
| hsa_circ_0043170_CBC1 | 0.004035779 | 2.277840299 |
| hsa_circ_0131596_CBC1 | 0.002510712 | 2.717223858 |
| hsa_circ_0060452_CBC1 | 0.001891529 | 2.267797661 |
| hsa_circ_0034697_CBC1 | 0.002019987 | 7.351914679 |
| hsa_circ_0065404_CBC1 | 0.000105567 | 2.608186616 |
| hsa_circ_0035180_CBC1 | 0.000832336 | 3.110601607 |
| hsa_circ_0044133_CBC1 | 0.000468383 | 3.188732752 |
| hsa_circ_0027112_CBC1 | 0.044708161 | 3.110038164 |
| hsa_circ_0040993_CBC1 | 6.4591E-05 | 3.844240678 |
| hsa-circRNA5028-9_CBC1 | 0.000188367 | 7.73599088 |
| hsa_circ_0004278_CBC1 | 0.000662852 | 6.517895317 |
| hsa_circ_0088936_CBC1 | 0.004835003 | 2.107035189 |
| hsa_circ_0016389_CBC1 | 0.004923871 | 4.945748441 |
| hsa_circ_0005301_CBC1 | 0.001417403 | 2.971923623 |
| hsa_circ_0021487_CBC1 | 0.001497651 | 2.259358101 |
| hsa_circ_0018517_CBC1 | 0.007178325 | 4.249220078 |
| hsa-circRNA7885-6_CBC1 | 0.010336618 | 3.138687188 |
| hsa_circ_0033591_CBC1 | 0.036174671 | 2.581166595 |
| hsa_circ_0040495_CBC1 | 0.000347464 | 2.757109532 |
| hsa_circ_0090149_CBC1 | 0.019619519 | 2.822240142 |
| hsa-circRNA9146-3_CBC1 | 0.01021046 | 2.12665086 |
| hsa_circ_0122848_CBC1 | 0.046925409 | 2.007353229 |
| hsa_circ_0004112_CBC1 | 0.000870139 | 2.421441504 |
| hsa-circRNA7413-7_CBC1 | 0.006571288 | 2.546467613 |
| hsa-circRNA11180-23_CBC1 | 3.40239E-05 | 2.849893876 |
| hsa_circ_0041889_CBC1 | 0.001537672 | 2.126493087 |
| hsa_circ_0089841_CBC1 | 0.006276667 | 2.743713067 |
| hsa_circ_0043950_CBC1 | 0.006404687 | 5.607253844 |
| hsa_circ_0077412_CBC1 | 0.009331437 | 3.729690635 |
| hsa_circ_0025034_CBC1 | 1.92219E-05 | 3.219008093 |
| hsa-circRNA10166-16_CBC1 | 0.036109256 | 2.303659363 |
| hsa_circ_0075190_CBC1 | 0.009950894 | 2.025895343 |
| hsa-circRNA6436-17_CBC1 | 0.000969779 | 2.338220154 |
| hsa-circRNA11458-3_CBC1 | 0.001287824 | 3.065635691 |
| hsa-circRNA9929-15_CBC1 | 0.000408739 | 4.456422421 |
| hsa-circRNA8054-17_CBC1 | 0.000245403 | 6.274513939 |
| hsa_circ_0125030_CBC1 | 0.014757962 | 3.921766954 |
| hsa_circ_0102235_CBC1 | 0.009785221 | 2.30015936 |
| hsa-circRNA3390-3_CBC1 | 9.32533E-05 | 3.462591261 |
| hsa_circ_0097638_CBC1 | 0.000397482 | 2.369565395 |
| hsa_circ_0062836_CBC1 | 0.003804141 | 2.472131253 |
| hsa_circ_0029081_CBC1 | 0.007832304 | 2.46598956 |
| hsa-circRNA3542-2_CBC1 | 0.028616687 | 2.504936822 |
| hsa_circ_0040620_CBC1 | 0.00595561 | 3.295027559 |
| hsa-circRNA8500-3_CBC1 | 0.004622738 | 2.324840895 |
| hsa_circ_0134620_CBC1 | 0.000243015 | 2.275911657 |
| hsa-circRNA4002-2_CBC1 | 0.00688312 | 2.580904744 |
| hsa-circRNA9929-30_CBC1 | 8.28568E-05 | 5.071497584 |
| hsa_circ_0081398_CBC1 | 0.000739582 | 5.933266217 |
| hsa_circ_0005881_CBC1 | 7.17412E-06 | 2.727113369 |
| hsa_circ_0052581_CBC1 | 0.00032162 | 5.766733204 |
| hsa-circRNA7044-26_CBC1 | 0.000538674 | 4.658836946 |
| hsa_circ_0059713_CBC1 | 6.97102E-05 | 7.330816884 |
| hsa_circ_0063031_CBC1 | 9.39023E-06 | 4.017876628 |
| hsa_circ_0091719_CBC1 | 0.035840449 | 3.489281662 |
| hsa_circ_0105091_CBC1 | 0.00612163 | 2.401387354 |
| hsa-circRNA3279-12_CBC1 | 0.003946523 | 2.083011524 |
| hsa_circ_0135023_CBC1 | 0.046761645 | 2.068092328 |
| hsa_circ_0053017_CBC1 | 0.000159646 | 5.488727346 |
| hsa-circRNA13698-53_CBC1 | 0.030111933 | 3.973347663 |
| hsa-circRNA946-9_CBC1 | 0.00069407 | 2.736127022 |
| hsa_circ_0005403_CBC1 | 0.004253484 | 3.943108949 |
| hsa_circ_0029109_CBC1 | 0.002152897 | 2.290364194 |
| hsa_circ_0027012_CBC1 | 0.011907197 | 2.834183745 |
| hsa_circ_0016386_CBC1 | 0.002288412 | 7.089331398 |
| hsa_circ_0059403_CBC1 | 0.026922629 | 3.115420449 |
| hsa_circ_0050818_CBC1 | 0.002033312 | 2.380830551 |
| hsa_circ_0109298_CBC1 | 0.03825224 | 2.045960463 |
| hsa_circ_0080075_CBC1 | 0.000102241 | 2.118376057 |
| hsa_circ_0042105_CBC1 | 0.001688148 | 3.137342874 |
| hsa_circ_0049403_CBC1 | 2.3469E-05 | 7.724048173 |
| hsa-circRNA11393-16_CBC1 | 0.000273089 | 8.296385305 |
| hsa_circ_0000010_CBC1 | 0.017011344 | 2.024928762 |
| hsa-circRNA14265-44_CBC1 | 0.006067345 | 2.007384769 |
| hsa_circ_0022422_CBC1 | 0.000226207 | 3.151258956 |
| hsa-circRNA3507-1_CBC1 | 0.010928168 | 2.004983579 |
| hsa_circ_0009820_CBC1 | 0.006245556 | 2.099864018 |
| hsa-circRNA1257-35_CBC1 | 0.000273748 | 2.946277067 |
| hsa_circ_0052583_CBC1 | 0.000157613 | 6.123706818 |
| hsa_circ_0139349_CBC1 | 0.014788824 | 3.30801794 |
| hsa-circRNA617-14_CBC1 | 2.76681E-05 | 3.023896713 |
| hsa_circ_0047928_CBC1 | 0.014659092 | 2.620970053 |
| hsa_circ_0051976_CBC1 | 0.017205628 | 2.587603051 |
| hsa_circ_0051936_CBC1 | 0.002275218 | 3.362536045 |
| hsa-circRNA2719-15_CBC1 | 0.001208404 | 4.597382657 |
| hsa_circ_0007547_CBC1 | 0.001954484 | 5.667934767 |
| hsa-circRNA9920-34_CBC1 | 0.008482055 | 2.743135527 |
| hsa_circ_0101887_CBC1 | 0.038166882 | 3.12811774 |
| hsa-circRNA2330-43_CBC1 | 0.02905187 | 2.156418042 |
| hsa-circRNA8223-7_CBC1 | 0.016326341 | 2.014202566 |
| hsa_circ_0029505_CBC1 | 0.003177548 | 6.631465078 |
| hsa_circ_0003604_CBC1 | 0.000404065 | 2.667740509 |
| hsa_circ_0000308_CBC1 | 0.005281599 | 2.414330296 |
| hsa-circRNA15655-15_CBC1 | 0.006327921 | 3.609951875 |
| hsa-circRNA5028-29_CBC1 | 0.000164837 | 4.521911622 |
| hsa_circ_0031276_CBC1 | 0.01236131 | 2.19384504 |
| hsa_circ_0041061_CBC1 | 0.006763903 | 2.675340706 |
| hsa_circ_0126660_CBC1 | 0.003546712 | 3.100421234 |
| hsa_circ_0041993_CBC1 | 8.30922E-06 | 7.849598784 |
| hsa_circ_0084350_CBC1 | 0.001799134 | 2.317376464 |
| hsa-circRNA11084-6_CBC1 | 0.000353263 | 8.898734471 |
| hsa_circ_0041984_CBC1 | 0.000155733 | 2.046743716 |
| hsa_circ_0014761_CBC1 | 0.004034193 | 5.142375867 |
| hsa_circ_0064214_CBC1 | 0.014999375 | 2.89556477 |
| hsa_circ_0043534_CBC1 | 0.000218337 | 5.55341729 |
| hsa-circRNA10149-60_CBC1 | 9.69001E-05 | 4.764299499 |
| hsa_circ_0104338_CBC1 | 0.007172536 | 2.474592209 |
| hsa_circ_0140158_CBC1 | 0.002843275 | 3.698486459 |
| hsa_circ_0021738_CBC1 | 0.011951068 | 2.065837374 |
| hsa_circ_0040483_CBC1 | 4.54282E-05 | 3.211575106 |
| hsa_circ_0033655_CBC1 | 0.012204747 | 2.561055787 |
| hsa-circRNA11015-3_CBC1 | 0.004317981 | 2.343612994 |
| hsa-circRNA881-4_CBC1 | 0.002610944 | 2.443597262 |
| hsa_circ_0051720_CBC1 | 0.000474881 | 5.631294813 |
| hsa_circ_0015825_CBC1 | 0.000119266 | 19.23634762 |
| hsa-circRNA7413-15_CBC1 | 0.001303908 | 2.859996921 |
| hsa-circRNA3774-3_CBC1 | 0.00910936 | 2.294670303 |
| hsa-circRNA2506-3_CBC1 | 0.002239304 | 3.231598844 |
| hsa_circ_0029384_CBC1 | 0.002204099 | 2.808453153 |
| hsa_circ_0036742_CBC1 | 0.000339375 | 2.59183431 |
| hsa_circ_0015722_CBC1 | 0.045417449 | 2.014477035 |
| hsa_circ_0029114_CBC1 | 0.002664116 | 2.086014024 |
| hsa_circ_0088982_CBC1 | 0.002913682 | 2.031006525 |
| hsa-circRNA12057-16_CBC1 | 2.78056E-05 | 5.169896669 |
| hsa_circ_0106671_CBC1 | 0.0091452 | 2.070421251 |
| hsa_circ_0038923_CBC1 | 0.000100052 | 5.270695944 |
| hsa_circ_0029126_CBC1 | 0.009464343 | 2.972550222 |
| hsa-circRNA391-13_CBC1 | 0.022088619 | 4.155252485 |
| hsa_circ_0098260_CBC1 | 0.009995237 | 2.029831057 |
| hsa_circ_0083664_CBC1 | 0.00055333 | 2.999304385 |
| hsa_circ_0084362_CBC1 | 0.020534481 | 2.468368227 |
| hsa_circ_0035042_CBC1 | 0.013800334 | 2.270294901 |
| hsa_circ_0035490_CBC1 | 4.78378E-05 | 6.083084898 |
| hsa_circ_0135328_CBC1 | 0.008749655 | 2.758050062 |
| hsa_circ_0130129_CBC1 | 0.002934221 | 3.232854834 |
| hsa_circ_0041060_CBC1 | 0.001555834 | 3.114659178 |
| hsa-circRNA8881-13_CBC1 | 0.00115309 | 14.63668152 |
| hsa_circ_0049608_CBC1 | 0.000485736 | 4.949859275 |
| hsa_circ_0004250_CBC1 | 0.041571024 | 2.436015298 |
| hsa_circ_0007634_CBC1 | 0.021465093 | 2.345084233 |
| hsa_circ_0021484_CBC1 | 0.006477524 | 2.166155546 |
| hsa_circ_0036867_CBC1 | 0.04410358 | 2.973689084 |
| hsa_circ_0047685_CBC1 | 0.001375016 | 6.759930865 |
| hsa_circ_0044130_CBC1 | 0.000142524 | 13.45565629 |
| hsa_circ_0098771_CBC1 | 0.016884182 | 2.016788779 |
| hsa_circ_0002205_CBC1 | 0.021600181 | 3.20201577 |
| hsa_circ_0032202_CBC1 | 0.00158583 | 2.502857313 |
| hsa-circRNA15208-15_CBC1 | 0.000295476 | 2.088933545 |
| hsa_circ_0136673_CBC1 | 0.003358725 | 2.013062857 |
| hsa-circRNA11493-22_CBC1 | 0.009309792 | 5.887138875 |
| hsa_circ_0134617_CBC1 | 0.001899777 | 2.334274215 |
| hsa_circ_0063512_CBC1 | 0.000153014 | 2.22664453 |
| hsa_circ_0036875_CBC1 | 0.022572397 | 2.646442794 |
| hsa_circ_0035013_CBC1 | 0.010833927 | 2.647900686 |
| hsa_circ_0028547_CBC1 | 0.001151642 | 2.10736785 |
| hsa_circ_0106908_CBC1 | 0.000109006 | 6.170947882 |
| hsa-circRNA6200-18_CBC1 | 0.046094314 | 2.51763041 |
| hsa-circRNA7663-18_CBC1 | 0.000259594 | 2.558245329 |
| hsa_circ_0026367_CBC1 | 0.002059764 | 3.888490779 |
| hsa-circRNA9443-21_CBC1 | 0.020429027 | 2.799403757 |
| hsa_circ_0027109_CBC1 | 0.004211153 | 5.987935575 |
| hsa_circ_0032006_CBC1 | 0.014635656 | 2.460445791 |
| hsa_circ_0088963_CBC1 | 0.003074895 | 2.32834521 |
| hsa-circRNA8885-26_CBC1 | 0.001266288 | 9.742146113 |
| hsa_circ_0082507_CBC1 | 0.002105541 | 2.065328075 |
| hsa_circ_0004280_CBC1 | 0.000371496 | 4.121802821 |
| hsa_circ_0059876_CBC1 | 0.002432595 | 2.73965144 |
| hsa_circ_0125627_CBC1 | 0.00412369 | 3.499966759 |
| hsa_circ_0045020_CBC1 | 0.003657026 | 2.810306845 |
| hsa-circRNA5427-64_CBC1 | 0.00278829 | 4.826744223 |
| hsa_circ_0029523_CBC1 | 0.001078774 | 2.160816353 |
| hsa-circRNA4737-5_CBC1 | 0.032374365 | 2.006961411 |
| hsa_circ_0067706_CBC1 | 0.000672728 | 2.710063805 |
| hsa_circ_0113482_CBC1 | 0.02726778 | 4.683750425 |
| hsa-circRNA10187-43_CBC1 | 0.010425805 | 2.006502055 |
| hsa-circRNA10149-50_CBC1 | 0.000136543 | 6.32856607 |
| hsa_circ_0102836_CBC1 | 0.000613704 | 2.725546627 |
| hsa_circ_0026618_CBC1 | 8.78991E-05 | 2.172561238 |
| hsa_circ_0066952_CBC1 | 0.000647137 | 6.102074503 |
| hsa_circ_0029135_CBC1 | 0.002741828 | 3.225311326 |
| hsa-circRNA11873-3_CBC1 | 0.010841058 | 2.431625495 |
| hsa_circ_0018036_CBC1 | 0.009278533 | 2.249249062 |
| hsa_circ_0046677_CBC1 | 1.20799E-05 | 5.315106571 |
| hsa_circ_0031787_CBC1 | 0.006247003 | 2.40372769 |
| hsa_circ_0062262_CBC1 | 0.011191532 | 6.197622216 |
| hsa_circ_0041042_CBC1 | 0.000775946 | 4.061619897 |
| hsa-circRNA3109-8_CBC1 | 2.44463E-05 | 2.786315806 |
| hsa_circ_0061805_CBC1 | 0.001223326 | 2.16170405 |
| hsa_circ_0088906_CBC1 | 0.006980362 | 2.04681517 |
| hsa_circ_0022710_CBC1 | 0.026309173 | 2.340126537 |
| hsa-circRNA16051_CBC1 | 0.009526027 | 7.628501207 |
| hsa-circRNA3109-5_CBC1 | 0.000380649 | 2.92373617 |
| hsa_circ_0104536_CBC1 | 0.007099721 | 2.128017822 |
| hsa_circ_0073744_CBC1 | 0.003323299 | 3.438952741 |
| hsa_circ_0057201_CBC1 | 0.000476458 | 2.10897083 |
| hsa-circRNA3353-13_CBC1 | 0.023169556 | 2.319370074 |
| hsa_circ_0130751_CBC1 | 0.014814228 | 2.769759823 |
| hsa_circ_0090952_CBC1 | 0.028180661 | 3.307235001 |
| hsa-circRNA4973-15_CBC1 | 0.024540284 | 2.140622914 |
| hsa-circRNA6289-5_CBC1 | 0.001185924 | 2.416138019 |
| hsa_circ_0079869_CBC1 | 8.22577E-06 | 5.443785845 |
| hsa_circ_0056039_CBC1 | 2.58655E-05 | 9.392310217 |
| hsa-circRNA9996-1_CBC1 | 0.014800584 | 2.263820155 |
| hsa_circ_0043951_CBC1 | 0.004910333 | 5.887864755 |
| hsa_circ_0095899_CBC1 | 0.005833139 | 2.038207304 |
| hsa_circ_0040997_CBC1 | 0.009391659 | 2.683717562 |
| hsa-circRNA2719-4_CBC1 | 0.00101753 | 2.876880156 |
| hsa_circ_0119485_CBC1 | 0.000808424 | 4.112281803 |
| hsa_circ_0012366_CBC1 | 0.003390346 | 3.84117485 |
| hsa-circRNA12269-21_CBC1 | 8.38498E-06 | 5.897429852 |
| hsa_circ_0007425_CBC1 | 0.014095396 | 4.500311245 |
| hsa_circ_0088283_CBC1 | 0.006508855 | 2.179772728 |
| hsa_circ_0068018_CBC1 | 0.046747188 | 6.4612187 |
| hsa_circ_0073748_CBC1 | 0.00240025 | 5.756310234 |
| hsa_circ_0029092_CBC1 | 0.001924992 | 2.671575114 |
| hsa_circ_0045011_CBC1 | 0.002322042 | 2.288861118 |
| hsa_circ_0037539_CBC1 | 0.000294691 | 4.793885535 |
| hsa_circ_0012321_CBC1 | 0.006814875 | 3.041662825 |
| hsa_circ_0102864_CBC1 | 0.005195131 | 3.051830876 |
| hsa_circ_0084642_CBC1 | 0.020707606 | 2.262932996 |
| hsa_circ_0106445_CBC1 | 0.001240627 | 15.00364373 |
| hsa-circRNA16133-24_CBC1 | 2.79841E-05 | 3.248655345 |
| hsa_circ_0008926_CBC1 | 0.000507728 | 3.105931713 |
| hsa_circ_0057199_CBC1 | 0.015426529 | 2.091297141 |
| hsa-circRNA2719-37_CBC1 | 0.001296502 | 4.49031199 |
| hsa_circ_0041887_CBC1 | 0.025426959 | 2.636583563 |
| hsa_circ_0034580_CBC1 | 0.000293339 | 2.204907355 |
| hsa_circ_0088966_CBC1 | 0.000591554 | 2.22333279 |
| hsa_circ_0067168_CBC1 | 0.001827991 | 2.244463945 |
| hsa-circRNA11981-24_CBC1 | 0.000655842 | 2.660863957 |
| hsa_circ_0133412_CBC1 | 0.004799772 | 2.420307453 |
| hsa-circRNA5427-32_CBC1 | 0.00600784 | 2.675996804 |
| hsa_circ_0015818_CBC1 | 0.000281064 | 5.350183937 |
| hsa_circ_0012242_CBC1 | 0.004167475 | 2.125073048 |
| hsa_circ_0041733_CBC1 | 0.000869348 | 5.078038167 |
| hsa-circRNA7393-7_CBC1 | 0.001624827 | 5.023341326 |
| hsa_circ_0036056_CBC1 | 0.001694409 | 2.001531149 |
| hsa-circRNA10206-59_CBC1 | 0.010096599 | 2.760522779 |
| hsa_circ_0086012_CBC1 | 0.035215649 | 2.610550987 |
| hsa-circRNA15339-13_CBC1 | 0.011048476 | 2.049700972 |
| hsa-circRNA2246-5_CBC1 | 0.025237047 | 2.582958785 |
| hsa-circRNA11981-45_CBC1 | 5.57474E-05 | 3.852471137 |
| hsa_circ_0059715_CBC1 | 0.000463698 | 5.650102633 |
| hsa_circ_0085507_CBC1 | 0.001013249 | 2.040950335 |
| hsa-circRNA11981-51_CBC1 | 0.002626888 | 2.068282954 |
| hsa_circ_0048673_CBC1 | 0.006257347 | 4.152237656 |
| hsa_circ_0072452_CBC1 | 0.020681452 | 2.622864221 |
| hsa_circ_0088947_CBC1 | 7.91353E-05 | 2.131759738 |
| hsa_circ_0011521_CBC1 | 0.01399655 | 2.340981494 |
| hsa_circ_0122324_CBC1 | 0.014218845 | 4.960589289 |
| hsa_circ_0022843_CBC1 | 0.001682363 | 2.044138574 |
| hsa_circ_0003707_CBC1 | 0.005283339 | 3.486323363 |
| hsa_circ_0000868_CBC1 | 0.002327128 | 2.194471065 |
| hsa-circRNA6411-7_CBC1 | 0.004095003 | 3.402405294 |
| hsa_circ_0104342_CBC1 | 0.027049371 | 2.404864004 |
| hsa-circRNA10312-25_CBC1 | 0.005860303 | 3.084722493 |
| hsa_circ_0028683_CBC1 | 0.000439308 | 5.03770679 |
| hsa_circ_0063531_CBC1 | 0.000102407 | 2.270851396 |
| hsa_circ_0095020_CBC1 | 0.031587377 | 2.992379175 |
| hsa-circRNA4923-5_CBC1 | 0.019583511 | 2.296200973 |
| hsa_circ_0070509_CBC1 | 0.0248037 | 2.066729942 |
| hsa_circ_0098262_CBC1 | 0.006638342 | 2.171067487 |
| hsa-circRNA1396-6_CBC1 | 0.000240532 | 2.313971319 |
| hsa_circ_0022171_CBC1 | 0.0034484 | 4.304688825 |
| hsa_circ_0027925_CBC1 | 0.048659373 | 3.282260668 |
| hsa_circ_0041883_CBC1 | 0.000931686 | 2.010539209 |
| hsa_circ_0009989_CBC1 | 0.00491204 | 2.742276315 |
| hsa_circ_0107249_CBC1 | 0.001048927 | 2.432412913 |
| hsa_circ_0085994_CBC1 | 0.015190997 | 2.205661924 |
| hsa_circ_0043532_CBC1 | 0.000452191 | 5.076748541 |
| hsa_circ_0054613_CBC1 | 0.021776209 | 2.345002824 |
| hsa-circRNA3458-8_CBC1 | 2.40441E-05 | 2.62849881 |
| hsa_circ_0059722_CBC1 | 2.37971E-05 | 6.407338767 |
| hsa_circ_0061581_CBC1 | 0.010065559 | 3.088439543 |
| hsa_circ_0089602_CBC1 | 0.002657784 | 2.352210844 |
| hsa_circ_0041265_CBC1 | 0.036690443 | 2.09643171 |
| hsa-circRNA5427-61_CBC1 | 0.008589955 | 5.966362669 |
| hsa-circRNA1257-47_CBC1 | 0.000965987 | 2.456076586 |
| hsa-circRNA15389-7_CBC1 | 0.000280856 | 4.082828136 |
| hsa_circ_0061545_CBC1 | 0.004841438 | 2.565452958 |
| hsa_circ_0026606_CBC1 | 0.01636871 | 2.577721628 |
| hsa_circ_0005538_CBC1 | 0.006021692 | 2.008658536 |
| hsa_circ_0097686_CBC1 | 0.029434189 | 2.015201703 |
| hsa-circRNA10187-15_CBC1 | 0.014216709 | 2.040250812 |
| hsa_circ_0084482_CBC1 | 0.019762021 | 2.63006229 |
| hsa-circRNA11493-29_CBC1 | 0.000181057 | 4.345750536 |
| hsa_circ_0006779_CBC1 | 0.001493305 | 3.539592043 |
| hsa_circ_0003791_CBC1 | 9.36308E-05 | 2.270321122 |
| hsa_circ_0086748_CBC1 | 8.80881E-05 | 4.710541369 |
| hsa_circ_0124529_CBC1 | 0.040693037 | 3.151861175 |
| hsa_circ_0125979_CBC1 | 7.40779E-05 | 2.93173079 |
| hsa-circRNA12510-17_CBC1 | 0.006810163 | 2.454448847 |
| hsa_circ_0040490_CBC1 | 0.00017417 | 2.692954289 |
| hsa_circ_0102826_CBC1 | 0.005543085 | 2.037484545 |
| hsa_circ_0088985_CBC1 | 0.000216251 | 2.089835551 |
| hsa_circ_0134927_CBC1 | 0.027769952 | 2.03571502 |
| hsa_circ_0020489_CBC1 | 0.000189069 | 12.09095559 |
| hsa-circRNA7041-1_CBC1 | 8.82743E-05 | 3.218192993 |
| hsa_circ_0078998_CBC1 | 5.05322E-05 | 3.246526067 |
| hsa-circRNA1649-29_CBC1 | 6.14748E-05 | 3.221331264 |
| hsa_circ_0071653_CBC1 | 0.00439783 | 2.553770244 |
| hsa-circRNA15389-8_CBC1 | 0.009263627 | 4.730407877 |
| hsa_circ_0084765_CBC1 | 0.00277132 | 2.675656255 |
| hsa_circ_0022134_CBC1 | 0.001319066 | 2.018261869 |
| hsa_circ_0084978_CBC1 | 0.003376463 | 2.337570839 |
| hsa-circRNA11733-28_CBC1 | 0.014661593 | 2.007941126 |
| hsa-circRNA1649-24_CBC1 | 0.000223712 | 2.827098241 |
| hsa_circ_0008777_CBC1 | 0.001628549 | 2.757539118 |
| hsa_circ_0056030_CBC1 | 0.000159785 | 6.667743634 |
| hsa-circRNA4923-11_CBC1 | 0.020430711 | 2.429897878 |
| hsa-circRNA2719-22_CBC1 | 0.002094291 | 3.731224905 |
| hsa_circ_0135662_CBC1 | 0.003189936 | 4.610585667 |
| hsa-circRNA7275-25_CBC1 | 0.007417365 | 2.063264729 |
| hsa_circ_0036265_CBC1 | 0.032844064 | 2.361117863 |
| hsa_circ_0085461_CBC1 | 0.009493023 | 2.345904834 |
| hsa-circRNA5028-10_CBC1 | 0.000308976 | 6.920665898 |
| hsa_circ_0052242_CBC1 | 8.1559E-06 | 4.183304433 |
| hsa_circ_0051719_CBC1 | 0.000167971 | 6.976834807 |
| hsa_circ_0102517_CBC1 | 0.015243506 | 3.274911549 |
| hsa_circ_0067705_CBC1 | 0.000444736 | 2.574857188 |
| hsa_circ_0028436_CBC1 | 0.008833258 | 2.178927873 |
| hsa_circ_0067840_CBC1 | 0.000936623 | 4.801581215 |
| hsa_circ_0065851_CBC1 | 0.020293143 | 2.270909239 |
| hsa_circ_0098766_CBC1 | 0.003588921 | 2.591257082 |
| hsa-circRNA733-7_CBC1 | 0.007159033 | 4.308245295 |
| hsa-circRNA13165-13_CBC1 | 0.021053957 | 2.81653251 |
| hsa_circ_0060462_CBC1 | 3.74939E-05 | 4.51164878 |
| hsa_circ_0108845_CBC1 | 0.005128948 | 4.119868913 |
| hsa-circRNA617-7_CBC1 | 0.002269351 | 3.172518314 |
| hsa_circ_0025170_CBC1 | 0.000937888 | 3.31707442 |
| hsa_circ_0081399_CBC1 | 6.84366E-06 | 3.922368172 |
| hsa_circ_0059330_CBC1 | 0.003238344 | 5.311602178 |
| hsa-circRNA13520-14_CBC1 | 0.01292026 | 2.64147603 |
| hsa-circRNA8518-5_CBC1 | 0.005863257 | 2.086392504 |
| hsa-circRNA7183-8_CBC1 | 0.007456891 | 2.328522207 |
| hsa_circ_0088998_CBC1 | 0.000320936 | 2.132853797 |
| hsa_circ_0037810_CBC1 | 0.000527284 | 2.833036491 |
| hsa_circ_0094479_CBC1 | 0.002070973 | 5.391258752 |
| hsa_circ_0047948_CBC1 | 0.002488263 | 3.012838052 |
| hsa-circRNA6298-7_CBC1 | 0.002131847 | 2.522788458 |
| hsa_circ_0034598_CBC1 | 0.003083362 | 7.662756118 |
| hsa-circRNA11601-1_CBC1 | 0.002476768 | 2.260104077 |
| hsa_circ_0055671_CBC1 | 0.000103351 | 7.100166068 |
| hsa-circRNA1008-3_CBC1 | 0.002427597 | 3.047125675 |
| hsa-circRNA7228-6_CBC1 | 0.007837365 | 2.105034902 |
| hsa_circ_0034302_CBC1 | 0.000216161 | 2.887598975 |
| hsa_circ_0121607_CBC1 | 0.000906273 | 6.154601286 |
| hsa-circRNA14781-40_CBC1 | 0.002394599 | 3.051167269 |
| hsa_circ_0092947_CBC1 | 0.000282221 | 2.803942781 |
| hsa_circ_0027014_CBC1 | 0.000312203 | 2.16400486 |
| hsa-circRNA9487-65_CBC1 | 0.029305736 | 2.821807453 |
| hsa_circ_0032900_CBC1 | 3.23688E-05 | 2.892044126 |
| hsa_circ_0134220_CBC1 | 6.06631E-06 | 6.18990953 |
| hsa_circ_0036914_CBC1 | 3.52379E-05 | 6.567216971 |
| hsa_circ_0057242_CBC1 | 0.002399952 | 2.035006128 |
| hsa-circRNA15229-20_CBC1 | 0.000267793 | 3.576398303 |
| hsa-circRNA11493-23_CBC1 | 0.001612602 | 5.692640897 |
| hsa_circ_0088946_CBC1 | 9.83359E-05 | 2.198073475 |
| hsa_circ_0003143_CBC1 | 0.001946404 | 5.390356024 |
| hsa_circ_0125036_CBC1 | 0.00558901 | 2.439838719 |
| hsa_circ_0029195_CBC1 | 0.021551089 | 2.86403942 |
| hsa_circ_0036743_CBC1 | 0.000706516 | 2.432885948 |
| hsa_circ_0028595_CBC1 | 9.85633E-05 | 3.49173236 |
| hsa-circRNA7183-9_CBC1 | 0.002083795 | 2.042238643 |
| hsa_circ_0051977_CBC1 | 0.002319153 | 2.432160679 |
| hsa_circ_0000794_CBC1 | 0.012636388 | 3.772489402 |
| hsa_circ_0037521_CBC1 | 0.000229886 | 5.390369175 |
| hsa-circRNA626-24_CBC1 | 0.000677119 | 2.824435455 |
| hsa-circRNA2900-11_CBC1 | 0.002016398 | 2.177832829 |
| hsa_circ_0081401_CBC1 | 0.007392955 | 3.26627616 |
| hsa_circ_0025156_CBC1 | 0.002028287 | 3.234717277 |
| hsa_circ_0086759_CBC1 | 0.038239067 | 2.071229808 |
| hsa_circ_0032010_CBC1 | 0.006029564 | 2.524517291 |
| hsa_circ_0063035_CBC1 | 0.000107339 | 3.099086604 |
| hsa_circ_0012158_CBC1 | 0.000271581 | 7.531506904 |
| hsa-circRNA12269-3_CBC1 | 2.16065E-05 | 7.681521512 |
| hsa_circ_0009172_CBC1 | 0.033364262 | 2.012114875 |
| hsa-circRNA5427-45_CBC1 | 0.003632393 | 2.716679477 |
| hsa-circRNA11981-34_CBC1 | 0.000139404 | 2.379389637 |
| hsa_circ_0081552_CBC1 | 0.003351556 | 2.16023663 |
| hsa_circ_0133283_CBC1 | 0.002151969 | 3.202250757 |
| hsa_circ_0034562_CBC1 | 1.76685E-05 | 7.504888721 |
| hsa_circ_0078372_CBC1 | 0.019049436 | 2.004975202 |
| hsa-circRNA3109-3_CBC1 | 0.001025491 | 3.183540886 |
| hsa_circ_0064219_CBC1 | 0.001904723 | 6.769579794 |
| hsa-circRNA15993-22_CBC1 | 0.033165694 | 3.085906451 |
| hsa_circ_0049200_CBC1 | 0.0008289 | 2.673209059 |
| hsa_circ_0031782_CBC1 | 0.000516669 | 7.200997098 |
| hsa-circRNA10893-17_CBC1 | 8.50515E-05 | 5.349696706 |
| hsa_circ_0034600_CBC1 | 0.000981585 | 9.448712874 |
| hsa_circ_0035843_CBC1 | 0.007074034 | 2.609838418 |
| hsa_circ_0102163_CBC1 | 0.004355687 | 2.167463552 |
| hsa_circ_0029996_CBC1 | 0.009930321 | 3.04922492 |
| hsa_circ_0012159_CBC1 | 0.000190638 | 5.478371867 |
| hsa_circ_0017562_CBC1 | 0.04798643 | 2.062337289 |
| hsa_circ_0041053_CBC1 | 0.031447556 | 2.156486313 |
| hsa_circ_0017724_CBC1 | 0.006483449 | 2.42067348 |
| hsa_circ_0070108_CBC1 | 0.000570943 | 4.105731163 |
| hsa_circ_0129014_CBC1 | 0.022795275 | 2.829943497 |
| hsa_circ_0055547_CBC1 | 0.000277375 | 2.248648264 |
| hsa-circRNA2033-62_CBC1 | 0.001731251 | 3.525354102 |
| hsa_circ_0073743_CBC1 | 0.000209093 | 6.337957409 |
| hsa-circRNA5308-4_CBC1 | 0.001030626 | 3.235533944 |
| hsa-circRNA5427-62_CBC1 | 0.001542469 | 4.631799839 |
| hsa_circ_0049852_CBC1 | 0.004114661 | 8.160964198 |
| hsa_circ_0116641_CBC1 | 0.000499469 | 2.125465202 |
| hsa-circRNA14248-18_CBC1 | 0.000205623 | 2.235511478 |
| hsa_circ_0051491_CBC1 | 0.012497227 | 3.120790636 |
| hsa_circ_0040476_CBC1 | 0.000166621 | 3.433997411 |
| hsa_circ_0129074_CBC1 | 0.020458572 | 2.028448304 |
| hsa_circ_0118568_CBC1 | 0.002921435 | 2.059217855 |
| hsa-circRNA3009-1_CBC1 | 0.008823354 | 2.83766258 |
| hsa-circRNA14257-22_CBC1 | 0.020250091 | 2.085378773 |
| hsa_circ_0108995_CBC1 | 0.000226869 | 4.166446949 |
| hsa_circ_0048454_CBC1 | 0.000691163 | 2.047318916 |
| hsa_circ_0009840_CBC1 | 0.009086803 | 2.231243989 |
| hsa-circRNA1203-24_CBC1 | 0.003693462 | 2.002414088 |
| hsa-circRNA1781-13_CBC1 | 0.001461818 | 2.033335933 |
| hsa_circ_0031353_CBC1 | 0.000271325 | 2.004273459 |
| hsa-circRNA10149-43_CBC1 | 5.47105E-05 | 2.613643098 |
| hsa_circ_0093696_CBC1 | 0.039641768 | 2.456362304 |
| hsa-circRNA1463_CBC1 | 0.010503662 | 2.120748352 |
| hsa_circ_0043859_CBC1 | 0.000941788 | 2.061098274 |
| hsa_circ_0043858_CBC1 | 0.016283368 | 2.261406641 |
| hsa-circRNA8791-18_CBC1 | 0.003970951 | 2.789652959 |
| hsa-circRNA8492-2_CBC1 | 0.000668796 | 2.132987528 |
| hsa_circ_0070411_CBC1 | 0.035379085 | 2.935816796 |
| hsa_circ_0042572_CBC1 | 0.001482179 | 2.316368472 |
| hsa_circ_0038922_CBC1 | 0.000133024 | 3.136567654 |
| hsa_circ_0103432_CBC1 | 0.001381937 | 2.469398422 |
| hsa_circ_0046099_CBC1 | 0.006073969 | 2.119493438 |
| hsa-circRNA7970-51_CBC1 | 0.000207942 | 2.205757647 |
| hsa-circRNA3215-16_CBC1 | 0.000767445 | 2.076924159 |
| hsa_circ_0025643_CBC1 | 0.006912044 | 2.091448995 |
| hsa_circ_0014751_CBC1 | 0.015913552 | 2.101885591 |
| hsa_circ_0029775_CBC1 | 0.018459328 | 2.128413736 |
| hsa_circ_0008888_CBC1 | 0.000132943 | 3.441368295 |
| hsa-circRNA1649-23_CBC1 | 0.000814938 | 2.408525906 |
| hsa_circ_0088971_CBC1 | 0.005227002 | 2.610493458 |
| hsa_circ_0022418_CBC1 | 0.000182273 | 3.184650071 |
| hsa_circ_0137899_CBC1 | 0.007940956 | 2.161500787 |
| hsa_circ_0035043_CBC1 | 0.027674718 | 2.660439789 |
| hsa_circ_0036732_CBC1 | 0.004631304 | 2.483028967 |
| hsa_circ_0043954_CBC1 | 0.004861248 | 4.539433767 |
| hsa_circ_0097695_CBC1 | 0.001243036 | 2.243481806 |
| hsa_circ_0028697_CBC1 | 0.000950143 | 6.228827545 |
| hsa_circ_0102380_CBC1 | 0.006321556 | 3.366717655 |
| hsa-circRNA7836-29_CBC1 | 0.000103242 | 2.262202388 |
| hsa_circ_0085459_CBC1 | 0.01466219 | 2.115834945 |
| hsa_circ_0095264_CBC1 | 0.047664124 | 2.416459385 |
| hsa_circ_0039235_CBC1 | 0.018970445 | 3.547980344 |
| hsa_circ_0029522_CBC1 | 6.23163E-05 | 2.90925508 |
| hsa_circ_0044088_CBC1 | 1.87628E-05 | 3.379014777 |
| hsa_circ_0029090_CBC1 | 0.041730191 | 2.411158942 |
| hsa_circ_0106542_CBC1 | 0.027738008 | 3.246422709 |
| hsa_circ_0092043_CBC1 | 0.001004686 | 2.032536816 |
| hsa_circ_0041884_CBC1 | 0.000425073 | 2.296502757 |
| hsa_circ_0113445_CBC1 | 0.00204039 | 2.195220396 |
| hsa_circ_0031778_CBC1 | 0.012286198 | 2.539492598 |
| hsa_circ_0097453_CBC1 | 0.041162322 | 2.35168086 |
| hsa_circ_0054614_CBC1 | 0.013780206 | 3.238735688 |
| hsa-circRNA14838-113_CBC1 | 0.015175236 | 2.309395619 |
| hsa-circRNA813-7_CBC1 | 0.004837594 | 4.061100035 |
| hsa_circ_0133427_CBC1 | 0.003864806 | 2.154480901 |
| hsa_circ_0136186_CBC1 | 0.000889062 | 2.051329489 |
| hsa_circ_0012368_CBC1 | 0.001173884 | 2.795187183 |
| hsa_circ_0082837_CBC1 | 0.001187053 | 2.352682203 |
| hsa_circ_0049214_CBC1 | 0.00016641 | 4.331718312 |
| hsa_circ_0036706_CBC1 | 0.000934679 | 4.354783005 |
| hsa_circ_0023525_CBC1 | 0.043619706 | 2.158815929 |
| hsa-circRNA10149-45_CBC1 | 0.008800826 | 4.783107965 |
| hsa_circ_0062267_CBC1 | 0.001450309 | 3.034508229 |
| hsa-circRNA8518-25_CBC1 | 0.001476164 | 2.212096788 |
| hsa-circRNA3767-2_CBC1 | 0.007165365 | 2.005680835 |
| hsa_circ_0130472_CBC1 | 0.000797137 | 2.364884244 |
| hsa_circ_0041027_CBC1 | 0.002290915 | 5.367061252 |
| hsa_circ_0139912_CBC1 | 0.002173634 | 4.004967188 |
| hsa_circ_0082509_CBC1 | 0.001692748 | 2.432490833 |
| hsa_circ_0035486_CBC1 | 0.000201825 | 3.445964438 |
| hsa-circRNA5427-18_CBC1 | 0.00047031 | 3.653020169 |
| hsa_circ_0090637_CBC1 | 5.07348E-05 | 3.54429015 |
| hsa_circ_0122142_CBC1 | 0.008624154 | 2.634892635 |
| hsa_circ_0028704_CBC1 | 0.00330937 | 8.747249416 |
| hsa_circ_0086782_CBC1 | 0.021547355 | 2.398453788 |
| hsa_circ_0025041_CBC1 | 3.54279E-05 | 3.85830457 |
| hsa_circ_0066937_CBC1 | 0.016027846 | 2.164615572 |
| hsa-circRNA5333-4_CBC1 | 6.90434E-05 | 6.665606416 |
| hsa_circ_0059874_CBC1 | 0.012319756 | 2.165739088 |
| hsa_circ_0093083_CBC1 | 0.00061755 | 8.645121712 |
| hsa_circ_0077400_CBC1 | 0.009781273 | 3.816095126 |
| hsa_circ_0024689_CBC1 | 0.022734182 | 2.403641613 |
| hsa-circRNA7139-2_CBC1 | 0.011647807 | 2.394922937 |
| hsa-circRNA1396-9_CBC1 | 0.0008972 | 3.535219045 |
| hsa-circRNA15355-12_CBC1 | 0.002201077 | 2.313511139 |
| hsa_circ_0100760_CBC1 | 0.000172567 | 2.101531634 |
| hsa_circ_0036919_CBC1 | 0.003134171 | 5.31914119 |
| hsa_circ_0098270_CBC1 | 0.025117982 | 2.276434208 |
| hsa_circ_0012246_CBC1 | 0.001798252 | 2.130268329 |
| hsa_circ_0120400_CBC1 | 0.003235548 | 3.536780956 |
| hsa-circRNA9996-8_CBC1 | 0.009041595 | 2.339421995 |
| hsa-circRNA10149-26_CBC1 | 0.000782943 | 3.907580676 |
| hsa_circ_0125038_CBC1 | 0.002880528 | 2.223093116 |
| hsa_circ_0048349_CBC1 | 0.010343994 | 3.011070511 |
| hsa_circ_0072760_CBC1 | 2.51833E-05 | 8.125850737 |
| hsa_circ_0011950_CBC1 | 0.000872442 | 4.937446507 |
| hsa_circ_0097490_CBC1 | 0.005663219 | 2.31685222 |
| hsa-circRNA4973-10_CBC1 | 0.012194025 | 2.386794565 |
| hsa_circ_0066700_CBC1 | 0.03437271 | 2.330817149 |
| hsa_circ_0074324_CBC1 | 0.001527683 | 2.270297317 |
| hsa_circ_0027107_CBC1 | 0.040494671 | 2.215542991 |
| hsa-circRNA11175-1_CBC1 | 0.019609467 | 2.135629297 |
| hsa_circ_0138056_CBC1 | 0.003659188 | 2.316387992 |
| hsa_circ_0085410_CBC1 | 0.011574511 | 4.520647144 |
| hsa-circRNA12221_CBC1 | 0.027721406 | 2.148990868 |
| hsa_circ_0001395_CBC1 | 0.002687354 | 5.277101422 |
| hsa-circRNA2520-28_CBC1 | 0.000687968 | 9.093036553 |
| hsa_circ_0013306_CBC1 | 0.000996095 | 3.074274335 |
| hsa_circ_0083187_CBC1 | 0.000256289 | 5.212074835 |
| hsa_circ_0096005_CBC1 | 0.000692506 | 2.313577003 |
| hsa_circ_0022059_CBC1 | 0.002595077 | 2.662821962 |
| hsa_circ_0011007_CBC1 | 0.029271181 | 2.543277327 |
| hsa_circ_0051881_CBC1 | 0.046943061 | 2.724108146 |
| hsa-circRNA15428-9_CBC1 | 0.020612461 | 2.0426754 |
| hsa-circRNA10149-59_CBC1 | 0.00059738 | 3.124837152 |
| hsa_circ_0051731_CBC1 | 0.000165552 | 4.516675195 |
| hsa_circ_0086027_CBC1 | 0.013466592 | 3.332202391 |
| hsa_circ_0031770_CBC1 | 0.011992534 | 3.614345488 |
| hsa_circ_0061062_CBC1 | 0.031248956 | 2.188710437 |
| hsa_circ_0043547_CBC1 | 0.000371704 | 3.534648461 |
| hsa_circ_0110331_CBC1 | 0.005787648 | 2.097319812 |
| hsa-circRNA9158-25_CBC1 | 0.023390928 | 2.351290315 |
| hsa_circ_0059718_CBC1 | 0.00280461 | 6.00409645 |
| hsa_circ_0002015_CBC1 | 0.001232578 | 2.121773266 |
| hsa_circ_0064584_CBC1 | 0.002773065 | 2.015046017 |
| hsa_circ_0083780_CBC1 | 0.009197864 | 8.606439506 |
| hsa-circRNA5149-11_CBC1 | 0.000476327 | 3.407588569 |
| hsa-circRNA15330-21_CBC1 | 0.000902698 | 2.758624895 |
| hsa-circRNA11137-13_CBC1 | 0.019623457 | 2.337569958 |
| hsa_circ_0050719_CBC1 | 0.001759014 | 2.074955942 |
| hsa_circ_0043542_CBC1 | 2.22858E-05 | 7.04100062 |
| hsa_circ_0135420_CBC1 | 0.02744687 | 2.090992546 |
| hsa-circRNA3385-7_CBC1 | 0.000488534 | 2.187251713 |
| hsa_circ_0088952_CBC1 | 0.000125848 | 2.635171071 |
| hsa-circRNA1291-5_CBC1 | 0.001200698 | 4.196314535 |
| hsa_circ_0037532_CBC1 | 0.001334334 | 2.379651344 |
| hsa_circ_0038925_CBC1 | 0.009150656 | 2.149934717 |
| hsa_circ_0090638_CBC1 | 0.004103382 | 2.53924545 |
| hsa-circRNA2313-8_CBC1 | 0.000328625 | 2.606678607 |
| hsa_circ_0008617_CBC1 | 0.006174112 | 3.673134808 |
| hsa_circ_0064230_CBC1 | 0.001976622 | 4.772866176 |
| hsa_circ_0097846_CBC1 | 9.21783E-05 | 2.411162774 |
| hsa_circ_0045010_CBC1 | 0.011914567 | 2.647369268 |
| hsa_circ_0074741_CBC1 | 0.037943098 | 2.267131242 |
| hsa_circ_0012608_CBC1 | 0.004054911 | 3.118436651 |
| hsa_circ_0098798_CBC1 | 0.003775538 | 2.496354196 |
| hsa-circRNA7754-2_CBC1 | 0.019650671 | 2.019943122 |
| hsa_circ_0041877_CBC1 | 0.000944771 | 2.210156493 |
| hsa_circ_0010872_CBC1 | 0.000910464 | 2.682320597 |
| hsa_circ_0111433_CBC1 | 0.015904835 | 2.266303464 |
| hsa_circ_0048980_CBC1 | 0.000157221 | 2.677208016 |
| hsa_circ_0025172_CBC1 | 0.001168917 | 4.035011359 |
| hsa-circRNA2910-7_CBC1 | 1.30173E-05 | 6.931863046 |
| hsa_circ_0120405_CBC1 | 0.02944107 | 3.172991792 |
| hsa_circ_0041067_CBC1 | 0.000846848 | 3.073034147 |
| hsa_circ_0023000_CBC1 | 0.003134391 | 2.130422569 |
| hsa_circ_0081182_CBC1 | 0.003599929 | 2.088650537 |
| hsa-circRNA14089-1_CBC1 | 0.00244317 | 13.62758535 |
| hsa-circRNA5884-2_CBC1 | 0.005105234 | 3.786753411 |
| hsa_circ_0110327_CBC1 | 0.000421351 | 2.973664046 |
| hsa_circ_0013200_CBC1 | 0.003929576 | 3.012512558 |
| hsa_circ_0082187_CBC1 | 0.009075098 | 2.767445201 |
| hsa-circRNA6428_CBC1 | 1.98443E-05 | 3.967435454 |
| hsa_circ_0000768_CBC1 | 0.002348226 | 2.077683631 |
| hsa_circ_0027013_CBC1 | 0.00077734 | 3.441208477 |
| hsa-circRNA5936-16_CBC1 | 0.000501123 | 6.625491472 |
| hsa_circ_0076437_CBC1 | 0.000795744 | 2.012211158 |
| hsa_circ_0028680_CBC1 | 3.81347E-05 | 6.124238724 |
| hsa_circ_0090628_CBC1 | 5.22116E-06 | 3.231822307 |
| hsa_circ_0123862_CBC1 | 0.001567805 | 3.418200339 |
| hsa_circ_0111434_CBC1 | 0.01311209 | 2.258746412 |
| hsa-circRNA2033-68_CBC1 | 0.003615174 | 2.624389114 |
| hsa_circ_0021915_CBC1 | 0.006296384 | 2.062730721 |
| hsa_circ_0097648_CBC1 | 0.033529393 | 2.537856099 |
| hsa_circ_0099131_CBC1 | 0.008010673 | 3.448307808 |
| hsa-circRNA4049-15_CBC1 | 0.000197223 | 3.811015935 |
| hsa_circ_0119089_CBC1 | 0.009600883 | 2.554635642 |
| hsa_circ_0088326_CBC1 | 0.003155063 | 2.037601185 |
| hsa-circRNA3767-9_CBC1 | 0.000558064 | 2.173970614 |
| hsa-circRNA10312-14_CBC1 | 0.001372212 | 3.20207226 |
| hsa_circ_0015762_CBC1 | 0.003888322 | 8.910530212 |
| hsa_circ_0069701_CBC1 | 0.005196651 | 2.057619167 |
| hsa_circ_0044087_CBC1 | 2.33274E-05 | 3.749775125 |
| hsa_circ_0113867_CBC1 | 0.04529836 | 2.063746369 |
| hsa_circ_0064207_CBC1 | 0.00028575 | 6.082997186 |
| hsa-circRNA11838-1_CBC1 | 0.004321567 | 2.637457436 |
| hsa-circRNA13515-26_CBC1 | 0.016803124 | 2.317111197 |
| hsa-circRNA10712-4_CBC1 | 0.022681586 | 2.514016511 |
| hsa_circ_0115128_CBC1 | 0.026696235 | 3.376179473 |
| hsa_circ_0006829_CBC1 | 0.046090052 | 2.07469808 |
| hsa_circ_0067428_CBC1 | 0.015591245 | 2.422931942 |
| hsa-circRNA13330-6_CBC1 | 0.008565855 | 4.47506072 |
| hsa-circRNA10887-6_CBC1 | 0.000426204 | 2.646113613 |
| hsa-circRNA1036-5_CBC1 | 0.001355107 | 2.298247167 |
| hsa_circ_0064856_CBC1 | 0.010347664 | 2.874674911 |
| hsa-circRNA2730-3_CBC1 | 0.001730476 | 8.160988459 |
| hsa-circRNA11838-55_CBC1 | 0.036503388 | 2.080826154 |
| hsa_circ_0140152_CBC1 | 0.007095684 | 2.808908692 |
| hsa_circ_0045846_CBC1 | 0.000397072 | 2.651211738 |
| hsa_circ_0116801_CBC1 | 8.66165E-06 | 8.292102148 |
| hsa_circ_0029118_CBC1 | 0.005460943 | 2.703003405 |
| hsa_circ_0025036_CBC1 | 6.23041E-05 | 3.39191813 |
| hsa-circRNA2290_CBC1 | 0.010346873 | 2.386037701 |
| hsa_circ_0072759_CBC1 | 6.02479E-06 | 5.905872135 |
| hsa_circ_0060459_CBC1 | 0.000126301 | 3.410345168 |
| hsa_circ_0077408_CBC1 | 0.027821136 | 2.290358471 |
| hsa-circRNA8791-3_CBC1 | 0.001216522 | 11.26376777 |
| hsa_circ_0015169_CBC1 | 0.015772973 | 2.525794639 |
| hsa_circ_0076747_CBC1 | 0.009253342 | 3.065473873 |
| hsa-circRNA10149-31_CBC1 | 3.39683E-05 | 6.298735672 |
| hsa-circRNA1760-3_CBC1 | 0.007096364 | 2.47192583 |
| hsa-circRNA10466-1_CBC1 | 0.045251267 | 2.188841346 |
| hsa_circ_0049674_CBC1 | 0.001155366 | 2.420157268 |
| hsa_circ_0050793_CBC1 | 0.000119035 | 2.168296784 |
| hsa_circ_0034608_CBC1 | 0.000477443 | 4.915529522 |
| hsa_circ_0032485_CBC1 | 0.030078067 | 2.668454527 |
| hsa-circRNA9487-67_CBC1 | 0.022907295 | 2.271413137 |
| hsa_circ_0011275_CBC1 | 0.016373402 | 2.789474768 |
| hsa_circ_0045025_CBC1 | 0.010056988 | 2.439118148 |
| hsa-circRNA6498-2_CBC1 | 0.001405556 | 2.681159001 |
| hsa-circRNA5806-8_CBC1 | 0.001858413 | 2.60748584 |
| hsa_circ_0050309_CBC1 | 0.00218625 | 2.263224118 |
| hsa_circ_0126601_CBC1 | 0.037305961 | 2.10583799 |
| hsa_circ_0044128_CBC1 | 5.14762E-05 | 7.594958228 |
| hsa_circ_0074308_CBC1 | 0.005123291 | 2.075254874 |
| hsa_circ_0068021_CBC1 | 0.001041755 | 3.402821773 |
| hsa_circ_0047686_CBC1 | 5.86439E-05 | 7.527217549 |
| hsa_circ_0050707_CBC1 | 4.4995E-05 | 6.355147535 |
| hsa-circRNA2313-21_CBC1 | 0.006328621 | 2.143402773 |
| hsa_circ_0016385_CBC1 | 0.00371155 | 5.081221089 |
| hsa_circ_0007322_CBC1 | 0.017965312 | 2.346365316 |
| hsa-circRNA5213-12_CBC1 | 0.019521965 | 2.205535801 |
| hsa_circ_0056025_CBC1 | 6.02616E-05 | 10.82162696 |
| hsa_circ_0034604_CBC1 | 0.001946484 | 3.669937282 |
| hsa_circ_0079863_CBC1 | 0.000105664 | 7.121291553 |
| hsa_circ_0046101_CBC1 | 0.001630286 | 2.570905398 |
| hsa-circRNA9158-46_CBC1 | 0.014223709 | 4.740233605 |
| hsa_circ_0004853_CBC1 | 0.009748754 | 2.736820065 |
| hsa_circ_0063034_CBC1 | 0.000907193 | 2.421281238 |
| hsa_circ_0009822_CBC1 | 0.00022475 | 2.211357227 |
| hsa-circRNA14257-20_CBC1 | 0.002674497 | 2.038693832 |
| hsa-circRNA9333-9_CBC1 | 1.71668E-05 | 15.06137311 |
| hsa_circ_0043701_CBC1 | 0.014165175 | 2.094536905 |
| hsa-circRNA15389-3_CBC1 | 0.002961688 | 3.697215433 |
| hsa_circ_0122585_CBC1 | 0.000301385 | 4.636891513 |
| hsa_circ_0020490_CBC1 | 1.40417E-05 | 6.777546241 |
| hsa-circRNA9443-18_CBC1 | 0.000324211 | 5.591290022 |
| hsa_circ_0090950_CBC1 | 1.59547E-05 | 11.93904944 |
| hsa_circ_0102363_CBC1 | 0.001049884 | 3.606003852 |
| hsa_circ_0032896_CBC1 | 0.000741264 | 2.153818851 |
| hsa-circRNA281-3_CBC1 | 0.001558649 | 5.17828824 |
| hsa_circ_0082300_CBC1 | 0.000364483 | 3.171343881 |
| hsa-circRNA5308-16_CBC1 | 0.003641103 | 2.062885136 |
| hsa-circRNA5886-35_CBC1 | 0.000356629 | 2.017260117 |
| hsa-circRNA2520-39_CBC1 | 0.000927281 | 9.733828137 |
| hsa_circ_0016674_CBC1 | 0.000550909 | 2.022074615 |
| hsa-circRNA7663-22_CBC1 | 0.009221817 | 2.780204176 |
| hsa-circRNA13820-3_CBC1 | 0.046908918 | 2.405854436 |
| hsa-circRNA12452-1_CBC1 | 0.000350204 | 2.952296076 |
| hsa_circ_0046765_CBC1 | 0.002214492 | 2.817698253 |
| hsa_circ_0038933_CBC1 | 0.005436618 | 2.158904296 |
| hsa_circ_0027447_CBC1 | 0.007203817 | 2.432602479 |
| hsa-circRNA3215-44_CBC1 | 0.000550672 | 2.100517172 |
| hsa_circ_0050703_CBC1 | 0.000252212 | 4.839351404 |
| hsa_circ_0108999_CBC1 | 0.000119876 | 2.687694481 |
| hsa_circ_0068318_CBC1 | 0.000729691 | 2.01463153 |
| hsa-circRNA2033-42_CBC1 | 0.011846972 | 2.521028016 |
| hsa-circRNA9443-17_CBC1 | 0.000446105 | 4.339416997 |
| hsa_circ_0074843_CBC1 | 8.11061E-05 | 4.24143233 |
| hsa_circ_0125860_CBC1 | 0.006445223 | 2.596060617 |
| hsa_circ_0070556_CBC1 | 4.81559E-05 | 12.59539853 |
| hsa_circ_0079034_CBC1 | 0.011401426 | 2.247999948 |
| hsa_circ_0043174_CBC1 | 0.011975364 | 2.207434852 |
| hsa_circ_0059720_CBC1 | 9.67577E-06 | 5.55121617 |
| hsa-circRNA8092-11_CBC1 | 0.002366348 | 7.753619485 |
| hsa_circ_0128497_CBC1 | 0.000824372 | 3.265129368 |
| hsa_circ_0046676_CBC1 | 3.84341E-06 | 5.582997566 |
| hsa-circRNA2719-9_CBC1 | 0.002563597 | 4.385197135 |
| hsa_circ_0059716_CBC1 | 9.59262E-05 | 14.70911375 |
| hsa_circ_0082542_CBC1 | 0.000412785 | 2.157347904 |
| hsa-circRNA427-2_CBC1 | 0.001204138 | 3.976315566 |
| hsa-circRNA11372-1_CBC1 | 0.000924697 | 4.469034556 |
| hsa-circRNA11838-11_CBC1 | 0.007626339 | 2.373467545 |
| hsa_circ_0126179_CBC1 | 0.015733885 | 2.070616853 |
| hsa-circRNA11838-13_CBC1 | 0.008180558 | 2.619089339 |
| hsa-circRNA4049-44_CBC1 | 0.000891612 | 4.66099804 |
| hsa_circ_0119083_CBC1 | 0.031845396 | 2.37090665 |
| hsa_circ_0098269_CBC1 | 0.003596857 | 2.999407094 |
| hsa_circ_0064215_CBC1 | 0.005569034 | 4.962524484 |
| hsa_circ_0126442_CBC1 | 0.003014536 | 3.829088148 |
| hsa_circ_0065027_CBC1 | 0.003836653 | 8.002819414 |
| hsa_circ_0015771_CBC1 | 0.000482776 | 12.07633632 |
| hsa-circRNA2520-27_CBC1 | 7.50593E-05 | 6.810713732 |
| hsa_circ_0002427_CBC1 | 0.00893044 | 2.7094492 |
| hsa_circ_0124204_CBC1 | 0.007972519 | 2.274217621 |
| hsa-circRNA6201-9_CBC1 | 2.46309E-05 | 6.878674545 |
| hsa_circ_0075961_CBC1 | 0.00060824 | 16.7903732 |
| hsa_circ_0088780_CBC1 | 0.006525185 | 2.022396424 |
| hsa_circ_0053156_CBC1 | 0.00132009 | 2.173000686 |
| hsa_circ_0012617_CBC1 | 0.001289595 | 2.013608808 |
| hsa_circ_0037623_CBC1 | 0.009331992 | 2.125529401 |
| hsa_circ_0094042_CBC1 | 0.021649114 | 2.665627404 |
| hsa-circRNA5059-20_CBC1 | 0.00349424 | 2.009082409 |
| hsa-circRNA391-18_CBC1 | 0.002373714 | 3.195786785 |
| hsa_circ_0113715_CBC1 | 0.000555347 | 2.031042778 |
| hsa_circ_0083182_CBC1 | 0.004294222 | 3.907824536 |
| hsa_circ_0059885_CBC1 | 0.015860672 | 2.093722965 |
| hsa_circ_0084381_CBC1 | 0.001800704 | 2.015921478 |
| hsa_circ_0064240_CBC1 | 0.000484011 | 4.596161607 |
| hsa_circ_0009148_CBC1 | 0.016980672 | 2.463322703 |
| hsa_circ_0047931_CBC1 | 0.000280427 | 2.346060807 |
| hsa_circ_0081415_CBC1 | 3.58359E-05 | 2.736820404 |
| hsa-circRNA2502-5_CBC1 | 0.001773932 | 2.703067635 |
| hsa_circ_0064212_CBC1 | 0.000609182 | 5.315905626 |
| hsa_circ_0126555_CBC1 | 0.01551571 | 2.313373751 |
| hsa_circ_0008317_CBC1 | 0.002792391 | 6.063069826 |
| hsa_circ_0018985_CBC1 | 0.001321796 | 2.435568932 |
| hsa_circ_0018516_CBC1 | 0.002523563 | 3.17874463 |
| hsa_circ_0059729_CBC1 | 0.000216647 | 8.585817724 |
| hsa_circ_0104882_CBC1 | 0.011649089 | 5.511309175 |
| hsa_circ_0023845_CBC1 | 0.000907343 | 3.069279186 |
| hsa-circRNA3353-12_CBC1 | 0.033550085 | 3.086904554 |
| hsa_circ_0002149_CBC1 | 2.57441E-05 | 3.346752853 |
| hsa_circ_0006831_CBC1 | 0.004464625 | 2.485250833 |
| hsa_circ_0081410_CBC1 | 0.00018656 | 3.347656533 |
| hsa_circ_0060235_CBC1 | 0.033308268 | 2.447797472 |
| hsa_circ_0074322_CBC1 | 0.00440071 | 2.104326194 |
| hsa_circ_0081471_CBC1 | 0.006111907 | 2.469990863 |
| hsa_circ_0113650_CBC1 | 0.002790919 | 2.383690908 |
| hsa-circRNA8092-5_CBC1 | 0.001140162 | 6.124536204 |
| hsa_circ_0050709_CBC1 | 0.000333659 | 8.592900882 |
| hsa_circ_0088278_CBC1 | 0.000167249 | 2.128382091 |
| hsa_circ_0028438_CBC1 | 0.014936319 | 3.303022053 |
| hsa_circ_0007223_CBC1 | 1.89445E-05 | 8.125099971 |
| hsa_circ_0036695_CBC1 | 0.000331168 | 4.258735375 |
| hsa_circ_0067166_CBC1 | 0.001181064 | 3.722277479 |
| hsa_circ_0091260_CBC1 | 0.010064826 | 3.211529525 |
| hsa_circ_0050688_CBC1 | 0.003342307 | 2.105928039 |
| hsa_circ_0039604_CBC1 | 0.001516513 | 2.530723315 |
| hsa_circ_0035635_CBC1 | 0.024115582 | 2.002348381 |
| hsa-circRNA10312-17_CBC1 | 2.09797E-06 | 2.791383177 |
| hsa_circ_0058986_CBC1 | 0.034563221 | 2.3171487 |
| hsa_circ_0022130_CBC1 | 0.000414337 | 2.14449303 |
| hsa-circRNA2527-4_CBC1 | 0.001737019 | 4.080551573 |
| hsa_circ_0003543_CBC1 | 0.000103901 | 5.830164657 |
| hsa_circ_0036264_CBC1 | 0.033073847 | 2.186491052 |
| hsa_circ_0072691_CBC1 | 0.001136373 | 3.290260699 |
| hsa_circ_0015822_CBC1 | 0.00119749 | 4.866882601 |
| hsa_circ_0111196_CBC1 | 0.000544764 | 2.231933364 |
| hsa_circ_0029139_CBC1 | 0.002542902 | 2.723539897 |
| hsa_circ_0084438_CBC1 | 0.025508527 | 2.160859215 |
| hsa_circ_0035485_CBC1 | 0.000204167 | 5.98671398 |
| hsa_circ_0133423_CBC1 | 0.018183839 | 2.209233175 |
| hsa_circ_0017195_CBC1 | 0.013072579 | 3.67898921 |
| hsa_circ_0002592_CBC1 | 0.003898316 | 2.216580714 |
| hsa-circRNA7836-5_CBC1 | 0.000335434 | 2.061211682 |
| hsa_circ_0074010_CBC1 | 0.014640809 | 2.012200591 |
| hsa_circ_0060211_CBC1 | 0.000277873 | 3.241968062 |
| hsa-circRNA946-4_CBC1 | 0.01236803 | 3.937367903 |
| hsa-circRNA3220_CBC1 | 0.002201234 | 3.95377332 |
| hsa_circ_0139881_CBC1 | 0.002638253 | 4.058295877 |
| hsa-circRNA2520-4_CBC1 | 0.000621273 | 7.684407368 |
| hsa_circ_0034619_CBC1 | 0.003564791 | 4.229814274 |
| hsa-circRNA8791-8_CBC1 | 0.000172684 | 8.315588794 |
| hsa_circ_0133417_CBC1 | 0.005304617 | 2.518263963 |
| hsa_circ_0008335_CBC1 | 0.003497266 | 2.39951528 |
| hsa_circ_0079338_CBC1 | 0.042089963 | 2.154471926 |
| hsa_circ_0013376_CBC1 | 0.022501184 | 2.499642721 |
| hsa-circRNA4547-12_CBC1 | 7.57231E-05 | 10.3047822 |
| hsa_circ_0003883_CBC1 | 0.017963263 | 3.050786583 |
| hsa_circ_0116798_CBC1 | 3.33964E-05 | 12.60541299 |
| hsa_circ_0032943_CBC1 | 0.003945078 | 2.421889516 |
| hsa_circ_0049115_CBC1 | 0.001022055 | 6.142995154 |
| hsa_circ_0076749_CBC1 | 0.010177195 | 2.920022901 |
| hsa-circRNA9989-5_CBC1 | 0.005852047 | 3.99044755 |
| hsa_circ_0030524_CBC1 | 0.018470246 | 2.213833409 |
| hsa_circ_0082498_CBC1 | 0.004267544 | 2.076611613 |
| hsa-circRNA249-8_CBC1 | 0.020888343 | 2.130244705 |
| hsa_circ_0061634_CBC1 | 0.001668688 | 2.93066173 |
| hsa_circ_0070687_CBC1 | 0.032161646 | 3.125916068 |
| hsa_circ_0036740_CBC1 | 0.000226153 | 2.297793952 |
| hsa-circRNA2033-11_CBC1 | 0.003536315 | 2.821300867 |
| hsa-circRNA1008-5_CBC1 | 0.014292205 | 3.557207043 |
| hsa-circRNA11065-2_CBC1 | 0.00036814 | 2.411866165 |
| hsa_circ_0016380_CBC1 | 0.006356201 | 5.402767444 |
| hsa_circ_0021537_CBC1 | 0.000780903 | 5.762508856 |
| hsa_circ_0043538_CBC1 | 5.79253E-05 | 4.878923395 |
| hsa_circ_0097917_CBC1 | 0.025046928 | 2.960475736 |
| hsa_circ_0131602_CBC1 | 0.006568058 | 3.12151162 |
| hsa_circ_0002023_CBC1 | 0.004819864 | 3.282245186 |
| hsa-circRNA3792-18_CBC1 | 0.013861953 | 2.712500981 |
| hsa_circ_0102830_CBC1 | 0.003688091 | 2.798812209 |
| hsa_circ_0013726_CBC1 | 3.59959E-05 | 3.743320692 |
| hsa_circ_0021920_CBC1 | 0.000338996 | 2.303121896 |
| hsa-circRNA9443-7_CBC1 | 0.041933456 | 3.418742015 |
| hsa-circRNA2719-14_CBC1 | 0.00011034 | 5.611255415 |
| hsa_circ_0101921_CBC1 | 0.002821525 | 2.090039385 |
| hsa_circ_0017794_CBC1 | 0.028834646 | 5.25448265 |
| hsa_circ_0015978_CBC1 | 0.014969892 | 5.433367157 |
| hsa_circ_0049197_CBC1 | 1.50772E-05 | 4.338301181 |
| hsa_circ_0059734_CBC1 | 6.20675E-05 | 8.978709886 |
| hsa_circ_0102389_CBC1 | 0.002986374 | 4.251263336 |
| hsa-circRNA5535-5_CBC1 | 0.017010014 | 4.254850058 |
| hsa-circRNA5149-15_CBC1 | 0.002344547 | 3.008893245 |
| hsa-circRNA11372-4_CBC1 | 0.006790299 | 4.171068583 |
| hsa_circ_0091297_CBC1 | 0.011483432 | 2.005120972 |
| hsa_circ_0067290_CBC1 | 0.000522912 | 2.102610759 |
| hsa_circ_0076741_CBC1 | 0.000362415 | 4.548445726 |
| hsa_circ_0029511_CBC1 | 0.004158006 | 3.493553373 |
| hsa_circ_0010020_CBC1 | 0.049067613 | 3.100081424 |
| hsa_circ_0111661_CBC1 | 0.00070329 | 7.476673966 |
| hsa_circ_0056029_CBC1 | 0.000473386 | 9.166844172 |
| hsa-circRNA7836-33_CBC1 | 0.000812341 | 2.206902944 |
| hsa-circRNA1649-33_CBC1 | 0.000684098 | 3.534308489 |
| hsa-circRNA1030-23_CBC1 | 0.001973561 | 4.337606324 |
| hsa_circ_0081403_CBC1 | 0.000633613 | 2.938691517 |
| hsa-circRNA8791-15_CBC1 | 0.000110014 | 7.5615857 |
| hsa_circ_0088988_CBC1 | 3.0103E-07 | 2.566348564 |
| hsa_circ_0045416_CBC1 | 2.93238E-05 | 2.818934304 |
| hsa_circ_0128429_CBC1 | 0.027183483 | 2.049754368 |
| hsa_circ_0007279_CBC1 | 0.01994252 | 2.14415248 |
| hsa_circ_0111626_CBC1 | 0.0002373 | 5.59482199 |
| hsa-circRNA5796-2_CBC1 | 0.000225901 | 6.686929107 |
| hsa_circ_0049793_CBC1 | 0.033915652 | 2.25128128 |
| hsa_circ_0100118_CBC1 | 0.036848447 | 2.643510378 |
| hsa_circ_0095563_CBC1 | 2.52446E-06 | 2.837036141 |
| hsa-circRNA280-1_CBC1 | 9.75741E-05 | 13.76918811 |
| hsa-circRNA2866-50_CBC1 | 0.000395525 | 2.060726625 |
| hsa_circ_0110325_CBC1 | 8.9384E-05 | 2.186159948 |
| hsa_circ_0061793_CBC1 | 0.010041329 | 2.540226706 |
| hsa-circRNA882-3_CBC1 | 0.000313984 | 3.675534339 |
| hsa_circ_0064255_CBC1 | 0.000343292 | 10.20556961 |
| hsa_circ_0056375_CBC1 | 0.002226429 | 2.01637095 |
| hsa_circ_0088975_CBC1 | 2.71814E-05 | 2.335754953 |
| hsa_circ_0059727_CBC1 | 3.03132E-05 | 8.821995676 |
| hsa_circ_0084348_CBC1 | 0.005203335 | 2.034252089 |
| hsa-circRNA14781-32_CBC1 | 0.00599374 | 2.283676582 |
| hsa_circ_0028675_CBC1 | 6.17547E-05 | 3.491540831 |
| hsa_circ_0012923_CBC1 | 0.000948258 | 8.220291104 |
| hsa-circRNA10312-5_CBC1 | 0.025322721 | 3.063476353 |
| hsa_circ_0001925_CBC1 | 0.001387324 | 10.57621397 |
| hsa_circ_0088933_CBC1 | 0.000139574 | 2.418745803 |
| hsa-circRNA10893-11_CBC1 | 0.000122277 | 5.413784667 |
| hsa-circRNA3029-11_CBC1 | 0.01451187 | 2.083882654 |
| hsa-circRNA13698-58_CBC1 | 0.002279728 | 2.456322984 |
| hsa_circ_0064247_CBC1 | 0.000284113 | 5.243604971 |
| hsa_circ_0122582_CBC1 | 0.000172263 | 4.912586497 |
| hsa_circ_0012371_CBC1 | 0.000842253 | 3.436945423 |
| hsa_circ_0066208_CBC1 | 0.019081943 | 2.515544406 |
| hsa-circRNA9957-19_CBC1 | 0.001506651 | 2.391800084 |
| hsa_circ_0087793_CBC1 | 0.010699114 | 2.721931626 |
| hsa_circ_0060214_CBC1 | 0.004855865 | 2.203327882 |
| hsa_circ_0090624_CBC1 | 0.002114032 | 4.010863368 |
| hsa_circ_0080957_CBC1 | 0.014801449 | 2.07713342 |
| hsa_circ_0071679_CBC1 | 0.000128691 | 7.475233897 |
| hsa-circRNA743-7_CBC1 | 0.000129988 | 5.818178764 |
| hsa_circ_0026196_CBC1 | 0.000107793 | 5.340166088 |
| hsa_circ_0088305_CBC1 | 0.004753889 | 3.145242169 |
| hsa_circ_0063044_CBC1 | 5.51637E-05 | 3.19540533 |
| hsa_circ_0088978_CBC1 | 0.001422816 | 3.192294449 |
| hsa_circ_0030369_CBC1 | 0.011784105 | 4.109480864 |
| hsa_circ_0088957_CBC1 | 0.002856 | 2.685561247 |
| hsa_circ_0061942_CBC1 | 0.003153519 | 2.116511468 |
| hsa_circ_0062266_CBC1 | 0.001827856 | 4.502758499 |
| hsa-circRNA12113-4_CBC1 | 0.007660978 | 2.182460886 |
| hsa-circRNA7215-2_CBC1 | 0.003428756 | 3.493215596 |
| hsa-circRNA1594-6_CBC1 | 0.033572033 | 2.151773764 |
| hsa_circ_0032819_CBC1 | 0.017769662 | 3.772879899 |
| hsa_circ_0037541_CBC1 | 0.000264611 | 2.644125201 |
| hsa_circ_0076669_CBC1 | 0.003351071 | 2.480574177 |
| hsa_circ_0074524_CBC1 | 0.000820191 | 2.178673826 |
| hsa-circRNA2866-133_CBC1 | 0.001890303 | 2.605846682 |
| hsa-circRNA2097-12_CBC1 | 0.0021364 | 3.253572537 |
| hsa_circ_0052397_CBC1 | 0.00068227 | 3.789519343 |
| hsa-circRNA858-11_CBC1 | 0.04431582 | 4.123924591 |
| hsa-circRNA2033-97_CBC1 | 0.000212562 | 3.27814765 |
| hsa-circRNA7275-5_CBC1 | 0.001882784 | 2.05217479 |
| hsa-circRNA264-8_CBC1 | 0.000165901 | 5.460594795 |
| hsa_circ_0032171_CBC1 | 0.000309496 | 4.416936461 |
| hsa-circRNA3897-8_CBC1 | 0.002544503 | 3.898386482 |
| hsa-circRNA118-7_CBC1 | 0.000252407 | 2.1187729 |
| hsa-circRNA16277-2_CBC1 | 0.003084116 | 2.042011054 |
| hsa_circ_0012248_CBC1 | 0.02095571 | 2.37274585 |
| hsa_circ_0046098_CBC1 | 0.002034498 | 2.464987663 |
| hsa_circ_0032000_CBC1 | 0.033062349 | 2.186007639 |
| hsa_circ_0005345_CBC1 | 0.001610866 | 2.695881435 |
| hsa_circ_0000410_CBC1 | 0.003040018 | 3.337499223 |
| hsa_circ_0006357_CBC1 | 0.001286963 | 2.32043872 |
| hsa_circ_0023846_CBC1 | 0.003334351 | 4.820872457 |
| hsa_circ_0064203_CBC1 | 7.82637E-05 | 3.865871141 |
| hsa_circ_0086751_CBC1 | 5.76825E-05 | 8.527142993 |
| hsa-circRNA7228-7_CBC1 | 0.00998784 | 2.099867607 |
| hsa-circRNA2097-19_CBC1 | 0.00217692 | 8.110624818 |
| hsa_circ_0013510_CBC1 | 0.009379989 | 2.566394844 |
| hsa-circRNA1008-4_CBC1 | 0.002550436 | 2.344492001 |
| hsa-circRNA7663-35_CBC1 | 0.016803941 | 2.340585676 |
| hsa-circRNA733-18_CBC1 | 0.001582108 | 8.355286745 |
| hsa-circRNA7836-38_CBC1 | 0.001256928 | 2.054990983 |
| hsa-circRNA8786-14_CBC1 | 0.046673126 | 2.1118921 |
| hsa_circ_0084211_CBC1 | 0.016335448 | 2.065368883 |
| hsa-circRNA10312-19_CBC1 | 0.016851774 | 3.07041438 |
| hsa_circ_0113464_CBC1 | 0.016109617 | 2.255510453 |
| hsa-circRNA5884-6_CBC1 | 0.002046593 | 2.7715876 |
| hsa_circ_0070148_CBC1 | 0.024837408 | 2.507907351 |
| hsa-circRNA12677-65_CBC1 | 6.75561E-05 | 9.23233036 |
| hsa-circRNA8272-13_CBC1 | 0.008976237 | 2.070184615 |
| hsa-circRNA5996-19_CBC1 | 0.037170416 | 2.590059869 |
| hsa-circRNA11838-20_CBC1 | 0.022844415 | 2.216047623 |
| hsa_circ_0056158_CBC1 | 0.003128974 | 3.932664737 |
| hsa-circRNA8500-41_CBC1 | 0.000438841 | 4.171836505 |
| hsa_circ_0012161_CBC1 | 0.002394638 | 7.837988388 |
| hsa_circ_0043949_CBC1 | 0.000922196 | 6.874744157 |
| hsa_circ_0041026_CBC1 | 0.003364681 | 4.880517608 |
| hsa_circ_0034323_CBC1 | 0.00205332 | 2.283686821 |
| hsa-circRNA15988-10_CBC1 | 0.004260742 | 2.794142284 |
| hsa_circ_0049794_CBC1 | 0.002156979 | 2.208863477 |
| hsa-circRNA12953-2_CBC1 | 0.020942931 | 2.808230673 |
| hsa_circ_0064216_CBC1 | 0.00177554 | 3.442420325 |
| hsa-circRNA3353-6_CBC1 | 0.029092072 | 3.500285167 |
| hsa-circRNA5308-17_CBC1 | 0.000192537 | 3.391330902 |
| hsa_circ_0015769_CBC1 | 0.000215986 | 12.40822028 |
| hsa_circ_0059878_CBC1 | 0.003693566 | 2.331185045 |
| hsa_circ_0059402_CBC1 | 0.011956539 | 2.264346331 |
| hsa-circRNA1401-5_CBC1 | 0.001019492 | 2.597585813 |
| hsa_circ_0070918_CBC1 | 0.037708882 | 4.09105688 |
| hsa_circ_0041005_CBC1 | 7.52634E-05 | 2.670498368 |
| hsa_circ_0009051_CBC1 | 0.011247871 | 2.906964432 |
| hsa-circRNA7836-89_CBC1 | 2.49242E-05 | 2.276688849 |
| hsa-circRNA3792-1_CBC1 | 0.03948025 | 2.520007998 |
| hsa-circRNA71-3_CBC1 | 0.039910281 | 2.159788518 |
| hsa-circRNA11981-12_CBC1 | 0.000217061 | 4.134611392 |
| hsa-circRNA10887-4_CBC1 | 0.00045702 | 2.784786429 |
| hsa-circRNA4737-3_CBC1 | 0.004499355 | 2.911390938 |
| hsa_circ_0090946_CBC1 | 0.000281293 | 9.150780551 |
| hsa-circRNA9555-15_CBC1 | 0.003658537 | 2.117427784 |
| hsa-circRNA12452-2_CBC1 | 0.000458396 | 2.135786221 |
| hsa_circ_0052244_CBC1 | 5.09586E-06 | 3.153571548 |
| hsa_circ_0026592_CBC1 | 0.006935897 | 3.464201729 |
| hsa_circ_0000216_CBC1 | 0.001090493 | 5.42213636 |
| hsa_circ_0040989_CBC1 | 0.002289884 | 2.590086446 |
| hsa_circ_0122579_CBC1 | 0.020394826 | 3.902096659 |
| hsa_circ_0103037_CBC1 | 0.002565892 | 2.366976859 |
| hsa-circRNA6901-10_CBC1 | 0.002269348 | 2.034031549 |
| hsa-circRNA5059-52_CBC1 | 0.001106034 | 2.096380099 |
| hsa_circ_0082518_CBC1 | 0.01715555 | 2.64452021 |
| hsa_circ_0105416_CBC1 | 0.000943686 | 4.802251383 |
| hsa_circ_0034570_CBC1 | 0.000168029 | 20.74464306 |
| hsa-circRNA391-11_CBC1 | 0.004451813 | 4.008558179 |
| hsa_circ_0052243_CBC1 | 8.15235E-06 | 3.775244441 |
| hsa_circ_0120428_CBC1 | 0.015573175 | 2.420836823 |
| hsa_circ_0020318_CBC1 | 0.019755144 | 2.411325146 |
| hsa_circ_0004819_CBC1 | 0.004228014 | 2.699987884 |
| hsa_circ_0093723_CBC1 | 0.00039591 | 3.5122342 |
| hsa-circRNA1808_CBC1 | 0.000240515 | 2.766153031 |
| hsa_circ_0029516_CBC1 | 0.000399796 | 2.287481337 |
| hsa-circRNA10149-48_CBC1 | 6.32312E-05 | 10.23953772 |
| hsa-circRNA8885-23_CBC1 | 0.00147914 | 15.19030976 |
| hsa_circ_0114890_CBC1 | 6.02724E-06 | 7.861159372 |
| hsa_circ_0076664_CBC1 | 0.002972026 | 2.325990262 |
| hsa_circ_0079873_CBC1 | 0.00015588 | 5.64319519 |
| hsa-circRNA2639-6_CBC1 | 0.000148809 | 3.952671607 |
| hsa-circRNA2729-1_CBC1 | 0.006445962 | 3.103474027 |
| hsa-circRNA13577-2_CBC1 | 0.000139316 | 3.405558947 |
| hsa-circRNA11233-12_CBC1 | 0.00090893 | 2.149796084 |
| hsa_circ_0076634_CBC1 | 0.00487619 | 2.633547764 |
| hsa-circRNA7445-13_CBC1 | 0.000668285 | 2.793912352 |
| hsa_circ_0133420_CBC1 | 0.002895482 | 2.029064841 |
| hsa-circRNA10149-58_CBC1 | 0.000443791 | 4.685053593 |
| hsa_circ_0043959_CBC1 | 0.012527368 | 4.248246044 |
| hsa-circRNA2771-3_CBC1 | 0.002300173 | 2.533787812 |
| hsa_circ_0088309_CBC1 | 4.81699E-05 | 4.080359938 |
| hsa-circRNA11588-12_CBC1 | 0.021455902 | 2.329017455 |
| hsa_circ_0045207_CBC1 | 0.012219287 | 2.225335385 |
| hsa-circRNA11493-44_CBC1 | 0.031002876 | 3.307191626 |
| hsa_circ_0062890_CBC1 | 0.002523531 | 2.305895275 |
| hsa-circRNA3575-2_CBC1 | 0.004463409 | 2.010099306 |
| hsa_circ_0061631_CBC1 | 0.001091429 | 3.598010659 |
| hsa-circRNA13671-6_CBC1 | 0.001094423 | 7.519867332 |
| hsa_circ_0139453_CBC1 | 0.005206551 | 6.64085758 |
| hsa_circ_0090642_CBC1 | 0.002292614 | 2.810494464 |
| hsa_circ_0123866_CBC1 | 0.000889363 | 4.043770652 |
| hsa-circRNA7777-2_CBC1 | 9.13103E-05 | 2.560825122 |
| hsa-circRNA15162-47_CBC1 | 0.00352126 | 2.936103096 |
| hsa_circ_0128334_CBC1 | 0.000232577 | 2.626576902 |
| hsa-circRNA8964-26_CBC1 | 0.000608304 | 2.1608497 |
| hsa_circ_0061210_CBC1 | 0.000720352 | 3.414060311 |
| hsa_circ_0049720_CBC1 | 0.002833935 | 13.70027388 |
| hsa-circRNA11242-104_CBC1 | 0.039340566 | 2.231104894 |
| hsa-circRNA2536-10_CBC1 | 8.61998E-05 | 7.101342622 |
| hsa_circ_0102360_CBC1 | 0.002781221 | 4.197539095 |
| hsa-circRNA6298-2_CBC1 | 0.035197698 | 2.794529509 |
| hsa-circRNA7836-25_CBC1 | 0.013453603 | 2.580140983 |
| hsa_circ_0065391_CBC1 | 0.01585495 | 2.075618215 |
| hsa-circRNA2033-40_CBC1 | 0.001360177 | 2.81728342 |
| hsa_circ_0134294_CBC1 | 0.001533211 | 2.739196352 |
| hsa_circ_0040503_CBC1 | 0.00479569 | 2.676870052 |
| hsa_circ_0070211_CBC1 | 0.037755998 | 2.357057685 |
| hsa_circ_0117824_CBC1 | 0.00506025 | 3.355869238 |
| hsa_circ_0004298_CBC1 | 0.000532729 | 2.400138643 |
| hsa-circRNA7859-75_CBC1 | 0.018658754 | 2.030544696 |
| hsa_circ_0010022_CBC1 | 0.042566074 | 2.664435257 |
| hsa-circRNA7859-25_CBC1 | 0.004969149 | 2.045932251 |
| hsa_circ_0135138_CBC1 | 0.005962998 | 2.85848097 |
| hsa_circ_0024904_CBC1 | 0.000633145 | 4.554202921 |
| hsa-circRNA3215-21_CBC1 | 0.000421512 | 2.341369681 |
| hsa-circRNA4923-12_CBC1 | 0.031245817 | 2.07323355 |
| hsa-circRNA1510-8_CBC1 | 4.96023E-05 | 2.075196588 |
| hsa-circRNA7836-105_CBC1 | 0.000121679 | 3.08098681 |
| hsa_circ_0102835_CBC1 | 4.35032E-05 | 3.213418853 |
| hsa_circ_0135022_CBC1 | 0.044457608 | 3.600470842 |
| hsa_circ_0034611_CBC1 | 0.001485608 | 8.118724966 |
| hsa-circRNA9154-25_CBC1 | 0.001510958 | 2.321958016 |
| hsa_circ_0028708_CBC1 | 0.000156932 | 12.32327915 |
| hsa_circ_0050261_CBC1 | 0.04571955 | 2.058592329 |
| hsa-circRNA2033-119_CBC1 | 0.000299248 | 2.427987849 |
| hsa-circRNA5886-23_CBC1 | 0.000335184 | 2.988197025 |
| hsa-circRNA2639-4_CBC1 | 0.001729475 | 3.591291726 |
| hsa-circRNA2097-5_CBC1 | 0.00545472 | 5.387749854 |
| hsa_circ_0030371_CBC1 | 5.68161E-06 | 2.663481355 |
| hsa_circ_0043861_CBC1 | 0.024672094 | 2.343272247 |
| hsa_circ_0060469_CBC1 | 0.001228904 | 3.091276014 |
| hsa_circ_0029112_CBC1 | 0.008990743 | 2.097136659 |
| hsa_circ_0050683_CBC1 | 0.000214753 | 6.663554531 |
| hsa_circ_0098845_CBC1 | 0.00873601 | 4.276774376 |
| hsa_circ_0045481_CBC1 | 0.000397653 | 2.065185593 |
| hsa_circ_0094366_CBC1 | 0.002538368 | 3.670445318 |
| hsa_circ_0025168_CBC1 | 0.000123505 | 2.546670718 |
| hsa_circ_0132654_CBC1 | 0.005504237 | 2.151191948 |
| hsa-circRNA11458-12_CBC1 | 0.00254852 | 4.918393747 |
| hsa_circ_0113632_CBC1 | 0.021217289 | 3.257403761 |
| hsa_circ_0038739_CBC1 | 0.00720445 | 2.186179151 |
| hsa-circRNA9867-3_CBC1 | 0.043476625 | 2.219214744 |
| hsa_circ_0045265_CBC1 | 0.007656684 | 3.311180247 |
| hsa_circ_0115127_CBC1 | 0.028825445 | 2.918429616 |
| hsa_circ_0119651_CBC1 | 0.000406348 | 6.472150533 |
| hsa_circ_0072454_CBC1 | 0.023275506 | 2.735312876 |
| hsa-circRNA3588-5_CBC1 | 0.001331216 | 4.591701778 |
| hsa-circRNA15136_CBC1 | 0.001127524 | 3.28771341 |
| hsa-circRNA2520-9_CBC1 | 0.000138751 | 8.040355934 |
| hsa_circ_0032822_CBC1 | 0.008504781 | 3.125959129 |
| hsa_circ_0065392_CBC1 | 0.002722721 | 2.18216904 |
| hsa-circRNA14257-4_CBC1 | 0.004165355 | 2.122012894 |
| hsa-circRNA5806-13_CBC1 | 0.006601199 | 3.079088157 |
| hsa_circ_0064224_CBC1 | 0.000201348 | 9.298367919 |
| hsa_circ_0126440_CBC1 | 0.014231394 | 3.035215457 |
| hsa-circRNA1594-10_CBC1 | 0.009197701 | 3.763580856 |
| hsa_circ_0003093_CBC1 | 0.03139567 | 2.334334302 |
| hsa_circ_0109113_CBC1 | 0.003923746 | 2.454867405 |
| hsa_circ_0077946_CBC1 | 0.006907401 | 7.161775227 |
| hsa_circ_0113484_CBC1 | 0.000817727 | 6.270626091 |
| hsa_circ_0031431_CBC1 | 0.016351382 | 2.276345032 |
| hsa_circ_0100098_CBC1 | 0.002263148 | 2.751632295 |
| hsa_circ_0006102_CBC1 | 0.00094865 | 2.243281633 |
| hsa_circ_0061122_CBC1 | 0.023596527 | 3.156938392 |
| hsa-circRNA7663-5_CBC1 | 0.004555405 | 3.081566703 |
| hsa_circ_0026066_CBC1 | 0.002544462 | 2.105411026 |
| hsa-circRNA4965-23_CBC1 | 0.000140469 | 6.916045482 |
| hsa-circRNA14781-26_CBC1 | 0.011028893 | 2.682415038 |
| hsa_circ_0086373_CBC1 | 8.99759E-06 | 2.197029382 |
| hsa_circ_0010959_CBC1 | 0.004006156 | 3.587381387 |
| hsa-circRNA1790-15_CBC1 | 8.90026E-05 | 3.293181777 |
| hsa_circ_0094676_CBC1 | 0.022658186 | 2.898415174 |
| hsa_circ_0015939_CBC1 | 0.001556866 | 2.487673308 |
| hsa_circ_0020207_CBC1 | 0.000173209 | 2.678501999 |
| hsa_circ_0029127_CBC1 | 0.00067048 | 4.715359526 |
| hsa_circ_0023529_CBC1 | 0.02965066 | 2.174303463 |
| hsa_circ_0012160_CBC1 | 7.545E-06 | 5.668244707 |
| hsa_circ_0015284_CBC1 | 0.002771502 | 2.040166877 |
| hsa-circRNA858-18_CBC1 | 0.005034595 | 9.73982296 |
| hsa-circRNA5041-1_CBC1 | 6.4817E-05 | 6.23980123 |
| hsa-circRNA1231-3_CBC1 | 0.005236493 | 2.006482932 |
| hsa-circRNA1378-8_CBC1 | 0.026746823 | 2.028656508 |
| hsa_circ_0091241_CBC1 | 0.009997875 | 4.347414538 |
| hsa_circ_0132641_CBC1 | 0.00354738 | 2.667870956 |
| hsa_circ_0028129_CBC1 | 0.042316213 | 2.097604642 |
| hsa_circ_0040996_CBC1 | 0.00019769 | 4.857062618 |
| hsa_circ_0016363_CBC1 | 7.6528E-05 | 12.80832134 |
| hsa-circRNA7787-15_CBC1 | 0.004612659 | 2.310562111 |
| hsa-circRNA2502-15_CBC1 | 0.000267615 | 5.805727194 |
| hsa_circ_0033325_CBC1 | 0.010589857 | 2.082067683 |
| hsa_circ_0006506_CBC1 | 0.016582108 | 2.165636306 |
| hsa-circRNA6201-16_CBC1 | 0.000395331 | 7.349285186 |
| hsa_circ_0070555_CBC1 | 0.003027072 | 17.25764888 |
| hsa_circ_0085458_CBC1 | 0.008338019 | 2.106490091 |
| hsa_circ_0070101_CBC1 | 0.000590288 | 2.427559565 |
| hsa-circRNA1030-25_CBC1 | 0.000335432 | 6.941562091 |
| hsa-circRNA8881-9_CBC1 | 0.001134092 | 15.89105946 |
| hsa-circRNA16274-8_CBC1 | 0.001709045 | 4.850272318 |
| hsa_circ_0098259_CBC1 | 0.012376054 | 2.021097038 |
| hsa_circ_0057896_CBC1 | 0.011197545 | 2.053921702 |
| hsa_circ_0034561_CBC1 | 0.000208812 | 7.357512626 |
| hsa-circRNA11766-7_CBC1 | 0.000364648 | 2.838025787 |
| hsa_circ_0093452_CBC1 | 0.003423581 | 2.410600129 |
| hsa_circ_0014767_CBC1 | 6.62651E-05 | 7.88220187 |
| hsa-circRNA13787-4_CBC1 | 0.000136546 | 2.705425756 |
| hsa_circ_0079147_CBC1 | 0.001649325 | 2.228876193 |
| hsa_circ_0082294_CBC1 | 0.001730905 | 2.331960757 |
| hsa_circ_0015830_CBC1 | 0.000106902 | 14.20594587 |
| hsa_circ_0029099_CBC1 | 0.002019789 | 2.990605663 |
| hsa_circ_0028689_CBC1 | 8.05748E-06 | 8.197277204 |
| hsa_circ_0102870_CBC1 | 0.018914805 | 2.312736152 |
| hsa-circRNA9572-1_CBC1 | 3.35176E-05 | 2.426240805 |
| hsa-circRNA13520-8_CBC1 | 0.003339144 | 6.508997335 |
| hsa-circRNA2033-117_CBC1 | 0.004264505 | 2.729587167 |
| hsa_circ_0033135_CBC1 | 0.000425468 | 2.548677334 |
| hsa-circRNA9222-3_CBC1 | 0.003440548 | 3.873505454 |
| hsa-circRNA11981-61_CBC1 | 0.000133546 | 2.990978651 |
| hsa-circRNA2033-12_CBC1 | 0.002390032 | 2.26011044 |
| hsa_circ_0111666_CBC1 | 0.001261736 | 4.735600573 |
| hsa_circ_0091424_CBC1 | 0.008584976 | 2.352153618 |
| hsa-circRNA1291-38_CBC1 | 0.002248958 | 2.92879426 |
| hsa_circ_0106446_CBC1 | 0.001654698 | 4.614343789 |
| hsa_circ_0031978_CBC1 | 0.000641824 | 5.526018156 |
| hsa-circRNA10964-20_CBC1 | 0.000930272 | 2.209938576 |
| hsa-circRNA11595-19_CBC1 | 0.002968012 | 2.578613263 |
| hsa_circ_0102398_CBC1 | 0.000132989 | 3.735450434 |
| hsa_circ_0106541_CBC1 | 0.022809419 | 3.671626406 |
| hsa_circ_0137894_CBC1 | 0.023229387 | 2.419889149 |
| hsa_circ_0108844_CBC1 | 0.014457701 | 2.384874367 |
| hsa_circ_0022815_CBC1 | 0.002793063 | 2.445889202 |
| hsa_circ_0005413_CBC1 | 0.001420939 | 3.158962993 |
| hsa_circ_0073759_CBC1 | 0.001383035 | 2.05012153 |
| hsa-circRNA12953-5_CBC1 | 0.000619599 | 5.245951042 |
| hsa_circ_0036053_CBC1 | 0.002207498 | 2.06963559 |
| hsa-circRNA10206-25_CBC1 | 0.000198021 | 9.188736288 |
| hsa-circRNA8500-34_CBC1 | 0.001580832 | 3.37071977 |
| hsa_circ_0107289_CBC1 | 0.046483248 | 2.108151092 |
| hsa_circ_0072054_CBC1 | 0.000315407 | 2.435775174 |
| hsa_circ_0056157_CBC1 | 0.000297112 | 4.457989424 |
| hsa_circ_0048675_CBC1 | 0.005019352 | 3.485013445 |
| hsa_circ_0095427_CBC1 | 0.011504025 | 2.238788977 |
| hsa_circ_0003975_CBC1 | 0.001584763 | 3.025884545 |
| hsa-circRNA4049-51_CBC1 | 2.71628E-05 | 6.77563802 |
| hsa_circ_0081400_CBC1 | 0.022418994 | 2.714836427 |
| hsa-circRNA16246-3_CBC1 | 0.00135791 | 2.453711742 |
| hsa-circRNA10776-3_CBC1 | 0.033962412 | 2.202269571 |
| hsa_circ_0006686_CBC1 | 7.8854E-06 | 5.626215064 |
| hsa-circRNA1412-4_CBC1 | 0.017028497 | 2.155048914 |
| hsa_circ_0043177_CBC1 | 0.002914847 | 3.362943429 |
| hsa_circ_0045483_CBC1 | 0.000598941 | 3.165828381 |
| hsa_circ_0016457_CBC1 | 0.002174698 | 7.821480927 |
| hsa_circ_0103474_CBC1 | 0.000139722 | 11.1989676 |
| hsa-circRNA11521-15_CBC1 | 0.000485767 | 2.306483347 |
| hsa_circ_0118878_CBC1 | 0.010136829 | 2.913792743 |
| hsa_circ_0049171_CBC1 | 0.000510976 | 2.019645591 |
| hsa_circ_0120181_CBC1 | 0.002390837 | 2.82955337 |
| hsa_circ_0038734_CBC1 | 0.049419667 | 2.177201047 |
| hsa-circRNA4361-2_CBC1 | 0.000655934 | 6.626908496 |
| hsa-circRNA3279-9_CBC1 | 0.000625457 | 2.779450134 |
| hsa_circ_0051812_CBC1 | 0.002141261 | 2.607174932 |
| hsa_circ_0032901_CBC1 | 4.78178E-05 | 2.866027884 |
| hsa_circ_0121419_CBC1 | 0.016949128 | 3.964959581 |
| hsa_circ_0003559_CBC1 | 0.002064172 | 2.498611039 |
| hsa_circ_0068962_CBC1 | 0.018801681 | 2.699966201 |
| hsa-circRNA2033-51_CBC1 | 0.008889483 | 3.910995128 |
| hsa_circ_0009843_CBC1 | 0.007212783 | 2.298466328 |
| hsa-circRNA6498-4_CBC1 | 0.000641738 | 2.209497267 |
| hsa_circ_0029115_CBC1 | 0.001220369 | 2.20912881 |
| hsa_circ_0036185_CBC1 | 0.000748746 | 2.008905017 |
| hsa_circ_0046105_CBC1 | 0.001234505 | 2.697368733 |
| hsa-circRNA15902-15_CBC1 | 0.023865794 | 2.789010297 |
| hsa_circ_0038636_CBC1 | 0.000136545 | 11.19692978 |
| hsa_circ_0081404_CBC1 | 9.58408E-06 | 3.30415132 |
| hsa_circ_0088930_CBC1 | 0.002492891 | 3.003356308 |
| hsa_circ_0018984_CBC1 | 0.004225175 | 3.853529525 |
| hsa_circ_0076750_CBC1 | 0.018516287 | 2.915192164 |
| hsa-circRNA7445-18_CBC1 | 0.009292394 | 2.560817262 |
| hsa_circ_0073946_CBC1 | 0.004492293 | 2.196137414 |
| hsa_circ_0133664_CBC1 | 0.009407981 | 5.004330281 |
| hsa_circ_0001990_CBC1 | 0.016403382 | 3.078448893 |
| hsa_circ_0044831_CBC1 | 0.000302987 | 2.504507988 |
| hsa-circRNA3458-9_CBC1 | 4.38258E-05 | 3.237557352 |
| hsa_circ_0032817_CBC1 | 0.007771383 | 2.191647179 |
| hsa-circRNA13465-2_CBC1 | 7.23822E-05 | 6.734257748 |
| hsa-circRNA1988-15_CBC1 | 0.00136185 | 4.347196315 |
| hsa-circRNA2101_CBC1 | 0.007485417 | 3.793680569 |
| hsa_circ_0022816_CBC1 | 0.009878109 | 2.396897618 |
| hsa_circ_0081418_CBC1 | 0.00014348 | 3.021403481 |
| hsa_circ_0059737_CBC1 | 0.000898939 | 12.39584661 |
| hsa_circ_0080074_CBC1 | 0.003761079 | 2.065844023 |
| hsa_circ_0082299_CBC1 | 0.001269108 | 2.839183521 |
| hsa_circ_0076021_CBC1 | 0.000534768 | 4.22428746 |
| hsa_circ_0130414_CBC1 | 0.024644848 | 2.205422821 |
| hsa_circ_0011584_CBC1 | 0.020489318 | 3.759428337 |
| hsa-circRNA2329-6_CBC1 | 0.000304817 | 4.167404827 |
| hsa_circ_0005291_CBC1 | 0.007517767 | 2.759639901 |
| hsa_circ_0137503_CBC1 | 0.003109598 | 3.170829204 |
| hsa_circ_0041996_CBC1 | 0.023576884 | 2.161309288 |
| hsa_circ_0006676_CBC1 | 0.001568384 | 2.727547193 |
| hsa_circ_0128084_CBC1 | 3.17251E-05 | 14.10230106 |
| hsa_circ_0136265_CBC1 | 0.020830815 | 2.1640063 |
| hsa_circ_0068026_CBC1 | 0.001852572 | 2.980713607 |
| hsa_circ_0103598_CBC1 | 0.000456262 | 2.396362073 |
| hsa_circ_0025039_CBC1 | 3.89471E-06 | 4.465238267 |
| hsa-circRNA15511-5_CBC1 | 0.001175886 | 3.465178801 |
| hsa_circ_0029130_CBC1 | 0.001930483 | 3.192437524 |
| hsa-circRNA1021-4_CBC1 | 0.009615399 | 4.114113771 |
| hsa_circ_0038390_CBC1 | 0.014403784 | 2.596037767 |
| hsa_circ_0059712_CBC1 | 2.13437E-05 | 7.889558277 |
| hsa_circ_0011711_CBC1 | 9.84331E-06 | 8.340129327 |
| hsa_circ_0138059_CBC1 | 0.000388585 | 2.430011846 |
| hsa_circ_0102230_CBC1 | 0.017699261 | 2.5084103 |
| hsa_circ_0102236_CBC1 | 0.002371011 | 2.122967447 |
| hsa-circRNA7836-74_CBC1 | 0.000468796 | 2.150952725 |
| hsa-circRNA11180-24_CBC1 | 0.000943137 | 3.666503872 |
| hsa_circ_0110332_CBC1 | 0.011199566 | 5.263188449 |
| hsa_circ_0063508_CBC1 | 0.000728672 | 2.367203482 |
| hsa_circ_0065755_CBC1 | 0.000256273 | 6.77245063 |
| hsa_circ_0076008_CBC1 | 0.039996068 | 2.336737722 |
| hsa_circ_0140160_CBC1 | 0.012095923 | 2.648165878 |
| hsa_circ_0072577_CBC1 | 0.001918547 | 3.831000341 |
| hsa_circ_0093450_CBC1 | 0.002610041 | 3.180477462 |
| hsa_circ_0070152_CBC1 | 0.008948436 | 2.820862647 |
| hsa_circ_0091250_CBC1 | 0.00052744 | 2.342097608 |
| hsa_circ_0097637_CBC1 | 0.000211867 | 2.409870579 |
| hsa_circ_0021705_CBC1 | 0.015107679 | 3.159090001 |
| hsa_circ_0055195_CBC1 | 0.000925407 | 2.683913783 |
| hsa_circ_0090948_CBC1 | 0.000632868 | 7.575788533 |
| hsa_circ_0133415_CBC1 | 0.001346872 | 2.606700794 |
| hsa_circ_0040485_CBC1 | 7.67282E-05 | 2.856390839 |
| hsa_circ_0037533_CBC1 | 1.65112E-05 | 11.15692429 |
| hsa_circ_0087442_CBC1 | 0.002534646 | 5.939055584 |
| hsa_circ_0076155_CBC1 | 0.012911982 | 2.018131116 |
| hsa_circ_0050697_CBC1 | 0.004288431 | 2.795300672 |
| hsa_circ_0013580_CBC1 | 0.019866378 | 2.284252568 |
| hsa-circRNA14781-33_CBC1 | 0.010140548 | 3.07052527 |
| hsa-circRNA8511-3_CBC1 | 0.006722358 | 4.675760528 |
| hsa-circRNA12269-31_CBC1 | 0.002701589 | 7.310229582 |
| hsa_circ_0000358_CBC1 | 0.003076497 | 2.267137371 |
| hsa-circRNA10312-9_CBC1 | 0.000270574 | 3.342398809 |
| hsa_circ_0036189_CBC1 | 0.001774807 | 2.024815334 |
| hsa_circ_0059397_CBC1 | 0.019342811 | 2.391907385 |
| hsa_circ_0136542_CBC1 | 0.027902745 | 2.185145564 |
| hsa_circ_0052019_CBC1 | 0.011638856 | 2.825466505 |
| hsa_circ_0035041_CBC1 | 0.009310602 | 2.408731289 |
| hsa_circ_0055669_CBC1 | 8.21306E-06 | 7.912462383 |
| hsa_circ_0008408_CBC1 | 0.000147996 | 2.181657438 |
| hsa_circ_0079035_CBC1 | 0.004787844 | 2.850829488 |
| hsa_circ_0014746_CBC1 | 0.000343613 | 7.6212091 |
| hsa_circ_0076746_CBC1 | 0.019303349 | 2.70890062 |
| hsa_circ_0067331_CBC1 | 0.00767929 | 2.464967497 |
| hsa_circ_0074845_CBC1 | 6.63314E-05 | 4.560238274 |
| hsa-circRNA2033-5_CBC1 | 0.001392743 | 2.546037488 |
| hsa_circ_0131458_CBC1 | 0.000834599 | 3.061529862 |
| hsa_circ_0068866_CBC1 | 0.000118741 | 3.559161949 |
| hsa_circ_0041010_CBC1 | 0.004499975 | 3.565612949 |
| hsa-circRNA813-10_CBC1 | 0.006768849 | 5.802246103 |
| hsa_circ_0132938_CBC1 | 0.002475579 | 2.786275219 |
| hsa_circ_0007522_CBC1 | 0.003448902 | 5.366435361 |
| hsa_circ_0044407_CBC1 | 0.007660815 | 2.839086133 |
| hsa_circ_0026924_CBC1 | 0.010119725 | 2.104456989 |
| hsa-circRNA8791-20_CBC1 | 8.44966E-05 | 5.041768213 |
| hsa_circ_0028445_CBC1 | 0.003482554 | 3.858440125 |
| hsa_circ_0140589_CBC1 | 0.038412745 | 2.282846069 |
| hsa-circRNA4965-37_CBC1 | 1.23046E-05 | 7.882309919 |
| hsa-circRNA7445-20_CBC1 | 0.011864658 | 2.921955663 |
| hsa-circRNA5806-19_CBC1 | 0.005743029 | 2.855627983 |
| hsa_circ_0117680_CBC1 | 0.000986363 | 2.043317744 |
| hsa_circ_0072768_CBC1 | 0.003993421 | 2.305030339 |
| hsa-circRNA5535-10_CBC1 | 0.003713624 | 4.26668798 |
| hsa_circ_0014757_CBC1 | 0.000492231 | 3.612052282 |
| hsa_circ_0059401_CBC1 | 0.018532286 | 2.085032844 |
| hsa_circ_0120408_CBC1 | 0.005805104 | 2.109802384 |
| hsa-circRNA7836-80_CBC1 | 0.000141926 | 3.131633126 |
| hsa_circ_0072761_CBC1 | 2.34019E-05 | 8.002599746 |
| hsa_circ_0042374_CBC1 | 0.001776088 | 3.368130266 |
| hsa_circ_0135665_CBC1 | 0.002068159 | 5.179820549 |
| hsa_circ_0001729_CBC1 | 0.000161878 | 3.152244712 |
| hsa_circ_0062595_CBC1 | 0.045966349 | 2.160994292 |
| hsa_circ_0007532_CBC1 | 3.33784E-06 | 6.755641856 |
| hsa_circ_0076631_CBC1 | 0.00118823 | 3.210677893 |
| hsa_circ_0025675_CBC1 | 0.020046534 | 2.74493191 |
| hsa_circ_0010977_CBC1 | 0.000571327 | 2.001431117 |
| hsa_circ_0011969_CBC1 | 0.004190841 | 2.625361049 |
| hsa-circRNA11137-12_CBC1 | 3.39867E-05 | 2.134544397 |
| hsa-circRNA2313-3_CBC1 | 0.014135545 | 2.084391926 |
| hsa_circ_0009824_CBC1 | 0.015413228 | 2.528310192 |
| hsa_circ_0004266_CBC1 | 0.000260578 | 2.573762606 |
| hsa_circ_0100252_CBC1 | 0.00608668 | 8.36999334 |
| hsa_circ_0048676_CBC1 | 0.005534118 | 3.81432863 |
| hsa_circ_0063604_CBC1 | 0.002340664 | 2.135558022 |
| hsa-circRNA2527-21_CBC1 | 0.000772633 | 4.196682902 |
| hsa_circ_0006912_CBC1 | 0.001831926 | 3.788568211 |
| hsa-circRNA1179-2_CBC1 | 0.001240822 | 2.2057801 |
| hsa_circ_0076952_CBC1 | 0.017050023 | 2.110107031 |
| hsa_circ_0026186_CBC1 | 0.002030449 | 2.16908116 |
| hsa_circ_0091718_CBC1 | 0.010476294 | 5.997516735 |
| hsa_circ_0109105_CBC1 | 0.006524118 | 3.482801752 |
| hsa_circ_0032021_CBC1 | 0.012716928 | 2.224756002 |
| hsa-circRNA6411-6_CBC1 | 0.015362346 | 2.073207352 |
| hsa_circ_0069014_CBC1 | 0.004121919 | 2.059722945 |
| hsa_circ_0003573_CBC1 | 0.007132911 | 2.117006241 |
| hsa_circ_0036051_CBC1 | 0.002868635 | 2.265657501 |
| hsa_circ_0098774_CBC1 | 0.014182103 | 2.658458904 |
| hsa_circ_0127800_CBC1 | 0.018816248 | 5.465563653 |
| hsa-circRNA1257-28_CBC1 | 0.010023847 | 3.255436609 |
| hsa_circ_0060454_CBC1 | 0.000371302 | 5.675264473 |
| hsa-circRNA13698-41_CBC1 | 0.03744043 | 3.519983719 |
| hsa-circRNA10312-7_CBC1 | 0.002571201 | 3.004629901 |
| hsa_circ_0036915_CBC1 | 0.000307654 | 2.928467045 |
| hsa-circRNA10466-2_CBC1 | 2.86742E-05 | 2.539319462 |
| hsa_circ_0025367_CBC1 | 0.041771861 | 2.188357811 |
| hsa-circRNA10149-15_CBC1 | 0.000376685 | 6.087240924 |
| hsa_circ_0097653_CBC1 | 0.012338533 | 2.631505305 |
| hsa_circ_0050698_CBC1 | 0.010475484 | 2.790675315 |
| hsa-circRNA6201-2_CBC1 | 4.03992E-05 | 8.754176169 |
| hsa_circ_0082539_CBC1 | 0.00341403 | 2.232193634 |
| hsa_circ_0006440_CBC1 | 0.016259451 | 2.757115848 |
| hsa_circ_0043153_CBC1 | 0.024384307 | 2.416038378 |
| hsa_circ_0068331_CBC1 | 0.003825127 | 2.090908219 |
| hsa_circ_0059850_CBC1 | 0.007957025 | 2.297625441 |
| hsa-circRNA14089-5_CBC1 | 0.001839686 | 9.585965843 |
| hsa_circ_0051818_CBC1 | 0.000125202 | 2.121094554 |
| hsa-circRNA1257-44_CBC1 | 0.001911763 | 3.069586004 |
| hsa_circ_0070916_CBC1 | 0.001998448 | 4.351291259 |
| hsa-circRNA8881-20_CBC1 | 0.00081236 | 7.466222903 |
| hsa_circ_0139863_CBC1 | 0.000701361 | 2.931386842 |
| hsa_circ_0081555_CBC1 | 0.000129656 | 2.19158222 |
| hsa_circ_0045872_CBC1 | 0.001895943 | 4.252780462 |
| hsa-circRNA7601-19_CBC1 | 1.2308E-05 | 2.402113368 |
| hsa_circ_0026360_CBC1 | 0.000150106 | 2.026772602 |
| hsa_circ_0015761_CBC1 | 0.006224525 | 4.033988721 |
| hsa_circ_0034618_CBC1 | 0.000379091 | 5.891267691 |
| hsa_circ_0063028_CBC1 | 0.001335562 | 2.681593074 |
| hsa_circ_0104335_CBC1 | 0.002759492 | 2.526088287 |
| hsa_circ_0029274_CBC1 | 0.004390105 | 2.162769608 |
| hsa-circRNA8500-4_CBC1 | 0.000650046 | 3.792386362 |
| hsa-circRNA11458-29_CBC1 | 0.001144853 | 4.526651846 |
| hsa_circ_0073749_CBC1 | 0.000152347 | 8.16381111 |
| hsa-circRNA10602-9_CBC1 | 0.009873966 | 2.051997834 |
| hsa_circ_0115109_CBC1 | 0.001859623 | 2.528901492 |
| hsa_circ_0007215_CBC1 | 0.004329712 | 2.01400924 |
| hsa_circ_0011164_CBC1 | 0.037190555 | 2.636138968 |
| hsa-circRNA12047-2_CBC1 | 6.70103E-05 | 8.224704758 |
| hsa_circ_0059717_CBC1 | 2.2959E-06 | 7.796371275 |
| hsa_circ_0094260_CBC1 | 0.000727888 | 2.794181689 |
| hsa_circ_0110375_CBC1 | 0.021433736 | 3.598260681 |
| hsa-circRNA2527-10_CBC1 | 0.000912665 | 8.366405113 |
| hsa_circ_0029116_CBC1 | 0.014705251 | 2.336861383 |
| hsa_circ_0098215_CBC1 | 0.004622788 | 2.185824547 |
| hsa_circ_0059731_CBC1 | 1.97099E-05 | 7.656121254 |
| hsa_circ_0041859_CBC1 | 0.000387172 | 2.071094631 |
| hsa-circRNA1940-5_CBC1 | 0.006232304 | 4.288712348 |
| hsa_circ_0054619_CBC1 | 0.035810941 | 2.441754747 |
| hsa_circ_0085993_CBC1 | 0.007334822 | 2.254758868 |
| hsa_circ_0088942_CBC1 | 1.35406E-05 | 2.732370907 |
| hsa_circ_0121668_CBC1 | 0.017153799 | 2.6877104 |
| hsa_circ_0044388_CBC1 | 0.038434586 | 2.123572107 |
| hsa_circ_0096712_CBC1 | 0.022249148 | 2.167401645 |
| hsa-circRNA5149-16_CBC1 | 0.005532651 | 2.066538511 |
| hsa-circRNA5806-3_CBC1 | 0.007304431 | 3.180152682 |
| hsa_circ_0050699_CBC1 | 4.49511E-05 | 4.946085346 |
| hsa-circRNA10593-1_CBC1 | 0.00209466 | 4.73525744 |
| hsa_circ_0002297_CBC1 | 0.000396743 | 3.045208396 |
| hsa-circRNA11084-7_CBC1 | 0.000523675 | 9.128081243 |
| hsa_circ_0078794_CBC1 | 0.015275812 | 2.071372428 |
| hsa-circRNA4065-24_CBC1 | 0.00012286 | 2.23913814 |
| hsa_circ_0073764_CBC1 | 0.012349291 | 2.450747222 |
| hsa_circ_0052972_CBC1 | 0.010527088 | 2.047072348 |
| hsa_circ_0136404_CBC1 | 0.002657634 | 2.690450374 |
| hsa_circ_0082502_CBC1 | 0.003850628 | 2.093695048 |
| hsa-circRNA2104-7_CBC1 | 0.000648301 | 2.05792754 |
| hsa-circRNA11981-18_CBC1 | 0.000106285 | 3.748252037 |
| hsa_circ_0113481_CBC1 | 0.001693405 | 3.313325762 |
| hsa_circ_0015767_CBC1 | 0.002697608 | 5.471832893 |
| hsa_circ_0022128_CBC1 | 0.00105331 | 2.290162059 |
| hsa_circ_0129250_CBC1 | 0.002489811 | 2.93385253 |
| hsa-circRNA12510-20_CBC1 | 0.007155848 | 2.577490647 |
| hsa_circ_0088969_CBC1 | 0.018154696 | 2.824421458 |
| hsa_circ_0108997_CBC1 | 0.001025649 | 2.745452676 |
| hsa_circ_0016323_CBC1 | 0.041180807 | 2.356166601 |
| hsa_circ_0018040_CBC1 | 0.000394434 | 2.921468585 |
| hsa_circ_0088328_CBC1 | 0.001391505 | 2.579663474 |
| hsa_circ_0042563_CBC1 | 0.001448233 | 9.658113141 |
| hsa_circ_0091421_CBC1 | 0.004191623 | 2.726100457 |
| hsa_circ_0013393_CBC1 | 0.003892487 | 2.273437076 |
| hsa_circ_0029136_CBC1 | 0.008849268 | 4.080433953 |
| hsa_circ_0124914_CBC1 | 0.00046249 | 3.617981944 |
| hsa_circ_0078745_CBC1 | 0.000440027 | 2.760990515 |
| hsa_circ_0005774_CBC1 | 0.001020009 | 4.20322958 |
| hsa_circ_0135172_CBC1 | 0.018257397 | 2.225290046 |
| hsa_circ_0090957_CBC1 | 0.000153969 | 3.299817208 |
| hsa-circRNA12269-19_CBC1 | 0.000612555 | 8.35367707 |
| hsa_circ_0029510_CBC1 | 1.33558E-05 | 3.493334387 |
| hsa_circ_0053000_CBC1 | 0.00076837 | 3.606577794 |
| hsa-circRNA5427-74_CBC1 | 9.1113E-05 | 9.843432945 |
| hsa-circRNA2407-21_CBC1 | 0.000237729 | 3.220619832 |
| hsa_circ_0045480_CBC1 | 0.001191621 | 2.143670849 |
| hsa_circ_0016239_CBC1 | 0.003258084 | 2.429205757 |
| hsa_circ_0041018_CBC1 | 0.000640539 | 8.839637983 |
| hsa-circRNA1257-24_CBC1 | 3.07061E-06 | 2.334879786 |
| hsa-circRNA11084-11_CBC1 | 0.001079375 | 13.53568575 |
| hsa-circRNA858-5_CBC1 | 0.002891025 | 5.733404048 |
| hsa_circ_0000899_CBC1 | 0.00405214 | 2.551551624 |
| hsa_circ_0050700_CBC1 | 0.000114989 | 6.496981108 |
| hsa_circ_0040965_CBC1 | 0.004147951 | 2.115662578 |
| hsa_circ_0059326_CBC1 | 7.06694E-05 | 4.639917904 |
| hsa_circ_0088803_CBC1 | 0.000291709 | 2.280616037 |
| hsa_circ_0116800_CBC1 | 0.034970069 | 3.255934443 |
| hsa-circRNA8881-19_CBC1 | 0.000386481 | 9.580284895 |
| hsa_circ_0018418_CBC1 | 0.001064771 | 3.31843661 |
| hsa_circ_0085472_CBC1 | 0.00125495 | 3.700424908 |
| hsa_circ_0018491_CBC1 | 0.001468966 | 3.757185389 |
| hsa_circ_0067843_CBC1 | 0.003737746 | 4.287317743 |
| hsa_circ_0001332_CBC1 | 0.017540065 | 4.767413716 |
| hsa_circ_0003507_CBC1 | 0.018778048 | 2.233775574 |
| hsa_circ_0095732_CBC1 | 0.016524407 | 2.453205313 |
| hsa_circ_0039475_CBC1 | 0.001613359 | 2.351794608 |
| hsa_circ_0019243_CBC1 | 0.014082535 | 3.654210484 |
| hsa_circ_0000179_CBC1 | 0.005897037 | 5.285434623 |
| hsa-circRNA11175-5_CBC1 | 0.009843368 | 2.747162766 |
| hsa-circRNA8621-9_CBC1 | 0.003675025 | 4.038173712 |
| hsa_circ_0041874_CBC1 | 0.000588712 | 2.155120378 |
| hsa_circ_0043589_CBC1 | 0.030428953 | 2.081131239 |
| hsa-circRNA13753-24_CBC1 | 0.001281381 | 2.160404861 |
| hsa_circ_0038767_CBC1 | 0.028714431 | 2.07116669 |
| hsa_circ_0102405_CBC1 | 0.005747798 | 4.144426113 |
| hsa_circ_0076739_CBC1 | 0.020811045 | 2.690389447 |
| hsa-circRNA15373-4_CBC1 | 0.038445053 | 2.085904491 |
| hsa_circ_0059877_CBC1 | 0.003626033 | 4.816013649 |
| hsa_circ_0063526_CBC1 | 0.002623283 | 2.019943308 |
| hsa_circ_0079872_CBC1 | 0.00023739 | 5.972715593 |
| hsa_circ_0044405_CBC1 | 0.007533221 | 2.081411956 |
| hsa-circRNA13698-31_CBC1 | 0.005952069 | 4.988934378 |
| hsa_circ_0102404_CBC1 | 0.001254929 | 6.159458878 |
| hsa_circ_0029094_CBC1 | 0.003518677 | 3.264025877 |
| hsa-circRNA13754-2_CBC1 | 0.011976441 | 2.346336115 |
| hsa_circ_0014760_CBC1 | 0.000159622 | 5.886330453 |
| hsa_circ_0068332_CBC1 | 0.000981834 | 2.387352635 |
| hsa-circRNA11180-26_CBC1 | 0.00180334 | 2.425633804 |
| hsa_circ_0102407_CBC1 | 0.000757736 | 2.86778966 |
| hsa_circ_0041007_CBC1 | 0.013440673 | 2.731272047 |
| hsa-circRNA11493-35_CBC1 | 0.009315113 | 3.637402391 |
| hsa_circ_0028599_CBC1 | 0.003879344 | 3.553067153 |
| hsa_circ_0053159_CBC1 | 0.000571717 | 2.128726648 |
| hsa-circRNA15598-1_CBC1 | 0.0053928 | 5.855232451 |
| hsa_circ_0040487_CBC1 | 0.000776727 | 2.501527539 |
| hsa_circ_0110335_CBC1 | 0.00028315 | 3.270886933 |
| hsa-circRNA1599-4_CBC1 | 0.036753193 | 3.389187593 |
| hsa-circRNA946-11_CBC1 | 0.000133318 | 4.191108723 |
| hsa-circRNA7445-26_CBC1 | 0.010380338 | 2.999966784 |
| hsa-circRNA8885-6_CBC1 | 4.5165E-05 | 16.52347746 |
| hsa_circ_0036731_CBC1 | 0.000969664 | 5.159088152 |
| hsa_circ_0039878_CBC1 | 0.005853836 | 2.01817016 |
| hsa-circRNA8621-8_CBC1 | 0.000951982 | 3.273567617 |
| hsa-circRNA15355-9_CBC1 | 0.01213283 | 3.06838989 |
| hsa_circ_0030813_CBC1 | 0.028656654 | 2.393907584 |
| hsa_circ_0038924_CBC1 | 0.006205326 | 2.005391188 |
| hsa_circ_0029145_CBC1 | 0.001116786 | 3.100277276 |
| hsa_circ_0041019_CBC1 | 0.000164758 | 5.625286457 |
| hsa_circ_0081407_CBC1 | 1.44202E-06 | 3.441008027 |
| hsa_circ_0060549_CBC1 | 2.52646E-05 | 7.680396013 |
| hsa-circRNA1649-30_CBC1 | 0.000778491 | 4.353592478 |
| hsa_circ_0012370_CBC1 | 0.008711344 | 3.133691698 |
| hsa_circ_0100755_CBC1 | 0.001210269 | 3.864961527 |
| hsa-circRNA9240-5_CBC1 | 0.045482196 | 2.18748144 |
| hsa_circ_0103038_CBC1 | 0.002837777 | 2.805977993 |
| hsa_circ_0025157_CBC1 | 0.000154538 | 3.975060387 |
| hsa_circ_0093963_CBC1 | 0.013794375 | 4.009006196 |
| hsa_circ_0101734_CBC1 | 0.018084871 | 2.15850862 |
| hsa-circRNA8500-20_CBC1 | 0.002873021 | 3.902606901 |
| hsa_circ_0043860_CBC1 | 0.001598298 | 2.282534069 |
| hsa-circRNA10149-36_CBC1 | 2.90057E-05 | 7.435920917 |
| hsa-circRNA8297-104_CBC1 | 0.000566133 | 2.168264852 |
| hsa_circ_0006964_CBC1 | 0.00131062 | 4.83221832 |
| hsa-circRNA2502-3_CBC1 | 0.000256073 | 6.494791013 |
| hsa_circ_0097651_CBC1 | 0.001680387 | 2.130917407 |
| hsa_circ_0104842_CBC1 | 0.008686418 | 3.338384126 |
| hsa_circ_0014766_CBC1 | 0.001781002 | 11.91983202 |
| hsa_circ_0049679_CBC1 | 0.008063496 | 2.414090636 |
| hsa_circ_0040773_CBC1 | 0.01482253 | 2.699987004 |
| hsa_circ_0110573_CBC1 | 0.00527411 | 2.027240941 |
| hsa_circ_0090944_CBC1 | 0.008878781 | 2.194892315 |
| hsa-circRNA12269-28_CBC1 | 0.000176179 | 6.576693473 |
| hsa_circ_0021608_CBC1 | 0.039203824 | 2.071707488 |
| hsa_circ_0038054_CBC1 | 0.005613516 | 2.250986687 |
| hsa_circ_0012154_CBC1 | 0.005206717 | 2.768259265 |
| hsa_circ_0025155_CBC1 | 2.26007E-06 | 3.566347322 |
| hsa-circRNA8885-37_CBC1 | 0.002306117 | 8.989655763 |
| hsa_circ_0096195_CBC1 | 0.005779648 | 3.758058375 |
| hsa_circ_0063608_CBC1 | 0.001903382 | 2.20725342 |
| hsa-circRNA15355-3_CBC1 | 0.002302206 | 3.257654806 |
| hsa-circRNA15229-31_CBC1 | 5.01247E-06 | 3.21230826 |
| hsa-circRNA4064-4_CBC1 | 0.003086534 | 2.119961726 |
| hsa_circ_0034578_CBC1 | 0.000664678 | 2.147260843 |
| hsa-circRNA6713-2_CBC1 | 0.03430169 | 2.650585799 |
| hsa_circ_0067170_CBC1 | 0.002243548 | 2.325720463 |
| hsa_circ_0086137_CBC1 | 1.85609E-05 | 2.445386795 |
| hsa_circ_0034601_CBC1 | 0.004527967 | 9.444818129 |
| hsa_circ_0051733_CBC1 | 0.000136046 | 4.772066167 |
| hsa_circ_0087035_CBC1 | 0.018520865 | 3.04401418 |
| hsa_circ_0015176_CBC1 | 0.014337372 | 3.83666226 |
| hsa_circ_0050678_CBC1 | 0.001330144 | 2.912971235 |
| hsa_circ_0029111_CBC1 | 0.025035409 | 2.012198992 |
| hsa_circ_0067172_CBC1 | 0.001510821 | 3.968838281 |
| hsa_circ_0019486_CBC1 | 0.011507376 | 2.220170136 |
| hsa_circ_0049083_CBC1 | 0.001852539 | 2.136179883 |
| hsa-circRNA10312-10_CBC1 | 0.008466476 | 2.340835018 |
| hsa_circ_0117736_CBC1 | 0.0007903 | 4.651105044 |
| hsa_circ_0045599_CBC1 | 0.010181409 | 2.163333331 |
| hsa_circ_0134447_CBC1 | 0.034884374 | 2.409906894 |
| hsa_circ_0045732_CBC1 | 0.026747105 | 2.015934868 |
| hsa-circRNA5796-6_CBC1 | 0.00077043 | 3.700552444 |
| hsa-circRNA2148_CBC1 | 0.000273335 | 5.386274015 |
| hsa-circRNA14838-64_CBC1 | 0.000230044 | 2.70723749 |
| hsa_circ_0028594_CBC1 | 0.00094436 | 3.251446541 |
| hsa-circRNA8486-3_CBC1 | 0.007092068 | 2.074430996 |
| hsa_circ_0077403_CBC1 | 0.019241259 | 3.280536438 |
| hsa_circ_0029697_CBC1 | 0.000966005 | 3.052377728 |
| hsa_circ_0088926_CBC1 | 0.00035582 | 2.255220516 |
| hsa_circ_0025677_CBC1 | 0.000472037 | 2.035381289 |
| hsa-circRNA6817-1_CBC1 | 0.000572402 | 3.744054356 |
| hsa_circ_0036708_CBC1 | 0.001537424 | 4.327757234 |
| hsa_circ_0084349_CBC1 | 0.013384964 | 2.233705274 |
| hsa_circ_0113189_CBC1 | 0.011679876 | 5.428319625 |
| hsa_circ_0049796_CBC1 | 0.001107255 | 2.135522964 |
| hsa-circRNA13365-2_CBC1 | 0.00032746 | 3.931121014 |
| hsa_circ_0046129_CBC1 | 0.001459719 | 2.595159266 |
| hsa-circRNA9267-5_CBC1 | 0.015863894 | 2.63100601 |
| hsa_circ_0132352_CBC1 | 0.012704286 | 2.199841459 |
| hsa-circRNA4065-22_CBC1 | 0.012911657 | 2.031488695 |
| hsa-circRNA13465-6_CBC1 | 0.000121534 | 6.197105231 |
| hsa_circ_0102161_CBC1 | 0.026871108 | 2.104371924 |
| hsa_circ_0060450_CBC1 | 0.000105446 | 3.886296603 |
| hsa_circ_0085475_CBC1 | 0.002625818 | 2.998730328 |
| hsa_circ_0128868_CBC1 | 0.004086972 | 3.022150197 |
| hsa-circRNA13365-4_CBC1 | 0.001633465 | 2.389777613 |
| hsa-circRNA9195-40_CBC1 | 0.023917474 | 2.620884965 |
| hsa_circ_0098616_CBC1 | 0.012452269 | 2.497669269 |
| hsa-circRNA14844-16_CBC1 | 0.002462694 | 3.140287217 |
| hsa_circ_0088941_CBC1 | 0.008556944 | 4.538016113 |
| hsa-circRNA12919_CBC1 | 0.002873323 | 2.398477078 |
| hsa_circ_0013195_CBC1 | 0.015080096 | 3.941086485 |
| hsa_circ_0060550_CBC1 | 1.99388E-05 | 5.565218095 |
| hsa_circ_0034606_CBC1 | 0.025773229 | 4.128277428 |
| hsa_circ_0084484_CBC1 | 0.015366156 | 2.5204034 |
| hsa_circ_0032090_CBC1 | 0.003438191 | 2.982230471 |
| hsa_circ_0040582_CBC1 | 0.000167136 | 3.377250821 |
| hsa_circ_0081394_CBC1 | 0.024217199 | 3.0161976 |
| hsa_circ_0060458_CBC1 | 2.27496E-07 | 12.59935878 |
| hsa_circ_0017788_CBC1 | 0.003291836 | 11.45475926 |
| hsa_circ_0069008_CBC1 | 0.00689576 | 2.026874681 |
| hsa_circ_0086745_CBC1 | 0.000832322 | 11.02533217 |
| hsa_circ_0068328_CBC1 | 0.02633742 | 2.143065595 |
| hsa-circRNA2283-3_CBC1 | 0.010528754 | 3.245117248 |
| hsa_circ_0041266_CBC1 | 0.016375921 | 2.208780901 |
| hsa_circ_0041003_CBC1 | 0.002955877 | 5.014489557 |
| hsa-circRNA5427-58_CBC1 | 0.000447269 | 6.061599634 |
| hsa_circ_0029696_CBC1 | 0.002517393 | 3.456139664 |
| hsa_circ_0036713_CBC1 | 0.000694447 | 3.403999766 |
| hsa_circ_0041988_CBC1 | 0.000422386 | 2.048252643 |
| hsa_circ_0076738_CBC1 | 0.010302546 | 2.651021789 |
| hsa_circ_0084230_CBC1 | 0.031261635 | 2.66099727 |
| hsa_circ_0025270_CBC1 | 0.002848365 | 2.216100704 |
| hsa-circRNA1291-51_CBC1 | 0.000913793 | 4.071671884 |
| hsa_circ_0107533_CBC1 | 0.004488061 | 2.735235828 |
| hsa_circ_0108832_CBC1 | 0.009956381 | 2.874842831 |
| hsa-circRNA13520-18_CBC1 | 0.002523854 | 2.628461959 |
| hsa_circ_0012363_CBC1 | 0.006111276 | 4.902280127 |
| hsa-circRNA15902-42_CBC1 | 0.008236359 | 2.244065016 |
| hsa_circ_0088312_CBC1 | 0.039510962 | 2.045664832 |
| hsa-circRNA1594-5_CBC1 | 0.04671519 | 2.261272486 |
| hsa_circ_0056041_CBC1 | 0.00048091 | 6.572744239 |
| hsa_circ_0021714_CBC1 | 0.006554542 | 2.07919691 |
| hsa_circ_0043539_CBC1 | 0.005162232 | 6.736327065 |
| hsa_circ_0076755_CBC1 | 0.009383706 | 2.657458855 |
| hsa-circRNA13465-4_CBC1 | 0.000245615 | 3.354674769 |
| hsa_circ_0001586_CBC1 | 0.029578461 | 2.040541107 |
| hsa-circRNA15389-17_CBC1 | 3.94095E-05 | 4.730592933 |
| hsa_circ_0088996_CBC1 | 0.000777859 | 2.205192971 |
| hsa_circ_0085991_CBC1 | 0.010949333 | 2.653010428 |
| hsa_circ_0064241_CBC1 | 0.000506235 | 4.235030966 |
| hsa_circ_0045873_CBC1 | 0.003198312 | 3.51214498 |
| hsa_circ_0004806_CBC1 | 0.000240825 | 2.407502497 |
| hsa_circ_0096045_CBC1 | 0.000228684 | 3.417640908 |
| hsa_circ_0108752_CBC1 | 0.012448768 | 2.059157302 |
| hsa_circ_0032004_CBC1 | 0.00569326 | 2.390764702 |
| hsa-circRNA11180-18_CBC1 | 0.001624755 | 3.076855623 |
| hsa_circ_0005971_CBC1 | 0.001947838 | 2.710717582 |
| hsa_circ_0034641_CBC1 | 0.005939587 | 2.227979438 |
| hsa_circ_0028884_CBC1 | 0.005697525 | 2.009241371 |
| hsa_circ_0019543_CBC1 | 0.000909395 | 2.250439434 |
| hsa_circ_0052586_CBC1 | 6.04305E-05 | 6.142396449 |
| hsa_circ_0049221_CBC1 | 0.001296585 | 2.417534916 |
| hsa_circ_0003153_CBC1 | 7.05297E-05 | 5.656990194 |
| hsa_circ_0006351_CBC1 | 0.001010628 | 3.658197988 |
| hsa_circ_0110329_CBC1 | 0.001817698 | 3.032646848 |
| hsa-circRNA11981-52_CBC1 | 0.000161186 | 2.862006955 |
| hsa_circ_0066940_CBC1 | 0.003791257 | 5.053540547 |
| hsa-circRNA9158-26_CBC1 | 0.010201203 | 5.342673486 |
| hsa_circ_0040999_CBC1 | 0.002508457 | 4.455795817 |
| hsa_circ_0003112_CBC1 | 0.001172479 | 2.101194801 |
| hsa-circRNA8621-5_CBC1 | 0.001536285 | 2.523141318 |
| hsa_circ_0060457_CBC1 | 7.75613E-05 | 3.477963598 |
| hsa_circ_0029539_CBC1 | 0.006528405 | 2.786037685 |
| hsa_circ_0059732_CBC1 | 3.65471E-05 | 6.237382494 |
| hsa-circRNA15162-124_CBC1 | 0.00162979 | 2.683729136 |
| hsa-circRNA2623-14_CBC1 | 0.000636248 | 2.263577458 |
| hsa_circ_0051810_CBC1 | 0.000392752 | 2.043639463 |
| hsa_circ_0120335_CBC1 | 0.015459377 | 2.418874773 |
| hsa_circ_0021609_CBC1 | 0.011810804 | 2.27049281 |
| hsa_circ_0127817_CBC1 | 0.002690266 | 2.481090546 |
| hsa-circRNA7777-4_CBC1 | 0.006848929 | 2.354664825 |
| hsa_circ_0025051_CBC1 | 0.004758241 | 2.768432963 |
| hsa_circ_0113771_CBC1 | 0.001632543 | 2.41127197 |
| hsa_circ_0003009_CBC1 | 0.003742144 | 2.261096229 |
| hsa_circ_0120414_CBC1 | 0.043507278 | 3.744934404 |
| hsa_circ_0053386_CBC1 | 0.021612978 | 2.383245824 |
| hsa_circ_0045477_CBC1 | 4.77305E-05 | 2.541352804 |
| hsa-circRNA3385-10_CBC1 | 0.011916339 | 2.539632646 |
| hsa_circ_0088925_CBC1 | 0.002204982 | 2.133752003 |
| hsa_circ_0049405_CBC1 | 1.64101E-06 | 7.846893935 |
| hsa_circ_0014755_CBC1 | 0.002525036 | 4.897629258 |
| hsa_circ_0013724_CBC1 | 0.000368114 | 2.871375879 |
| hsa-circRNA8518-29_CBC1 | 0.008603751 | 2.340574973 |
| hsa_circ_0064859_CBC1 | 0.004551916 | 2.468075274 |
| hsa_circ_0032821_CBC1 | 0.005332402 | 2.907747033 |
| hsa_circ_0088805_CBC1 | 0.002600002 | 2.361443558 |
| hsa-circRNA14229-7_CBC1 | 0.002923575 | 2.136193865 |
| hsa-circRNA15389-15_CBC1 | 0.000412649 | 3.929822309 |
| hsa-circRNA8433-14_CBC1 | 0.016978007 | 3.292466751 |
| hsa-circRNA8326-3_CBC1 | 0.020683971 | 2.140213016 |
| hsa-circRNA8518-11_CBC1 | 0.008028475 | 2.421544728 |
| hsa-circRNA11873-4_CBC1 | 0.032788862 | 2.040916455 |
| hsa_circ_0067390_CBC1 | 0.004299931 | 2.668941922 |
| hsa_circ_0091244_CBC1 | 0.002379965 | 4.518082308 |
| hsa_circ_0043862_CBC1 | 0.020059679 | 2.149762528 |
| hsa_circ_0036916_CBC1 | 0.000104796 | 5.886172812 |
| hsa-circRNA6265-12_CBC1 | 0.030126361 | 2.765986312 |
| hsa_circ_0005659_CBC1 | 0.000972136 | 7.692480184 |
| hsa-circRNA9929-9_CBC1 | 0.00023865 | 5.463860249 |
| hsa_circ_0000380_CBC1 | 0.002055384 | 2.05211176 |
| hsa_circ_0013385_CBC1 | 0.003511383 | 2.368136935 |
| hsa_circ_0021910_CBC1 | 0.000194869 | 2.499374262 |
| hsa_circ_0052582_CBC1 | 0.000235004 | 5.36455489 |
| hsa_circ_0012090_CBC1 | 0.006051642 | 2.071710456 |
| hsa_circ_0021493_CBC1 | 1.228E-05 | 2.543431572 |
| hsa_circ_0028701_CBC1 | 0.003970957 | 10.80868738 |
| hsa-circRNA8935-1_CBC1 | 0.000726207 | 10.27959151 |
| hsa_circ_0069015_CBC1 | 0.001146 | 2.004569426 |
| hsa_circ_0097652_CBC1 | 0.002502518 | 2.508416395 |
| hsa-circRNA14438-2_CBC1 | 0.007121796 | 7.813746244 |
| hsa_circ_0065833_CBC1 | 0.007592691 | 2.78692951 |
| hsa_circ_0072757_CBC1 | 4.40347E-05 | 6.92494223 |
| hsa_circ_0098849_CBC1 | 0.028802208 | 2.319078712 |
| hsa_circ_0128496_CBC1 | 0.000431866 | 7.554897241 |
| hsa_circ_0106036_CBC1 | 0.010301231 | 3.146343983 |
| hsa_circ_0105354_CBC1 | 0.00030946 | 2.049298622 |
| hsa_circ_0084351_CBC1 | 0.002352279 | 2.055772653 |
| hsa-circRNA1729-1_CBC1 | 0.000961756 | 3.068836637 |
| hsa_circ_0138061_CBC1 | 0.002568716 | 2.403261915 |
| hsa-circRNA14844-4_CBC1 | 0.040883418 | 2.740741028 |
| hsa_circ_0019215_CBC1 | 0.000223896 | 12.46620776 |
| hsa-circRNA858-1_CBC1 | 0.001738223 | 9.586909253 |
| hsa_circ_0047085_CBC1 | 0.048867106 | 2.252787882 |
| hsa_circ_0045026_CBC1 | 0.013457428 | 2.329157542 |
| hsa_circ_0059735_CBC1 | 0.00023638 | 12.72211271 |
| hsa_circ_0106818_CBC1 | 2.69111E-05 | 6.776920457 |
| hsa_circ_0037536_CBC1 | 0.000317584 | 4.055536495 |
| hsa_circ_0050721_CBC1 | 5.47583E-05 | 3.78522865 |
| hsa_circ_0004480_CBC1 | 0.000415424 | 3.839130786 |
| hsa-circRNA11981-14_CBC1 | 6.60399E-05 | 4.050441372 |
| hsa_circ_0044131_CBC1 | 0.000101475 | 9.020131865 |
| hsa_circ_0065416_CBC1 | 0.017669334 | 2.095777406 |
| hsa_circ_0012165_CBC1 | 1.87438E-06 | 7.515327662 |
| hsa-circRNA2033-23_CBC1 | 0.005899137 | 2.267383589 |
| hsa_circ_0097702_CBC1 | 0.024029086 | 3.079797586 |
| hsa_circ_0097491_CBC1 | 0.003463818 | 2.052203941 |
| hsa-circRNA11400-1_CBC1 | 0.025828007 | 2.150655302 |
| hsa_circ_0012164_CBC1 | 1.79745E-05 | 6.955136798 |
| hsa-circRNA13048-8_CBC1 | 0.019498564 | 2.186430704 |
| hsa_circ_0102397_CBC1 | 0.000300481 | 3.643412158 |
| hsa_circ_0139351_CBC1 | 0.000276291 | 4.155949775 |
| hsa_circ_0103439_CBC1 | 0.002523933 | 10.62335954 |
| hsa_circ_0060453_CBC1 | 0.001394966 | 12.14761603 |
| hsa_circ_0128570_CBC1 | 0.029202558 | 2.67135708 |
| hsa_circ_0035997_CBC1 | 0.001874606 | 3.084013161 |
| hsa_circ_0115125_CBC1 | 0.044744673 | 3.206464824 |
| hsa_circ_0119179_CBC1 | 0.001807567 | 2.346779393 |
| hsa_circ_0015976_CBC1 | 0.000354391 | 4.791704045 |
| hsa_circ_0049710_CBC1 | 0.046299281 | 2.496750401 |
| hsa_circ_0042380_CBC1 | 0.003564884 | 2.71851098 |
| hsa-circRNA2721-2_CBC1 | 0.000240307 | 6.590032557 |
| hsa_circ_0052391_CBC1 | 0.004840893 | 2.416686066 |
| hsa_circ_0064252_CBC1 | 0.000531462 | 8.694678476 |
| hsa_circ_0031784_CBC1 | 0.001426709 | 4.702899759 |
| hsa_circ_0042566_CBC1 | 0.000631854 | 5.395814071 |
| hsa_circ_0038056_CBC1 | 0.043082113 | 2.169532685 |
| hsa_circ_0032710_CBC1 | 0.04091264 | 2.113799218 |
| hsa_circ_0065832_CBC1 | 0.025965999 | 2.672523821 |
| hsa-circRNA9989-10_CBC1 | 0.000763818 | 6.680171664 |
| hsa_circ_0060468_CBC1 | 0.001891408 | 7.435800347 |
| hsa_circ_0137804_CBC1 | 0.004089144 | 2.327755215 |
| hsa_circ_0007922_CBC1 | 0.01281443 | 3.844097949 |
| hsa_circ_0065352_CBC1 | 0.001045696 | 6.039878254 |
| hsa_circ_0068325_CBC1 | 0.000171873 | 2.51194608 |
| hsa_circ_0008856_CBC1 | 0.000912428 | 2.689700977 |
| hsa_circ_0039010_CBC1 | 0.000660361 | 3.838113547 |
| hsa_circ_0036902_CBC1 | 0.012088722 | 2.555108337 |
| hsa-circRNA13526-1_CBC1 | 0.003105729 | 2.967329021 |
| hsa_circ_0026198_CBC1 | 5.87439E-05 | 5.688187128 |

*CircRNA* circular RNA

| **Table S2 Downregulated circRNAs between cocultured group and non-cocultured group** | | |
| --- | --- | --- |
| **ProbeName** | **P** | **FC (abs)** |
| hsa_circ_0126779_CBC1 | 0.038542478 | 2.56818661 |
| hsa_circ_0102179_CBC1 | 0.000452175 | 2.120031806 |
| hsa_circ_0079673_CBC1 | 0.000168854 | 3.66533717 |
| hsa_circ_0055287_CBC1 | 0.001411924 | 7.481492943 |
| hsa_circ_0007456_CBC1 | 0.026317147 | 2.209921448 |
| hsa_circ_0106646_CBC1 | 0.014085933 | 2.103797622 |
| hsa_circ_0016987_CBC1 | 0.001978175 | 2.167982765 |
| hsa_circ_0016996_CBC1 | 0.000199205 | 2.52881413 |
| hsa-circRNA7021-8_CBC1 | 9.99172E-05 | 3.77553773 |
| hsa-circRNA4925-11_CBC1 | 0.00461525 | 2.059155651 |
| hsa_circ_0043636_CBC1 | 0.03728221 | 3.382014645 |
| hsa_circ_0011172_CBC1 | 0.00474818 | 2.22773067 |
| hsa-circRNA15044-1_CBC1 | 0.001931041 | 2.141538486 |
| hsa_circ_0092150_CBC1 | 0.021405283 | 3.620342716 |
| hsa-circRNA13870-7_CBC1 | 0.029731594 | 2.448542362 |
| hsa_circ_0118250_CBC1 | 0.00696194 | 2.214275721 |
| hsa-circRNA5843-1_CBC1 | 0.001015923 | 2.456733847 |
| hsa_circ_0055862_CBC1 | 0.000287212 | 2.559639863 |
| hsa_circ_0086217_CBC1 | 0.013170659 | 2.278529251 |
| hsa_circ_0099944_CBC1 | 0.02844683 | 2.074908491 |
| hsa_circ_0111846_CBC1 | 0.017214863 | 2.025339329 |
| hsa_circ_0016489_CBC1 | 0.005618723 | 2.980472756 |
| hsa_circ_0120026_CBC1 | 0.0031118 | 2.252052298 |
| hsa_circ_0101694_CBC1 | 0.004691855 | 2.363285371 |
| hsa_circ_0086457_CBC1 | 0.001774118 | 2.752670003 |
| hsa_circ_0001216_CBC1 | 0.000934007 | 3.806823519 |
| hsa_circ_0045271_CBC1 | 0.002438412 | 2.219422011 |
| hsa-circRNA9834-41_CBC1 | 0.000637554 | 2.395824684 |
| hsa_circ_0098623_CBC1 | 0.025123001 | 2.23938608 |
| hsa_circ_0077769_CBC1 | 0.002400764 | 3.956316149 |
| hsa_circ_0040194_CBC1 | 0.000181109 | 2.142255904 |
| hsa-circRNA14989-11_CBC1 | 0.010036879 | 2.944646606 |
| hsa_circ_0084135_CBC1 | 0.000342523 | 2.367033296 |
| hsa_circ_0088564_CBC1 | 0.000352814 | 4.182675333 |
| hsa_circ_0093637_CBC1 | 0.045830139 | 3.189466542 |
| hsa_circ_0015659_CBC1 | 0.010035456 | 2.702592678 |
| hsa_circ_0084523_CBC1 | 0.000121606 | 2.797953121 |
| hsa_circ_0112684_CBC1 | 0.041487487 | 2.162704761 |
| hsa_circ_0061266_CBC1 | 0.001880001 | 3.241044987 |
| hsa-circRNA14494-2_CBC1 | 0.012330966 | 2.355549979 |
| hsa-circRNA6235-7_CBC1 | 0.005871267 | 2.008463199 |
| hsa_circ_0030251_CBC1 | 0.041947636 | 2.055240345 |
| hsa_circ_0027401_CBC1 | 0.004077631 | 3.437639874 |
| hsa_circ_0040167_CBC1 | 3.14317E-05 | 2.444589257 |
| hsa-circRNA7172-4_CBC1 | 0.013082599 | 2.633979064 |
| hsa_circ_0076603_CBC1 | 0.000737192 | 2.294929424 |
| hsa-circRNA968-3_CBC1 | 7.77796E-05 | 8.429239541 |
| hsa-circRNA9844-18_CBC1 | 0.004169902 | 2.028667605 |
| hsa_circ_0113070_CBC1 | 0.000107136 | 2.113822102 |
| hsa_circ_0088188_CBC1 | 0.012409107 | 2.030995707 |
| hsa_circ_0055868_CBC1 | 0.000986194 | 2.319071321 |
| hsa_circ_0052153_CBC1 | 0.042299793 | 2.763978526 |
| hsa_circ_0130388_CBC1 | 0.003435993 | 2.330619513 |
| hsa-circRNA7362-2_CBC1 | 0.013416795 | 3.20241937 |
| hsa_circ_0025857_CBC1 | 0.017780841 | 2.798213334 |
| hsa_circ_0040794_CBC1 | 7.22162E-05 | 3.407750542 |
| hsa_circ_0094714_CBC1 | 0.042370114 | 2.008352803 |
| hsa_circ_0007739_CBC1 | 3.60121E-06 | 2.599082102 |
| hsa_circ_0025975_CBC1 | 0.008360139 | 2.217025906 |
| hsa_circ_0119530_CBC1 | 0.01264719 | 2.107521892 |
| hsa_circ_0006604_CBC1 | 0.006648125 | 2.15356161 |
| hsa_circ_0095452_CBC1 | 0.002791074 | 2.020799437 |
| hsa_circ_0130726_CBC1 | 0.031414103 | 2.036248159 |
| hsa-circRNA11964_CBC1 | 0.033879177 | 2.090469163 |
| hsa-circRNA8991-66_CBC1 | 0.001173968 | 2.16231482 |
| hsa_circ_0104407_CBC1 | 0.001625432 | 2.083299016 |
| hsa_circ_0001188_CBC1 | 0.000132869 | 2.217854626 |
| hsa_circ_0045267_CBC1 | 0.002728443 | 2.380378391 |
| hsa_circ_0075623_CBC1 | 0.009697907 | 2.98849868 |
| hsa_circ_0013880_CBC1 | 0.00072118 | 3.842498564 |
| hsa-circRNA11158-38_CBC1 | 0.000338293 | 2.902876227 |
| hsa_circ_0113069_CBC1 | 0.000581439 | 2.116948195 |
| hsa-circRNA11420-2_CBC1 | 0.00394577 | 2.169513795 |
| hsa_circ_0058209_CBC1 | 0.005577803 | 2.37455209 |
| hsa_circ_0040804_CBC1 | 7.30092E-05 | 4.003309551 |
| hsa_circ_0040192_CBC1 | 4.33157E-05 | 2.801316195 |
| hsa-circRNA15492-4_CBC1 | 0.000859915 | 2.623917912 |
| hsa_circ_0074411_CBC1 | 0.00760229 | 2.052998011 |
| hsa_circ_0070762_CBC1 | 0.00164901 | 2.667481337 |
| hsa_circ_0133552_CBC1 | 0.024553971 | 3.150163388 |
| hsa_circ_0101557_CBC1 | 0.001836609 | 2.004371455 |
| hsa_circ_0102081_CBC1 | 0.014154 | 2.525607153 |
| hsa_circ_0126351_CBC1 | 0.005715412 | 3.033730511 |
| hsa-circRNA7224-3_CBC1 | 0.001037925 | 2.407449497 |
| hsa_circ_0112307_CBC1 | 0.00828085 | 2.403322218 |
| hsa-circRNA2283-18_CBC1 | 0.010417646 | 2.042036804 |
| hsa_circ_0055259_CBC1 | 0.014482136 | 2.315821035 |
| hsa_circ_0110250_CBC1 | 0.00987683 | 5.293871164 |
| hsa-circRNA1853-25_CBC1 | 0.000101635 | 3.943793725 |
| hsa_circ_0095362_CBC1 | 0.012827866 | 2.225722351 |
| hsa_circ_0054971_CBC1 | 0.000425459 | 2.187253136 |
| hsa_circ_0054657_CBC1 | 0.029454109 | 3.166766898 |
| hsa_circ_0080793_CBC1 | 0.006153671 | 4.348627205 |
| hsa_circ_0087486_CBC1 | 0.006711026 | 2.193326309 |
| hsa-circRNA2233-8_CBC1 | 0.002975888 | 2.950064287 |
| hsa-circRNA15292-17_CBC1 | 0.000919576 | 2.278364728 |
| hsa_circ_0084522_CBC1 | 0.001521751 | 3.020156397 |
| hsa_circ_0045268_CBC1 | 0.000326251 | 2.100684109 |
| hsa_circ_0011446_CBC1 | 6.19157E-05 | 2.591193117 |
| hsa_circ_0022817_CBC1 | 0.003146471 | 2.041224259 |
| hsa_circ_0094695_CBC1 | 0.010180676 | 2.633869903 |
| hsa_circ_0059575_CBC1 | 0.000530176 | 2.194529719 |
| hsa_circ_0083615_CBC1 | 0.001084074 | 2.717891718 |
| hsa_circ_0068491_CBC1 | 0.009665282 | 2.287486336 |
| hsa_circ_0096552_CBC1 | 0.010387769 | 2.246747828 |
| hsa-circRNA15822-21_CBC1 | 0.007680496 | 2.204244656 |
| hsa_circ_0083433_CBC1 | 0.005681917 | 2.800692039 |
| hsa_circ_0040160_CBC1 | 0.000442429 | 2.357833939 |
| hsa_circ_0040185_CBC1 | 8.13251E-05 | 3.09276702 |
| hsa_circ_0033191_CBC1 | 0.000628635 | 2.377965702 |
| hsa_circ_0112725_CBC1 | 0.012537062 | 2.372594747 |
| hsa_circ_0075611_CBC1 | 0.017454785 | 3.309249857 |
| hsa_circ_0040201_CBC1 | 9.64011E-05 | 2.981505058 |
| hsa_circ_0079543_CBC1 | 0.009627266 | 3.177825581 |
| hsa_circ_0107958_CBC1 | 0.009458932 | 2.27979992 |
| hsa_circ_0078355_CBC1 | 0.000697789 | 3.364970002 |
| hsa-circRNA14441-13_CBC1 | 0.003696822 | 2.176454044 |
| hsa_circ_0137524_CBC1 | 0.007436728 | 2.057122082 |
| hsa_circ_0121221_CBC1 | 5.07245E-05 | 2.154118947 |
| hsa_circ_0074372_CBC1 | 0.000797028 | 3.127794775 |
| hsa_circ_0138823_CBC1 | 0.026139739 | 2.348804607 |
| hsa-circRNA15492-3_CBC1 | 0.001568439 | 2.930885719 |
| hsa_circ_0047257_CBC1 | 0.023551904 | 2.640289536 |
| hsa_circ_0083429_CBC1 | 0.012731462 | 3.191391495 |
| hsa_circ_0058210_CBC1 | 0.038881711 | 2.877165168 |
| hsa_circ_0132784_CBC1 | 0.037974101 | 2.020516118 |
| hsa_circ_0016929_CBC1 | 0.000747709 | 2.276188714 |
| hsa-circRNA9643-17_CBC1 | 0.000227873 | 2.035897431 |
| hsa_circ_0124469_CBC1 | 0.009226708 | 3.750975139 |
| hsa_circ_0134059_CBC1 | 0.01357908 | 2.185052123 |
| hsa_circ_0027415_CBC1 | 6.25189E-05 | 3.26264911 |
| hsa_circ_0067718_CBC1 | 0.008869078 | 2.036446032 |
| hsa_circ_0121459_CBC1 | 0.006506048 | 10.54927004 |
| hsa-circRNA10328-6_CBC1 | 0.022550372 | 2.063762263 |
| hsa-circRNA5961_CBC1 | 0.007832665 | 2.195490556 |
| hsa_circ_0096071_CBC1 | 0.000480504 | 4.107807117 |
| hsa_circ_0055286_CBC1 | 0.000114656 | 4.652267818 |
| hsa_circ_0114146_CBC1 | 0.017649125 | 2.139745289 |
| hsa_circ_0047721_CBC1 | 0.002738756 | 2.042909887 |
| hsa_circ_0132161_CBC1 | 0.000422562 | 2.35559855 |
| hsa_circ_0013883_CBC1 | 0.012400901 | 2.175512172 |
| hsa_circ_0043643_CBC1 | 0.005997462 | 2.423303747 |
| hsa_circ_0034411_CBC1 | 0.013891893 | 3.881678902 |
| hsa_circ_0025770_CBC1 | 7.50811E-05 | 8.821067458 |
| hsa_circ_0070977_CBC1 | 0.001413185 | 8.387742973 |
| hsa_circ_0084133_CBC1 | 0.00379293 | 2.300910215 |
| hsa_circ_0058506_CBC1 | 0.001402036 | 2.893643545 |
| hsa_circ_0000233_CBC1 | 0.003195228 | 2.20860228 |
| hsa_circ_0022588_CBC1 | 0.000147138 | 4.270555568 |
| hsa_circ_0059439_CBC1 | 0.003817729 | 2.074254972 |
| hsa-circRNA1663_CBC1 | 0.035001084 | 3.130852158 |
| hsa_circ_0085417_CBC1 | 0.032743389 | 5.706329711 |
| hsa_circ_0017280_CBC1 | 0.034029677 | 2.050540249 |
| hsa_circ_0121356_CBC1 | 0.003351295 | 2.009006824 |
| hsa_circ_0103924_CBC1 | 0.001700717 | 3.768402636 |
| hsa_circ_0127134_CBC1 | 0.001573772 | 2.536539979 |
| hsa-circRNA15822-43_CBC1 | 0.001545544 | 2.219316498 |
| hsa_circ_0101546_CBC1 | 0.005511562 | 2.092482697 |
| hsa_circ_0003428_CBC1 | 0.003552028 | 2.010906745 |
| hsa_circ_0115587_CBC1 | 0.002383193 | 2.086889874 |
| hsa-circRNA8580_CBC1 | 0.011766043 | 2.030781893 |
| hsa_circ_0031740_CBC1 | 0.003757868 | 2.130774948 |
| hsa_circ_0072109_CBC1 | 0.002422122 | 2.043756326 |
| hsa_circ_0062719_CBC1 | 0.003534224 | 4.092653416 |
| hsa_circ_0089107_CBC1 | 0.045114515 | 2.132131928 |
| hsa-circRNA14441-6_CBC1 | 0.019305831 | 2.297148237 |
| hsa_circ_0057537_CBC1 | 0.012573873 | 2.053150984 |
| hsa_circ_0067381_CBC1 | 0.011285089 | 2.066721289 |
| hsa-circRNA165-2_CBC1 | 9.90593E-05 | 3.565568951 |
| hsa_circ_0079716_CBC1 | 3.6714E-05 | 3.308047422 |
| hsa_circ_0002333_CBC1 | 0.00826434 | 2.374773192 |
| hsa_circ_0010704_CBC1 | 0.033901547 | 2.405351654 |
| hsa_circ_0007481_CBC1 | 0.002783846 | 2.036427351 |
| hsa_circ_0082692_CBC1 | 0.000970286 | 3.202860247 |
| hsa_circ_0124599_CBC1 | 0.047384915 | 3.123610076 |
| hsa_circ_0129264_CBC1 | 0.014689182 | 2.130692576 |
| hsa-circRNA6240-2_CBC1 | 0.000247464 | 2.068668935 |
| hsa-circRNA1706-7_CBC1 | 0.025028641 | 4.215814932 |
| hsa_circ_0069908_CBC1 | 0.019522058 | 2.077487045 |
| hsa_circ_0126741_CBC1 | 0.035555988 | 2.687047853 |
| hsa_circ_0084521_CBC1 | 0.000818269 | 2.971851977 |
| hsa_circ_0027402_CBC1 | 9.78987E-05 | 3.481729951 |
| hsa-circRNA8952-4_CBC1 | 0.014147624 | 2.467956921 |
| hsa-circRNA9087-2_CBC1 | 0.018783864 | 2.375344877 |
| hsa_circ_0070363_CBC1 | 0.001582326 | 2.623715693 |
| hsa_circ_0078655_CBC1 | 0.003255039 | 2.396476712 |
| hsa-circRNA1102_CBC1 | 0.001113905 | 2.845244409 |
| hsa-circRNA874-4_CBC1 | 0.036514525 | 2.181368021 |
| hsa_circ_0012908_CBC1 | 0.001102443 | 2.48897162 |
| hsa-circRNA7021-9_CBC1 | 0.000160282 | 3.169436291 |
| hsa_circ_0051638_CBC1 | 0.000119673 | 2.092710878 |
| hsa_circ_0079726_CBC1 | 0.001102775 | 2.742140867 |
| hsa_circ_0129774_CBC1 | 0.008191334 | 2.033106082 |
| hsa_circ_0024152_CBC1 | 0.005940264 | 2.437702659 |
| hsa_circ_0121346_CBC1 | 0.032362592 | 2.551191345 |
| hsa_circ_0079731_CBC1 | 0.000673678 | 2.882893817 |
| hsa_circ_0022583_CBC1 | 0.000377588 | 3.496226429 |
| hsa_circ_0013204_CBC1 | 0.000396714 | 2.06697764 |
| hsa_circ_0125307_CBC1 | 0.00112296 | 2.020170907 |
| hsa-circRNA5612-1_CBC1 | 9.03301E-05 | 2.256037462 |
| hsa-circRNA9603-10_CBC1 | 0.026238422 | 2.088445451 |
| hsa_circ_0089093_CBC1 | 0.034966036 | 2.415287123 |
| hsa_circ_0027400_CBC1 | 0.000426027 | 3.092583319 |
| hsa-circRNA5843-13_CBC1 | 0.001902431 | 2.692033711 |
| hsa_circ_0120011_CBC1 | 0.036299711 | 2.40591529 |
| hsa_circ_0127222_CBC1 | 0.002791101 | 2.203031756 |
| hsa_circ_0003348_CBC1 | 0.001632398 | 3.195396775 |
| hsa-circRNA12990-22_CBC1 | 0.000509858 | 3.554989154 |
| hsa_circ_0023814_CBC1 | 0.048129386 | 2.079423937 |
| hsa_circ_0119607_CBC1 | 0.009314112 | 2.518491514 |
| hsa-circRNA714-14_CBC1 | 0.000777856 | 2.001458541 |
| hsa_circ_0085500_CBC1 | 0.000598902 | 2.123816971 |
| hsa_circ_0064628_CBC1 | 0.005870973 | 2.379654617 |
| hsa_circ_0057843_CBC1 | 0.000405581 | 2.149133911 |
| hsa_circ_0128198_CBC1 | 0.000664902 | 2.914505153 |
| hsa_circ_0004216_CBC1 | 0.001439849 | 3.800481555 |
| hsa_circ_0114830_CBC1 | 8.74872E-05 | 4.178976741 |
| hsa-circRNA7494-1_CBC1 | 0.000979112 | 2.138456623 |
| hsa-circRNA6482-4_CBC1 | 0.00458743 | 3.520851233 |
| hsa_circ_0013882_CBC1 | 1.02074E-05 | 3.08617109 |
| hsa_circ_0025760_CBC1 | 0.000886108 | 2.224645538 |
| hsa_circ_0061680_CBC1 | 0.043792639 | 2.061608764 |
| hsa_circ_0020827_CBC1 | 0.004660249 | 2.273688303 |
| hsa-circRNA9643-13_CBC1 | 0.002675447 | 2.188837544 |
| hsa_circ_0087271_CBC1 | 3.23811E-05 | 11.13100683 |
| hsa_circ_0108312_CBC1 | 0.004262566 | 2.008654677 |
| hsa_circ_0074156_CBC1 | 0.019641263 | 2.233028552 |
| hsa_circ_0040797_CBC1 | 5.03876E-06 | 5.933111121 |
| hsa-circRNA9859-5_CBC1 | 0.002874672 | 2.052468129 |
| hsa_circ_0123497_CBC1 | 0.003034263 | 2.247377475 |
| hsa_circ_0053904_CBC1 | 0.005802553 | 2.162324935 |
| hsa-circRNA12681_CBC1 | 0.007385493 | 4.406389407 |
| hsa_circ_0027416_CBC1 | 2.78851E-05 | 3.787261136 |
| hsa_circ_0007033_CBC1 | 0.003301148 | 2.163804649 |
| hsa-circRNA4392-51_CBC1 | 0.005795285 | 2.101423694 |
| hsa_circ_0130596_CBC1 | 0.030265192 | 2.003056647 |
| hsa-circRNA4420-5_CBC1 | 0.004971837 | 2.49075697 |
| hsa_circ_0086212_CBC1 | 0.005399957 | 2.025478183 |
| hsa_circ_0127506_CBC1 | 0.004941925 | 2.077703493 |
| hsa_circ_0000701_CBC1 | 0.024909209 | 2.192147306 |
| hsa_circ_0110692_CBC1 | 0.000115996 | 3.059003969 |
| hsa_circ_0015646_CBC1 | 5.13541E-05 | 2.300274821 |
| hsa_circ_0023801_CBC1 | 0.012757608 | 2.16097971 |
| hsa_circ_0114145_CBC1 | 0.028975271 | 2.291533774 |
| hsa_circ_0074367_CBC1 | 0.000669182 | 3.785812463 |
| hsa_circ_0055267_CBC1 | 0.010357692 | 2.949961079 |
| hsa_circ_0087045_CBC1 | 0.008609373 | 2.267709189 |
| hsa-circRNA8631-9_CBC1 | 0.002066323 | 2.263185363 |
| hsa-circRNA13203-1_CBC1 | 0.013823098 | 2.825058028 |
| hsa_circ_0126739_CBC1 | 0.031702074 | 2.2754737 |
| hsa_circ_0068488_CBC1 | 0.02139861 | 5.440449212 |
| hsa_circ_0086453_CBC1 | 0.002179351 | 2.740782837 |
| hsa_circ_0079544_CBC1 | 0.034830466 | 2.670655852 |
| hsa_circ_0020014_CBC1 | 0.028819732 | 2.091209944 |
| hsa_circ_0022563_CBC1 | 0.011689521 | 2.257890886 |
| hsa_circ_0102591_CBC1 | 0.0001947 | 2.283233854 |
| hsa_circ_0122157_CBC1 | 0.005748535 | 2.380169684 |
| hsa-circRNA4630-6_CBC1 | 0.001503352 | 2.33676618 |
| hsa-circRNA9410-3_CBC1 | 0.000285981 | 2.735250106 |
| hsa-circRNA7639-37_CBC1 | 0.015474355 | 3.267914767 |
| hsa_circ_0045269_CBC1 | 0.012848546 | 2.142894157 |
| hsa_circ_0103392_CBC1 | 0.02138197 | 3.353688755 |
| hsa-circRNA15925-1_CBC1 | 0.000436837 | 4.60296843 |
| hsa_circ_0007710_CBC1 | 0.000380338 | 2.133173787 |
| hsa_circ_0005025_CBC1 | 0.01338996 | 2.699879381 |
| hsa-circRNA1363-13_CBC1 | 0.000464193 | 3.707493649 |
| hsa_circ_0013881_CBC1 | 5.74011E-05 | 2.784240337 |
| hsa_circ_0113009_CBC1 | 0.003478977 | 2.142545643 |
| hsa-circRNA14469-6_CBC1 | 0.016158856 | 2.003545048 |
| hsa-circRNA6293-5_CBC1 | 0.000160195 | 8.44458708 |
| hsa_circ_0114343_CBC1 | 0.002114905 | 2.038169421 |
| hsa-circRNA9371-4_CBC1 | 0.000947577 | 2.355730514 |
| hsa_circ_0051634_CBC1 | 0.00053941 | 2.185496744 |
| hsa_circ_0023812_CBC1 | 0.000440952 | 2.043670595 |
| hsa_circ_0131469_CBC1 | 0.002572037 | 3.374148091 |
| hsa_circ_0011440_CBC1 | 0.000453841 | 2.384697157 |
| hsa-circRNA8046-2_CBC1 | 0.005847942 | 2.542642631 |
| hsa_circ_0114327_CBC1 | 0.000136404 | 2.852551784 |
| hsa_circ_0084516_CBC1 | 0.000802776 | 3.546727432 |
| hsa_circ_0095234_CBC1 | 0.016923022 | 2.412145356 |
| hsa_circ_0075828_CBC1 | 3.61563E-05 | 2.810309529 |
| hsa_circ_0126143_CBC1 | 0.005048279 | 3.381869447 |
| hsa_circ_0117170_CBC1 | 0.018323863 | 2.083183422 |
| hsa_circ_0033181_CBC1 | 0.000246246 | 2.147444381 |
| hsa-circRNA7021-3_CBC1 | 0.029756943 | 3.356510554 |
| hsa_circ_0039857_CBC1 | 0.039157727 | 2.025330358 |
| hsa_circ_0047302_CBC1 | 0.000820228 | 2.300224842 |
| hsa-circRNA13052-7_CBC1 | 0.002172977 | 2.298084682 |
| hsa-circRNA13643_CBC1 | 0.003583389 | 2.379952818 |
| hsa_circ_0087483_CBC1 | 0.001613327 | 2.334590336 |
| hsa_circ_0135201_CBC1 | 0.004221256 | 3.507650231 |
| hsa-circRNA6109-1_CBC1 | 0.001291865 | 2.037322667 |
| hsa_circ_0128189_CBC1 | 0.022878479 | 3.169117097 |
| hsa-circRNA12990-17_CBC1 | 0.004868723 | 2.259475969 |
| hsa_circ_0046282_CBC1 | 0.013311672 | 2.790417179 |
| hsa_circ_0102294_CBC1 | 0.040411927 | 2.262498314 |
| hsa-circRNA482-154_CBC1 | 5.40158E-05 | 2.77380466 |
| hsa_circ_0055052_CBC1 | 0.004840615 | 2.075552456 |
| hsa_circ_0134314_CBC1 | 0.048914427 | 2.091962926 |
| hsa_circ_0035372_CBC1 | 0.003840744 | 2.067113125 |
| hsa-circRNA2403-4_CBC1 | 0.001983005 | 2.138157465 |
| hsa_circ_0040798_CBC1 | 2.55504E-05 | 4.507324046 |
| hsa_circ_0128188_CBC1 | 0.006160523 | 3.623089245 |
| hsa_circ_0040161_CBC1 | 0.008069048 | 2.598301277 |
| hsa_circ_0135616_CBC1 | 7.89255E-08 | 2.703220543 |
| hsa_circ_0030394_CBC1 | 0.005569491 | 2.924079769 |
| hsa_circ_0089102_CBC1 | 0.038514591 | 2.203561888 |
| hsa_circ_0139640_CBC1 | 0.000616648 | 2.009138781 |
| hsa_circ_0073202_CBC1 | 0.001995135 | 3.084902851 |
| hsa_circ_0094622_CBC1 | 0.00303663 | 2.222189426 |
| hsa_circ_0090910_CBC1 | 0.02888086 | 2.512659498 |
| hsa_circ_0051382_CBC1 | 0.005866649 | 2.16093046 |
| hsa_circ_0067955_CBC1 | 0.00020621 | 2.09804702 |
| hsa_circ_0126149_CBC1 | 0.045920341 | 2.200044858 |
| hsa_circ_0115741_CBC1 | 0.015341079 | 3.521722823 |
| hsa_circ_0016485_CBC1 | 0.000795218 | 2.869734685 |
| hsa_circ_0019066_CBC1 | 0.00665904 | 5.498937509 |
| hsa_circ_0061666_CBC1 | 0.000533147 | 2.181755721 |
| hsa_circ_0091372_CBC1 | 0.000898049 | 2.137058594 |
| hsa-circRNA15681-10_CBC1 | 5.06631E-05 | 2.869030988 |
| hsa-circRNA14936_CBC1 | 0.013345812 | 2.05633053 |
| hsa_circ_0070371_CBC1 | 0.000479744 | 2.412008271 |
| hsa_circ_0140215_CBC1 | 0.002258447 | 6.195958683 |
| hsa_circ_0069865_CBC1 | 0.009835231 | 2.450662637 |
| hsa-circRNA4925-13_CBC1 | 0.002360235 | 2.170707871 |
| hsa_circ_0005628_CBC1 | 0.001742544 | 2.070121663 |
| hsa_circ_0014182_CBC1 | 0.00092772 | 2.039032799 |
| hsa-circRNA714-2_CBC1 | 7.90132E-05 | 2.011046607 |
| hsa_circ_0075061_CBC1 | 0.004621727 | 2.070265947 |
| hsa_circ_0015658_CBC1 | 0.020101127 | 2.654150035 |
| hsa_circ_0068460_CBC1 | 0.00433692 | 2.762362393 |
| hsa_circ_0055044_CBC1 | 0.012933839 | 2.022023211 |
| hsa-circRNA4561-16_CBC1 | 0.000749461 | 2.481159376 |
| hsa-circRNA14293-11_CBC1 | 0.01465773 | 2.241689671 |
| hsa_circ_0120815_CBC1 | 0.001219564 | 2.123779705 |
| hsa_circ_0087490_CBC1 | 0.0047147 | 2.462072641 |
| hsa_circ_0113071_CBC1 | 2.71051E-06 | 2.225907551 |
| hsa_circ_0070904_CBC1 | 0.001775264 | 2.673964561 |
| hsa-circRNA4561-4_CBC1 | 0.00523698 | 2.283092793 |
| hsa_circ_0094354_CBC1 | 0.001658064 | 2.164925099 |
| hsa-circRNA14983-1_CBC1 | 0.001568072 | 2.446576374 |
| hsa_circ_0093561_CBC1 | 0.002230763 | 2.300592038 |
| hsa_circ_0022556_CBC1 | 0.00374742 | 2.034422215 |
| hsa_circ_0113944_CBC1 | 0.001762817 | 2.173621805 |
| hsa_circ_0124859_CBC1 | 0.011446229 | 2.05829141 |
| hsa-circRNA1853-6_CBC1 | 6.6662E-05 | 3.360567997 |
| hsa-circRNA14469-12_CBC1 | 0.007919321 | 2.010578085 |
| hsa-circRNA14615-2_CBC1 | 0.031810422 | 2.001854588 |
| hsa_circ_0055864_CBC1 | 0.001115855 | 2.450994559 |
| hsa-circRNA8604-12_CBC1 | 0.001298491 | 2.341795883 |
| hsa_circ_0126751_CBC1 | 0.008332205 | 3.450537857 |
| hsa-circRNA8952-11_CBC1 | 0.000778634 | 2.317349919 |
| hsa_circ_0031891_CBC1 | 0.007642573 | 2.163400753 |
| hsa_circ_0019068_CBC1 | 0.004072464 | 2.68779458 |
| hsa_circ_0036955_CBC1 | 1.77686E-05 | 2.199423888 |
| hsa_circ_0031580_CBC1 | 0.013244472 | 2.087132131 |
| hsa_circ_0004932_CBC1 | 0.046770336 | 2.081099878 |
| hsa-circRNA8783-21_CBC1 | 4.87525E-05 | 7.765522838 |
| hsa_circ_0078649_CBC1 | 0.002509604 | 2.228639947 |
| hsa_circ_0055280_CBC1 | 0.000174174 | 4.369405873 |
| hsa_circ_0082093_CBC1 | 0.000159952 | 2.884366771 |
| hsa_circ_0081986_CBC1 | 0.014571048 | 2.105639451 |
| hsa-circRNA12528_CBC1 | 0.000359992 | 2.029643195 |
| hsa_circ_0027421_CBC1 | 8.16757E-05 | 3.247179661 |
| hsa_circ_0122154_CBC1 | 0.000880855 | 2.3570598 |
| hsa_circ_0086167_CBC1 | 0.045421133 | 3.458946583 |
| hsa-circRNA7224-5_CBC1 | 0.004184151 | 2.699845911 |
| hsa-circRNA1853-29_CBC1 | 6.62488E-05 | 3.860999622 |
| hsa_circ_0070970_CBC1 | 0.000699536 | 10.75829945 |
| hsa_circ_0074362_CBC1 | 0.003058793 | 2.310393532 |
| hsa_circ_0007868_CBC1 | 0.000258794 | 2.814716494 |
| hsa_circ_0060441_CBC1 | 0.00386238 | 2.267912032 |
| hsa_circ_0105853_CBC1 | 5.46485E-06 | 2.893756739 |
| hsa_circ_0033186_CBC1 | 0.001541026 | 2.533312284 |
| hsa_circ_0125256_CBC1 | 0.001958723 | 2.480128416 |
| hsa_circ_0051619_CBC1 | 0.008097402 | 2.344659746 |
| hsa_circ_0097070_CBC1 | 0.005501317 | 2.343455826 |
| hsa_circ_0129840_CBC1 | 0.00320304 | 2.768569524 |
| hsa_circ_0087493_CBC1 | 0.001413179 | 2.135116778 |
| hsa_circ_0073216_CBC1 | 0.000698908 | 3.501722883 |
| hsa_circ_0129457_CBC1 | 0.027107316 | 2.063734651 |
| hsa_circ_0013884_CBC1 | 5.30249E-05 | 3.161808215 |
| hsa_circ_0026910_CBC1 | 0.003612882 | 2.339383705 |
| hsa-circRNA9834-19_CBC1 | 0.002968503 | 2.08090914 |
| hsa_circ_0000121_CBC1 | 0.000151073 | 2.187702846 |
| hsa_circ_0093333_CBC1 | 0.011901792 | 2.319940234 |
| hsa_circ_0096526_CBC1 | 0.000830025 | 2.09285915 |
| hsa_circ_0047266_CBC1 | 0.027238797 | 4.370040739 |
| hsa_circ_0025768_CBC1 | 0.001260857 | 3.661393935 |
| hsa_circ_0127563_CBC1 | 0.040621685 | 2.37854194 |
| hsa_circ_0040159_CBC1 | 0.000778789 | 3.066827816 |
| hsa_circ_0096523_CBC1 | 0.001592796 | 2.299279065 |
| hsa_circ_0128191_CBC1 | 0.000640014 | 2.181267945 |
| hsa_circ_0045279_CBC1 | 0.000643811 | 2.065649017 |
| hsa_circ_0098596_CBC1 | 0.04327112 | 2.033881151 |
| hsa_circ_0027411_CBC1 | 0.002216994 | 4.189291319 |
| hsa_circ_0032039_CBC1 | 0.002895179 | 2.704166132 |
| hsa_circ_0040180_CBC1 | 2.0494E-05 | 2.871306837 |
| hsa_circ_0078848_CBC1 | 0.006043172 | 2.160685929 |
| hsa_circ_0127221_CBC1 | 0.000856096 | 2.678745921 |
| hsa_circ_0117517_CBC1 | 0.010237539 | 2.07609726 |
| hsa_circ_0000123_CBC1 | 0.000572016 | 3.835764142 |
| hsa-circRNA9533-14_CBC1 | 0.001062715 | 2.315641355 |
| hsa-circRNA15822-22_CBC1 | 0.000475275 | 2.176008327 |
| hsa_circ_0040174_CBC1 | 3.58434E-05 | 2.904699446 |
| hsa_circ_0068459_CBC1 | 0.003611386 | 2.30434144 |
| hsa-circRNA8420-13_CBC1 | 0.000502702 | 2.253343925 |
| hsa-circRNA9807-15_CBC1 | 0.002098513 | 3.246365876 |
| hsa_circ_0018143_CBC1 | 0.007385187 | 2.296782766 |
| hsa_circ_0112834_CBC1 | 0.034985172 | 2.546202491 |
| hsa_circ_0013375_CBC1 | 0.001097659 | 2.54725011 |
| hsa_circ_0003053_CBC1 | 0.008455604 | 2.983040496 |
| hsa_circ_0140210_CBC1 | 0.001018639 | 5.483609804 |
| hsa_circ_0016405_CBC1 | 0.000262174 | 2.156466875 |
| hsa-circRNA6610-7_CBC1 | 0.014457483 | 2.659980878 |
| hsa_circ_0125212_CBC1 | 0.007736581 | 2.003385509 |
| hsa_circ_0085942_CBC1 | 0.038467635 | 3.119571808 |
| hsa_circ_0066391_CBC1 | 0.00144868 | 2.676689284 |
| hsa_circ_0019065_CBC1 | 0.010791116 | 5.167205557 |
| hsa_circ_0056700_CBC1 | 0.001458234 | 2.04960589 |
| hsa-circRNA12990-19_CBC1 | 0.001092449 | 2.187980297 |
| hsa_circ_0079540_CBC1 | 0.002547944 | 2.741775005 |
| hsa_circ_0055255_CBC1 | 0.034302391 | 2.592169646 |
| hsa-circRNA8046-12_CBC1 | 0.029834978 | 2.600563615 |
| hsa_circ_0130269_CBC1 | 0.000197031 | 5.157418726 |
| hsa_circ_0017013_CBC1 | 0.019483513 | 2.179723311 |
| hsa_circ_0026912_CBC1 | 0.013292429 | 2.328484008 |
| hsa-circRNA2747-14_CBC1 | 0.00134005 | 2.02954335 |
| hsa_circ_0017912_CBC1 | 0.010718434 | 2.59247324 |
| hsa_circ_0131898_CBC1 | 0.003385882 | 2.16004634 |
| hsa_circ_0093563_CBC1 | 0.002193878 | 2.783336814 |
| hsa_circ_0060315_CBC1 | 0.026010444 | 2.473782236 |
| hsa_circ_0125672_CBC1 | 0.000707376 | 2.078051168 |
| hsa_circ_0137518_CBC1 | 0.016033636 | 2.020179519 |
| hsa-circRNA6630-15_CBC1 | 0.000163478 | 2.889268048 |
| hsa-circRNA9547_CBC1 | 0.013159694 | 2.142316764 |
| hsa_circ_0131855_CBC1 | 0.003760555 | 2.295185495 |
| hsa_circ_0069588_CBC1 | 0.003782717 | 2.012319927 |
| hsa_circ_0027406_CBC1 | 0.000235829 | 3.305170863 |
| hsa-circRNA8952-14_CBC1 | 9.41016E-05 | 2.83056672 |
| hsa_circ_0070367_CBC1 | 0.000548697 | 2.1660051 |
| hsa_circ_0125898_CBC1 | 0.002045068 | 2.015253806 |
| hsa_circ_0016486_CBC1 | 0.003252025 | 2.862984492 |
| hsa-circRNA15082-10_CBC1 | 6.97982E-05 | 5.128924703 |
| hsa-circRNA4925-2_CBC1 | 8.20346E-05 | 2.02510766 |
| hsa_circ_0087047_CBC1 | 0.006129176 | 2.033238817 |
| hsa-circRNA5151-9_CBC1 | 0.019143031 | 2.116797165 |
| hsa_circ_0070971_CBC1 | 0.000587939 | 7.629612472 |
| hsa_circ_0061222_CBC1 | 0.029952714 | 2.248867684 |
| hsa_circ_0006075_CBC1 | 0.002659341 | 2.376131793 |
| hsa_circ_0011177_CBC1 | 0.001600662 | 2.185242576 |
| hsa-circRNA16083-2_CBC1 | 0.009845829 | 7.355358045 |
| hsa_circ_0128614_CBC1 | 0.001555987 | 2.14197218 |
| hsa_circ_0033185_CBC1 | 0.000285462 | 2.323566013 |
| hsa_circ_0020825_CBC1 | 0.000907174 | 2.512521622 |
| hsa_circ_0059576_CBC1 | 8.41188E-05 | 2.284836449 |
| hsa-circRNA3173-2_CBC1 | 0.003829267 | 2.375440905 |
| hsa_circ_0095241_CBC1 | 0.004548731 | 2.780278569 |
| hsa_circ_0120813_CBC1 | 0.002964998 | 2.062501297 |
| hsa_circ_0025417_CBC1 | 0.000503666 | 2.030568435 |
| hsa_circ_0034413_CBC1 | 0.016911362 | 2.732560751 |
| hsa_circ_0122570_CBC1 | 0.014203566 | 2.012604995 |
| hsa_circ_0074397_CBC1 | 0.007042089 | 2.097021509 |
| hsa_circ_0097109_CBC1 | 6.98812E-05 | 5.550446006 |
| hsa_circ_0000091_CBC1 | 5.9596E-05 | 2.021831436 |
| hsa-circRNA3903-2_CBC1 | 0.013046995 | 2.158301329 |
| hsa_circ_0011445_CBC1 | 0.000204538 | 2.178332702 |
| hsa_circ_0129783_CBC1 | 0.002451353 | 2.100787122 |
| hsa_circ_0110255_CBC1 | 0.001308774 | 5.013673948 |
| hsa_circ_0103920_CBC1 | 0.000374622 | 4.380291315 |
| hsa_circ_0061664_CBC1 | 0.0128627 | 2.491524214 |
| hsa_circ_0073218_CBC1 | 0.00396938 | 2.917215482 |
| hsa_circ_0126113_CBC1 | 0.001257341 | 2.371261898 |
| hsa_circ_0025463_CBC1 | 0.004884426 | 2.14248985 |
| hsa_circ_0041340_CBC1 | 0.010054268 | 3.861274096 |
| hsa_circ_0087505_CBC1 | 0.004014726 | 2.199253657 |
| hsa_circ_0074377_CBC1 | 0.000668307 | 2.053888494 |
| hsa_circ_0008263_CBC1 | 0.0198291 | 2.921205754 |
| hsa-circRNA5151-45_CBC1 | 0.012535045 | 2.085722568 |
| hsa_circ_0061397_CBC1 | 0.006038959 | 3.093600164 |
| hsa-circRNA14993-26_CBC1 | 0.023958031 | 2.064425026 |
| hsa_circ_0083941_CBC1 | 0.038058578 | 2.315862065 |
| hsa_circ_0059435_CBC1 | 0.000437997 | 2.282817917 |
| hsa_circ_0047260_CBC1 | 0.012139632 | 2.004293617 |
| hsa_circ_0107908_CBC1 | 0.001405036 | 2.60397671 |
| hsa_circ_0127305_CBC1 | 0.026416682 | 3.282908307 |
| hsa_circ_0015660_CBC1 | 0.002109544 | 2.587271819 |
| hsa_circ_0016293_CBC1 | 0.022080439 | 2.259886749 |
| hsa-circRNA12990-28_CBC1 | 0.001067948 | 2.794935118 |
| hsa-circRNA8604-8_CBC1 | 0.00155393 | 2.718249587 |
| hsa_circ_0005643_CBC1 | 0.001531296 | 2.217151681 |
| hsa_circ_0022561_CBC1 | 0.001280992 | 2.15881673 |
| hsa-circRNA12487-120_CBC1 | 0.023257287 | 2.271132222 |
| hsa_circ_0124602_CBC1 | 0.009182719 | 2.929005182 |
| hsa-circRNA1505-2_CBC1 | 8.42624E-05 | 2.150222465 |
| hsa_circ_0011170_CBC1 | 0.008188448 | 2.269776701 |
| hsa_circ_0082077_CBC1 | 0.001874003 | 2.017748657 |
| hsa_circ_0072572_CBC1 | 0.014707801 | 4.221131799 |
| hsa_circ_0059134_CBC1 | 0.001538039 | 2.527690074 |
| hsa-circRNA10585-3_CBC1 | 5.87608E-05 | 2.292452789 |
| hsa-circRNA15190-4_CBC1 | 0.001684819 | 2.312994676 |
| hsa_circ_0027398_CBC1 | 0.001876375 | 2.917109326 |
| hsa-circRNA15822-7_CBC1 | 0.005516567 | 2.193897184 |
| hsa_circ_0017959_CBC1 | 0.002659715 | 2.107032014 |
| hsa-circRNA4892-3_CBC1 | 0.000292983 | 2.428536811 |
| hsa-circRNA333-4_CBC1 | 0.001076235 | 2.129548801 |
| hsa-circRNA1009-5_CBC1 | 0.018755843 | 2.108029064 |
| hsa_circ_0140213_CBC1 | 0.00755365 | 4.543802568 |
| hsa_circ_0087282_CBC1 | 0.009953209 | 2.371438363 |
| hsa_circ_0117953_CBC1 | 0.02017097 | 2.326948811 |
| hsa-circRNA15808-17_CBC1 | 0.019994602 | 2.038303281 |
| hsa_circ_0051629_CBC1 | 0.000125232 | 2.341572809 |
| hsa-circRNA11158-50_CBC1 | 9.33063E-05 | 3.230382241 |
| hsa-circRNA15822-58_CBC1 | 0.002487472 | 2.4537639 |
| hsa_circ_0078650_CBC1 | 0.001684633 | 2.706290219 |
| hsa_circ_0113003_CBC1 | 0.001025701 | 3.180291125 |
| hsa_circ_0101418_CBC1 | 0.000263104 | 2.713627452 |
| hsa_circ_0117750_CBC1 | 0.014108362 | 5.515643521 |
| hsa-circRNA14135-18_CBC1 | 0.000341504 | 5.88795698 |
| hsa-circRNA8604-5_CBC1 | 0.008169094 | 2.139536995 |
| hsa_circ_0018343_CBC1 | 0.017675643 | 2.093010124 |
| hsa_circ_0095361_CBC1 | 0.006150502 | 2.247404576 |
| hsa-circRNA5626-3_CBC1 | 0.039178059 | 2.199231265 |
| hsa_circ_0073217_CBC1 | 0.002182887 | 2.852829673 |
| hsa_circ_0011435_CBC1 | 0.000608213 | 2.124032904 |
| hsa_circ_0139352_CBC1 | 0.022307651 | 2.444726242 |
| hsa_circ_0098291_CBC1 | 0.003019287 | 2.485941116 |
| hsa-circRNA1853-8_CBC1 | 1.40037E-05 | 3.89645386 |
| hsa_circ_0126157_CBC1 | 0.008305372 | 2.156042801 |
| hsa-circRNA11226-15_CBC1 | 0.000122883 | 4.034671534 |
| hsa_circ_0089101_CBC1 | 0.008771721 | 2.624880246 |
| hsa-circRNA14298-9_CBC1 | 0.028147593 | 2.451093301 |
| hsa_circ_0025930_CBC1 | 0.011575028 | 2.247794003 |
| hsa_circ_0096888_CBC1 | 0.026497183 | 3.128842387 |
| hsa_circ_0089340_CBC1 | 0.001474102 | 2.166593663 |
| hsa-circRNA6884-7_CBC1 | 7.66502E-05 | 7.493407328 |
| hsa_circ_0074366_CBC1 | 0.006426942 | 5.082346092 |
| hsa_circ_0025354_CBC1 | 0.037784585 | 2.653118107 |
| hsa_circ_0012717_CBC1 | 0.014925644 | 2.52337337 |
| hsa_circ_0055285_CBC1 | 0.000269668 | 4.729953693 |
| hsa_circ_0128236_CBC1 | 0.021493313 | 2.06319179 |
| hsa_circ_0055281_CBC1 | 7.05829E-05 | 5.730092581 |
| hsa_circ_0077198_CBC1 | 0.024304886 | 2.146786289 |
| hsa-circRNA15785-2_CBC1 | 5.78634E-05 | 4.25551333 |
| hsa-circRNA14441-29_CBC1 | 0.020239524 | 2.04803244 |
| hsa_circ_0040793_CBC1 | 0.000278734 | 4.535938166 |
| hsa_circ_0120202_CBC1 | 0.003817136 | 2.024564437 |
| hsa_circ_0027409_CBC1 | 0.000303006 | 3.211339075 |
| hsa_circ_0005642_CBC1 | 0.027238734 | 2.871450078 |
| hsa_circ_0031102_CBC1 | 0.00022644 | 2.557136924 |
| hsa_circ_0125254_CBC1 | 0.003847989 | 2.407556852 |
| hsa_circ_0025431_CBC1 | 0.000697736 | 2.061149571 |
| hsa_circ_0129065_CBC1 | 0.000134568 | 3.943576899 |
| hsa_circ_0087478_CBC1 | 0.000143845 | 2.064816956 |
| hsa_circ_0079542_CBC1 | 0.000737175 | 2.328256179 |
| hsa_circ_0053209_CBC1 | 0.027101237 | 2.260632161 |
| hsa-circRNA7224-11_CBC1 | 0.000998672 | 3.490072755 |
| hsa_circ_0125357_CBC1 | 0.000597015 | 9.071495467 |
| hsa_circ_0047264_CBC1 | 0.012671924 | 2.196513475 |
| hsa-circRNA731-4_CBC1 | 0.004354952 | 2.218704826 |
| hsa-circRNA13340-6_CBC1 | 0.022254335 | 2.179262531 |
| hsa_circ_0138431_CBC1 | 0.004550912 | 2.117970608 |
| hsa-circRNA8648-4_CBC1 | 0.001191235 | 2.021352331 |
| hsa_circ_0076804_CBC1 | 0.000179103 | 2.551666504 |
| hsa_circ_0073213_CBC1 | 0.002915276 | 3.38290846 |
| hsa_circ_0051627_CBC1 | 6.66611E-06 | 2.147479203 |
| hsa_circ_0107960_CBC1 | 2.69727E-06 | 2.015383311 |
| hsa_circ_0054344_CBC1 | 0.015103769 | 2.021584132 |
| hsa_circ_0103932_CBC1 | 0.005782485 | 2.030656595 |
| hsa_circ_0083631_CBC1 | 0.023880828 | 2.447677504 |
| hsa-circRNA5406-67_CBC1 | 0.000297126 | 2.096262367 |
| hsa_circ_0012948_CBC1 | 0.024774093 | 2.456753777 |
| hsa_circ_0051628_CBC1 | 4.37202E-06 | 2.117792695 |
| hsa_circ_0031796_CBC1 | 0.00011154 | 2.392412895 |
| hsa_circ_0033182_CBC1 | 0.000705525 | 2.292962328 |
| hsa-circRNA11777-7_CBC1 | 0.010153861 | 2.340642426 |
| hsa_circ_0135686_CBC1 | 0.001545 | 2.897611741 |
| hsa_circ_0100533_CBC1 | 0.045737331 | 4.079532641 |
| hsa-circRNA6883-3_CBC1 | 0.000351619 | 4.134997957 |
| hsa_circ_0134116_CBC1 | 8.4242E-06 | 5.191081476 |
| hsa_circ_0001860_CBC1 | 0.010676532 | 2.108173556 |
| hsa_circ_0120840_CBC1 | 0.000805596 | 2.516735728 |
| hsa_circ_0127225_CBC1 | 0.009099865 | 2.063025791 |
| hsa_circ_0094781_CBC1 | 0.010962298 | 2.498574384 |
| hsa_circ_0125671_CBC1 | 0.00335683 | 3.680297897 |
| hsa_circ_0011864_CBC1 | 0.030456609 | 2.029723665 |
| hsa_circ_0077773_CBC1 | 0.000104851 | 5.925180764 |
| hsa_circ_0040165_CBC1 | 4.75682E-05 | 3.10372597 |
| hsa_circ_0111452_CBC1 | 0.011270967 | 2.159552312 |
| hsa_circ_0125684_CBC1 | 0.043629277 | 2.33744854 |
| hsa_circ_0031745_CBC1 | 0.046291166 | 2.105090708 |
| hsa_circ_0079717_CBC1 | 0.001197564 | 2.57406723 |
| hsa_circ_0055538_CBC1 | 0.001882181 | 2.512988949 |
| hsa_circ_0074403_CBC1 | 0.01249559 | 2.237641907 |
| hsa_circ_0047720_CBC1 | 0.012448877 | 2.458642588 |
| hsa_circ_0027265_CBC1 | 0.001026313 | 2.137076245 |
| hsa-circRNA6924-29_CBC1 | 0.045642924 | 2.071127959 |
| hsa_circ_0047303_CBC1 | 0.000200817 | 2.216932968 |
| hsa_circ_0054447_CBC1 | 0.015670749 | 2.231183727 |
| hsa_circ_0102592_CBC1 | 0.000617682 | 2.257634473 |
| hsa-circRNA8848-24_CBC1 | 0.02112491 | 2.024933561 |
| hsa_circ_0136967_CBC1 | 0.046862313 | 2.03446419 |
| hsa-circRNA2303-1_CBC1 | 0.000890179 | 2.652004372 |
| hsa_circ_0129670_CBC1 | 0.005542184 | 3.499969216 |
| hsa_circ_0012946_CBC1 | 0.005689801 | 2.471762026 |
| hsa_circ_0125539_CBC1 | 0.020045401 | 2.103549032 |
| hsa_circ_0110251_CBC1 | 0.000917648 | 2.66450332 |
| hsa-circRNA11158-19_CBC1 | 7.59071E-05 | 2.835453212 |
| hsa-circRNA7362-14_CBC1 | 0.042005239 | 3.265000697 |
| hsa-circRNA10585-28_CBC1 | 0.000403813 | 2.864989131 |
| hsa_circ_0055859_CBC1 | 0.000314557 | 2.183925881 |
| hsa_circ_0127219_CBC1 | 0.015621395 | 2.214155154 |
| hsa_circ_0033184_CBC1 | 0.000185665 | 2.172854251 |
| hsa_circ_0130266_CBC1 | 0.000340748 | 4.148368375 |
| hsa_circ_0025571_CBC1 | 0.012103193 | 6.496225034 |
| hsa_circ_0110810_CBC1 | 0.007955356 | 2.022639328 |
| hsa_circ_0011447_CBC1 | 0.000218994 | 2.525986245 |
| hsa_circ_0021215_CBC1 | 0.001925475 | 2.334224165 |
| hsa-circRNA2233-5_CBC1 | 0.000173383 | 3.225290195 |
| hsa-circRNA4561-1_CBC1 | 0.000719598 | 2.642738682 |
| hsa_circ_0003909_CBC1 | 0.012813041 | 2.55269099 |
| hsa_circ_0066390_CBC1 | 0.000326794 | 2.567628334 |
| hsa_circ_0098479_CBC1 | 0.046966775 | 2.155940541 |
| hsa_circ_0087482_CBC1 | 0.003592721 | 2.307248345 |
| hsa-circRNA15822-53_CBC1 | 0.000275346 | 2.019529197 |
| hsa_circ_0117944_CBC1 | 0.007235412 | 2.054626962 |
| hsa-circRNA7224-1_CBC1 | 0.0029515 | 2.49645542 |
| hsa_circ_0089109_CBC1 | 0.027790481 | 2.204317693 |
| hsa_circ_0054663_CBC1 | 0.019472638 | 2.331674623 |
| hsa_circ_0061864_CBC1 | 0.003731083 | 2.827384969 |
| hsa_circ_0126756_CBC1 | 0.007800157 | 2.055113233 |
| hsa_circ_0011740_CBC1 | 0.006683971 | 2.112913794 |
| hsa_circ_0031799_CBC1 | 0.009212588 | 2.027446712 |
| hsa_circ_0125311_CBC1 | 0.000513516 | 2.227015189 |
| hsa_circ_0139950_CBC1 | 0.000295827 | 2.729431422 |
| hsa_circ_0104390_CBC1 | 0.009229374 | 2.117221951 |
| hsa_circ_0047244_CBC1 | 0.000430298 | 3.00953839 |
| hsa_circ_0124605_CBC1 | 0.016079088 | 2.540759156 |
| hsa_circ_0134094_CBC1 | 0.002750463 | 2.949724944 |
| hsa_circ_0025352_CBC1 | 0.009293658 | 2.499762395 |
| hsa-circRNA15492-1_CBC1 | 0.002222222 | 2.793794993 |
| hsa_circ_0077998_CBC1 | 0.004017306 | 2.154594329 |
| hsa_circ_0125317_CBC1 | 0.003723345 | 2.253550423 |
| hsa-circRNA165-6_CBC1 | 0.004443529 | 2.694623172 |
| hsa-circRNA8574-3_CBC1 | 0.002021174 | 2.07747192 |
| hsa-circRNA7052-1_CBC1 | 0.000326243 | 2.059689536 |
| hsa_circ_0023804_CBC1 | 0.001071228 | 2.395536105 |
| hsa_circ_0013767_CBC1 | 0.001471061 | 3.89177583 |
| hsa-circRNA14348-21_CBC1 | 0.000746094 | 3.150545448 |
| hsa_circ_0075060_CBC1 | 0.00827375 | 2.187024878 |
| hsa_circ_0059452_CBC1 | 0.00303929 | 2.234998484 |
| hsa_circ_0136575_CBC1 | 0.004976078 | 2.451113804 |
| hsa_circ_0131927_CBC1 | 0.000633044 | 2.046660744 |
| hsa-circRNA5843-15_CBC1 | 0.032816999 | 3.428065909 |
| hsa_circ_0126253_CBC1 | 0.001485163 | 2.259299853 |
| hsa-circRNA10661-4_CBC1 | 0.022299388 | 2.658112738 |
| hsa_circ_0082549_CBC1 | 0.000154289 | 2.037546501 |
| hsa_circ_0009036_CBC1 | 0.005503399 | 2.397003376 |
| hsa_circ_0040204_CBC1 | 0.000110844 | 2.947984473 |
| hsa_circ_0086363_CBC1 | 0.002862156 | 3.560026399 |
| hsa_circ_0076138_CBC1 | 0.01555025 | 2.286770955 |
| hsa_circ_0055043_CBC1 | 0.001965797 | 2.070484277 |
| hsa-circRNA173-10_CBC1 | 0.002560709 | 2.083362157 |
| hsa_circ_0121309_CBC1 | 0.024818789 | 2.553580592 |
| hsa_circ_0114560_CBC1 | 0.018751394 | 2.566474727 |
| hsa_circ_0087043_CBC1 | 0.011951995 | 2.775113857 |
| hsa-circRNA4392-69_CBC1 | 3.09906E-05 | 2.437474855 |
| hsa_circ_0074414_CBC1 | 0.008881297 | 2.096128957 |
| hsa_circ_0062720_CBC1 | 0.003278186 | 3.811302585 |
| hsa_circ_0031590_CBC1 | 0.019916079 | 2.150256168 |
| hsa_circ_0004766_CBC1 | 0.010563212 | 2.408878073 |
| hsa-circRNA15700-2_CBC1 | 0.000747683 | 2.028350809 |
| hsa_circ_0022562_CBC1 | 0.00259704 | 2.312044651 |
| hsa-circRNA12761-1_CBC1 | 0.028170734 | 2.448229983 |
| hsa_circ_0124861_CBC1 | 0.034821576 | 2.090719548 |
| hsa_circ_0103921_CBC1 | 0.000459235 | 4.608197882 |
| hsa_circ_0139339_CBC1 | 0.002016185 | 2.419904013 |
| hsa_circ_0070975_CBC1 | 0.005608096 | 7.773725167 |
| hsa_circ_0072113_CBC1 | 0.013279965 | 2.119159539 |
| hsa_circ_0101550_CBC1 | 0.02010159 | 2.467336081 |
| hsa-circRNA3017-1_CBC1 | 2.09259E-05 | 3.20042572 |
| hsa_circ_0013672_CBC1 | 0.000109704 | 2.781292424 |
| hsa_circ_0126254_CBC1 | 1.02902E-05 | 2.237912848 |
| hsa_circ_0059147_CBC1 | 0.001554805 | 2.753544571 |
| hsa-circRNA1853-5_CBC1 | 1.83945E-05 | 4.311367164 |
| hsa_circ_0070370_CBC1 | 0.002158925 | 2.184812593 |
| hsa-circRNA5800-3_CBC1 | 0.030975233 | 2.360859974 |
| hsa_circ_0110373_CBC1 | 4.27317E-05 | 2.42203143 |
| hsa_circ_0076800_CBC1 | 0.02318922 | 2.986428123 |
| hsa_circ_0033183_CBC1 | 4.48879E-05 | 2.236968144 |
| hsa_circ_0059902_CBC1 | 0.016481406 | 2.160175917 |
| hsa_circ_0126153_CBC1 | 0.027252064 | 2.280386184 |
| hsa_circ_0033180_CBC1 | 0.00105053 | 2.162170584 |
| hsa_circ_0056821_CBC1 | 0.000232569 | 2.068138476 |
| hsa_circ_0089096_CBC1 | 0.045822941 | 2.139245554 |
| hsa_circ_0025573_CBC1 | 0.017748951 | 7.44365715 |
| hsa_circ_0132561_CBC1 | 0.029087855 | 2.023996869 |
| hsa-circRNA5151-37_CBC1 | 0.003227251 | 2.227439624 |
| hsa_circ_0080259_CBC1 | 2.84412E-05 | 2.964138678 |
| hsa-circRNA14135-19_CBC1 | 0.002249278 | 7.261643025 |
| hsa_circ_0043635_CBC1 | 0.026852199 | 2.929110303 |
| hsa_circ_0040197_CBC1 | 1.26152E-05 | 3.029754798 |
| hsa-circRNA14135-6_CBC1 | 0.000261089 | 5.115056106 |
| hsa-circRNA6482-24_CBC1 | 0.005650499 | 4.446736738 |
| hsa_circ_0102211_CBC1 | 0.000993305 | 2.090297395 |
| hsa-circRNA14329-13_CBC1 | 0.011248486 | 2.356601036 |
| hsa-circRNA7418-9_CBC1 | 0.031571777 | 2.109552175 |
| hsa_circ_0074408_CBC1 | 0.013824048 | 2.036088187 |
| hsa_circ_0047277_CBC1 | 0.004202146 | 2.063260318 |
| hsa-circRNA5268-1_CBC1 | 0.004597319 | 2.451263599 |
| hsa-circRNA14135-2_CBC1 | 0.029363986 | 2.10353108 |
| hsa_circ_0074399_CBC1 | 0.017231525 | 2.170992978 |
| hsa_circ_0132579_CBC1 | 0.00862986 | 2.467675374 |
| hsa_circ_0079713_CBC1 | 3.32339E-05 | 3.206320244 |
| hsa_circ_0082693_CBC1 | 0.00929098 | 3.67439405 |
| hsa_circ_0022585_CBC1 | 0.000502909 | 3.7203366 |
| hsa-circRNA5757-10_CBC1 | 0.004845068 | 2.001770187 |
| hsa-circRNA11467-65_CBC1 | 0.026127 | 2.301091381 |
| hsa_circ_0083616_CBC1 | 0.027869628 | 2.364929324 |
| hsa_circ_0051630_CBC1 | 0.000145429 | 2.386950689 |
| hsa-circRNA3815_CBC1 | 4.20994E-05 | 3.163776107 |
| hsa_circ_0122870_CBC1 | 0.00377084 | 2.040708964 |
| hsa_circ_0057969_CBC1 | 0.002176668 | 2.068692499 |
| hsa_circ_0043016_CBC1 | 0.001686854 | 2.137772714 |
| hsa_circ_0079714_CBC1 | 1.73288E-05 | 3.460676125 |
| hsa-circRNA15604-13_CBC1 | 0.001119829 | 2.111625285 |
| hsa_circ_0040183_CBC1 | 8.55456E-05 | 3.003687566 |
| hsa_circ_0138523_CBC1 | 0.000112421 | 13.74153109 |
| hsa_circ_0139340_CBC1 | 0.000547932 | 2.199210121 |
| hsa_circ_0031887_CBC1 | 0.011893289 | 2.124312521 |
| hsa_circ_0008478_CBC1 | 0.001266835 | 2.679430799 |
| hsa-circRNA12476-24_CBC1 | 0.005855496 | 2.133045271 |
| hsa_circ_0020157_CBC1 | 0.000231759 | 2.032902847 |
| hsa_circ_0024328_CBC1 | 0.005442003 | 2.690384249 |
| hsa_circ_0081897_CBC1 | 0.000209 | 2.318520172 |
| hsa_circ_0139812_CBC1 | 0.021714691 | 2.116817829 |
| hsa_circ_0131925_CBC1 | 0.005207682 | 3.07861524 |
| hsa_circ_0040168_CBC1 | 0.000190571 | 3.252118145 |
| hsa_circ_0104946_CBC1 | 0.000906198 | 8.156979531 |
| hsa_circ_0134047_CBC1 | 0.027235411 | 2.075400589 |
| hsa_circ_0087206_CBC1 | 0.006604238 | 5.702724256 |
| hsa_circ_0031894_CBC1 | 0.000580532 | 2.118071088 |
| hsa_circ_0061390_CBC1 | 0.015200839 | 2.013306444 |
| hsa_circ_0117419_CBC1 | 0.021001948 | 2.043365855 |
| hsa_circ_0135689_CBC1 | 0.000673627 | 2.786635055 |
| hsa_circ_0127233_CBC1 | 0.023287868 | 2.351753068 |
| hsa_circ_0139281_CBC1 | 0.00237466 | 2.157679945 |
| hsa_circ_0027412_CBC1 | 0.000180544 | 3.830153383 |
| hsa_circ_0117749_CBC1 | 0.005194968 | 4.363614192 |
| hsa_circ_0068468_CBC1 | 0.006130728 | 2.535989841 |
| hsa_circ_0121731_CBC1 | 0.002334854 | 2.261294519 |
| hsa_circ_0055873_CBC1 | 0.000538224 | 2.275622727 |
| hsa_circ_0079672_CBC1 | 0.000129082 | 2.772747112 |
| hsa_circ_0001618_CBC1 | 0.009077173 | 2.096286791 |
| hsa_circ_0079539_CBC1 | 0.013757427 | 5.027225446 |
| hsa_circ_0025351_CBC1 | 0.000884909 | 2.456941856 |
| hsa-circRNA14989-1_CBC1 | 0.002344462 | 3.03274873 |
| hsa_circ_0090941_CBC1 | 0.00035329 | 2.307764745 |
| hsa_circ_0087269_CBC1 | 0.000382654 | 8.473007514 |
| hsa_circ_0121460_CBC1 | 0.008516999 | 13.73468175 |
| hsa-circRNA482-44_CBC1 | 0.000285608 | 3.674582463 |
| hsa-circRNA14132_CBC1 | 0.00714697 | 2.751468627 |
| hsa_circ_0027977_CBC1 | 0.000574985 | 15.3631077 |
| hsa_circ_0025759_CBC1 | 0.001930785 | 3.856343528 |
| hsa_circ_0054221_CBC1 | 0.038942207 | 4.286348132 |
| hsa_circ_0064043_CBC1 | 0.001820093 | 2.36121154 |
| hsa_circ_0113007_CBC1 | 0.000618776 | 2.037655285 |
| hsa_circ_0040802_CBC1 | 2.44199E-05 | 4.637264356 |
| hsa_circ_0000047_CBC1 | 0.000650526 | 2.067602018 |
| hsa_circ_0079750_CBC1 | 5.45657E-05 | 4.532321297 |
| hsa_circ_0061657_CBC1 | 0.00336419 | 2.676876781 |
| hsa-circRNA8648-5_CBC1 | 0.00032012 | 2.052995315 |
| hsa_circ_0039216_CBC1 | 0.000516661 | 2.250357575 |
| hsa_circ_0039519_CBC1 | 0.000347063 | 3.273699623 |
| hsa-circRNA9643-22_CBC1 | 0.001428881 | 2.469927711 |
| hsa_circ_0022579_CBC1 | 0.000146867 | 5.935657094 |
| hsa_circ_0022591_CBC1 | 0.000432072 | 3.432878302 |
| hsa_circ_0128618_CBC1 | 0.003816042 | 2.303587902 |
| hsa_circ_0025572_CBC1 | 0.001573914 | 3.319892747 |
| hsa_circ_0112004_CBC1 | 7.77448E-05 | 2.466785967 |
| hsa_circ_0133553_CBC1 | 0.003223701 | 3.657921168 |
| hsa-circRNA15292-20_CBC1 | 0.00060804 | 2.025542079 |
| hsa_circ_0124597_CBC1 | 0.00077958 | 2.168987291 |
| hsa_circ_0101495_CBC1 | 0.020060958 | 2.261685572 |
| hsa_circ_0006652_CBC1 | 0.008715459 | 3.457140803 |
| hsa_circ_0108518_CBC1 | 0.026757882 | 2.199071093 |
| hsa_circ_0063455_CBC1 | 0.0180241 | 2.147133964 |
| hsa_circ_0029421_CBC1 | 0.00072651 | 2.259929967 |
| hsa-circRNA4352-4_CBC1 | 0.001891852 | 2.364011116 |
| hsa_circ_0043638_CBC1 | 0.024821184 | 3.356856702 |
| hsa_circ_0114057_CBC1 | 0.027602998 | 2.771842703 |
| hsa_circ_0139347_CBC1 | 0.001684433 | 2.057794362 |
| hsa_circ_0046287_CBC1 | 0.00399135 | 3.136188193 |
| hsa-circRNA11226-16_CBC1 | 0.000156238 | 3.937401539 |
| hsa-circRNA7224-2_CBC1 | 0.001044636 | 3.199037552 |
| hsa_circ_0025108_CBC1 | 0.005694579 | 3.532339714 |
| hsa_circ_0125464_CBC1 | 0.000139512 | 2.897027196 |
| hsa_circ_0093558_CBC1 | 0.00136823 | 3.066993171 |
| hsa_circ_0027259_CBC1 | 0.001745838 | 2.478524775 |
| hsa_circ_0040805_CBC1 | 0.000161354 | 5.824899102 |
| hsa_circ_0091392_CBC1 | 0.005331869 | 2.154739398 |
| hsa_circ_0126765_CBC1 | 0.011500739 | 2.613178965 |
| hsa_circ_0086458_CBC1 | 0.000251685 | 2.735659459 |
| hsa_circ_0025356_CBC1 | 0.002992986 | 2.283068244 |
| hsa_circ_0070368_CBC1 | 0.001872238 | 2.397469239 |
| hsa_circ_0140022_CBC1 | 0.012396216 | 2.087250038 |
| hsa-circRNA7362-10_CBC1 | 0.01326656 | 4.142904816 |
| hsa-circRNA1853-15_CBC1 | 0.000140451 | 3.50510518 |
| hsa_circ_0137865_CBC1 | 0.003140332 | 2.052619009 |
| hsa-circRNA7988-5_CBC1 | 0.046311374 | 3.038262858 |
| hsa_circ_0123890_CBC1 | 0.000324363 | 2.908985699 |
| hsa_circ_0104647_CBC1 | 0.001445917 | 2.152163297 |
| hsa_circ_0073209_CBC1 | 0.007069947 | 3.607274667 |
| hsa_circ_0018267_CBC1 | 0.000327106 | 4.990138645 |
| hsa-circRNA14135-1_CBC1 | 0.000275973 | 9.389864332 |
| hsa-circRNA1826-9_CBC1 | 7.08605E-05 | 2.643738245 |
| hsa_circ_0133862_CBC1 | 0.00954021 | 2.020005293 |
| hsa_circ_0036113_CBC1 | 0.000454143 | 2.087679139 |
| hsa_circ_0131929_CBC1 | 0.002057355 | 2.414124088 |
| hsa_circ_0016480_CBC1 | 7.668E-06 | 2.724311507 |
| hsa_circ_0113010_CBC1 | 0.00131145 | 2.091106331 |
| hsa_circ_0043621_CBC1 | 0.014472135 | 3.521632022 |
| hsa_circ_0040202_CBC1 | 0.000276066 | 3.29163682 |
| hsa_circ_0111630_CBC1 | 0.00124849 | 2.01482092 |
| hsa_circ_0012135_CBC1 | 0.000661894 | 2.019848866 |
| hsa-circRNA6482-28_CBC1 | 0.002331686 | 2.952842718 |
| hsa_circ_0061232_CBC1 | 0.001662624 | 2.189671812 |
| hsa_circ_0082699_CBC1 | 0.00158281 | 3.922268015 |
| hsa-circRNA11158-43_CBC1 | 0.000302832 | 3.061462107 |
| hsa-circRNA1363-19_CBC1 | 0.000484656 | 3.656758417 |
| hsa_circ_0117232_CBC1 | 0.002832785 | 2.212623582 |
| hsa_circ_0091039_CBC1 | 0.001147155 | 2.060427759 |
| hsa_circ_0000829_CBC1 | 0.000680584 | 2.043431805 |
| hsa_circ_0131473_CBC1 | 0.003567192 | 2.126149737 |
| hsa_circ_0133551_CBC1 | 0.000895652 | 3.231405287 |
| hsa_circ_0016602_CBC1 | 0.030668896 | 2.12539687 |
| hsa-circRNA4480-1_CBC1 | 0.002851268 | 2.160455883 |
| hsa_circ_0128072_CBC1 | 0.00130931 | 2.26955563 |
| hsa_circ_0130232_CBC1 | 0.004220679 | 3.268960154 |
| hsa_circ_0129727_CBC1 | 0.00995057 | 2.037444343 |
| hsa_circ_0127230_CBC1 | 0.00253455 | 2.298189922 |
| hsa_circ_0054653_CBC1 | 0.014735101 | 2.530459255 |
| hsa_circ_0025981_CBC1 | 0.01134826 | 3.065887563 |
| hsa_circ_0070969_CBC1 | 0.000148161 | 10.94995528 |
| hsa_circ_0027413_CBC1 | 0.000520749 | 3.710147293 |
| hsa_circ_0007810_CBC1 | 0.001622033 | 2.86886051 |
| hsa_circ_0073130_CBC1 | 0.001989588 | 2.672701154 |
| hsa-circRNA11663-7_CBC1 | 0.020755841 | 2.222474529 |
| hsa_circ_0057707_CBC1 | 0.00206101 | 2.167165677 |
| hsa_circ_0108314_CBC1 | 0.000319139 | 2.023261475 |
| hsa-circRNA15708-1_CBC1 | 0.001928566 | 2.848071779 |
| hsa_circ_0047598_CBC1 | 0.019226352 | 2.139268095 |
| hsa_circ_0118384_CBC1 | 0.009877108 | 2.983887438 |
| hsa_circ_0016977_CBC1 | 0.000286369 | 2.087431029 |
| hsa-circRNA6775-6_CBC1 | 0.0079593 | 2.023062182 |
| hsa_circ_0054328_CBC1 | 0.010375301 | 2.184703201 |
| hsa_circ_0114454_CBC1 | 0.047183206 | 2.36381878 |
| hsa_circ_0120774_CBC1 | 0.00168892 | 2.153614662 |
| hsa_circ_0031882_CBC1 | 0.00288108 | 2.172265785 |
| hsa_circ_0079721_CBC1 | 0.001037186 | 3.17397841 |
| hsa_circ_0002835_CBC1 | 0.000623203 | 2.152603448 |
| hsa-circRNA6775-2_CBC1 | 0.000441255 | 2.242017053 |
| hsa-circRNA12761-6_CBC1 | 0.018056921 | 2.01579405 |
| hsa_circ_0025566_CBC1 | 0.016389965 | 4.519842858 |
| hsa_circ_0124891_CBC1 | 0.001104269 | 2.010358873 |
| hsa_circ_0124567_CBC1 | 0.042365561 | 2.181157922 |
| hsa_circ_0031422_CBC1 | 0.02906772 | 2.035286206 |
| hsa_circ_0130494_CBC1 | 0.004019918 | 2.029706036 |
| hsa-circRNA9873-17_CBC1 | 0.016476199 | 3.821634781 |
| hsa_circ_0130653_CBC1 | 0.00370946 | 2.399621289 |
| hsa_circ_0070972_CBC1 | 0.000562258 | 7.730968815 |
| hsa_circ_0027403_CBC1 | 0.000159087 | 3.683909051 |
| hsa_circ_0112013_CBC1 | 0.000867797 | 2.949388671 |
| hsa-circRNA11592-37_CBC1 | 0.001527224 | 2.375637347 |
| hsa_circ_0129829_CBC1 | 0.000209327 | 3.101802099 |
| hsa_circ_0084888_CBC1 | 0.003184667 | 2.005450415 |
| hsa_circ_0137131_CBC1 | 0.015385457 | 2.250182477 |
| hsa_circ_0070231_CBC1 | 0.015704469 | 2.267306408 |
| hsa-circRNA14441-28_CBC1 | 0.020505766 | 2.070425093 |
| hsa_circ_0140137_CBC1 | 0.039694079 | 2.156865751 |
| hsa_circ_0105918_CBC1 | 0.004065957 | 2.091455834 |
| hsa_circ_0097081_CBC1 | 0.007709359 | 2.028804577 |
| hsa_circ_0022569_CBC1 | 0.020721069 | 2.090724436 |
| hsa_circ_0055266_CBC1 | 0.028355296 | 2.053740513 |
| hsa_circ_0079960_CBC1 | 0.013372869 | 2.202560983 |
| hsa_circ_0139328_CBC1 | 0.003749645 | 2.113667279 |
| hsa_circ_0139337_CBC1 | 0.001272614 | 2.260756435 |
| hsa_circ_0112868_CBC1 | 0.002432078 | 2.031723543 |
| hsa_circ_0045270_CBC1 | 0.006711253 | 2.007528632 |
| hsa-circRNA13091_CBC1 | 0.041062312 | 7.031842403 |
| hsa-circRNA1853-1_CBC1 | 0.000175775 | 3.60602574 |
| hsa_circ_0113000_CBC1 | 0.000981248 | 2.193416715 |
| hsa_circ_0069596_CBC1 | 0.002793066 | 2.010793639 |
| hsa_circ_0094957_CBC1 | 0.017378657 | 2.31739019 |
| hsa_circ_0128861_CBC1 | 0.034919915 | 2.858244707 |
| hsa_circ_0027619_CBC1 | 0.002343254 | 2.405831606 |
| hsa_circ_0040173_CBC1 | 0.00010391 | 3.061518722 |
| hsa_circ_0093325_CBC1 | 0.000995692 | 2.033606 |
| hsa_circ_0125462_CBC1 | 0.000566088 | 2.47496266 |
| hsa_circ_0040191_CBC1 | 0.000133568 | 2.87967011 |
| hsa_circ_0055860_CBC1 | 0.001997673 | 2.18395063 |
| hsa_circ_0087487_CBC1 | 0.004659342 | 2.291424809 |
| hsa_circ_0118387_CBC1 | 0.004993197 | 2.461602875 |
| hsa-circRNA15681-1_CBC1 | 0.027714671 | 2.057102408 |
| hsa_circ_0129826_CBC1 | 0.002392452 | 6.793008785 |
| hsa_circ_0040203_CBC1 | 0.000101852 | 2.757980062 |
| hsa_circ_0084809_CBC1 | 0.000262177 | 2.292132586 |
| hsa-circRNA352-10_CBC1 | 0.012462775 | 2.037623623 |
| hsa-circRNA8953_CBC1 | 0.036556164 | 3.093501544 |
| hsa_circ_0070940_CBC1 | 0.000335082 | 2.175178126 |
| hsa_circ_0121334_CBC1 | 0.004657757 | 2.042876571 |
| hsa_circ_0133544_CBC1 | 0.014851745 | 2.654440739 |
| hsa_circ_0130728_CBC1 | 0.024602402 | 2.388576942 |
| hsa_circ_0108633_CBC1 | 0.011871368 | 2.145907584 |
| hsa_circ_0108853_CBC1 | 0.015133001 | 2.05926403 |
| hsa_circ_0104394_CBC1 | 0.00182517 | 2.14175892 |
| hsa_circ_0005094_CBC1 | 0.045984159 | 3.318582424 |
| hsa_circ_0070973_CBC1 | 0.003421101 | 6.543509836 |
| hsa_circ_0047265_CBC1 | 0.035958679 | 2.109131393 |
| hsa_circ_0070441_CBC1 | 0.009661382 | 2.450659502 |
| hsa_circ_0003438_CBC1 | 2.33268E-05 | 3.025062053 |
| hsa-circRNA1363-15_CBC1 | 0.000410009 | 4.396598861 |
| hsa_circ_0027233_CBC1 | 0.000523346 | 2.340421871 |
| hsa_circ_0074026_CBC1 | 0.022352231 | 7.665344064 |
| hsa_circ_0027268_CBC1 | 0.006482116 | 2.202438098 |
| hsa_circ_0073221_CBC1 | 0.00117325 | 2.741838892 |
| hsa_circ_0003134_CBC1 | 0.017612553 | 2.604613518 |
| hsa_circ_0070976_CBC1 | 0.002550616 | 8.538156714 |
| hsa_circ_0027420_CBC1 | 0.000622444 | 3.304369479 |
| hsa_circ_0011444_CBC1 | 0.000989918 | 2.485303199 |
| hsa_circ_0124388_CBC1 | 0.049673096 | 3.664219866 |
| hsa_circ_0086215_CBC1 | 0.019419298 | 2.465512651 |
| hsa_circ_0072515_CBC1 | 0.014183937 | 2.413967597 |
| hsa_circ_0018045_CBC1 | 3.3186E-05 | 2.246918244 |
| hsa_circ_0112426_CBC1 | 0.001975606 | 2.573170283 |
| hsa_circ_0075618_CBC1 | 0.011456201 | 3.370275773 |
| hsa-circRNA4501-3_CBC1 | 1.49253E-05 | 6.5502991 |
| hsa_circ_0079724_CBC1 | 0.002184672 | 3.188056432 |
| hsa_circ_0112006_CBC1 | 8.03517E-05 | 2.544920895 |
| hsa_circ_0069236_CBC1 | 0.028834243 | 2.158005503 |
| hsa_circ_0111995_CBC1 | 0.0206515 | 2.693097636 |
| hsa-circRNA8991-78_CBC1 | 5.34964E-05 | 2.141396376 |
| hsa_circ_0055282_CBC1 | 0.004163458 | 12.77455319 |
| hsa_circ_0027974_CBC1 | 7.10996E-06 | 8.587166013 |
| hsa_circ_0025441_CBC1 | 0.005379827 | 2.220560643 |
| hsa_circ_0085326_CBC1 | 0.011060623 | 2.123374841 |
| hsa_circ_0072158_CBC1 | 3.93624E-05 | 2.234067187 |
| hsa-circRNA7032-23_CBC1 | 0.000161487 | 2.707437939 |
| hsa_circ_0103923_CBC1 | 0.001913747 | 3.510815495 |
| hsa-circRNA165-1_CBC1 | 0.000763576 | 2.934965373 |
| hsa-circRNA13180_CBC1 | 3.38717E-05 | 3.813580735 |
| hsa_circ_0092634_CBC1 | 0.003361642 | 2.589778348 |
| hsa_circ_0125665_CBC1 | 0.048760638 | 3.97138819 |
| hsa_circ_0070980_CBC1 | 0.000509745 | 10.33713538 |
| hsa_circ_0079728_CBC1 | 0.000623995 | 2.846007619 |
| hsa_circ_0055865_CBC1 | 0.000268959 | 2.449424789 |
| hsa-circRNA3173-4_CBC1 | 0.010940145 | 3.263769721 |
| hsa-circRNA7172-2_CBC1 | 0.007993493 | 2.964988254 |
| hsa_circ_0007199_CBC1 | 0.014080097 | 2.074793391 |
| hsa-circRNA11158-51_CBC1 | 0.001211849 | 2.410853863 |
| hsa_circ_0117949_CBC1 | 0.014644152 | 2.242715681 |
| hsa_circ_0022584_CBC1 | 0.000219568 | 4.714829312 |
| hsa_circ_0072774_CBC1 | 0.001605526 | 2.017456042 |
| hsa_circ_0024329_CBC1 | 0.011854982 | 3.155183798 |
| hsa_circ_0073215_CBC1 | 0.000236923 | 3.134014227 |
| hsa_circ_0002618_CBC1 | 0.017414415 | 2.235734634 |
| hsa_circ_0043619_CBC1 | 0.031700251 | 3.509062574 |
| hsa-circRNA15822-48_CBC1 | 0.009895961 | 2.07070825 |
| hsa_circ_0135685_CBC1 | 2.48423E-05 | 2.594945916 |
| hsa_circ_0070905_CBC1 | 0.038272452 | 4.630059158 |
| hsa_circ_0087467_CBC1 | 0.004361516 | 2.554050609 |
| hsa_circ_0067960_CBC1 | 0.001355271 | 2.418056264 |
| hsa_circ_0119525_CBC1 | 0.004215795 | 2.520257597 |
| hsa_circ_0098129_CBC1 | 0.04051611 | 2.016721694 |
| hsa-circRNA1364-2_CBC1 | 0.012523972 | 2.037338956 |
| hsa_circ_0128617_CBC1 | 0.005719285 | 2.40593335 |
| hsa_circ_0001398_CBC1 | 0.003356615 | 2.002557001 |
| hsa_circ_0030759_CBC1 | 0.004756144 | 2.086421251 |
| hsa_circ_0012009_CBC1 | 0.010372243 | 2.088379768 |
| hsa_circ_0027975_CBC1 | 2.68624E-05 | 7.661133628 |
| hsa_circ_0093831_CBC1 | 0.03036629 | 4.091089157 |
| hsa-circRNA8649-8_CBC1 | 0.029385633 | 2.25094929 |
| hsa_circ_0040799_CBC1 | 8.91266E-05 | 6.373812901 |
| hsa_circ_0087472_CBC1 | 0.003011815 | 2.099109496 |
| hsa_circ_0124858_CBC1 | 0.004891685 | 2.396680571 |
| hsa-circRNA13870-5_CBC1 | 0.005539799 | 2.292220871 |
| hsa_circ_0042106_CBC1 | 0.026848756 | 2.446838701 |
| hsa_circ_0011439_CBC1 | 0.001002347 | 2.224235413 |
| hsa_circ_0030756_CBC1 | 0.010990318 | 2.059781158 |
| hsa_circ_0101549_CBC1 | 0.001578964 | 2.274828662 |
| hsa_circ_0016481_CBC1 | 1.15428E-05 | 2.605155758 |
| hsa-circRNA14285-1_CBC1 | 0.000330778 | 2.160261909 |
| hsa_circ_0117950_CBC1 | 0.025191401 | 2.445920887 |
| hsa_circ_0035436_CBC1 | 0.034985084 | 5.538784093 |
| hsa_circ_0134109_CBC1 | 4.76572E-05 | 3.559014966 |
| hsa_circ_0122325_CBC1 | 0.005433992 | 2.015772671 |
| hsa-circRNA10421-1_CBC1 | 5.02798E-05 | 2.184177959 |
| hsa-circRNA734-4_CBC1 | 0.001472111 | 2.0608586 |
| hsa_circ_0083436_CBC1 | 0.019982882 | 3.285969314 |
| hsa_circ_0034646_CBC1 | 0.001103953 | 3.619305203 |
| hsa-circRNA9900-8_CBC1 | 0.001110059 | 2.166921352 |
| hsa-circRNA3440-13_CBC1 | 0.002475253 | 2.226835208 |
| hsa_circ_0122578_CBC1 | 0.00305495 | 2.131853469 |
| hsa_circ_0084053_CBC1 | 0.025606781 | 2.138275355 |
| hsa_circ_0103919_CBC1 | 0.044082998 | 2.834920724 |
| hsa_circ_0055857_CBC1 | 0.003245495 | 2.519420171 |
| hsa_circ_0137492_CBC1 | 0.001690567 | 2.137301435 |
| hsa_circ_0125024_CBC1 | 0.002593423 | 2.454250869 |
| hsa_circ_0068490_CBC1 | 0.011345988 | 2.078223298 |
| hsa_circ_0122158_CBC1 | 0.004221146 | 2.026048221 |
| hsa-circRNA628-32_CBC1 | 0.031466742 | 2.148673045 |
| hsa_circ_0012971_CBC1 | 0.023531635 | 2.470250161 |
| hsa_circ_0068456_CBC1 | 0.010066307 | 2.758392327 |
| hsa_circ_0008356_CBC1 | 0.006039405 | 2.056791063 |
| hsa-circRNA3626-3_CBC1 | 0.003317825 | 2.160378508 |
| hsa-circRNA14343-12_CBC1 | 0.037704447 | 2.120274011 |
| hsa_circ_0039978_CBC1 | 0.041728949 | 2.278404152 |
| hsa_circ_0024325_CBC1 | 0.007965614 | 2.128202132 |
| hsa_circ_0040157_CBC1 | 5.35956E-05 | 3.293146999 |
| hsa_circ_0136584_CBC1 | 0.008061807 | 2.164623366 |
| hsa_circ_0134152_CBC1 | 0.017806073 | 2.209188392 |
| hsa_circ_0119066_CBC1 | 0.021996499 | 2.173020944 |
| hsa-circRNA1706-10_CBC1 | 0.028613247 | 5.835601581 |
| hsa_circ_0027972_CBC1 | 0.000178236 | 6.225749478 |
| hsa_circ_0118385_CBC1 | 0.006593652 | 2.594253157 |
| hsa_circ_0139984_CBC1 | 0.002307446 | 6.608487855 |
| hsa_circ_0102640_CBC1 | 0.000204487 | 2.111334022 |
| hsa_circ_0040200_CBC1 | 1.69297E-05 | 3.092040504 |
| hsa_circ_0130230_CBC1 | 0.001127545 | 2.101490835 |
| hsa_circ_0063395_CBC1 | 0.000664053 | 2.559149664 |
| hsa_circ_0110374_CBC1 | 2.11061E-05 | 2.288937587 |
| hsa_circ_0114522_CBC1 | 0.008323166 | 3.226816377 |
| hsa_circ_0012131_CBC1 | 0.000100632 | 2.531507921 |
| hsa_circ_0118253_CBC1 | 0.027485884 | 5.715808216 |
| hsa_circ_0078631_CBC1 | 0.043130613 | 2.084630065 |
| hsa_circ_0121509_CBC1 | 0.0105927 | 2.208105695 |
| hsa-circRNA15785-1_CBC1 | 7.08935E-05 | 3.989522949 |
| hsa_circ_0006268_CBC1 | 0.003528886 | 4.043452177 |
| hsa_circ_0068467_CBC1 | 0.00064727 | 2.560236316 |
| hsa_circ_0025979_CBC1 | 0.003624528 | 2.340768412 |
| hsa_circ_0001158_CBC1 | 0.015536094 | 2.099200038 |
| hsa_circ_0006677_CBC1 | 0.001111465 | 2.315228455 |
| hsa_circ_0011890_CBC1 | 0.00521045 | 2.262127159 |
| hsa_circ_0025418_CBC1 | 0.000135019 | 2.072390742 |
| hsa_circ_0043644_CBC1 | 0.036312274 | 2.29905531 |
| hsa-circRNA11819-15_CBC1 | 0.020109011 | 2.183456868 |
| hsa_circ_0073531_CBC1 | 0.000945512 | 2.274921185 |
| hsa_circ_0057167_CBC1 | 2.72491E-05 | 2.092386463 |
| hsa_circ_0027404_CBC1 | 0.000860457 | 3.459992665 |
| hsa-circRNA14469-26_CBC1 | 0.029647796 | 2.681750137 |
| hsa_circ_0077758_CBC1 | 0.034177654 | 2.35969156 |
| hsa_circ_0032038_CBC1 | 0.002008834 | 2.031009812 |
| hsa_circ_0047273_CBC1 | 0.012725471 | 2.117248096 |
| hsa-circRNA13470-5_CBC1 | 0.000104901 | 2.111120173 |
| hsa-circRNA15822-29_CBC1 | 0.000479564 | 2.202330512 |
| hsa-circRNA6610-9_CBC1 | 0.002699974 | 3.351796827 |
| hsa_circ_0136534_CBC1 | 0.000451361 | 2.117370011 |
| hsa_circ_0124470_CBC1 | 0.003079572 | 4.884329923 |
| hsa_circ_0087506_CBC1 | 0.002493585 | 2.441866547 |
| hsa-circRNA6988-70_CBC1 | 0.007782927 | 2.035967363 |
| hsa_circ_0045709_CBC1 | 0.002821778 | 2.030112291 |
| hsa-circRNA1506-38_CBC1 | 0.00160619 | 2.727727322 |
| hsa_circ_0012947_CBC1 | 0.042872025 | 2.258613512 |
| hsa-circRNA11158-56_CBC1 | 7.89308E-05 | 2.793490999 |
| hsa_circ_0016923_CBC1 | 0.011562308 | 2.178006709 |
| hsa_circ_0041339_CBC1 | 0.017485937 | 3.488337739 |
| hsa-circRNA7032-9_CBC1 | 1.94163E-05 | 3.566155229 |
| hsa_circ_0054651_CBC1 | 0.018884493 | 2.591054179 |
| hsa_circ_0089092_CBC1 | 0.014917361 | 2.775916029 |
| hsa_circ_0071460_CBC1 | 0.029482549 | 2.650431596 |
| hsa_circ_0125662_CBC1 | 0.000314477 | 2.881286136 |
| hsa_circ_0096080_CBC1 | 0.017035321 | 2.447174223 |
| hsa_circ_0081896_CBC1 | 0.000776274 | 2.121236248 |
| hsa_circ_0054590_CBC1 | 0.011805575 | 2.003098939 |
| hsa-circRNA11158-53_CBC1 | 2.74293E-06 | 3.102212821 |
| hsa_circ_0073212_CBC1 | 0.001893748 | 2.816971154 |
| hsa-circRNA7514-2_CBC1 | 0.009111025 | 2.12104029 |
| hsa_circ_0086476_CBC1 | 0.010885535 | 2.681963304 |
| hsa-circRNA9533-27_CBC1 | 0.005004776 | 2.064699647 |
| hsa_circ_0110372_CBC1 | 0.00073097 | 2.164225635 |
| hsa-circRNA12412-175_CBC1 | 0.001432296 | 2.198430139 |
| hsa_circ_0027273_CBC1 | 0.00061745 | 2.042338297 |
| hsa_circ_0054968_CBC1 | 0.002363649 | 3.783944353 |
| hsa-circRNA14765_CBC1 | 0.000384591 | 2.103341544 |
| hsa_circ_0040188_CBC1 | 9.43732E-05 | 3.190004683 |
| hsa_circ_0112997_CBC1 | 0.007775677 | 2.741268876 |
| hsa_circ_0077868_CBC1 | 0.027905589 | 2.245267747 |
| hsa_circ_0072108_CBC1 | 0.017111815 | 2.030645324 |
| hsa_circ_0027399_CBC1 | 0.000196749 | 3.069852779 |
| hsa-circRNA4501-7_CBC1 | 3.63103E-05 | 6.253959413 |
| hsa_circ_0107170_CBC1 | 0.040588337 | 2.135752111 |
| hsa_circ_0004417_CBC1 | 0.001084314 | 2.723757118 |
| hsa_circ_0112014_CBC1 | 0.003704976 | 3.075232152 |
| hsa-circRNA10095_CBC1 | 0.000153484 | 6.866946111 |
| hsa_circ_0122155_CBC1 | 0.008887293 | 2.120696036 |
| hsa_circ_0034414_CBC1 | 0.021030543 | 4.587771781 |
| hsa-circRNA1706-30_CBC1 | 0.026125197 | 3.769707784 |
| hsa-circRNA8991-87_CBC1 | 4.07179E-05 | 2.167999306 |
| hsa_circ_0127213_CBC1 | 0.002384328 | 2.380647687 |
| hsa_circ_0051622_CBC1 | 0.00158694 | 2.243935398 |
| hsa_circ_0137749_CBC1 | 0.007045031 | 2.187499637 |
| hsa_circ_0089982_CBC1 | 0.016702339 | 2.393534992 |
| hsa-circRNA16053-1_CBC1 | 0.004155571 | 3.732956358 |
| hsa-circRNA3634-5_CBC1 | 0.039685302 | 2.121473508 |
| hsa_circ_0062722_CBC1 | 0.002169438 | 2.160156965 |
| hsa_circ_0051637_CBC1 | 0.000117107 | 2.228649876 |
| hsa_circ_0004689_CBC1 | 0.03852396 | 2.073867676 |
| hsa-circRNA15082-12_CBC1 | 2.75389E-05 | 4.376413859 |
| hsa_circ_0095239_CBC1 | 0.002516447 | 2.189657239 |
| hsa_circ_0127508_CBC1 | 0.007545005 | 2.044332055 |
| hsa_circ_0076798_CBC1 | 0.008033913 | 2.038028521 |
| hsa-circRNA15292-8_CBC1 | 0.000146315 | 2.095074739 |
| hsa_circ_0101547_CBC1 | 0.000336102 | 2.113474832 |
| hsa_circ_0022590_CBC1 | 0.000464785 | 3.631008551 |
| hsa-circRNA9873-23_CBC1 | 0.000540018 | 3.089458032 |
| hsa_circ_0044784_CBC1 | 0.007758514 | 3.353127719 |
| hsa-circRNA4561-9_CBC1 | 0.002275473 | 2.189272524 |
| hsa_circ_0091054_CBC1 | 0.020040664 | 2.041847482 |
| hsa_circ_0124380_CBC1 | 0.027644649 | 2.281537771 |
| hsa_circ_0014209_CBC1 | 0.005701925 | 3.536497384 |
| hsa-circRNA14960-19_CBC1 | 0.000235775 | 2.292012718 |
| hsa_circ_0003903_CBC1 | 0.031443344 | 2.020610657 |
| hsa-circRNA4501-10_CBC1 | 8.2689E-05 | 4.914579715 |
| hsa_circ_0072110_CBC1 | 0.008569584 | 2.406321127 |
| hsa_circ_0127979_CBC1 | 0.02819198 | 2.010737628 |
| hsa_circ_0108109_CBC1 | 0.010368182 | 2.085046164 |
| hsa_circ_0009000_CBC1 | 0.000454781 | 2.475392632 |
| hsa_circ_0027231_CBC1 | 0.000779774 | 2.137291098 |
| hsa_circ_0079710_CBC1 | 0.001754009 | 3.311971513 |
| hsa_circ_0114330_CBC1 | 0.002211078 | 2.097454327 |
| hsa_circ_0139578_CBC1 | 0.040923825 | 2.170729999 |
| hsa_circ_0115035_CBC1 | 0.011126695 | 2.242906651 |
| hsa-circRNA10054-2_CBC1 | 0.003338938 | 4.461524893 |
| hsa_circ_0079536_CBC1 | 0.001434319 | 2.630528541 |
| hsa_circ_0008221_CBC1 | 0.003663365 | 2.083174326 |
| hsa-circRNA9873-13_CBC1 | 0.001503643 | 3.307205567 |
| hsa_circ_0092636_CBC1 | 0.018284972 | 3.469331985 |
| hsa_circ_0070974_CBC1 | 0.004748896 | 7.998350705 |
| hsa_circ_0038428_CBC1 | 0.035866425 | 2.561816685 |
| hsa-circRNA7503-16_CBC1 | 0.009411726 | 2.022792332 |
| hsa_circ_0119284_CBC1 | 0.034722482 | 2.089858598 |
| hsa-circRNA9873-9_CBC1 | 0.048738159 | 5.06156266 |
| hsa-circRNA14293-13_CBC1 | 0.005702324 | 2.112405582 |
| hsa_circ_0073206_CBC1 | 0.015191475 | 2.735507863 |
| hsa_circ_0117751_CBC1 | 0.002218894 | 6.447105388 |
| hsa-circRNA2247-6_CBC1 | 0.009997578 | 2.256369596 |
| hsa-circRNA14135-16_CBC1 | 0.002430315 | 7.202331932 |
| hsa-circRNA14785-3_CBC1 | 0.032577673 | 2.433919768 |
| hsa_circ_0080946_CBC1 | 0.00935608 | 2.185966139 |
| hsa_circ_0076608_CBC1 | 0.002659447 | 2.45774122 |
| hsa-circRNA10585-11_CBC1 | 0.003267115 | 2.528975376 |
| hsa_circ_0021155_CBC1 | 0.000167549 | 2.161824537 |
| hsa_circ_0098319_CBC1 | 0.008497287 | 4.39183388 |
| hsa_circ_0087105_CBC1 | 0.035891455 | 2.596753099 |
| hsa_circ_0069339_CBC1 | 0.006895161 | 2.18636458 |
| hsa-circRNA9834-43_CBC1 | 0.003640414 | 2.151176175 |
| hsa-circRNA3631-18_CBC1 | 0.006235363 | 2.552568776 |
| hsa_circ_0025569_CBC1 | 0.040905074 | 4.333046492 |
| hsa-circRNA4561-28_CBC1 | 0.003104556 | 2.526535946 |
| hsa_circ_0055866_CBC1 | 0.002670679 | 2.281986139 |
| hsa_circ_0123459_CBC1 | 0.04492795 | 2.226249913 |
| hsa_circ_0070981_CBC1 | 0.001423894 | 8.59184554 |
| hsa_circ_0005304_CBC1 | 0.000357458 | 2.496409896 |
| hsa_circ_0140214_CBC1 | 0.001735803 | 4.688052723 |
| hsa_circ_0024151_CBC1 | 0.004665552 | 2.816928676 |
| hsa_circ_0114680_CBC1 | 0.001208484 | 2.631741139 |
| hsa_circ_0128097_CBC1 | 0.010140691 | 2.274371946 |
| hsa-circRNA2713-39_CBC1 | 0.048870884 | 2.095270471 |
| hsa-circRNA6235-11_CBC1 | 0.01187361 | 2.051095119 |
| hsa_circ_0074374_CBC1 | 0.00316431 | 2.166885522 |
| hsa_circ_0115571_CBC1 | 0.004165467 | 3.032410896 |
| hsa_circ_0007733_CBC1 | 0.011050271 | 2.287506584 |
| hsa_circ_0037209_CBC1 | 0.023696283 | 2.040657292 |
| hsa_circ_0127099_CBC1 | 0.010311439 | 2.378046667 |
| hsa_circ_0002503_CBC1 | 0.002916137 | 2.28421872 |
| hsa_circ_0040164_CBC1 | 2.1489E-06 | 3.341780271 |
| hsa_circ_0100615_CBC1 | 0.003990427 | 3.193626456 |
| hsa_circ_0016483_CBC1 | 0.002884539 | 2.272457921 |
| hsa-circRNA9643-9_CBC1 | 0.034097146 | 2.332900463 |
| hsa_circ_0112566_CBC1 | 0.001666393 | 2.234721652 |
| hsa_circ_0106181_CBC1 | 0.00024855 | 2.387207877 |
| hsa-circRNA9830-1_CBC1 | 0.015257757 | 2.866515277 |
| hsa_circ_0134743_CBC1 | 0.009954474 | 2.669749782 |
| hsa_circ_0134117_CBC1 | 0.001038704 | 4.340528676 |
| hsa-circRNA7337_CBC1 | 0.000581346 | 2.173484089 |
| hsa_circ_0101925_CBC1 | 0.017159476 | 2.204417099 |
| hsa_circ_0081989_CBC1 | 1.86859E-05 | 2.830908855 |
| hsa_circ_0083432_CBC1 | 0.023570233 | 4.314896261 |
| hsa_circ_0051631_CBC1 | 0.000135795 | 2.21520182 |
| hsa_circ_0079752_CBC1 | 0.00598714 | 5.008554635 |
| hsa_circ_0025432_CBC1 | 0.00549869 | 2.301043389 |
| hsa_circ_0081195_CBC1 | 1.15619E-05 | 13.04164134 |
| hsa_circ_0121458_CBC1 | 0.003244335 | 7.469455458 |
| hsa_circ_0054974_CBC1 | 0.002050119 | 2.105934227 |
| hsa_circ_0035614_CBC1 | 0.001375409 | 2.292247627 |
| hsa_circ_0087468_CBC1 | 0.006290943 | 2.135245702 |
| hsa_circ_0103657_CBC1 | 0.032926264 | 2.268905248 |
| hsa_circ_0085322_CBC1 | 0.013695885 | 2.205958043 |
| hsa_circ_0003971_CBC1 | 0.018746265 | 2.051582148 |
| hsa_circ_0027976_CBC1 | 0.000969648 | 5.036601482 |
| hsa_circ_0079722_CBC1 | 4.19657E-05 | 3.162752445 |
| hsa_circ_0087476_CBC1 | 0.001619503 | 2.063217498 |
| hsa_circ_0066929_CBC1 | 0.010890705 | 2.697652211 |
| hsa_circ_0134894_CBC1 | 0.018388 | 5.041625717 |
| hsa_circ_0054970_CBC1 | 5.50503E-05 | 2.305765456 |
| hsa_circ_0040190_CBC1 | 5.98654E-05 | 3.195129784 |
| hsa_circ_0079670_CBC1 | 0.000563225 | 2.117306349 |
| hsa-circRNA3645-4_CBC1 | 0.003396545 | 2.29306808 |
| hsa_circ_0094299_CBC1 | 0.034589503 | 2.006828178 |
| hsa_circ_0094611_CBC1 | 0.020240061 | 2.994314467 |
| hsa-circRNA14441-23_CBC1 | 0.020055053 | 2.156313037 |
| hsa_circ_0034416_CBC1 | 0.006827321 | 2.957521576 |
| hsa_circ_0013414_CBC1 | 0.000322467 | 2.420474673 |
| hsa-circRNA1364-3_CBC1 | 0.006375194 | 2.148698865 |
| hsa_circ_0087499_CBC1 | 0.000205135 | 2.040380356 |
| hsa_circ_0054193_CBC1 | 0.002454191 | 2.151262107 |
| hsa-circRNA5151-7_CBC1 | 0.027514482 | 2.276749276 |
| hsa_circ_0041514_CBC1 | 0.026007886 | 2.371025242 |
| hsa-circRNA4961-2_CBC1 | 0.024426503 | 2.012553786 |
| hsa_circ_0049526_CBC1 | 0.035141038 | 2.164351476 |
| hsa_circ_0081899_CBC1 | 0.018098166 | 2.200317377 |
| hsa_circ_0064338_CBC1 | 0.000652322 | 2.265556277 |
| hsa_circ_0034100_CBC1 | 0.019010417 | 2.178012735 |
| hsa_circ_0080794_CBC1 | 0.000437835 | 3.199739346 |
| hsa_circ_0125312_CBC1 | 0.000707118 | 2.254955357 |
| hsa-circRNA8615-2_CBC1 | 0.004789861 | 2.361807376 |
| hsa_circ_0074369_CBC1 | 0.000111098 | 3.217691817 |
| hsa_circ_0096894_CBC1 | 0.036776112 | 4.263576543 |
| hsa-circRNA5612-4_CBC1 | 0.000404654 | 2.024254099 |
| hsa-circRNA968-1_CBC1 | 0.000196748 | 10.39060354 |
| hsa_circ_0013669_CBC1 | 0.013395701 | 2.295638925 |
| hsa_circ_0079727_CBC1 | 0.002518835 | 2.809360651 |
| hsa_circ_0126107_CBC1 | 0.001403539 | 2.474164847 |
| hsa_circ_0094788_CBC1 | 0.022544394 | 2.482461192 |
| hsa_circ_0056921_CBC1 | 0.016343049 | 2.45352337 |
| hsa_circ_0025563_CBC1 | 0.015115628 | 3.156935076 |
| hsa_circ_0055062_CBC1 | 1.75671E-05 | 2.178461595 |
| hsa_circ_0072511_CBC1 | 0.004554558 | 2.113063155 |
| hsa_circ_0043394_CBC1 | 0.011624623 | 2.096088175 |
| hsa-circRNA11158-17_CBC1 | 0.000958973 | 3.160740358 |
| hsa_circ_0019063_CBC1 | 0.015468126 | 3.567459506 |
| hsa-circRNA1706-9_CBC1 | 0.013710432 | 6.627064601 |
| hsa_circ_0108815_CBC1 | 0.000944802 | 2.025018389 |
| hsa_circ_0087040_CBC1 | 0.004015511 | 2.339168508 |
| hsa_circ_0130271_CBC1 | 0.000297235 | 5.013956385 |
| hsa_circ_0021977_CBC1 | 0.004543795 | 3.314266022 |
| hsa_circ_0053365_CBC1 | 0.004424021 | 2.366939527 |
| hsa-circRNA11226-5_CBC1 | 0.000212036 | 6.980621297 |
| hsa_circ_0040169_CBC1 | 0.000104538 | 2.949124353 |
| hsa_circ_0079725_CBC1 | 0.002769737 | 3.045223707 |
| hsa_circ_0093374_CBC1 | 0.004423705 | 5.96509019 |
| hsa_circ_0129358_CBC1 | 0.049754552 | 2.184000177 |
| hsa_circ_0079723_CBC1 | 0.001764642 | 2.503547242 |
| hsa_circ_0084518_CBC1 | 0.002125248 | 3.697444648 |
| hsa-circRNA6532-14_CBC1 | 0.015664631 | 2.235604198 |
| hsa_circ_0016488_CBC1 | 0.000242125 | 2.664447866 |
| hsa_circ_0133080_CBC1 | 0.000637771 | 2.703137976 |
| hsa_circ_0023809_CBC1 | 2.38837E-05 | 2.35249646 |
| hsa-circRNA1706-21_CBC1 | 0.018659225 | 5.454727275 |
| hsa-circRNA14053-4_CBC1 | 0.000805702 | 2.29047544 |
| hsa_circ_0076883_CBC1 | 0.005664267 | 2.09135592 |
| hsa_circ_0046325_CBC1 | 0.003215522 | 2.263145413 |
| hsa_circ_0083434_CBC1 | 0.021842535 | 3.25319497 |
| hsa_circ_0072559_CBC1 | 0.009643873 | 2.732234232 |
| hsa_circ_0011157_CBC1 | 0.011233238 | 2.59087544 |
| hsa_circ_0110184_CBC1 | 0.00157012 | 2.474579985 |
| hsa_circ_0011442_CBC1 | 0.001449299 | 2.255241416 |
| hsa_circ_0028008_CBC1 | 0.009805733 | 2.288597616 |
| hsa_circ_0047918_CBC1 | 0.004629636 | 2.060115861 |
| hsa_circ_0027789_CBC1 | 0.041110042 | 2.112622098 |
| hsa_circ_0047259_CBC1 | 0.002247618 | 2.112256248 |
| hsa_circ_0098631_CBC1 | 0.000564592 | 2.298068586 |
| hsa_circ_0107905_CBC1 | 0.022120483 | 2.149070506 |
| hsa_circ_0095996_CBC1 | 0.000732662 | 2.439875348 |
| hsa_circ_0113068_CBC1 | 0.000530269 | 2.156121689 |
| hsa_circ_0079753_CBC1 | 9.31355E-06 | 4.773111157 |
| hsa_circ_0012500_CBC1 | 0.011380137 | 2.172418845 |
| hsa-circRNA7850-5_CBC1 | 0.041670033 | 2.216203575 |
| hsa_circ_0114829_CBC1 | 0.004763034 | 2.298536975 |
| hsa_circ_0027230_CBC1 | 0.001966648 | 2.238690175 |
| hsa_circ_0069767_CBC1 | 0.002792844 | 2.654619539 |
| hsa_circ_0116883_CBC1 | 0.015259503 | 2.040811251 |
| hsa_circ_0140211_CBC1 | 0.001614532 | 5.80198901 |
| hsa_circ_0123493_CBC1 | 0.000987721 | 2.295222483 |
| hsa_circ_0040198_CBC1 | 9.56154E-05 | 2.812337948 |
| hsa_circ_0033954_CBC1 | 0.004313876 | 8.37860294 |
| hsa-circRNA6828-2_CBC1 | 0.001386459 | 2.049164525 |
| hsa_circ_0118249_CBC1 | 0.000126333 | 2.532134322 |
| hsa_circ_0027622_CBC1 | 0.005106661 | 2.309776698 |
| hsa_circ_0128865_CBC1 | 0.014531258 | 2.014845906 |
| hsa-circRNA14293-5_CBC1 | 0.001657977 | 2.142623465 |
| hsa-circRNA1950-22_CBC1 | 0.003939202 | 2.217675153 |
| hsa-circRNA7418-5_CBC1 | 0.016154537 | 2.349720121 |
| hsa_circ_0040182_CBC1 | 0.000242309 | 3.172456945 |
| hsa_circ_0108315_CBC1 | 0.001610047 | 2.450462167 |
| hsa_circ_0111847_CBC1 | 0.016880251 | 2.04028677 |
| hsa_circ_0081898_CBC1 | 0.005689229 | 2.49298534 |
| hsa_circ_0116114_CBC1 | 0.004655616 | 2.011128429 |
| hsa_circ_0102933_CBC1 | 0.00859539 | 2.166441929 |
| hsa_circ_0055284_CBC1 | 0.000145568 | 4.884647444 |
| hsa_circ_0122375_CBC1 | 0.007603719 | 2.262110812 |
| hsa_circ_0136718_CBC1 | 0.042873439 | 2.298620969 |
| hsa_circ_0006182_CBC1 | 0.002952665 | 2.207994699 |
| hsa_circ_0100593_CBC1 | 0.014890131 | 2.763302024 |
| hsa_circ_0027414_CBC1 | 9.2206E-05 | 6.989293463 |
| hsa-circRNA8991-30_CBC1 | 0.002257695 | 2.375258799 |
| hsa_circ_0013412_CBC1 | 0.003748784 | 2.258743285 |
| hsa_circ_0114889_CBC1 | 0.002633193 | 2.275719602 |
| hsa-circRNA7032-6_CBC1 | 0.004486404 | 2.561376644 |
| hsa_circ_0054220_CBC1 | 0.00371564 | 5.210918047 |
| hsa_circ_0022580_CBC1 | 7.89824E-05 | 7.446642756 |
| hsa-circRNA10658-1_CBC1 | 0.018159657 | 3.018963377 |
| hsa_circ_0011129_CBC1 | 0.001593815 | 2.693556584 |
| hsa-circRNA14816-15_CBC1 | 0.013821132 | 2.037167341 |
| hsa_circ_0012133_CBC1 | 0.001670794 | 3.280122812 |
| hsa_circ_0079675_CBC1 | 0.003524481 | 2.16286945 |
| hsa_circ_0032841_CBC1 | 0.000115246 | 2.059762638 |
| hsa_circ_0132845_CBC1 | 0.027449722 | 2.12290852 |
| hsa_circ_0126745_CBC1 | 0.011621861 | 3.344176811 |
| hsa_circ_0027345_CBC1 | 0.012593428 | 2.015027091 |
| hsa_circ_0012779_CBC1 | 0.027784038 | 2.075178317 |
| hsa_circ_0078630_CBC1 | 0.002362851 | 2.698144437 |
| hsa_circ_0121574_CBC1 | 0.024122581 | 2.223910772 |
| hsa_circ_0078656_CBC1 | 0.021647993 | 2.135475008 |
| hsa_circ_0078652_CBC1 | 0.000501299 | 2.47870847 |
| hsa_circ_0091374_CBC1 | 0.000127762 | 2.059668191 |
| hsa-circRNA14119-10_CBC1 | 0.00720289 | 2.232030783 |
| hsa_circ_0139934_CBC1 | 0.001389938 | 2.039687958 |
| hsa-circRNA8578-2_CBC1 | 0.002007361 | 2.579617457 |
| hsa_circ_0128196_CBC1 | 0.000209765 | 3.231830244 |
| hsa-circRNA10585-16_CBC1 | 0.003413793 | 2.439972245 |
| hsa_circ_0084139_CBC1 | 0.003065733 | 2.854633374 |
| hsa-circRNA345-8_CBC1 | 0.004669899 | 2.85113781 |
| hsa-circRNA10096-4_CBC1 | 0.000111048 | 8.028536842 |
| hsa_circ_0111622_CBC1 | 0.027000923 | 2.296083234 |
| hsa_circ_0035384_CBC1 | 3.24225E-05 | 3.636626145 |
| hsa_circ_0003436_CBC1 | 0.00153511 | 2.155779831 |
| hsa-circRNA4561-20_CBC1 | 0.004995164 | 2.422784147 |
| hsa-circRNA12514-8_CBC1 | 0.000129908 | 2.050932672 |
| hsa_circ_0113072_CBC1 | 0.001465826 | 2.586907649 |
| hsa_circ_0126174_CBC1 | 0.011795313 | 2.064184162 |
| hsa-circRNA2380-3_CBC1 | 0.00114061 | 2.153178401 |
| hsa_circ_0112616_CBC1 | 0.022317938 | 2.038673954 |
| hsa_circ_0050104_CBC1 | 0.005622822 | 2.776558098 |
| hsa-circRNA7003-11_CBC1 | 0.018360214 | 4.360335889 |
| hsa_circ_0005935_CBC1 | 7.12676E-05 | 2.152924707 |
| hsa-circRNA6482-16_CBC1 | 0.00540736 | 2.184157476 |
| hsa_circ_0040171_CBC1 | 0.000376034 | 3.2219036 |
| hsa_circ_0042127_CBC1 | 0.001632402 | 2.349748889 |
| hsa_circ_0126259_CBC1 | 0.000191555 | 2.02290998 |
| hsa_circ_0068355_CBC1 | 0.009736769 | 2.262420096 |
| hsa_circ_0098180_CBC1 | 0.009880723 | 2.066664196 |
| hsa_circ_0055922_CBC1 | 0.007798337 | 2.060418637 |
| hsa_circ_0030321_CBC1 | 0.000346165 | 2.109021258 |
| hsa_circ_0071469_CBC1 | 0.007357129 | 2.302799739 |
| hsa_circ_0038529_CBC1 | 0.049226276 | 3.043200338 |
| hsa_circ_0123015_CBC1 | 0.043595517 | 2.484591898 |
| hsa_circ_0129833_CBC1 | 0.004727482 | 2.821986191 |
| hsa_circ_0087481_CBC1 | 0.002211792 | 2.330998425 |
| hsa-circRNA1363-26_CBC1 | 0.000431602 | 3.430464276 |
| hsa_circ_0115634_CBC1 | 0.0003342 | 2.742517597 |
| hsa_circ_0040803_CBC1 | 6.82435E-06 | 5.037658177 |
| hsa-circRNA10254-1_CBC1 | 0.000717881 | 2.34155489 |
| hsa_circ_0133547_CBC1 | 0.041062821 | 2.502839692 |
| hsa_circ_0121494_CBC1 | 0.001489918 | 2.098718788 |
| hsa-circRNA1826-2_CBC1 | 0.000113978 | 2.319941254 |
| hsa-circRNA14060_CBC1 | 0.002098008 | 2.273616755 |
| hsa_circ_0112010_CBC1 | 0.000257911 | 2.795129126 |
| hsa_circ_0121950_CBC1 | 0.000951707 | 2.151829616 |
| hsa_circ_0125308_CBC1 | 4.2365E-05 | 2.346906165 |
| hsa-circRNA14076-8_CBC1 | 0.014707355 | 2.058785024 |
| hsa_circ_0017964_CBC1 | 0.013628007 | 2.19345107 |
| hsa-circRNA9077-9_CBC1 | 0.010257605 | 2.021797519 |
| hsa_circ_0084812_CBC1 | 0.006127425 | 2.607130907 |
| hsa-circRNA673-10_CBC1 | 0.014623286 | 2.295532628 |
| hsa_circ_0030752_CBC1 | 0.042391108 | 2.405476547 |
| hsa_circ_0137186_CBC1 | 0.000534387 | 2.70471223 |
| hsa_circ_0006348_CBC1 | 0.000489164 | 2.183650289 |
| hsa_circ_0030030_CBC1 | 0.00019082 | 2.022202077 |
| hsa-circRNA13506-4_CBC1 | 0.001183068 | 2.808306726 |
| hsa_circ_0104949_CBC1 | 0.001205352 | 2.064222183 |
| hsa_circ_0131480_CBC1 | 0.020787949 | 2.261368928 |
| hsa_circ_0099730_CBC1 | 0.002726558 | 2.327166144 |
| hsa_circ_0045275_CBC1 | 0.00259592 | 2.735432508 |
| hsa_circ_0136723_CBC1 | 0.01833021 | 2.288990389 |
| hsa-circRNA1826-8_CBC1 | 0.000702114 | 2.530758996 |
| hsa_circ_0140431_CBC1 | 0.000222209 | 2.023248238 |
| hsa_circ_0031885_CBC1 | 0.005849834 | 2.145350508 |
| hsa_circ_0077878_CBC1 | 0.024376989 | 2.34260243 |
| hsa_circ_0087272_CBC1 | 5.52216E-05 | 11.58666427 |
| hsa_circ_0031317_CBC1 | 0.000449067 | 2.352583112 |
| hsa-circRNA12990-24_CBC1 | 0.010216128 | 3.309432793 |
| hsa-circRNA3302-7_CBC1 | 0.001905848 | 2.307289723 |
| hsa_circ_0067569_CBC1 | 0.004707874 | 2.08806442 |
| hsa-circRNA15343-10_CBC1 | 0.00425082 | 2.012873988 |
| hsa_circ_0084138_CBC1 | 0.006794728 | 2.000577971 |
| hsa_circ_0015994_CBC1 | 0.012813225 | 3.055499377 |
| hsa_circ_0026913_CBC1 | 0.000945522 | 2.306444887 |
| hsa-circRNA10585-18_CBC1 | 0.000688631 | 2.57485787 |
| hsa_circ_0092434_CBC1 | 0.024241559 | 2.016854139 |
| hsa_circ_0099798_CBC1 | 0.034341298 | 2.051967452 |
| hsa-circRNA4561-2_CBC1 | 0.000516623 | 3.256719483 |
| hsa_circ_0112839_CBC1 | 0.028054874 | 2.26435635 |
| hsa-circRNA8991-22_CBC1 | 0.000907926 | 2.276432963 |
| hsa_circ_0073131_CBC1 | 0.002361612 | 2.907659444 |
| hsa-circRNA9533-11_CBC1 | 0.004510191 | 2.139358322 |
| hsa-circRNA2988-12_CBC1 | 0.002920149 | 2.255494107 |
| hsa_circ_0130070_CBC1 | 0.006703205 | 2.714148175 |
| hsa_circ_0087274_CBC1 | 0.000158275 | 10.08198464 |
| hsa_circ_0081990_CBC1 | 0.009257708 | 2.832339578 |
| hsa_circ_0016936_CBC1 | 0.000221029 | 2.119800343 |
| hsa_circ_0140209_CBC1 | 0.010957593 | 3.456238883 |
| hsa_circ_0134118_CBC1 | 4.77435E-05 | 5.066242197 |
| hsa-circRNA3482-3_CBC1 | 0.005112412 | 2.256714135 |
| hsa_circ_0034189_CBC1 | 0.010783881 | 2.43309809 |
| hsa_circ_0124377_CBC1 | 0.000296222 | 3.105240924 |
| hsa_circ_0006957_CBC1 | 5.09189E-05 | 2.515331321 |
| hsa-circRNA660-2_CBC1 | 0.001184941 | 2.903934704 |
| hsa_circ_0139709_CBC1 | 0.021967208 | 2.095645893 |
| hsa_circ_0082072_CBC1 | 0.001359156 | 2.180543863 |
| hsa_circ_0045347_CBC1 | 0.033112452 | 2.380816029 |
| hsa-circRNA5131-3_CBC1 | 0.016411528 | 2.791047197 |
| hsa_circ_0104398_CBC1 | 0.009288055 | 2.044413856 |
| hsa_circ_0013359_CBC1 | 0.049596536 | 2.721103289 |
| hsa_circ_0086452_CBC1 | 0.000745923 | 2.49190654 |
| hsa-circRNA14898-2_CBC1 | 0.006577593 | 2.511048711 |
| hsa_circ_0054654_CBC1 | 0.007470918 | 2.079351672 |
| hsa_circ_0000541_CBC1 | 0.003356682 | 2.560795761 |
| hsa_circ_0105646_CBC1 | 3.10904E-05 | 2.652630163 |
| hsa-circRNA8036-2_CBC1 | 0.001148217 | 2.321809859 |
| hsa-circRNA5027-2_CBC1 | 0.001109996 | 2.240053549 |
| hsa_circ_0000714_CBC1 | 9.12075E-05 | 2.510295141 |
| hsa_circ_0130767_CBC1 | 0.022057586 | 2.031043633 |
| hsa_circ_0083915_CBC1 | 0.008895283 | 2.108402554 |
| hsa_circ_0123456_CBC1 | 0.012447323 | 2.74662937 |
| hsa_circ_0126761_CBC1 | 0.037515214 | 2.282415491 |
| hsa_circ_0047423_CBC1 | 0.01157154 | 2.144084764 |
| hsa_circ_0128235_CBC1 | 0.007529945 | 2.187762856 |
| hsa-circRNA7032-2_CBC1 | 0.001097382 | 2.936237926 |
| hsa_circ_0139052_CBC1 | 0.019150111 | 2.070770246 |
| hsa-circRNA6049-17_CBC1 | 0.015999911 | 2.053397758 |
| hsa-circRNA15292-3_CBC1 | 0.00190752 | 2.352678465 |
| hsa_circ_0055854_CBC1 | 0.000735249 | 2.006306276 |
| hsa_circ_0087207_CBC1 | 0.001987821 | 6.451438246 |
| hsa_circ_0105103_CBC1 | 0.007357858 | 2.033974615 |
| hsa-circRNA3626-2_CBC1 | 0.002295717 | 2.687967265 |
| hsa_circ_0018412_CBC1 | 0.012024191 | 3.04864851 |
| hsa-circRNA14113-15_CBC1 | 0.006736983 | 2.165823407 |
| hsa_circ_0130493_CBC1 | 0.006095213 | 2.007378667 |
| hsa_circ_0018608_CBC1 | 0.043631512 | 2.356126849 |
| hsa_circ_0136496_CBC1 | 0.002800861 | 2.131500733 |
| hsa_circ_0140019_CBC1 | 0.000166042 | 2.028343803 |
| hsa_circ_0055874_CBC1 | 0.000897636 | 2.174584928 |
| hsa_circ_0092689_CBC1 | 0.031591853 | 2.004523547 |
| hsa_circ_0040177_CBC1 | 0.00095966 | 3.286447062 |
| hsa_circ_0002219_CBC1 | 0.000947625 | 2.077418432 |
| hsa_circ_0064671_CBC1 | 0.039314758 | 2.053921837 |
| hsa-circRNA6482-14_CBC1 | 0.028241554 | 3.205119704 |
| hsa_circ_0079537_CBC1 | 0.000186838 | 3.150245126 |
| hsa_circ_0125303_CBC1 | 0.003703766 | 2.01083891 |
| hsa_circ_0064060_CBC1 | 0.014330377 | 2.511778519 |
| hsa_circ_0101419_CBC1 | 0.005060861 | 2.513970539 |
| hsa_circ_0061671_CBC1 | 0.008966801 | 2.054813247 |
| hsa_circ_0036112_CBC1 | 0.011691131 | 2.10094487 |
| hsa-circRNA4925-14_CBC1 | 0.000708331 | 2.418329447 |
| hsa_circ_0049510_CBC1 | 0.003293012 | 2.278521367 |
| hsa-circRNA13692-2_CBC1 | 0.022249309 | 2.231897159 |
| hsa_circ_0093323_CBC1 | 0.008180458 | 2.847658917 |
| hsa-circRNA14918_CBC1 | 0.015574156 | 4.457040846 |
| hsa-circRNA11158-49_CBC1 | 0.000365275 | 3.124171862 |
| hsa_circ_0022549_CBC1 | 0.001574144 | 2.137274872 |
| hsa_circ_0047662_CBC1 | 0.006728836 | 2.074258103 |
| hsa_circ_0025439_CBC1 | 0.014018262 | 2.193596475 |
| hsa_circ_0131757_CBC1 | 0.005509935 | 2.199354632 |
| hsa_circ_0123450_CBC1 | 4.13877E-05 | 2.52638553 |
| hsa_circ_0007970_CBC1 | 0.00816228 | 2.009076074 |
| hsa-circRNA3045-3_CBC1 | 2.17924E-05 | 2.122000245 |
| hsa_circ_0079535_CBC1 | 7.79051E-05 | 2.749978658 |
| hsa-circRNA8952-22_CBC1 | 0.00084085 | 2.5923831 |
| hsa_circ_0040178_CBC1 | 0.000246279 | 3.271142467 |
| hsa_circ_0124379_CBC1 | 0.000129114 | 2.080035481 |
| hsa_circ_0059440_CBC1 | 0.011693148 | 2.434995343 |
| hsa-circRNA2887-2_CBC1 | 0.002331684 | 2.017623039 |
| hsa_circ_0023817_CBC1 | 0.020678695 | 2.259048099 |
| hsa_circ_0084134_CBC1 | 0.001011728 | 2.285754238 |
| hsa_circ_0063717_CBC1 | 0.049946217 | 2.837676666 |
| hsa_circ_0111317_CBC1 | 0.028438244 | 2.424006126 |
| hsa_circ_0089097_CBC1 | 0.04023335 | 2.229226077 |
| hsa_circ_0043623_CBC1 | 0.001799369 | 2.402904201 |
| hsa_circ_0125355_CBC1 | 0.004123757 | 4.877020654 |
| hsa_circ_0108036_CBC1 | 0.002870304 | 2.064187794 |
| hsa-circRNA614-1_CBC1 | 0.015168756 | 2.168053355 |
| hsa_circ_0020829_CBC1 | 0.001394835 | 2.300491239 |
| hsa_circ_0136110_CBC1 | 0.03466832 | 2.150636959 |
| hsa_circ_0025416_CBC1 | 6.19361E-05 | 2.01934197 |
| hsa-circRNA9077-13_CBC1 | 0.001727182 | 2.026523497 |
| hsa_circ_0008572_CBC1 | 0.00409094 | 2.03910409 |
| hsa_circ_0079719_CBC1 | 0.000148676 | 3.828193085 |
| hsa-circRNA9844-5_CBC1 | 0.02553185 | 2.227723508 |
| hsa_circ_0090908_CBC1 | 0.049897681 | 2.604096595 |
| hsa-circRNA7664-4_CBC1 | 0.00166627 | 2.009049318 |
| hsa_circ_0054658_CBC1 | 0.032638936 | 4.097433565 |
| hsa_circ_0059577_CBC1 | 0.003972481 | 2.695980693 |
| hsa_circ_0019064_CBC1 | 0.012582048 | 3.680171113 |
| hsa_circ_0089983_CBC1 | 0.003433307 | 2.014909245 |
| hsa_circ_0127868_CBC1 | 0.026807419 | 2.17132124 |
| hsa_circ_0024334_CBC1 | 0.009120505 | 2.013542205 |
| hsa_circ_0123763_CBC1 | 0.000412607 | 2.274014105 |
| hsa_circ_0066884_CBC1 | 0.002371751 | 2.032339599 |
| hsa-circRNA12825-14_CBC1 | 0.011739693 | 2.050455771 |
| hsa_circ_0004762_CBC1 | 0.008458765 | 2.133395616 |
| hsa-circRNA9227-8_CBC1 | 0.027094742 | 3.751814281 |
| hsa_circ_0108114_CBC1 | 0.005398569 | 2.309913146 |
| hsa-circRNA5391-4_CBC1 | 0.007014961 | 2.357464416 |
| hsa_circ_0033188_CBC1 | 0.019099215 | 2.13006379 |
| hsa_circ_0075020_CBC1 | 0.018877292 | 2.053212471 |
| hsa-circRNA7476_CBC1 | 0.013068342 | 2.191386563 |
| hsa-circRNA11158-47_CBC1 | 7.19845E-05 | 2.910021123 |
| hsa_circ_0020154_CBC1 | 0.001719397 | 2.087014261 |
| hsa-circRNA8800-2_CBC1 | 0.012817579 | 6.177156719 |
| hsa_circ_0065102_CBC1 | 0.001233576 | 2.514007919 |
| hsa_circ_0068461_CBC1 | 0.001087938 | 2.786721806 |
| hsa_circ_0096869_CBC1 | 0.010946969 | 2.977970385 |
| hsa_circ_0084141_CBC1 | 0.000664484 | 2.132177964 |
| hsa_circ_0048179_CBC1 | 0.024750502 | 2.648060928 |
| hsa_circ_0081894_CBC1 | 0.018597826 | 2.261683935 |
| hsa_circ_0078654_CBC1 | 0.004044125 | 2.610539136 |
| hsa-circRNA7032-30_CBC1 | 0.000203348 | 3.607351169 |
| hsa_circ_0140661_CBC1 | 0.020261297 | 2.343318719 |
| hsa-circRNA2747-6_CBC1 | 0.003820272 | 2.049649168 |
| hsa_circ_0103223_CBC1 | 0.001977073 | 2.019173724 |
| hsa_circ_0137129_CBC1 | 0.006754802 | 2.085506682 |
| hsa-circRNA15273-30_CBC1 | 0.002228946 | 2.091653968 |
| hsa_circ_0128616_CBC1 | 0.000628798 | 2.302613182 |
| hsa-circRNA714-4_CBC1 | 0.011781285 | 2.252305618 |
| hsa_circ_0005149_CBC1 | 0.002800395 | 2.642044927 |
| hsa_circ_0045276_CBC1 | 0.045777992 | 2.15594128 |
| hsa_circ_0003917_CBC1 | 0.002497868 | 2.114559896 |
| hsa_circ_0051385_CBC1 | 0.007170997 | 2.223109856 |
| hsa_circ_0025766_CBC1 | 0.006791463 | 6.509057764 |
| hsa-circRNA14308-23_CBC1 | 0.003987595 | 2.323636221 |
| hsa_circ_0068465_CBC1 | 0.008929982 | 2.51393725 |
| hsa_circ_0135688_CBC1 | 0.013778787 | 2.599270198 |
| hsa_circ_0129831_CBC1 | 0.000615277 | 3.18155253 |
| hsa_circ_0087475_CBC1 | 0.003139312 | 2.132474088 |
| hsa_circ_0069449_CBC1 | 0.000812283 | 2.213705001 |
| hsa_circ_0112481_CBC1 | 0.00392935 | 2.002386621 |
| hsa_circ_0135188_CBC1 | 3.99407E-05 | 11.81452999 |
| hsa-circRNA4925-8_CBC1 | 0.000386882 | 2.252018268 |
| hsa_circ_0039521_CBC1 | 7.11902E-06 | 3.281271269 |
| hsa-circRNA1506-17_CBC1 | 0.00607838 | 2.007461886 |
| hsa_circ_0087276_CBC1 | 0.040577532 | 5.854449109 |
| hsa_circ_0067568_CBC1 | 0.001982051 | 2.012422297 |
| hsa-circRNA10069-4_CBC1 | 0.006374271 | 2.052923965 |
| hsa_circ_0025461_CBC1 | 0.00236359 | 2.252625107 |
| hsa-circRNA15925-5_CBC1 | 0.000294995 | 4.12992947 |
| hsa_circ_0125258_CBC1 | 0.000612459 | 2.385475669 |
| hsa_circ_0094351_CBC1 | 1.05967E-05 | 2.012255014 |
| hsa_circ_0098970_CBC1 | 2.27706E-05 | 3.491972852 |
| hsa_circ_0082074_CBC1 | 0.007724376 | 2.077155508 |
| hsa_circ_0131278_CBC1 | 0.016401455 | 3.930345204 |
| hsa_circ_0016487_CBC1 | 0.002522001 | 2.892445923 |
| hsa_circ_0017962_CBC1 | 0.004394349 | 2.716211806 |
| hsa-circRNA15822-36_CBC1 | 0.002418785 | 2.379793054 |
| hsa_circ_0131298_CBC1 | 0.001107714 | 2.260921981 |
| hsa_circ_0015647_CBC1 | 0.000590859 | 2.313843552 |
| hsa_circ_0004462_CBC1 | 0.009117671 | 2.208235281 |
| hsa_circ_0133861_CBC1 | 0.008707814 | 2.495112965 |
| hsa_circ_0021231_CBC1 | 0.008841805 | 2.015681235 |
| hsa_circ_0012909_CBC1 | 0.000521481 | 2.234118714 |
| hsa_circ_0078651_CBC1 | 0.000928248 | 2.89008493 |
| hsa_circ_0023800_CBC1 | 0.000423006 | 2.380973191 |
| hsa_circ_0073207_CBC1 | 0.001147142 | 3.233997966 |
| hsa_circ_0051636_CBC1 | 0.000450005 | 2.182913413 |
| hsa_circ_0073219_CBC1 | 0.003974237 | 3.046566642 |
| hsa_circ_0031318_CBC1 | 0.00398135 | 3.312481192 |
| hsa_circ_0011133_CBC1 | 0.000381096 | 3.40367967 |
| hsa_circ_0125356_CBC1 | 0.000835338 | 9.162637522 |
| hsa_circ_0013766_CBC1 | 9.15976E-05 | 2.758548724 |
| hsa-circRNA14989-2_CBC1 | 0.002939272 | 3.063254652 |
| hsa_circ_0059144_CBC1 | 2.97862E-05 | 13.36618586 |
| hsa_circ_0040181_CBC1 | 0.000129378 | 3.089142279 |
| hsa_circ_0034415_CBC1 | 0.014948706 | 4.38888174 |
| hsa_circ_0137689_CBC1 | 0.006300189 | 2.840316004 |
| hsa_circ_0064512_CBC1 | 0.010816592 | 2.184485573 |
| hsa_circ_0112007_CBC1 | 5.76386E-05 | 2.591697943 |
| hsa_circ_0064084_CBC1 | 0.001555676 | 3.270928576 |
| hsa-circRNA11607-16_CBC1 | 0.000633637 | 2.510010522 |
| hsa_circ_0079730_CBC1 | 0.000576636 | 2.689371809 |
| hsa_circ_0084140_CBC1 | 0.000169819 | 2.278075823 |
| hsa-circRNA9371-9_CBC1 | 0.000813117 | 2.242275753 |
| hsa_circ_0064568_CBC1 | 0.015365326 | 2.075404746 |
| hsa_circ_0139991_CBC1 | 0.040393375 | 4.196105434 |
| hsa_circ_0087470_CBC1 | 0.004106264 | 2.077673239 |
| hsa_circ_0047430_CBC1 | 0.006268137 | 2.992906639 |
| hsa_circ_0115759_CBC1 | 0.000789435 | 2.709216692 |
| hsa_circ_0062880_CBC1 | 0.019667998 | 2.046730734 |
| hsa_circ_0100352_CBC1 | 0.03500775 | 5.496846493 |
| hsa_circ_0079538_CBC1 | 0.018646576 | 3.909629223 |
| hsa-circRNA4883-10_CBC1 | 0.039018399 | 3.762606345 |
| hsa-circRNA7223-6_CBC1 | 0.000674585 | 2.290092839 |
| hsa_circ_0083371_CBC1 | 0.000785773 | 3.693622126 |
| hsa_circ_0129728_CBC1 | 0.034102945 | 2.215178907 |
| hsa_circ_0061387_CBC1 | 0.001877493 | 3.084311239 |
| hsa_circ_0092682_CBC1 | 0.038246129 | 2.416292837 |
| hsa_circ_0055856_CBC1 | 0.00200979 | 2.680960625 |
| hsa_circ_0083821_CBC1 | 0.033588109 | 2.18314309 |
| hsa-circRNA13414-2_CBC1 | 0.00376586 | 2.323205553 |
| hsa_circ_0091723_CBC1 | 0.025085199 | 2.16723371 |
| hsa_circ_0023811_CBC1 | 2.75835E-06 | 2.005927417 |
| hsa_circ_0067567_CBC1 | 0.002734305 | 2.026709805 |
| hsa_circ_0073204_CBC1 | 0.000249971 | 3.055413971 |
| hsa_circ_0134121_CBC1 | 4.9165E-05 | 5.381876909 |
| hsa_circ_0123457_CBC1 | 0.042740249 | 2.189477631 |
| hsa_circ_0083431_CBC1 | 0.022915976 | 4.270596751 |
| hsa-circRNA10544-7_CBC1 | 0.000255822 | 2.065999943 |
| hsa-circRNA816-3_CBC1 | 0.028486032 | 2.528980136 |
| hsa_circ_0103922_CBC1 | 0.007153126 | 3.353007777 |
| hsa_circ_0005022_CBC1 | 0.000225265 | 2.561737204 |
| hsa-circRNA1531-32_CBC1 | 0.009757677 | 3.550049836 |
| hsa_circ_0075062_CBC1 | 0.002353545 | 2.168017674 |
| hsa-circRNA9227-1_CBC1 | 0.004156649 | 3.510267774 |
| hsa-circRNA933-1_CBC1 | 0.012272669 | 2.077825925 |
| hsa_circ_0132003_CBC1 | 0.000552075 | 2.211013025 |
| hsa_circ_0129221_CBC1 | 0.007145086 | 2.040661592 |
| hsa_circ_0113111_CBC1 | 0.040733456 | 2.526847651 |
| hsa-circRNA15875-14_CBC1 | 0.041895665 | 2.487177028 |
| hsa_circ_0055283_CBC1 | 3.90019E-05 | 5.646286216 |
| hsa_circ_0114483_CBC1 | 0.006556967 | 2.120812241 |
| hsa_circ_0001859_CBC1 | 0.002810349 | 2.004244581 |
| hsa-circRNA10096-2_CBC1 | 9.06711E-07 | 5.369463654 |
| hsa_circ_0012945_CBC1 | 0.023058916 | 2.848244717 |
| hsa_circ_0135127_CBC1 | 0.004589958 | 2.193371386 |
| hsa_circ_0075825_CBC1 | 0.000325477 | 2.285877991 |
| hsa_circ_0015329_CBC1 | 0.049567158 | 2.115191016 |
| hsa-circRNA11158-25_CBC1 | 0.000123316 | 2.577633368 |
| hsa-circRNA7021-10_CBC1 | 0.000529035 | 2.414019745 |
| hsa_circ_0072111_CBC1 | 0.002105233 | 2.070609861 |
| hsa-circRNA14298-20_CBC1 | 0.012670892 | 2.760165619 |
| hsa_circ_0059450_CBC1 | 0.003813354 | 2.599357353 |
| hsa_circ_0120845_CBC1 | 0.022005064 | 2.005415465 |
| hsa_circ_0140205_CBC1 | 0.001637622 | 9.59528461 |
| hsa_circ_0003400_CBC1 | 0.001659787 | 2.508237144 |
| hsa-circRNA5151-47_CBC1 | 0.011251125 | 2.085738703 |
| hsa_circ_0123490_CBC1 | 0.048996907 | 2.003599231 |
| hsa_circ_0068458_CBC1 | 0.001552032 | 3.196848656 |
| hsa_circ_0104404_CBC1 | 0.002282839 | 2.21835035 |
| hsa-circRNA5434-5_CBC1 | 0.000651365 | 2.266323349 |
| hsa_circ_0098140_CBC1 | 0.014663605 | 2.330755777 |
| hsa_circ_0101732_CBC1 | 0.049960617 | 2.316704478 |
| hsa_circ_0114956_CBC1 | 0.008069263 | 2.043227779 |
| hsa_circ_0133078_CBC1 | 0.002647668 | 2.242740036 |
| hsa_circ_0033192_CBC1 | 0.000671831 | 3.491458808 |
| hsa_circ_0081985_CBC1 | 0.00946445 | 2.197630377 |
| hsa_circ_0091373_CBC1 | 0.00914076 | 2.039599055 |
| hsa_circ_0007304_CBC1 | 0.006344658 | 2.199041684 |
| hsa_circ_0063878_CBC1 | 0.011080525 | 3.349975607 |
| hsa_circ_0031424_CBC1 | 0.004468896 | 2.109661145 |
| hsa_circ_0026470_CBC1 | 0.02538482 | 2.080947209 |
| hsa_circ_0112011_CBC1 | 0.000246693 | 2.836499566 |
| hsa_circ_0070251_CBC1 | 0.000642465 | 2.065825067 |
| hsa-circRNA8952-20_CBC1 | 0.000223393 | 2.249710965 |
| hsa_circ_0074375_CBC1 | 3.78092E-05 | 2.471846597 |
| hsa_circ_0069994_CBC1 | 0.046800071 | 2.895295519 |
| hsa_circ_0072507_CBC1 | 0.00268682 | 2.070726755 |
| hsa_circ_0060330_CBC1 | 0.000975957 | 2.155411308 |
| hsa_circ_0055861_CBC1 | 0.000806028 | 2.419496266 |
| hsa_circ_0123889_CBC1 | 0.000511831 | 2.5264905 |
| hsa_circ_0023805_CBC1 | 6.75698E-05 | 2.272544813 |
| hsa_circ_0081196_CBC1 | 4.37801E-05 | 14.08616734 |
| hsa_circ_0132618_CBC1 | 0.00163198 | 2.595057547 |
| hsa-circRNA11776-22_CBC1 | 0.010866574 | 2.104856092 |
| hsa_circ_0135684_CBC1 | 0.005615984 | 2.200698475 |
| hsa_circ_0043618_CBC1 | 0.018804128 | 2.36662423 |
| hsa_circ_0069766_CBC1 | 0.024731713 | 2.113225746 |
| hsa-circRNA15822-57_CBC1 | 0.003819909 | 2.114091571 |
| hsa-circRNA7418-4_CBC1 | 0.028042429 | 2.38898021 |
| hsa_circ_0011436_CBC1 | 0.000779755 | 2.227020295 |
| hsa_circ_0023813_CBC1 | 0.000102385 | 2.335295032 |
| hsa-circRNA10585-20_CBC1 | 0.000344591 | 2.404269422 |
| hsa-circRNA14076-2_CBC1 | 0.001870585 | 2.084367795 |
| hsa-circRNA475-6_CBC1 | 0.000458812 | 3.575713374 |
| hsa_circ_0100544_CBC1 | 0.023517402 | 2.793369213 |
| hsa_circ_0079801_CBC1 | 0.0088943 | 2.418978589 |
| hsa_circ_0006916_CBC1 | 0.000122917 | 2.214124827 |
| hsa-circRNA15273-18_CBC1 | 5.14321E-05 | 2.379261954 |
| hsa_circ_0059903_CBC1 | 0.019826948 | 2.112378431 |
| hsa-circRNA13044_CBC1 | 0.008331869 | 3.46774668 |
| hsa_circ_0068492_CBC1 | 0.01567446 | 2.109586618 |
| hsa-circRNA14293-18_CBC1 | 0.001739129 | 2.03321763 |
| hsa-circRNA8578-1_CBC1 | 0.011496147 | 2.103539479 |
| hsa_circ_0099732_CBC1 | 3.16648E-05 | 2.02371726 |
| hsa_circ_0096525_CBC1 | 4.09203E-05 | 2.158673002 |
| hsa-circRNA14301-2_CBC1 | 0.026504351 | 2.914402312 |
| hsa_circ_0099729_CBC1 | 0.004128146 | 2.467668683 |
| hsa_circ_0115524_CBC1 | 0.013725444 | 2.514654284 |
| hsa_circ_0101695_CBC1 | 0.001277429 | 2.071754517 |
| hsa_circ_0017032_CBC1 | 0.021878807 | 2.038280862 |
| hsa_circ_0014417_CBC1 | 0.030352599 | 2.067062469 |
| hsa_circ_0000530_CBC1 | 0.014852587 | 2.188827291 |
| hsa-circRNA11158-63_CBC1 | 0.000552878 | 2.700884681 |
| hsa_circ_0016484_CBC1 | 0.002542909 | 2.515113453 |
| hsa_circ_0022587_CBC1 | 0.000123629 | 4.899516335 |
| hsa-circRNA475-5_CBC1 | 0.000492114 | 2.420915358 |
| hsa_circ_0016482_CBC1 | 0.000206056 | 2.595075076 |
| hsa_circ_0013176_CBC1 | 0.003258481 | 2.20270472 |
| hsa-circRNA9900-24_CBC1 | 0.004627597 | 2.267467502 |
| hsa_circ_0080257_CBC1 | 0.000171535 | 2.354461127 |
| hsa_circ_0110691_CBC1 | 0.000344845 | 2.766600112 |
| hsa_circ_0027973_CBC1 | 1.08616E-05 | 4.457720698 |
| hsa-circRNA2988-18_CBC1 | 0.00083754 | 2.016201252 |
| hsa_circ_0114886_CBC1 | 0.01241457 | 2.14419693 |
| hsa-circRNA5132-5_CBC1 | 0.001737043 | 2.008385303 |
| hsa_circ_0124601_CBC1 | 0.002006216 | 2.489611336 |
| hsa_circ_0089108_CBC1 | 0.01121845 | 2.100870252 |
| hsa_circ_0078187_CBC1 | 0.007020154 | 2.797315721 |
| hsa_circ_0126617_CBC1 | 0.01075207 | 2.573853703 |
| hsa_circ_0066797_CBC1 | 0.002934741 | 9.000249162 |
| hsa_circ_0070369_CBC1 | 0.029507044 | 2.235880303 |
| hsa_circ_0098181_CBC1 | 0.001016312 | 2.454333187 |
| hsa_circ_0084136_CBC1 | 0.002307587 | 2.346278544 |
| hsa_circ_0140517_CBC1 | 0.002395337 | 2.12110738 |
| hsa-circRNA4115-1_CBC1 | 0.009531005 | 3.524277098 |
| hsa_circ_0128190_CBC1 | 5.63533E-05 | 3.151647062 |
| hsa_circ_0007156_CBC1 | 0.015075109 | 3.069259494 |
| hsa_circ_0119531_CBC1 | 8.13927E-05 | 2.546009356 |
| hsa_circ_0032842_CBC1 | 0.000265784 | 2.022614494 |
| hsa_circ_0025411_CBC1 | 0.005081898 | 2.024014697 |
| hsa-circRNA4925-18_CBC1 | 1.17122E-05 | 2.203717753 |
| hsa_circ_0040186_CBC1 | 0.000160456 | 3.259294654 |
| hsa_circ_0129838_CBC1 | 0.005894535 | 2.880538376 |
| hsa-circRNA6235-2_CBC1 | 0.007495926 | 2.134879245 |
| hsa_circ_0104718_CBC1 | 0.038782115 | 2.458935165 |
| hsa_circ_0120814_CBC1 | 0.002894573 | 2.115331892 |
| hsa-circRNA6092-1_CBC1 | 0.004688727 | 2.314061549 |
| hsa_circ_0022548_CBC1 | 0.003585665 | 2.015922253 |
| hsa-circRNA8604-7_CBC1 | 0.006831192 | 2.121491296 |
| hsa_circ_0036111_CBC1 | 9.21762E-05 | 2.028695076 |
| hsa-circRNA10544-15_CBC1 | 0.011492361 | 2.010689721 |
| hsa_circ_0139983_CBC1 | 0.012980129 | 3.073646028 |
| hsa_circ_0096893_CBC1 | 0.04798181 | 2.510352152 |
| hsa_circ_0124314_CBC1 | 0.021217162 | 2.447028382 |
| hsa-circRNA1123-11_CBC1 | 2.29219E-05 | 2.075429835 |
| hsa_circ_0081365_CBC1 | 0.033278112 | 2.536392497 |
| hsa_circ_0091384_CBC1 | 0.008921363 | 3.084450674 |
| hsa-circRNA15050_CBC1 | 0.010471883 | 9.530737803 |
| hsa_circ_0134115_CBC1 | 1.99363E-05 | 3.517671875 |
| hsa_circ_0118402_CBC1 | 0.004986417 | 2.37439664 |
| hsa_circ_0107895_CBC1 | 0.001023163 | 2.120372429 |
| hsa_circ_0040162_CBC1 | 0.000136349 | 3.086468373 |
| hsa-circRNA10054-1_CBC1 | 0.002568619 | 5.444284942 |
| hsa_circ_0100547_CBC1 | 0.006314187 | 2.393097193 |
| hsa_circ_0047300_CBC1 | 0.00050208 | 2.620936367 |
| hsa_circ_0075820_CBC1 | 0.020337349 | 2.226623895 |
| hsa_circ_0055546_CBC1 | 0.004957212 | 2.125495435 |
| hsa_circ_0066129_CBC1 | 0.005362016 | 2.135133188 |
| hsa_circ_0000099_CBC1 | 0.004474116 | 2.904072672 |
| hsa_circ_0085323_CBC1 | 0.005216922 | 2.498285365 |
| hsa-circRNA1950-17_CBC1 | 0.000453784 | 2.042906566 |
| hsa_circ_0140212_CBC1 | 0.008169385 | 2.171947864 |
| hsa_circ_0084519_CBC1 | 9.43348E-05 | 2.716691264 |
| hsa_circ_0068487_CBC1 | 0.030521481 | 5.385984018 |
| hsa_circ_0024330_CBC1 | 0.031932364 | 3.267861007 |
| hsa_circ_0101542_CBC1 | 0.002795097 | 2.023207483 |
| hsa_circ_0052419_CBC1 | 0.000477131 | 2.554530698 |
| hsa-circRNA11226-8_CBC1 | 4.07941E-06 | 5.819199957 |
| hsa_circ_0128519_CBC1 | 0.029373908 | 4.734062145 |
| hsa_circ_0054209_CBC1 | 0.00181502 | 2.042327259 |
| hsa_circ_0128876_CBC1 | 0.001616918 | 2.051852772 |
| hsa_circ_0137187_CBC1 | 0.000669678 | 2.3396598 |
| hsa_circ_0069451_CBC1 | 0.001743325 | 2.145298501 |
| hsa-circRNA6689-10_CBC1 | 0.006167169 | 2.230903651 |
| hsa_circ_0070943_CBC1 | 0.003201338 | 2.065837963 |
| hsa_circ_0120134_CBC1 | 0.000516547 | 2.396104318 |
| hsa_circ_0018215_CBC1 | 0.035189112 | 2.011845827 |
| hsa-circRNA13228-2_CBC1 | 0.033328485 | 3.120429826 |
| hsa_circ_0011438_CBC1 | 0.036603225 | 2.385286581 |
| hsa_circ_0131297_CBC1 | 0.00710469 | 2.392716257 |
| hsa_circ_0134908_CBC1 | 0.00599528 | 2.740272104 |
| hsa_circ_0043393_CBC1 | 0.004924135 | 2.011975714 |
| hsa_circ_0093638_CBC1 | 0.000926723 | 2.051968492 |
| hsa-circRNA9077-15_CBC1 | 0.005942174 | 2.085261662 |
| hsa_circ_0045288_CBC1 | 0.036457254 | 2.297202177 |
| hsa_circ_0077668_CBC1 | 1.35986E-05 | 4.143857938 |
| hsa_circ_0054973_CBC1 | 0.002328446 | 2.321953287 |
| hsa_circ_0003194_CBC1 | 0.000229991 | 3.155094831 |
| hsa_circ_0126777_CBC1 | 0.014462152 | 2.8460639 |
| hsa_circ_0088565_CBC1 | 0.000101381 | 4.726856721 |
| hsa-circRNA15091_CBC1 | 0.011183017 | 2.061116028 |
| hsa-circRNA8952-17_CBC1 | 5.28772E-05 | 2.836529469 |
| hsa-circRNA7514-5_CBC1 | 0.001163998 | 2.102225915 |
| hsa_circ_0122758_CBC1 | 0.035142918 | 2.278778428 |
| hsa-circRNA735-7_CBC1 | 5.96832E-05 | 2.067311297 |
| hsa_circ_0035195_CBC1 | 0.007959447 | 2.017307453 |
| hsa_circ_0138432_CBC1 | 0.000387286 | 2.154509017 |
| hsa_circ_0018681_CBC1 | 0.000158613 | 4.586403075 |
| hsa_circ_0074142_CBC1 | 0.00184427 | 2.386278428 |
| hsa_circ_0051626_CBC1 | 3.00519E-05 | 2.443198284 |
| hsa_circ_0072560_CBC1 | 0.005835851 | 2.680269901 |
| hsa_circ_0073422_CBC1 | 0.014973261 | 2.266846291 |
| hsa_circ_0129836_CBC1 | 0.002077852 | 3.515596465 |
| hsa-circRNA6782-1_CBC1 | 0.001350255 | 2.38476938 |
| hsa_circ_0053926_CBC1 | 0.022878747 | 2.069489796 |
| hsa_circ_0096524_CBC1 | 0.000639373 | 2.174563926 |
| hsa_circ_0086455_CBC1 | 4.04026E-05 | 2.655490966 |
| hsa_circ_0112561_CBC1 | 0.003670666 | 2.163294821 |
| hsa_circ_0105645_CBC1 | 0.001090919 | 3.187576737 |
| hsa_circ_0079751_CBC1 | 3.65058E-05 | 5.064162743 |
| hsa_circ_0064417_CBC1 | 0.04291455 | 2.843159783 |
| hsa_circ_0027417_CBC1 | 0.004934616 | 3.895253304 |
| hsa-circRNA9089-3_CBC1 | 0.005076484 | 3.475498131 |
| hsa_circ_0002732_CBC1 | 0.003943095 | 2.149356551 |
| hsa_circ_0040796_CBC1 | 0.005280465 | 5.903497312 |
| hsa_circ_0078308_CBC1 | 0.000989777 | 4.311259313 |
| hsa_circ_0087273_CBC1 | 4.27015E-05 | 8.631758269 |
| hsa_circ_0084520_CBC1 | 0.006221475 | 3.009329573 |
| hsa_circ_0018682_CBC1 | 0.000429815 | 6.483916878 |
| hsa_circ_0138716_CBC1 | 6.22877E-05 | 2.948824369 |
| hsa_circ_0055061_CBC1 | 0.001059966 | 2.063477559 |
| hsa-circRNA1364-9_CBC1 | 0.006084043 | 2.128264781 |
| hsa_circ_0113876_CBC1 | 0.00506222 | 2.24920487 |
| hsa_circ_0070944_CBC1 | 0.00046802 | 2.349519837 |
| hsa_circ_0087496_CBC1 | 0.000623727 | 2.153860784 |
| hsa-circRNA14469-23_CBC1 | 0.009723163 | 2.236898257 |
| hsa_circ_0127223_CBC1 | 0.010185786 | 2.285962997 |
| hsa_circ_0063394_CBC1 | 0.003145119 | 2.986608686 |
| hsa_circ_0136234_CBC1 | 0.004877055 | 2.000046004 |
| hsa_circ_0027418_CBC1 | 0.000127711 | 3.719891931 |
| hsa_circ_0119741_CBC1 | 0.020639312 | 4.051666977 |
| hsa_circ_0089103_CBC1 | 0.023588971 | 2.490416501 |
| hsa_circ_0084517_CBC1 | 0.000896638 | 2.915670371 |
| hsa-circRNA13203-2_CBC1 | 0.006368796 | 2.42021196 |
| hsa_circ_0073205_CBC1 | 0.001790317 | 2.962013175 |
| hsa_circ_0084797_CBC1 | 0.012632571 | 2.032947325 |
| hsa_circ_0139342_CBC1 | 0.001177063 | 2.053068334 |
| hsa_circ_0098324_CBC1 | 0.000140307 | 3.587347505 |
| hsa_circ_0045272_CBC1 | 0.030289693 | 2.109224384 |
| hsa_circ_0101937_CBC1 | 0.018303345 | 2.109086561 |
| hsa_circ_0077770_CBC1 | 0.025873672 | 4.75115624 |
| hsa_circ_0133081_CBC1 | 0.003890975 | 2.223070091 |
| hsa_circ_0007155_CBC1 | 0.001675726 | 2.131812275 |
| hsa_circ_0125473_CBC1 | 0.001600047 | 2.581515429 |
| hsa_circ_0086480_CBC1 | 0.0277701 | 2.620516636 |
| hsa_circ_0134749_CBC1 | 0.023036241 | 2.027191504 |
| hsa_circ_0133928_CBC1 | 0.03163175 | 2.572553066 |
| hsa-circRNA8991-45_CBC1 | 0.000916313 | 2.254454815 |
| hsa_circ_0139336_CBC1 | 0.001250083 | 2.590776803 |
| hsa_circ_0125257_CBC1 | 0.011930087 | 2.778087121 |
| hsa_circ_0108310_CBC1 | 0.000536574 | 2.289746782 |
| hsa-circRNA6482-8_CBC1 | 0.000318848 | 2.8879088 |
| hsa_circ_0072773_CBC1 | 0.000213256 | 2.462603907 |
| hsa-circRNA5118-2_CBC1 | 0.001203076 | 2.220314456 |
| hsa_circ_0081893_CBC1 | 0.03264341 | 2.167506938 |
| hsa_circ_0139345_CBC1 | 1.32896E-05 | 2.052426379 |
| hsa_circ_0025445_CBC1 | 0.00923871 | 2.099707502 |
| hsa-circRNA8604-4_CBC1 | 0.001765273 | 2.570279274 |
| hsa_circ_0096896_CBC1 | 0.001626122 | 2.470414438 |
| hsa-circRNA1545-1_CBC1 | 0.004754198 | 2.563425597 |
| hsa-circRNA12390-3_CBC1 | 0.009470587 | 2.032613404 |
| hsa_circ_0073203_CBC1 | 0.001164846 | 3.312376897 |
| hsa_circ_0098315_CBC1 | 0.005835547 | 4.246808416 |
| hsa-circRNA12552_CBC1 | 0.003512575 | 2.052744045 |
| hsa_circ_0140202_CBC1 | 0.00868398 | 4.540553378 |
| hsa_circ_0006963_CBC1 | 0.005292733 | 2.604404738 |
| hsa_circ_0008679_CBC1 | 0.002839994 | 2.136422458 |
| hsa_circ_0073208_CBC1 | 0.001685668 | 3.420744133 |
| hsa_circ_0092192_CBC1 | 0.022996672 | 2.113500344 |
| hsa_circ_0054449_CBC1 | 0.023714422 | 2.203278936 |
| hsa-circRNA14293-10_CBC1 | 0.01389207 | 2.18706333 |
| hsa_circ_0112999_CBC1 | 0.011708153 | 2.097235148 |
| hsa-circRNA8868-3_CBC1 | 0.002390218 | 2.02013073 |
| hsa_circ_0138800_CBC1 | 0.019942531 | 2.177203265 |
| hsa_circ_0011168_CBC1 | 0.007255381 | 2.577482361 |
| hsa_circ_0025594_CBC1 | 0.019701704 | 2.102453987 |
| hsa_circ_0098626_CBC1 | 0.006246521 | 2.11383803 |
| hsa_circ_0129869_CBC1 | 0.006490922 | 2.167972201 |
| hsa_circ_0011726_CBC1 | 0.004565621 | 2.051312812 |
| hsa_circ_0082691_CBC1 | 0.00037003 | 2.7533763 |
| hsa_circ_0040189_CBC1 | 3.87961E-05 | 2.727749244 |
| hsa_circ_0075617_CBC1 | 0.002111968 | 2.772987725 |
| hsa_circ_0126616_CBC1 | 0.000283211 | 2.565115828 |
| hsa-circRNA1853-2_CBC1 | 0.00285747 | 3.568993144 |
| hsa_circ_0055265_CBC1 | 0.017004546 | 2.618239065 |
| hsa_circ_0015286_CBC1 | 0.002195605 | 2.281236082 |
| hsa_circ_0045284_CBC1 | 0.002040656 | 2.539139629 |
| hsa_circ_0112998_CBC1 | 0.003447301 | 2.016033502 |
| hsa_circ_0108316_CBC1 | 0.000174035 | 2.055977581 |
| hsa_circ_0087270_CBC1 | 0.002673261 | 4.495480426 |
| hsa-circRNA15822-37_CBC1 | 0.000538731 | 2.326523032 |
| hsa_circ_0131282_CBC1 | 0.001712727 | 2.50475204 |
| hsa-circRNA4188-1_CBC1 | 0.003625426 | 2.115884526 |
| hsa-circRNA4561-21_CBC1 | 0.00117976 | 2.668903666 |
| hsa_circ_0087473_CBC1 | 0.000903374 | 2.135603339 |
| hsa_circ_0087503_CBC1 | 2.85422E-05 | 2.120626456 |
| hsa_circ_0079729_CBC1 | 0.000302018 | 3.310493969 |
| hsa_circ_0070903_CBC1 | 0.000476072 | 2.98377273 |
| hsa_circ_0014680_CBC1 | 0.000232928 | 2.21030183 |
| hsa_circ_0079488_CBC1 | 0.007366875 | 2.18098311 |
| hsa_circ_0086349_CBC1 | 0.004347047 | 3.536521 |
| hsa-circRNA513-2_CBC1 | 0.000141297 | 2.246510779 |
| hsa_circ_0140107_CBC1 | 0.000819971 | 2.544639006 |
| hsa-circRNA874-3_CBC1 | 0.005107967 | 2.33628682 |
| hsa_circ_0119529_CBC1 | 0.020632241 | 2.205461773 |
| hsa_circ_0004555_CBC1 | 0.008608855 | 2.081174881 |
| hsa_circ_0074400_CBC1 | 0.006813671 | 2.088998012 |
| hsa_circ_0043613_CBC1 | 0.011771316 | 3.460112827 |
| hsa-circRNA15822-63_CBC1 | 0.001296171 | 2.258945584 |
| hsa_circ_0074155_CBC1 | 0.004429248 | 2.179897438 |
| hsa-circRNA10308-10_CBC1 | 0.001748726 | 2.043795947 |
| hsa_circ_0007180_CBC1 | 0.004156115 | 2.35535969 |
| hsa_circ_0051381_CBC1 | 0.001617726 | 2.222594482 |
| hsa_circ_0047271_CBC1 | 2.80729E-05 | 2.108212742 |
| hsa-circRNA14468-2_CBC1 | 0.00985636 | 2.58542791 |
| hsa_circ_0064337_CBC1 | 0.005218127 | 2.352809687 |
| hsa_circ_0023802_CBC1 | 0.000304681 | 2.110639335 |
| hsa_circ_0096527_CBC1 | 0.002709927 | 2.383097791 |
| hsa_circ_0138746_CBC1 | 0.007897233 | 2.358450474 |
| hsa_circ_0134297_CBC1 | 0.010506257 | 2.260389525 |
| hsa_circ_0022557_CBC1 | 0.001464908 | 2.156453657 |
| hsa_circ_0110274_CBC1 | 0.008127253 | 2.366932857 |
| hsa_circ_0071015_CBC1 | 0.000148505 | 2.069557231 |
| hsa_circ_0130233_CBC1 | 0.010597103 | 2.461731059 |
| hsa-circRNA14298-11_CBC1 | 0.015356578 | 3.376605484 |
| hsa-circRNA1363-8_CBC1 | 0.000145984 | 3.569908978 |
| hsa-circRNA345-3_CBC1 | 0.00507543 | 2.31809434 |
| hsa-circRNA5409-11_CBC1 | 0.02155276 | 2.426231033 |
| hsa_circ_0104393_CBC1 | 0.008484332 | 2.145535656 |
| hsa_circ_0016532_CBC1 | 0.023457555 | 2.021842624 |
| hsa_circ_0047786_CBC1 | 0.023302821 | 2.025334158 |
| hsa_circ_0105657_CBC1 | 0.000817288 | 2.084111133 |
| hsa_circ_0116893_CBC1 | 0.000754236 | 2.601402497 |
| hsa_circ_0055867_CBC1 | 0.00641525 | 2.433396984 |
| hsa_circ_0118386_CBC1 | 0.044915163 | 2.353043278 |
| hsa-circRNA9900-15_CBC1 | 0.001868556 | 2.240293984 |
| hsa-circRNA12223_CBC1 | 0.005178533 | 2.104601324 |
| hsa_circ_0039520_CBC1 | 0.003523581 | 3.273347144 |
| hsa_circ_0051384_CBC1 | 0.011134257 | 2.244653032 |
| hsa_circ_0112723_CBC1 | 4.64569E-05 | 2.121514893 |
| hsa_circ_0132159_CBC1 | 0.014524243 | 2.146909056 |
| hsa_circ_0012907_CBC1 | 0.000928711 | 2.423578858 |
| hsa_circ_0014180_CBC1 | 0.006396382 | 2.074727809 |
[truncated: 878,637 more chars]
